# Repositioning VU‐0365114 as a novel microtubule‐destabilizing agent for treating cancer and overcoming drug resistance

**DOI:** 10.1002/1878-0261.13536

**Published:** 2023-10-22

**Authors:** Yao‐Yu Hsieh, Jia‐Ling Du, Pei‐Ming Yang

**Affiliations:** ^1^ Division of Hematology and Oncology Taipei Medical University Shuang Ho Hospital New Taipei City Taiwan; ^2^ Division of Hematology and Oncology, Department of Internal Medicine, School of Medicine, College of Medicine Taipei Medical University Taipei Taiwan; ^3^ Taipei Cancer Center Taipei Medical University Taipei Taiwan; ^4^ TMU and Affiliated Hospitals Pancreatic Cancer Groups Taipei Medical University Taipei Taiwan; ^5^ Graduate Institute of Cancer Biology and Drug Discovery, College of Medical Science and Technology Taipei Medical University New Taipei City Taiwan; ^6^ Ph.D. Program for Cancer Molecular Biology and Drug Discovery, College of Medical Science and Technology Taipei Medical University New Taipei City Taiwan; ^7^ TMU Research Center of Cancer Translational Medicine Taipei Taiwan; ^8^ Cancer Center, Wan Fang Hospital Taipei Medical University Taipei Taiwan

**Keywords:** colorectal cancer, connectivity map, drug repositioning, drug resistance, microtubule‐targeting agent, polypharmacology

## Abstract

Microtubule‐targeting agents represent one of the most successful classes of anticancer agents. However, the development of drug resistance and the appearance of adverse effects hamper their clinical implementation. Novel microtubule‐targeting agents without such limitations are urgently needed. By employing a gene expression‐based drug repositioning strategy, this study identifies VU‐0365114, originally synthesized as a positive allosteric modulator of human muscarinic acetylcholine receptor M5 (M5 mAChR), as a novel type of tubulin inhibitor by destabilizing microtubules. VU‐0365114 exhibits a broad‐spectrum *in vitro* anticancer activity, especially in colorectal cancer cells. A tumor xenograft study in nude mice shows that VU‐0365114 slowed the *in vivo* colorectal tumor growth. The anticancer activity of VU‐0365114 is not related to its original target, M5 mAChR. In addition, VU‐0365114 does not serve as a substrate of multidrug resistance (MDR) proteins, and thus, it can overcome MDR. Furthermore, a kinome analysis shows that VU‐0365114 did not exhibit other significant off‐target effects. Taken together, our study suggests that VU‐0365114 primarily targets microtubules, offering potential for repurposing in cancer treatment, although more studies are needed before further drug development.

## Graphical abstract

Microtubule‐targeting agents combat cancer, but resistance and adverse effects hinder their use. Employing gene expression‐based repositioning, we identified VU‐0365114 as a microtubule‐destabilizing agent. It shows broad‐spectrum anticancer activity, particularly in colorectal cancer cells. VU‐0365114 overcomes multidrug resistance (MDR) and lacks significant off‐target effects. Repurposing VU‐0365114 holds promise for cancer treatment and resistance reversal.

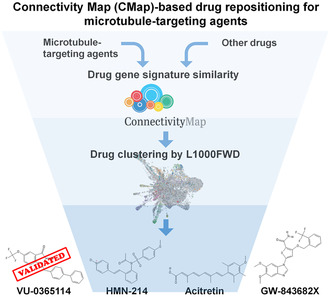

**Abbreviations**
ABC: ATP‐binding cassette
BCRP: breast cancer‐resistance protein
CAMK1D: calcium/calmodulin‐dependent protein kinase ID
CBSI: colchicine‐binding site inhibitor
CCNB1: cyclin B1
CDK1: cyclin‐dependent kinase 1
CETSA: cellular thermal shift assay
CMap: Connectivity Map
CREB: cAMP responsive element‐binding protein
CYP: cytochrome P450
EGFP: enhanced green fluorescent protein
FOB: functional observational battery
IGF1R: insulin‐like growth factor 1 receptor
KNL1: kinetochore scaffold 1
mAChR: muscarinic acetylcholine receptor
MDR1: monopolar spindle 1
MDR1: multidrug‐resistance protein 1
MOAs: mechanisms of action
MPS1: MDR multidrug resistance
MRP: multidrug‐resistance‐associated protein
NCI: National Cancer Institute
Op18: oncoprotein 18
PARP: poly(ADP ribose) polymerase
PCC: Pearson's correlation coefficient
P‐gp: P‐glycoprotein
SAC: spindle‐assembly checkpoint
STMN1: stathmin 1

## 1 Introduction

The process of drug discovery is still risky, lengthy, and costly, and only a few new anticancer drugs receive approval in recent years [1]. Drug repositioning or drug repurposing is a novel strategy for drug discovery that uses existing therapeutic drugs to investigate their new disease indications. Even shelved drugs that never enter or are failed during clinical trials might be rescued if novel mechanisms of action (MOAs) or targets are identified [2]. Polypharmacology describes the ability of a drug to affect more than one molecular target, which is now commonly accepted as a basic property of small molecules and serves as a foundational principle for drug repositioning. Exploring polypharmacology is highly useful for discovering drug's novel MOAs to improve their therapeutic efficacies and treat different diseases [3]. The advantages of drug repositioning include the reduction of costs and the bypass of safety concerns because extensive drug data are often available [2].

Mutations in cancer cells frequently result in cell‐cycle alterations that lead to unrestricted growth. Accordingly, many drugs have been developed to inhibit different cell‐cycle phases [4]. Polymerization of heterodimers of α/β‐tubulins forms microtubule polymers that are essential for mitosis and cell division. Drugs that target microtubules belong to a successful class of anticancer agents that have been utilized for the past two decades [5]. Two major types of microtubule‐targeting agents are microtubule stabilizers and destabilizers. Microtubule‐stabilizing agents (such as taxanes and epothilones) bind to microtubule polymers and stabilize them against disassembly. In contrast, microtubule‐destabilizing agents (such as colchicine and vinca alkaloids) bind to tubulin dimers and destabilize microtubule polymers [5]. Six tubulin‐binding sites for microtubule‐targeting agents have been discovered, including taxane and laulimalide/peloruside sites for microtubule stabilizers, and vinca, colchicine, maytansine, and pironetin sites for microtubule destabilizers [6, 7]. Most binding sites are located within β‐tubulin and only the pironetin site is within α‐tubulin [7]. By binding and perturbing microtubule dynamics during mitosis, microtubule‐targeting agents activate the spindle‐assembly checkpoint (SAC) and causes mitotic cell death [8].

Connectivity Map (CMap) gains popularity in recent year for exploring the functional relationships among drugs, genes, and diseases. CMap allows researchers to compare the gene expression profiles of cells that have been treated with different drugs, providing insights into the biological effects of those drugs and potential new therapeutic applications. CMap has been used in various studies to identify potential drug targets, predict drug responses, and uncover novel mechanisms of disease [9]. The pilot CMap database was established in 2006 by treating three cancer cell lines with 164 compounds and then collecting Affymetrix GeneChip microarray gene expression profiles [10]. The small scale of the CMap pilot dataset limits its utility and accuracy due to the lacking of a diversity of chemical and genetic perturbations as well as a diversity of cell types. In addition, the expansion of CMap dataset is limited by the high costs of commercial microarray and RNA‐Seq techniques. Therefore, a new approach based on the Luminex bead array, called L1000 platform, was developed by measuring 1000 landmark transcripts that are sufficient to recover 81% of the entire transcriptome [11]. Based on this relatedly inexpensive and rapid high‐throughput gene expression profiling technology, the next‐generation CMap database, launched in 2017, collects more than 30 000 chemical and 9000 genetic perturbations in nine cell lines [11].

In this study, we employed the next‐generation L1000‐based CMap to repurpose VU‐0365114 as a novel microtubule‐destabilizing agent with potent anticancer activity and the ability to overcome multidrug resistance (MDR), especially in colorectal cancer. A further kinome analysis indicated that VU‐0365114 did not exhibit other significant off‐target effects. Therefore, our results identify that microtubule is the major target of VU‐0365114 for treating cancers and overcoming MDR.

## 2 Materials and methods

### 2.1 Bioinformatics resources

For the CMap analyses, the online “Touchstone” tool at the CMap website (https://clue.io/) [11] was used to directly explore connections of gene signatures of drugs and/or gene knockdown. L1000FWD was developed based on CMap data and further provides visualization of drug‐induced gene signatures and their similarities in mechanisms of action (MOAs) [12]. Alternatively, differentially expressed genes from drug‐treated samples were queried to compare with internal gene signatures in the CMap database. The inputted gene numbers for a CMap query were between 10 and 150 genes. Hierarchical clustering of drug structures was performed using the ChemBioServer (https://chembioserver.vi‐seem.eu/) [13]. The Soergel (Tanimoto Coefficient) distance and Ward clustering linkage were selected, and the clustering threshold was defined as 0.5. Protein expressions in human normal and tumor tissues were obtained from the Human Proteome Map (https://www.humanproteomemap.org/) and Human Protein Atlas (https://www.proteinatlas.org/) [14, 15]. Protein–protein networks were constructed using the STRING database (version 11.5: https://string‐db.org/) [16]. We set the following parameters: organism: homo sapiens; network style: full STRING network; meaning of network edges: evidence; active interaction sources: experiments and databases; minimum required interaction score: 0.4; max number of interactors to show: query proteins only; network display option: hide disconnected nodes in the network. Gene expression data in NCI‐60 cancer cell lines were obtained from the CellMinerCDB (https://discover.nci.nih.gov/cellminercdb/) [17].

### 2.2 Chemicals, reagents, and antibodies

Actinomycin D (#11421) and cycloheximide (#14126) were purchased from Cayman Chemical (Ann Arbor, MI, USA). Verapamil (#ab120140) was purchased from Abcam (Cambridge, MA, USA). Paclitaxel (#TXD01) and purified tubulin protein (#T240‐A) were purchased from Cytoskeleton (Denver, CO, USA). 5‐Fluorouracil (5‐FU) was from Merck Millipore (Billerica, MA, USA). Dasatinib (#D‐3307), doxorubicin (#D‐4000), and vinblastine (#V‐7300) were purchased from LC Laboratories (Woburn, MA, USA). VU‐0365114 (#HY‐107651), GW‐843682X (#HY‐11003), ML‐380 (#HY‐12439), VU‐0238429 (#HY‐12157), Ro3306 (#HY‐12529), and AZ3146 (#HY‐14710) were purchased from MedChemExpress (Monmouth Junction, NJ, USA). Colchicine (#A10238), cevipabulin (#A12591), HMN‐214 (#A10452), acitretin (#A10035), MG132 (#A11043), and oxaliplatin (#A10346) were purchased from Adooq BioScience (Irvine, CA, USA). Apigenin (#A3145), nocodazole (#M1404), dimethyl sulfoxide (DMSO) (D5879), and 3‐(4,5‐dimethylthiazol‐2‐yl)‐2,5‐diphenyl tetrazolium bromide (MTT) (M2128) were purchased from Sigma‐Aldrich (St. Louis, MO, USA). Ponceau S solution (#3342701) was purchased from SERVA (Heidelberg, Germany). Roswell Park Memorial Institute (RPMI)‐1640 (#22400071), Dulbecco's modified Eagle medium (DMEM) (#11965084), L‐glutamine (#25030081), sodium pyruvate (#11360070), glucose (#15023021), antibiotic‐antimycotic solution (penicillin G, streptomycin, and amphotericin B) (#15240062), non‐essential amino acids (#11140050), trypsin–EDTA (#15400054), Opti‐minimum essential medium (MEM) (#31985062), radioimmunoprecipitation (RIPA) buffer (#89901), and BODIPY FL‐conjugated vinblastine (#V12390) were purchased from Thermo Fisher Scientific (Waltham, MA, USA). Fetal bovine serum (FBS) (#35‐010‐CV), phosphate‐buffered saline (PBS) (#46‐013‐CM), and Matrigel (#35428) were purchased from Corning (Corning, NY, USA). The caspase‐3 (#IMG‐144A) antibody was purchased from Imgenex (San Diego, CA, USA). The p53 (#sc‐126) antibody was purchased from Santa Cruz Biotechnology (Santa Cruz, CA, USA). The CHRM5 (#A5367) antibody was purchased from ABclonal (Wuhan, China). The β‐tubulin (#IR1‐2) antibody was purchased from iReal Biotechnology (HsinChu, Taiwan). PARP (#9542), phosphorylated (phospho)‐Ser139‐histone H2AX (#2577S), and phospho‐Thr943/Thr1155‐KNL1 (#40758) were purchased from Cell Signaling Technology (Beverly, MA, USA). Cyclin B1 (#ab72), phospho‐Ser10‐histone H3 (#ab5176), CHRM5 (#ab150531), and P‐glycoprotein (#ab170904) antibodies were purchased from Abcam. CDK1 (#GTX108120), phospho‐Tyr15‐CDK1 (#GTX128155), phospho‐Thr161‐CDK1 (#GTX38597), stathmin 1 (#GTX104704), phospho‐Ser38‐stathmin 1 (#GTX50261), phospho‐Tyr1131‐IGF1R (#GTX133450), SRC (#GTX50504), phospho‐Tyr416‐SRC (#GTX24816), phospho‐Tyr527‐SRC (#GTX24816), p21 (#GTX112898), CREB (#GTX112846), phospho‐Ser133‐CREB (#GTX130379), and α‐tubulin (#GTX112141) antibodies were purchased from GeneTex (Hsinchu, Taiwan). Horseradish peroxidase (HRP)‐conjugated goat anti‐rabbit (#111‐035‐003) and anti‐mouse (#115‐035‐003) secondary antibodies were purchased from Jackson ImmunoResearch (West Grove, PA, USA).

### 2.3 Cell culture

U2OS (RRID: CVCL_0042; #60187), HepG2/C3A (RRID: CVCL_1098; #60177), MES‐SA (RRID: CVCL_1404; #60333), and MES‐SA/Dx5 (RRID: CVCL_2598; #60331) cell lines were purchased from the Bioresource Collection and Research Center (Hsinchu, Taiwan). DLD‐1 (RRID: CVCL_0248; #HD‐PAR‐111) and RKO (RRID: CVCL_0504; #HD‐PAR‐059) cell lines were purchased from Horizon Discovery (Cambridge, UK). The HCT116 (RRID: CVCL_0291; #183) cell line was purchased from the CORE Cell Center (The Johns Hopkins University School of Medicine, Baltimore, MD, USA). HeLa cells (Kyoto strain) stably expressing α‐tubulin‐enhanced green fluorescent protein (EGFP) and histone H2B‐mCherry (HeLa‐Kyoto‐tubulin‐EGFP/H2B‐mCherry; RRID: CVCL_L802; #330670) were purchased from CLS Cell Line Service (Eppelheim, Germany). HeLa (RRID: CVCL_0030) and SKOV3 (RRID: CVCL_0532) cell lines were kindly provided by Dr. Chien‐Fu Hung (The Johns Hopkins University School of Medicine, Baltimore, MD, USA). AsPC‐1 (RRID: CVCL_0152), BxPC‐3 (RRID: CVCL_0186), HPAC (RRID: CVCL_3517), and PANC‐1 (RRID: CVCL_0480) cells were kindly provided by Dr. Hsin‐Yi Chen (Taipei Medical University, Taipei, Taiwan). HT29 (RRID: CVCL_A8EZ) and p53‐knockout HCT116 (HCT116‐p53‐KO; RRID: CVCL_HD97) cells were kindly provided by Dr. Ya‐Wen Cheng (Taipei Medical University, Taipei, Taiwan). Oxaliplatin‐resistant HCT116 cells were kindly provided by Dr. Tsui‐Chin Huang (Taipei Medical University, Taipei, Taiwan). Cells were cultured in RPMI‐1640 (U2OS, HepG2/C3A, HeLa, SKOV3, AsPC‐1, BxPC‐3, HT29), DMEM (DLD‐1, RKO, HeLa‐Kyoto‐tubulin‐EGFP/H2B‐mCherry, HPAC, PANC‐1), or McCoy's 5A (MES‐SA, MES‐SA/Dx5, HCT116, HCT116‐p53‐KO) supplemented with 10% FBS, 2 mm L‐glutamine, 1 mm sodium pyruvate, 1× non‐essential amino acids, glucose (4.5 g L^−1^ for RPMI‐1640/DMEM and 3 g L^−1^ for McCoy's 5A), and 1× antibiotic‐antimycotic solution. All cell lines have undergone authentication using short tandem repeat (STR) profiling within the past 3 years and have consistently been tested to ensure that they are free of mycoplasma contamination. Experiments were performed on cells at passage numbers < 10 after received.

### 2.4 *In vitro* tubulin polymerization assay

*In vitro* tubulin polymerization was measured with a commercial kit (#BK006P; Cytoskeleton) according to the manufacturer's instructions. Briefly, purified tubulins (300 μg) were incubated with drugs in reaction buffer (80 mm PIPES pH 6.9, 2 mm MgCl_2_, 0.5 mm EGTA, 1 mm GTP, and 10.2% glycerol). Tubulin turbidity (the absorbance at 340 nm) was recorded every minute for 60 min at 37 °C on the Synergy HTX Multi‐Mode Microplate Reader (BioTek Instruments, Winooski, VT, USA). The scattering of light by microtubules is directly proportional to the concentration of microtubule polymer.

### 2.5 Microtubule assembly in cells

Cells were treated with VU‐0365114 (10 μm), colchicine (10 μm), and paclitaxel (0.5 μm) for 3 h, and then lysed in reaction buffer (80 mm PIPES pH 6.9, 2 mm MgCl_2_, 0.5 mm EGTA, 1 mm GTP, 1% NP‐40, and 15% glycerol) for 5 min at room temperature. After centrifugation at 19 000 *g* for 5 min, supernatants containing soluble free‐tubulins were collected. Pellets containing assembled tubulin were further lysed with 1× sodium dodecyl sulfate (SDS) sample buffer. Both supernatants and pellets were analyzed by SDS‐polyacrylamide gel electrophoresis (PAGE) and Western blotting as previously described [18]. Tubulins were detected using the anti‐α‐tubulin antibody.

### 2.6 Competitive tubulin‐binding assay

Purified tubulins (20 μg) were first incubated with various doses of VU‐0365114 or vinblastine in reaction buffer (80 mm PIPES pH 6.9, 2 mm MgCl_2_, and 0.5 mm EGTA) at 37 °C for 1 h. Then, colchicine (10 μm) or BODIPY FL‐vinblastine (3 μm) was added and incubated at 37 °C for an additional 30 min. The fluorescence reading of the colchicine‐tubulin complex (Ex/Em = 365/435 nm) or BODIPY FL‐vinblastine (Ex/Em = 488/512 nm) was measured on a Synergy HTX Multi‐Mode Microplate Reader (BioTek Instruments). After subtracting the background, the fluorescence intensity was normalized to the control. The reduction in fluorescence intensity indicates that the test drug can compete for the binding of tubulins with colchicine or BODIPY FL‐vinblastine.

### 2.7 Limited proteolysis assay

Purified tubulins (20 μg) were incubated with drugs (100 μm) in reaction buffer (80 mm PIPES pH 6.9, 2 mm MgCl_2_, 0.5 mm EGTA, 1 mm GTP, and 10.2% glycerol) at 30 °C for 30 min. Then, tubulins and tubulin‐drug complexes were digested on ice for 20 min with 20 μg·mL^−1^ L‐(tosylamido‐2‐phenyl) ethyl chloromethyl ketone (TPCK)‐treated trypsin (1 : 40, w/w to tubulin). TPCK was used to inhibit contaminating chymotryptic activity. The reaction mixture was mixed with 1× SDS sample buffer and boiled for 5 min, and then resolved on 7.5% and 15% SDS/PAGE. Tubulins and drug‐tubulin complexes were visualized using the One‐Step Blue Protein Gel Stain (#21003‐1L; Biotium, Hayward, CA, USA).

### 2.8 RNA sequencing and real‐time quantitative polymerase chain reaction (qPCR)

AsPC‐1 and PANC‐1 cells were treated with 10 μm VU‐0365114 for 18 h. Total RNA were isolated using the GENEzol TriRNA Pure Kit (#GZX100) that was purchased from Geneaid (New Taipei City, Taiwan). RNA sequencing was performed as previously described [18]. The differentially expressed genes are shown in Tables S1 and S2. Raw and processed data (GSE168020) were deposited in the Gene Expression Omnibus (GEO). For real‐time qPCR, total RNA were reverse‐transcribed into complementary (c)DNA using the iScript cDNA Synthesis Kit (#1708891) that was purchased from Bio‐Rad Laboratories (Hercules, CA, USA). PCR amplification on a Light‐Cycler 96 System (Roche, Indianapolis, IN, USA) was performed using the IQ2 SYBR Green Fast qPCR System Master Mix (#DBU‐006) that was purchased from Bio‐Genesis Technologies (Taipei, Taiwan). The following primer pairs were used: 5′‐GTGTTGGACCGAATTCGCAA‐3′ (forward) and 5′‐AGCTTGGACTTCTTGCCATAATCA‐3′ (reverse) for human α‐Tubulin (TUBA1A, TUBA1B, TUBA1C, and TUBAP2); 5′‐TGGACTCTGTTCGCTCAGGT‐3′ (forward) and 5′‐TGCCTCCTTCCGTACCACAT‐3′ (reverse) for human β‐Tubulin (TUBB); 5′‐TTCCTCCCAGTGCCTGAAT‐3′ (forward) and 5′‐GGTTCAGAGGCTGATTGTGAT‐3′ (reverse) for human EGFR; 5′‐AGTCCCTCTAAAGCAGCTCAAAAG‐3′ (forward) and 5′‐GCCATTTCCTAGGTCTGCCTC‐3′ (reverse) for human HMGA2; and 5′‐GTTGCTATCCAGGCTGTGCT‐3′ (forward) and 5′‐AGGGCATACCCCTCGTAGAT‐3′ (reverse) for human β‐Actin (ACTB). The fold‐changes in expression were calculated using the comparative cycle threshold (CT) method.

### 2.9 Cell viability, cell cycle, and apoptosis assays

After drug treatment, cell viability was determined by an MTT assay [18]. Both floating and adherent cells were collected for the cell‐cycle and apoptosis analyses on a Muse Cell Analyzer (Merck Millipore). The cell‐cycle distribution was determined by propidium iodide (PI) staining as previously described [19]. Apoptosis was detected using the Muse Annexin V & Dead Cell Kit (#MCH100105; Luminex, Austin, TX, USA).

### 2.10 Western blot analysis

Cells were lysed in ice‐cold RIPA buffer supplemented with 1× protease and phosphatase inhibitor cocktails at 4 °C for 30 min. Whole‐cell lysates were subjected to SDS/PAGE and Western blotting as previously described [18]. Protein bands were detected with the Western Lightning Plus enhanced chemiluminescence (ECL) detecting reagent (PerkinElmer, Waltham, MA, USA) and visualized using GE Amersham Imager 600 (GE Healthcare Life Sciences, Marlborough, MA, USA).

### 2.11 Cellular thermal shift assay (CETSA)

The CETSA was performed as previously described [20]. Briefly, cells were treated with 10 μm VU‐0365114 for 1 h and then harvested by trypsinization. Cell suspensions (10^6^ cells in 100 μL PBS) were heated for 3 min on a polymerase chain reaction (PCR) machine. After cooling down to room temperature for 3 min, cells were immediately lysed with two freeze–thaw cycles. After centrifugation, the supernatants were mixed with SDS sample buffer and subjected to Western blot analysis.

### 2.12 Tumor xenograft assay

The animal experiments were conducted according to the guidelines and regulations at the Institutional Animal Care and Use Committee (IACUC) of Taipei Medical University, Taiwan, with the approval numbers LAC‐2019‐0403 and LAC‐2021‐0373. Mice were housed in individually ventilated cages (IVC) with sterile bedding, maintaining a controlled environment at a temperature of 22 ± 3 °C, relative humidity of 50 ± 20%, and a 12/12 h light/dark cycle. Food and water were free to access throughout the entire study period. HCT116 cells (5 × 10^6^) were subcutaneously injected into the flank of 6‐week‐old female BALB/C nude mice (Trineo Biotechnology, New Taipei City, Taiwan). After the tumor volumes had reached about 150 mm^3^ on day 10, mice (*n* = 8 in each group) were intraperitoneally injected with 5 daily doses of VU‐0365114 (5 mg kg^−1^) or the vehicle solvent control (50 μL DMSO). The tumor size and body weight were measured twice per week, and the tumor volume was calculated by the formula: 0.52 × length × width^2^. At the end of the experiment, mice were sacrificed, and essential organs were removed and subjected to hematoxylin and eosin (H&E) staining to examine whether histological damage had occurred due to VU‐0365114 treatment.

### 2.13 Functional observational battery (FOB) test

The animal housing and handling conditions were as described in the above section. Six‐week‐old male BALB/C mice (Trineo Biotechnology) were intraperitoneally injected with 5 daily doses of VU‐0365114 (5 mg·kg^−1^; *n* = 3) or the vehicle solvent control (4% DMSO in PBS; *n* = 3) for 3 weeks. Neurotoxicity was assessed using the functional observational battery (FOB) [21], both before and after administering VU‐0365114 treatment. The FOB encompassed a comprehensive evaluation of the animals' physical appearance, behavior, and overall functional condition, following guidelines set by the United States Environmental Protection Agency (US‐EPA) [22]. These evaluations were conducted within the home cage and also during the animals' free movement in an open field, as well as during manipulative tests. Detailed procedures and scoring criteria for the FOB protocol in this study can be found in Table S3, which was executed based on methodologies outlined by Moser and Ross [23] and adapted for mice [24, 25]. Initially, assessments were conducted within the home cage. The observer recorded each animal's posture, activity level, and eye closure. The presence or absence of clonic or tonic movements, spontaneous vocalizations, and biting were also documented. Subsequently, the animal was gently removed from its cage, and the ease of handling and removal was rated. All instances of lacrimation, salivation, and piloerection were noted, along with any other abnormal signs. The next step involved placing the animal in an open field arena containing clean absorbent paper on the surface, allowing the animal to explore freely for a duration of 3 min. During this period, the observer assessed various aspects for each mouse, including its level of alertness, gait, activity, rearing behavior, as well as any abnormal postures, repetitive movements, stereotypical behaviors, pelvis elevation, and tail position. At the end of the 3 min, the number of fecal boluses and urine pools was counted, and the presence or absence of diarrhea was recorded. Finally, the tail suspension test [26] was conducted. In this test, mice were suspended by the tail with their heads approximately 10 cm above the ground and were recorded for 5 min. Initially, mice displayed active behaviors such as struggling, attempting to climb the tail, or escape. Over time, these behaviors might transition to periods of reduced activity or immobility. The point at which the mouse stopped struggling and remained still was noted, serving as an indicator of the animal's stress response and potential antidepressant effects [26].

### 2.14 Transfection analysis

For the small interfering (si)RNA knockdown analysis, ON‐TARGETplus SMARTpool siRNA for human CHRM5 (#L‐005466‐00‐0005) and its non‐targeting siRNA (#D‐001810‐10‐05) were purchased from Horizon Discovery, and siRNA for human MPS1 (5′‐GCACGUGACUACUUUCAAA‐3′) and its non‐targeting siRNA (5′‐UUCUCCGAACGUGUCACGU‐3′) were purchased from MDBio (Taipei, Taiwan). siRNAs were transfected into 50% confluent cells in 6‐cm dishes with Lipofectamine RNAiMAX (#13778150; Thermo Fisher Scientific) according to the manufacturer's instructions. For the gene overexpression analysis, pCMV3 (#CV011), pCMV3‐CHRM5 (#HG20062‐UT), pCMV3‐ABCB1 (#HG12030‐UT), and pCMV3‐MPS1 (#HG11529‐UT) plasmids were purchased from SinoBiological (Beijing, China). Plasmids were transfected into 80% confluent cells in 6‐cm dishes with Lipofectamine 3000 (#L3000015; Thermo Fisher Scientific) according to the manufacturer's instructions. Transfected cells were incubated at 37 °C for 1 ~ 3 days for further experiments.

### 2.15 Multidrug resistance (MDR) assay

Multidrug resistance pump activity was determined by a fluorometric MDR assay kit (#ab112142; Abcam) according to the manufacturer's instructions. Briefly, cells were spread in a 96‐well black plate with a clear bottom and incubated overnight. After reaching confluence, cells were treated with drugs (10 μm) at 37 °C for 2 h, followed by MDR dye‐loading at room temperature for 1 h. The fluorescence intensity was monitored at Ex/Em = 485/528 nm on the Synergy HTX Multi‐Mode Microplate Reader (BioTek Instruments).

### 2.16 P‐glycoprotein (P‐gp) ATPase assay

The P‐gp ATPase activity was determined with the Pgp‐Glo Assay Systems (#V3601; Promega, Madison, WI, USA) according to the manufacturer's instruction. P‐gp membranes were first mixed with Na_3_VO_4_ (0.25 mm), verapamil (0.5 mm), or VU‐0365114 (10 μm) in Pgp‐Glo assay buffer. After incubation at 37 °C for 5 min, MgATP (5 mm) was added to initiate the reaction (at 37 °C for 40 min). Luminescence was developed by adding an ATP detection reagent (at room temperature for 20 min) and then detected on the Synergy HTX Multi‐Mode Microplate Reader (BioTek Instruments). Differences in sample luminescence (ΔRLU) compared to the Na_3_VO_4_‐treated sample were used to calculate basal P‐gp ATPase activity and the effect of a test compound on P‐gp ATPase activity.

### 2.17 Statistical analysis

Data from the competitive tubulin‐binding, qPCR, cell viability, tumor xenograft, MDR, and P‐gp ATPase assays were plotted and analyzed with a two‐tailed paired Student's *t*‐test or one‐way analysis of variance (ANOVA) for multiple comparison using the graphpad prism 9 (GraphPad Software, San Diego, CA, USA). Results are shown as the mean ± standard error of the mean (SEM) or mean ± standard deviation (SD). The 50% inhibitory concentration (IC_50_) values for the cell viability assay were calculated using the online Very Simple IC50 Tool Kit (http://www.ic50.tk/). For the correlation between gene expressions and VU‐0365114 drug activity in NCI‐60 cell lines, data were calculated by the online Pearson Correlation Coefficient Calculator (https://www.socscistatistics.com/tests/pearson/default2.aspx). Absolute values of Pearson's correlation coefficient (*r*) of 0 ~ 0.19, 0.2 ~ 0.39, 0.4 ~ 0.59, 0.6 ~ 0.79, and 0.8 ~ 1 were respectively considered very weak, weak, moderate, strong, and very strong. For data generated from other bioinformatics resources, statistical analysis was performed by built‐in programs in each database. For all tests, *P* < 0.05 was considered statistically significant.

## 3 Results

### 3.1 Prediction of potential microtubule‐targeting agents by the CMap analysis

**Fig. 1:**
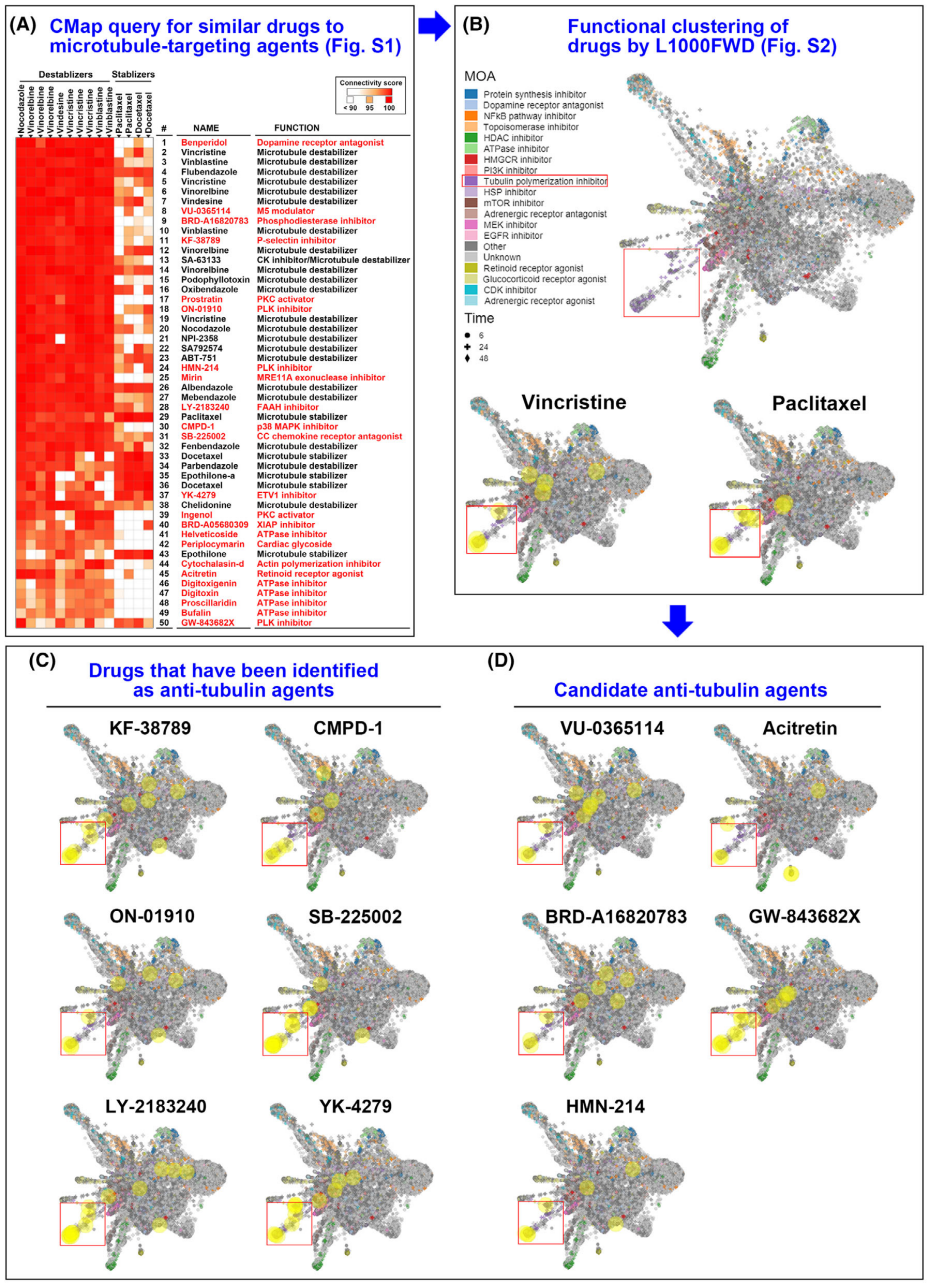
Prediction of novel microtubule‐targeting agents by the CMap analysis. (A) Connections of drug‐gene signatures were analyzed using the “Touchstone” tool in the CMap database (https://clue.io/). Connections were viewed as a heat map ranked by the summary connectivity score. Drugs highlighted in red were potential microtubule‐targeting agents. Some drug names were duplicated because they have multiple gene signatures in the CMap database. An enlarged image was provided in Fig. S1. (B) L1000FWD visualization of drug‐gene signatures. Drugs sharing similar mechanisms of action (MOAs) were clustered together. The red box indicates the cluster of tubulin polymerization inhibitors. The lower panel shows the representative L1000FWD figures of microtubule‐targeting agents (vinblastine and paclitaxel). An enlarged image is provided in Fig. S2. (C) L1000FWD visualization of candidate drugs that were identified as microtubule‐targeting agents in other studies. (D) L1000FWD visualization of candidate drugs that had not been previously identified as microtubule‐targeting agents. In panels B–D, each clustered point represents the gene signature of a drug in different cell lines, treated with varying doses and time intervals. The yellow circles highlight the queried drugs' gene signature.

To overcome the major limitations of the classical microtubule‐targeting agents, we employed CMap to predict new microtubule‐targeting agents. The CMap database consists of gene expression signatures from small‐molecule‐treated human cancer cell lines, which can be used to compare queried and existing gene signatures, and then the connections among small molecules sharing similar MOAs can be found [10, 27]. We found several candidate drugs (Fig. 1A and Fig. S1, highlighted in red) sharing similar gene signatures to well‐established microtubule stabilizers and/or destabilizers. To narrow down the number of candidates, these drugs were further analyzed by L1000FWD [12] according to a strategy proposed in our previous study [28]. The L1000FWD map can be used to identify the MOAs of drugs through unsupervised clustering [12]. In Fig. 1B (upper panel) and Fig. S2, tubulin polymerization inhibitors (purple dots) were clustered in a specific region, highlighted in a red box. By examining the gene signatures of vincristine and paclitaxel (the yellow circles), it was found that they were mostly located within this red box region (Fig. 1B, lower panel). Similarly, the gene signatures of 11 candidate drugs (the yellow circles) also mostly located within the red box region (Fig. 1C,D), indicating that they are potential microtubule‐targeting agents. Indeed, six drugs (KF‐38789, ON‐01910, LY‐2183240, CMPD‐1, SB‐225002, and YK‐4279; Fig. 1C) were previously demonstrated to exhibit microtubule‐targeting effects [29, 30, 31, 32, 33, 34], which supports the feasibility of our analytic strategy. The five remaining drugs (VU‐0365114, BRD‐A16820783, HMN‐214, acitretin, and GW‐843682X; Fig. 1D), which did not have any reported microtubule‐targeting activity, were subjected to further investigation. Other 12 drugs with distinct L1000FWD mapping patterns were excluded (Fig. S3).

### 3.2 VU‐0365114 inhibits tubulin polymerization

**Fig. 2:**
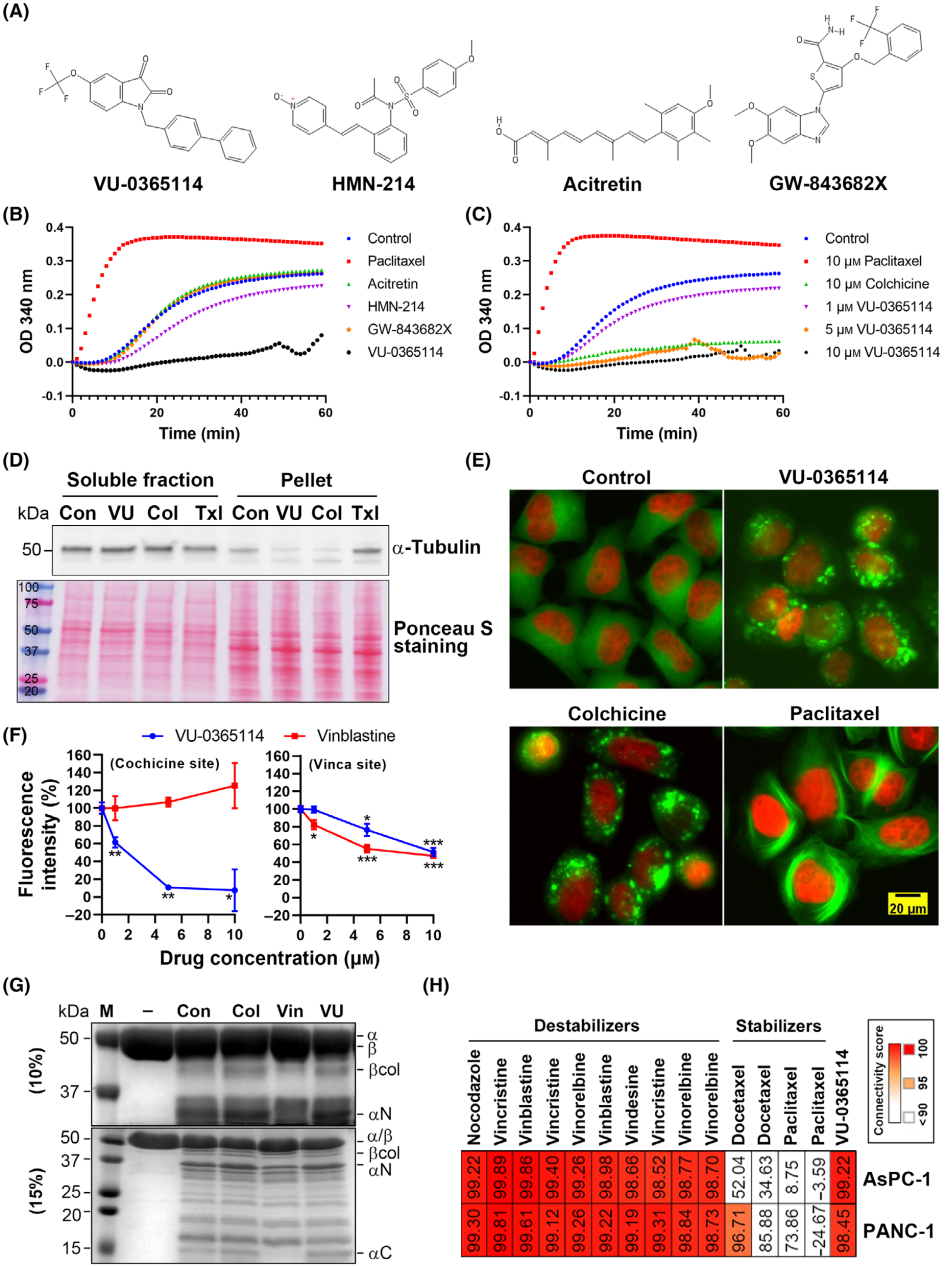
Identification of VU‐0365114 as a potent microtubule‐targeting agent. (A) The chemical structures of VU‐0365114, HMN‐214, acitretin, and GW‐843682X. (B, C) Purified tubulins were incubated with 10 μm of the drugs (B) or the indicated concentration of the drugs (C) for 60 min. Tubulin polymerization was detected by measuring the absorbance at 340 nm. Data represent three independent experiments. (D) HeLa cells were treated with VU‐0365114 (VU; 10 μm), colchicine (Col; 10 μm), or paclitaxel (Txl; 0.5 μm) for 3 h. Soluble and assembled (pellet) tubulins were isolated and analyzed by Western blotting. The corresponding nitrocellulose membrane stained with Ponceau S was used as a loading control. Data represent three independent experiments. (E) HeLa‐Kyoto‐tubulin‐EGFP/H2B‐mCherry cells were treated with VU‐0365114 (10 μm), colchicine (10 μm), or paclitaxel (0.5 μm) for 4 h. The EGFP and mCherry fluorescence was observed under a fluorescence microscope (scale bar: 20 μm). Data represent three independent experiments. (F) The binding site of VU‐0365114 on tubulin was examined by the competition of tubulin‐binding with colchicine or BODIPY FL‐vinblastine. The intrinsic fluorescence of the colchicine‐tubulin complex or the fluorescence of BODIPY FL‐vinblastine was analyzed on a plate reader. The error bars are the mean ± SD (*n* = 3). Statistical significance, compared to untreated controls (*P < 0.05, **P < 0.01, and ***P < 0.001), was determined using a one‐way ANOVA with Tukey's *post hoc* test. (G) Tubulins were treated with DMSO (Con), colchicine (Col; 100 μm), vinblastine (Vin; 100 μm), or VU‐0365114 (VU; 100 μm), and then digested by TPCK‐treated trypsin. Samples were analyzed by SDS/PAGE. M, protein molecular weight marker; −, tubulin without digestion. Data represent three independent experiments. (H) AsPC‐1 and PANC‐1 cells were treated with 10 μm VU‐0365114 for 18 h, and then RNA sequencing analysis was performed. The differentially expressed genes were used to query the CMap database. The gene expression‐based similarity of VU‐0365114 to CMap drugs was visualized as a heat map. The original image was provided in Fig. S6. Some drug names were duplicated because they have multiple gene signatures in the CMap database. Data were obtained from one experiment conducted on two technical replicates.

Because BRD‐A16820783 is not commercially available, we only examined the effects of VU‐0365114, HMN‐214, acitretin, and GW‐843682X (Fig. 2A). In a cell‐free system, paclitaxel promoted and stabilized tubulin polymerization. In contrast, VU‐0365114 significantly inhibited tubulin polymerization. No significant effects were observed in HMN‐214‐, acitretin‐, or GW‐843682X‐treated samples (Fig. 2B). A dose‐dependent experiment indicated that 5 and 10 μm VU‐0365114 completely inhibited tubulin polymerization, which was comparable to colchicine, the positive control, at 10 μm (Fig. 2C). Because VU‐0365114 was originally synthesized as a positive allosteric modulator of M5 mAChR [35], its effect on tubulin polymerization was compared with other positive allosteric modulators of M5 mAChR (ML‐380 [36] and VU‐0238429 [37]). When compared their chemical structures with VU‐0365114, VU‐0238429 was more similar to VU‐0365114 than ML‐380 (Fig. S4A). Like VU‐0365114, VU‐0238429, but not ML‐380, completely inhibited tubulin polymerization (Fig. S4B), suggesting that the unique chemical structures of VU‐0365114 and VU‐0238429 were necessary to inhibit tubulin polymerization. To investigate whether VU‐0365114 disturbed the assembly of microtubules in cells, levels of soluble (unpolymerized) and polymerized (pellet) tubulins in HeLa cells were analyzed. As expected, VU‐0365114 and colchicine reduced the polymerized fractions, whereas paclitaxel had the opposite effect (Fig. 2D). Furthermore, HeLa cells stably expressing α‐tubulin‐enhanced green fluorescent protein (EGFP) and histone H2B‐mCherry were treated with VU‐0365114, colchicine, and paclitaxel to observe their effects on microtubule organization. In support of this, microtubule fragments appeared scattered in the cytoplasm of VU‐0365114‐ and colchicine‐treated cells compared to untreated cells. In contrast, integrated microtubule fibers increased in paclitaxel‐treated cells (Fig. 2E). Therefore, VU‐0365114 acted as a microtubule destabilizer.

Vinca and colchicine sites are major binding sites for microtubule‐destabilizing agents [5, 6, 7]. To investigate whether VU‐0365114 interacted with tubulin through these two sites, colchicine and BODIPY FL‐vinblastine competition assays were performed. As shown in Fig. 2F (left panel), VU‐0365114, but not vinblastine (as a negative control) competed for tubulin‐binding with colchicine, which displayed its intrinsic fluorescence [38]. Interestingly, both VU‐0365114 and unlabeled vinblastine, but not nocodazole (as a negative control), competed with BODIPY FL‐vinblastine to bind to tubulins (Fig. 2F, Fig. S5). These results indicated that VU‐0365114 may bind to both colchicine and vinca sites. Because the binding of drugs to tubulin dimers causes protein conformational changes, resulting in different proteolysis patterns by trypsin [39], a limited proteolysis assay was performed. Like colchicine, pre‐incubation of tubulins with VU‐0365114 produced an increase in βcol fragments. In contrast, vinblastine decreased the intensity of the βcol, αN, and αC fragments (Fig. 2G). These results indicated that the colchicine site is the major binding site of VU‐0365114 on tubulins.

Because we predicted VU‐0365114 to be a microtubule‐targeting agent according to the internal gene signatures in the CMap database, we wondered if the external gene signature of VU‐0365114 could, *vice versa*, be predicted as a microtubule‐targeting agent. The core cell lines used to establish CMap data included human malignant melanoma (A375), human non‐small cell lung carcinoma (A549), human non‐small cell lung adenocarcinoma (HCC515), human hepatocellular carcinoma (HepG2), human breast adenocarcinoma (MCF7), human prostate adenocarcinoma (PC3), human metastatic prostate cancer (VCAP), and human colorectal adenocarcinoma (HT29) cell lines [11]. Thus, gene expression profiles of VU‐0365114 were prepared by RNA sequencing from two unrelated pancreatic cancer cell lines (AsPC‐1 and PANC‐1). In support of this, VU‐0365114's gene signatures were highly similar to those of microtubule‐destabilizing agents and VU‐0365114 itself, but not to microtubule‐stabilizing agents (Fig. 2H, Fig. S6). It should be noted that colchicine was not selected due to its absence from the CMap database.

### 3.3 VU‐0365114 destabilizes tubulin mRNA but not protein

**Fig. 3:**
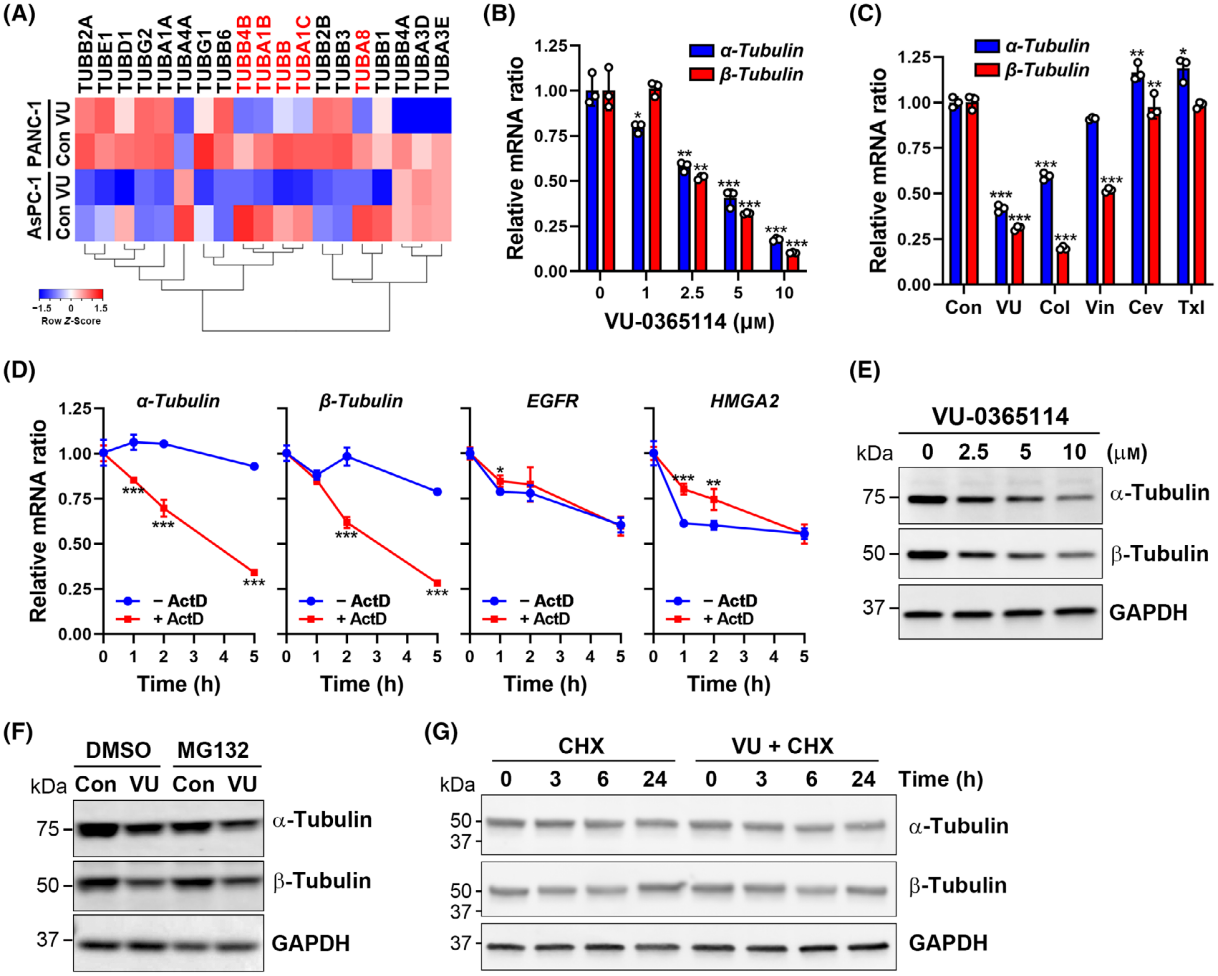
Effects of VU‐0365114 on tubulin mRNA and protein stability. (A) AsPC‐1 and PANC‐1 cells were treated with 10 μm VU‐0365114 for 18 h, and then RNA sequencing analysis was performed. The tubulin family gene expressions were visualized as a heat map. Genes highlighted in red were inhibited by VU‐0365114 in both AsPC‐1 and PANC‐1 cells. Con, untreated control cells; VU, VU‐0365114‐treated cells. Data were obtained from one experiment conducted on two technical replicates. (B) HeLa cells were treated with VU‐0365114 for 18 h, and gene expressions were examined by real‐time qPCR. The error bars are the mean ± SD (*n* = 3). Statistical significance, compared to untreated controls (*P < 0.05, **P < 0.01, and ***P < 0.001), was determined using a one‐way ANOVA with Tukey's *post hoc* test. (C) HeLa cells were treated with 2.5 μm VU‐0365114 (VU), 2.5 μm colchicine (Col), 2.5 μm vinblastine (Vin), 2.5 μm cevipabulin (Cev), or 0.1 μm paclitaxel (txl) for 18 h, and gene expressions were examined by real‐time qPCR. The error bars are the mean ± SD (*n* = 3). Statistical significance, compared to untreated controls (*P < 0.05, **P < 0.01, and ***P < 0.001), was determined using a one‐way ANOVA with Tukey's *post hoc* test. (D) HeLa cells were pretreated with 10 μm VU‐0365114 for 1 h and then exposed to 10 μg mL^−1^ actinomycin D (ActD) for the indicated time intervals. The gene expressions were examined by real‐time qPCR. The error bars are the mean ± SD (*n* = 3). Statistical significance, compared to untreated controls in each time point (*P < 0.05, **P < 0.01, and ***P < 0.001), was determined using a two‐tailed paired Student's *t*‐test. (E) HeLa cells were treated with VU‐0365114 for 18 h, and protein expressions were examined by Western blotting. Data represent three independent experiments. (F) HeLa cells were treated with 10 μm VU‐0365114 with or without 10 μm MG132 for 18 h, and protein expressions were examined by Western blotting. Con, untreated control cells; VU, VU‐0365114‐treated cells. Data represent three independent experiments. (G) HeLa cells were pretreated with 10 μm VU‐0365114 (VU) for 1 h and then exposed to 3 μg·mL^−1^ cycloheximide (CHX) for the indicated time intervals. The protein expressions were examined by Western blotting. Data represent three independent experiments.

Tubulin autoregulation is a feedback control mechanism in which the synthesis of tubulin is activated by microtubule stabilization and suppressed by microtubule destabilization through the degradation of tubulin mRNA [40]. RNA sequencing analysis in AsPC‐1 and PANC‐1 cells indicated that VU‐0365114 inhibited many tubulin family gene expressions, especially *TUBA1A*, *TUBA1C*, *TUBA8*, *TUBB*, and *TUBB4B* (Fig. 3A). Interestingly, downregulation of *TUBB4A*, *TUBA3D*, and *TUBA3E* genes was only found in PANC‐1 cells, suggesting that a cell‐type specific effect of VU‐0365114 on tubulin mRNA expression exists. A dose‐dependent inhibition of α/β‐tubulin mRNA expressions was confirmed in HeLa cells (Fig. 3B). Consistent with tubulin autoregulation [40], VU‐0365114 and microtubule‐destabilizing agents (colchicine and vinblastine) inhibited α/β‐tubulin mRNA expressions, whereas microtubule‐stabilizing agents (cevipabulin and paclitaxel) slightly increased α‐tubulin mRNA expression (Fig. 3C). To measure the effect of VU‐0365114 on α/β‐tubulin mRNA stabilities, HeLa cells were treated with VU‐0365114 with or without actinomycin D. Actinomycin D was used to inhibit transcription by binding to DNA and preventing RNA polymerase from elongating the nascent RNA chain. This results in the degradation of existing mRNA transcripts over time, enabling the measurement of mRNA half‐life. Indeed, VU‐0365114 significantly reduced α/β‐tubulin mRNA stabilities in the presence of actinomycin D (Fig. 3D). The mRNA stabilities of two microtubule‐unrelated genes (*EGFR* and *HMGA2*) were not reduced by VU‐0365114 (Fig. 3D), which excludes the possibility that VU‐0365114 may have a general transcription inhibition effect. The inhibition of α/β‐tubulin mRNA stabilities and expressions correlated with their protein downregulations (Fig. 3E). To investigate whether VU‐0365114 also promoted tubulin protein degradation, a proteasome inhibitor, MG132, was used. However, MG132 did not prevent VU‐0365114‐induced α/β‐tubulin protein downregulations (Fig. 3F). In addition, the stabilities of α/β‐tubulin proteins were examined by treating cells with cycloheximide to inhibit *de novo* protein synthesis. Consistently, VU‐0365114 did not alter the protein stabilities of α/β‐tubulins (Fig. 3G). Therefore, VU‐0365114 destabilizes tubulin mRNA but not protein.

### 3.4 VU‐0365114 exhibits broad‐spectrum anticancer activity, especially in colorectal cancer

**Fig. 4:**
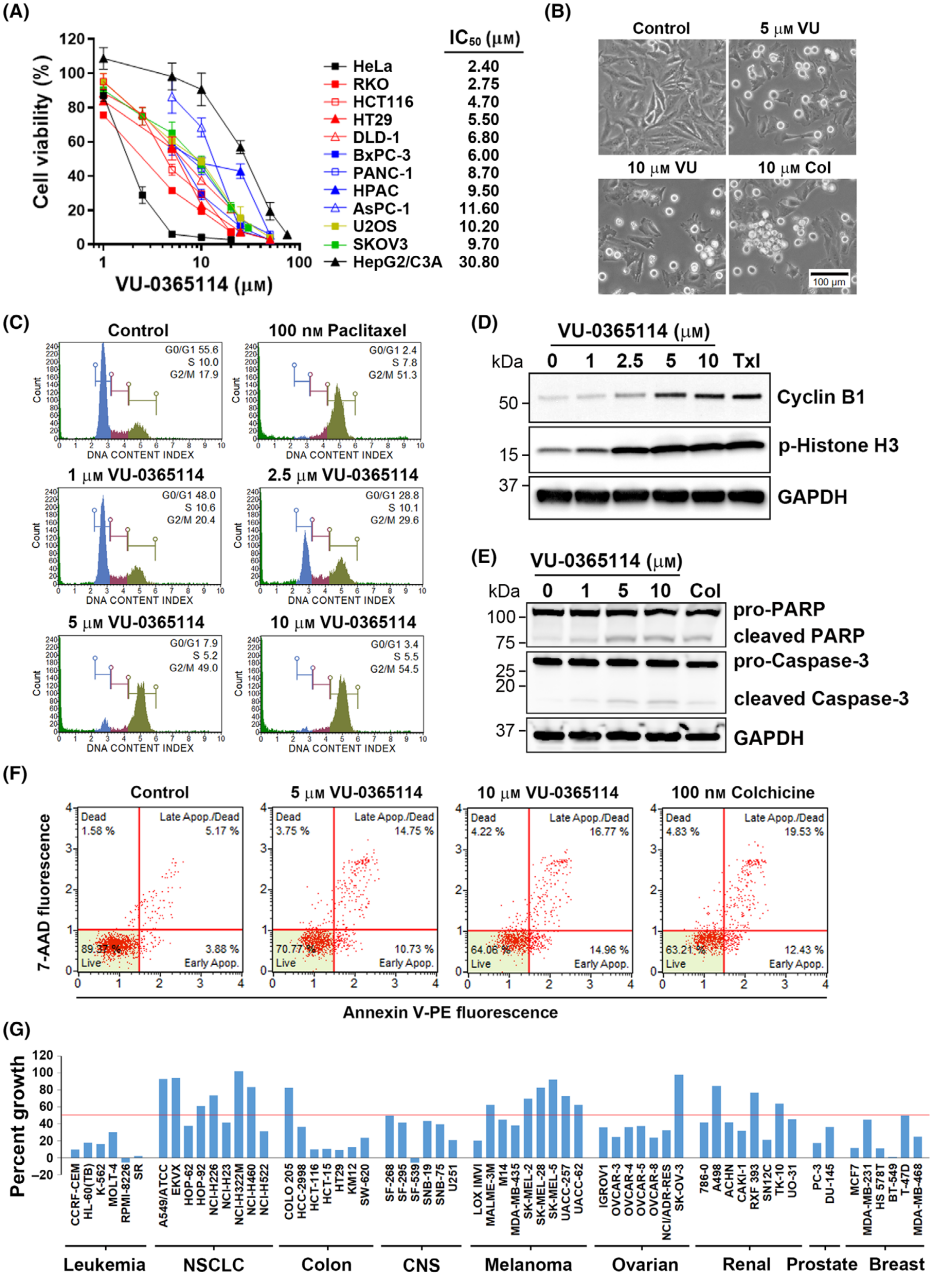
Characterization of the *in vitro* anticancer activity of VU‐0365114. (A) Cancer cells were treated with VU‐0365114 for 72 h, and then the cell viability was examined by an MTT assay. The error bars are the mean ± SD (*n* = 5). (B) HeLa cells were treated with VU‐0365114 (VU) or colchicine (Col) for 24 h, and then changes in the cell morphology were observed under bright‐field microscopy (scale bar: 100 μm). Data represent three independent experiments. (C) HeLa cells were treated with VU‐0365114 or paclitaxel for 24 h, and then the cell‐cycle status was analyzed by flow cytometry. Data represent three independent experiments. (D) HeLa cells were treated with VU‐0365114 or paclitaxel (Txl) for 24 h, and protein expressions were examined by Western blotting. Data represent three independent experiments. (E) HeLa cells were treated with VU‐0365114 or colchicine (Col) for 24 h, and protein expressions were examined by Western blotting. Data represent three independent experiments. (F) HeLa cells were treated with VU‐0365114 or colchicine for 24 h, and apoptosis was analyzed by Annexin V‐PE/7‐AAD double staining. Data represent three independent experiments. (G) VU‐0365114 was submitted to the NCI‐60 cell screen at a single dose of 10 μm for 24 h. The percentage growth inhibition is shown. Data were obtained from one experiment conducted on two technical replicates. CNS, central nervous system; NSCLC, non‐small‐cell lung cancer.

VU‐0365114 was originally synthesized as a positive allosteric modulator of human M5 mAChR [35], and its anticancer activity had never been reported. To study whether VU‐0365114 exhibited anticancer activity, various types of cancer cell lines were used, including human cervical adenocarcinoma (HeLa), human colorectal cancer (RKO, HCT116, HT29, and DLD‐1), human pancreatic cancer (BxPC‐3, PANC‐1, HPAC, and AsPC‐1), human osteosarcoma (U2OS), ovarian adenocarcinoma (SKOV3), and human hepatocellular carcinoma (HepG2/C3A) cell lines. As shown in Fig. 4A, VU‐0365114 exhibited potent anticancer activity (50% of inhibitory concentration (IC_50_) values of around 2.4 ~ 11.6 μm) against these cancer cell lines except for HepG2/C3A cells (IC_50_ = 30.8 μm). Like colchicine or paclitaxel, VU‐0365114 induced mitotic cell rounding (Fig. 4B) and G2/M cell‐cycle arrest (Fig. 4C) in HeLa cells. In addition, VU‐0365114 induced markers of mitotic arrest, including the accumulation of cyclin B1 [41] and phosphorylation of histone H3 [42] (Fig. 4D). Consistently, both VU‐0365114 and colchicine increased the mitotic index of HeLa cells (Fig. S7). Furthermore, VU‐0365114 induced apoptosis as indicated by the cleavages of poly(ADP ribose) polymerase (PARP) and caspase‐3 (Fig. 4E) and the phosphatidylserine externalization detected by an Annexin V‐PE/7‐AAD double‐staining assay (Fig. 4F). Although treatment with 10 μm VU‐0365114 for 72 h almost completely inhibited the cell viability in Hela cells (Fig. 4A), treatment with the same concentration of VU‐0365114 for 24 h only induced about 36% of cell death (Fig. 4F). Such discrepancy may be resulted from the difference in time (72‐h vs. 24‐h exposure) and indicates that a longer exposure of VU‐0365114 is needed to completely kill the cancer cells. Therefore, VU‐0365114 is a potent anticancer agent via inducing mitotic cell‐cycle arrest and apoptosis.

**Fig. 5:**
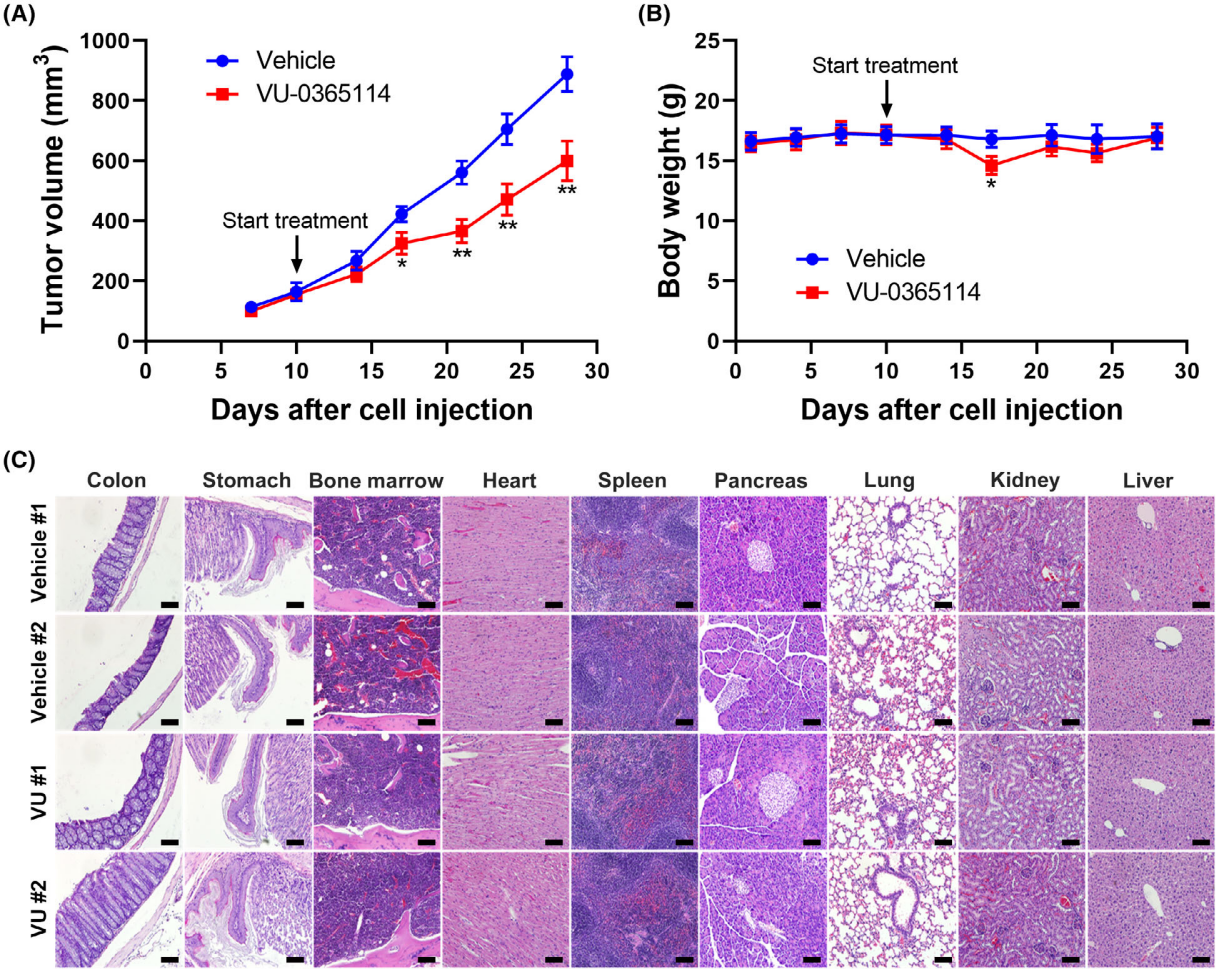
Characterization of the *in vivo* anticancer activity of VU‐0365114. HCT116 xenograft mice received VU‐0365114 (5 mg·kg^−1^; *n* = 8) or the vehicle solvent control (*n* = 8). Tumor volumes (A) and mice body weights (B) were measured twice per week. At the end of the experiment, histological examination (200× magnification; scale bar: 100 μm) of essential organs was performed in two randomly selected mice of each group (C). The error bars are the mean ± SEM (*n* = 8). Statistical significance, compared to the vehicle control group at each time point (*P < 0.05 and **P < 0.01), was determined using a two‐tailed paired Student's *t*‐test.

To more comprehensively characterize the anticancer activity of VU‐0365114, we submitted this compound to the Developmental Therapeutics Program of the National Cancer Institute (NCI) at the National Institutes of Health for screening against the NCI‐60 panel of human cancer cell lines [43] at a single dose of 10 μm. Results showed that VU‐0365114 inhibited 43 of 60 of the NCI‐60 cell lines by > 50% at 10 μm. In particular, cell lines from leukemia, colon, central nervous system (CNS), breast, ovarian, and prostate cancers were more sensitive to VU‐0365114 (Fig. 4G). Although our results suggested that VU‐0365114 exhibited the best anticancer activity in HeLa cells, its effect on other cervical cancer cell lines was unclear. Because the potency of VU‐0365114 against colorectal cancer cells was validated in our and NCI‐60 cell tests (Fig. 4A,G), we further examined the *in vivo* anticancer activity of VU‐0365114 in colorectal cancer using an HCT116 tumor xenograft assay. As shown in Fig. 5A, VU‐0365114 slowed the growth of HCT116 xenograft in mice. VU‐0365114 treatment for 1 week initially induced body weight loss, which progressively recovered afterward (Fig. 5B). The appearance and physical activity were not significantly impacted by VU‐0365114 treatment. In addition, VU‐0365114 did not cause histopathological lesions in essential organs (Fig. 5C). Although M5 mAChR, the reported target of VU‐0365114, is expressed in the brain in low abundance [41], it is still possible that side effects from VU‐0365114 treatment may manifest in brain function. To assess any potential neurological effects of VU‐0365114 in mice, a functional observation battery (FOB) test was performed before and after administering VU‐0365114 treatment. The FOB test involves observing and recording various behaviors and physical conditions to assess overall health, neurological function, and treatment effects [21]. As shown in Table S4, VU‐0365114 did not alter the FOB parameters, suggesting the absence of neurological toxicity. Therefore, VU‐0365114 exhibited broad‐spectrum anticancer activity, especially against colorectal cancer, and it was well‐tolerated and caused no severe adverse effects.

### 3.5 The anticancer activity of VU‐0365114 is not affected by muscarinic acetylcholine receptor M5 (M5 mAChR) expression

**Fig. 6:**
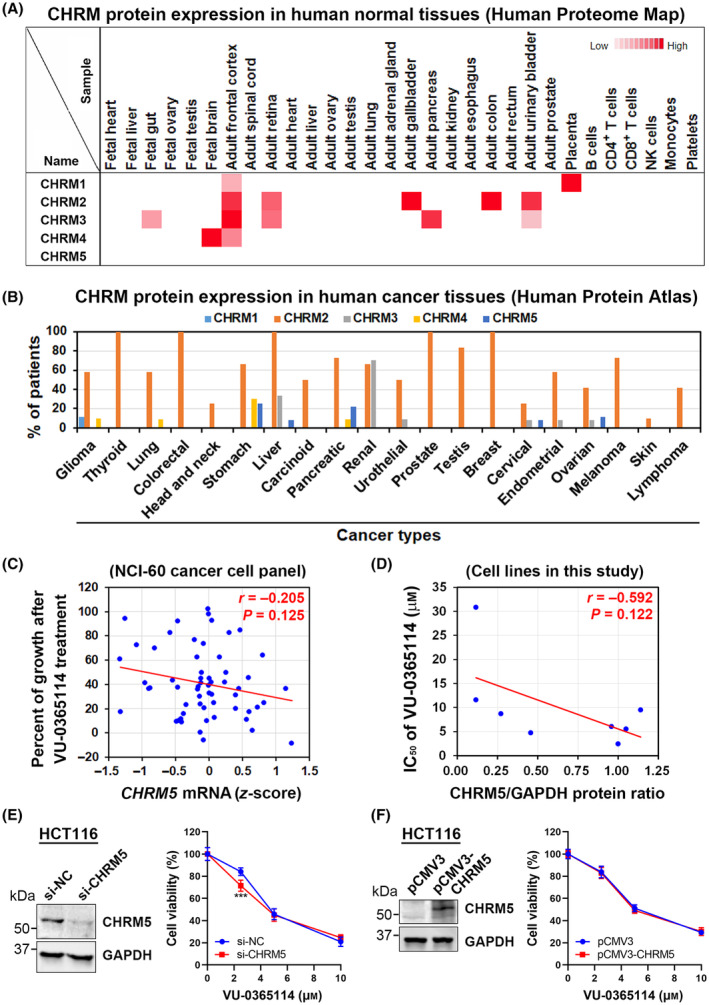
The role of M5 muscarinic acetylcholine receptor (mAChR) in the *in vitro* anticancer activity of VU‐0365114. (A) Protein expression levels of mAChRs (CHRM1 ~ CHRM5) in various human normal cell types were obtained from the Human Proteome Map. (B) Protein expression levels of mAChRs (CHRM1 ~ 5) in various human cancer types were obtained from the Human Protein Atlas. (C) The correlation of *CHRM5* mRNA and growth inhibition by VU‐0365114 in NCI‐60 cancer cell panel. *CHRM5* mRNA levels in NCI‐60 were obtained from the CellMinerCDB database. Statistical significance was determined using the Pearson's correlation coefficient (*r*) and the corresponding *P* value. (D) Protein lysates from HeLa, HCT116, HT29, BxPC‐3, PANC‐1, HPAC, AsPC‐1, and HepG2/C3A cell lines were subjected to Western blotting to assess basal CHRM5 protein expression (Fig. S6A). The correlation between the IC_50_ values of VU‐0365114 and CHRM5 protein expressions (normalized to HeLa) in these cell lines was calculated using Pearson's correlation coefficient (*r*). Data represent two independent experiments. (E, F) HCT116 cells were transfected with *CHRM5* siRNA in E or plasmid in F for 48 h, and then exposed to VU‐0365114 for 72 h. Knockdown or overexpression of M5 mAChR in E or F was confirmed by Western blotting (left panel). Cell viability was examined by an MTT assay (right panel). The error bars are the mean ± SD (*n* = 5). Statistical significance, compared to the transfection control group at each dose (***P < 0.001), was determined using a two‐tailed paired Student's *t*‐test. si‐NC, negative control siRNA.

Accumulating evidence suggested that mAChR‐dependent signaling can promote cancer cell proliferation and progression, and therefore targeting specific mAChR subtypes represents a novel anticancer strategy [44, 45, 46]. However, the role of M5 mAChR in cancer has rarely been characterized. By mining the Human Proteome Map and Human Protein Atlas [14, 15], we found that M5 mAChR was not detected in most normal and cancer tissues compared to other mAChRs (Fig. 6A,B). Only certain types of cancers (stomach, pancreas, ovarian, liver, and cervix) expressed the M5 mAChR protein (Fig. 6B). No significant association between *CHRM5* mRNA levels and VU‐0365114 drug activity in NCI‐60 cell lines was found (Fig. 6C). Similarly, the IC_50_ values were not significantly correlated with the expressions of M5 mAChR protein among the tested cancer cell lines (Fig. 6D, Fig. S8). Despite the absence of statistical significance, cancer cell lines with higher M5 mAChR expressions exhibited a tendency to be more sensitive to VU‐0365114 treatment (Fig. 6C,D). To validate the role of M5 mAChR in the anticancer activity of VU‐0365114 against colorectal cancer cells, the expressions of M5 mAChR in HCT116 cells were individually manipulated using siRNA and plasmid transfections. However, both knockdown and overexpression of M5 mAChR did not alter the cytotoxicity of VU‐0365114 (Fig. 6E,F). Similar phenomena were also observed in HeLa and PANC‐1 cells transfected with CHRM5‐siRNA and ‐plasmid (Fig. S9). Therefore, we concluded that the anticancer effect of VU‐0365114 was not affected by the existence of its original target, M5 mAChR.

### 3.6 VU‐0365114 overcomes multidrug resistance (MDR)

**Fig. 7:**
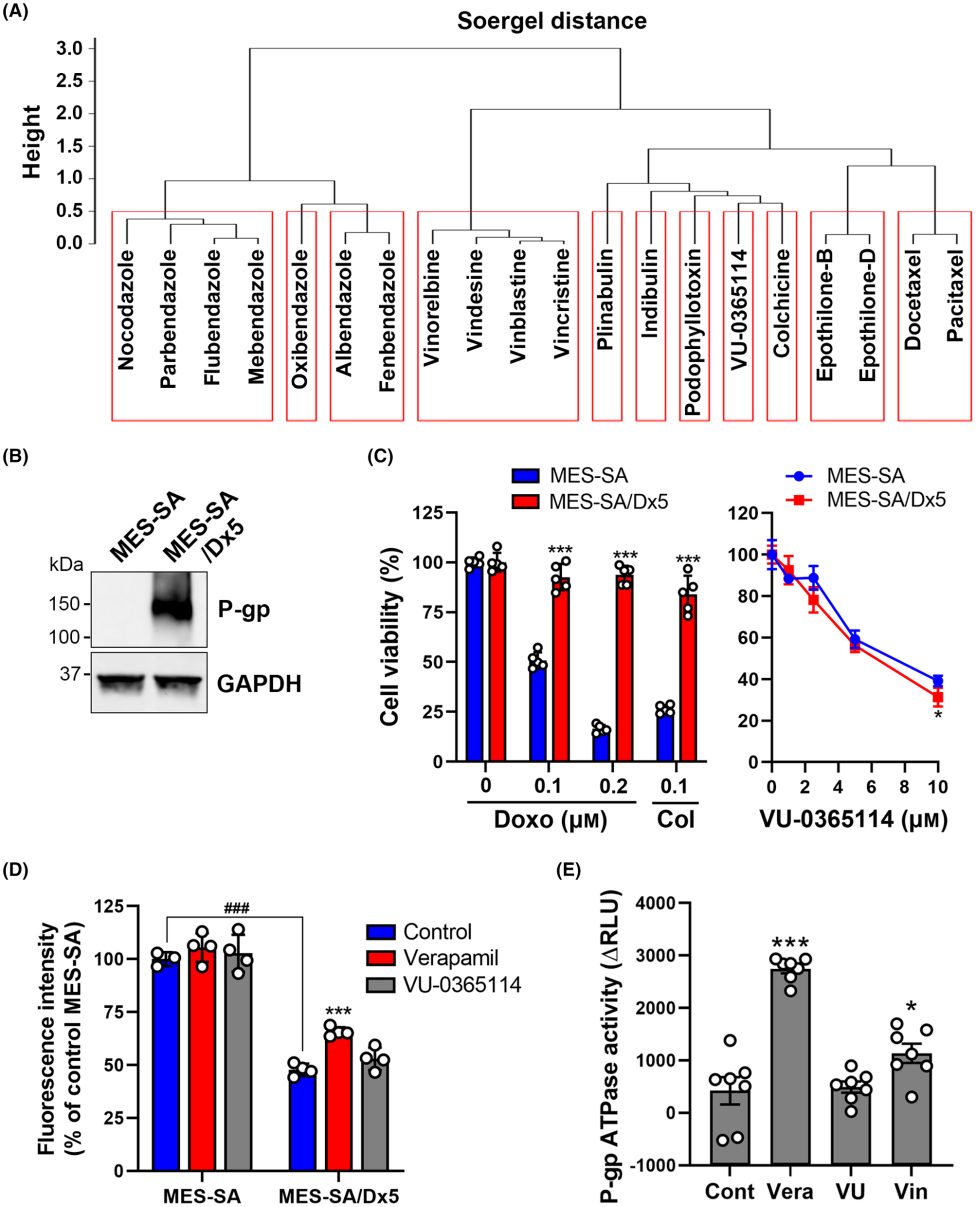
The effect of VU‐0365114 on overcoming multidrug resistance (MDR). (A) ChemBioServer hierarchical clustering of compounds based on structural similarities. The parameters selected were as follows: distance = Soergel (Tanimoto coefficient); clustering linkage = Ward; and clustering threshold = 0.5. Similar drugs were included within the same red box. (B) The P‐glycoprotein (P‐gp) expression in MES‐SA and MES‐SA/Dx5 cells were examined by Western blotting. Data represent three independent experiments. (C) MES‐SA and MES‐SA/Dx5 cells were treated with various concentrations of doxorubicin (Doxo), colchicine (Col), or VU‐0365114 for 72 h. Then, cell viability was examined by an MTT assay. The error bars are the mean ± SD (*n* = 5). Statistical significance, compared to parental MES‐SA cells at each dose (*P < 0.05 and ***P < 0.001), was determined using a two‐tailed paired Student's *t*‐test. (D) MES‐SA and MES‐SA/Dx5 cells were treated with verapamil (10 μm) or VU‐0365114 (10 μm) for 2 h. Then, an MDR assay was performed. The error bars are the mean ± SD (*n* = 4). Statistical significance, compared to untreated controls (***P < 0.001), was determined using a one‐way ANOVA with Tukey's *post hoc* test. Statistical significance, compared between MES‐SA/Dx5 and MES‐SA cells (^###^ *P* < 0.001), was determined using a two‐tailed paired Student's *t*‐test. (E) A P‐glycoprotein (P‐gp) ATPase activity assay was performed to examine the effect of verapamil (Vera; 0.5 mm), VU‐0365114 (VU; 10 μm), or vinblastine (Vin; 10 μm). The error bars are the mean ± SEM (*n* = 7). Statistical significance, compared to untreated controls (*P < 0.05 and ***P < 0.001), was determined using a one‐way ANOVA with Tukey's *post hoc* test.

The increase of drug efflux is one of the major mechanisms for multi‐drug resistance (MDR). The main category of drug efflux pumps is ATP‐binding cassette (ABC) drug transporters, such as P‐gp, multidrug‐resistance‐associated proteins (MRPs), and breast cancer‐resistance protein (BCRP, also known as ABCG2) [47]. Drugs with a bulky or planar structure, such as anthracyclines (e.g., doxorubicin), taxanes (e.g., paclitaxel), and vinca alkaloids (e.g., vincristine), have a high affinity for P‐gp and are more likely to be recognized by the pump, leading to MDR [48]. When comparing the chemical structure of VU‐0365114 with classical microtubule‐targeting agents by hierarchical clustering using ChemBioServer [13], we found that VU‐0365114 was dissimilar (Soergel distance > 0.5) to existing microtubule‐targeting agents (Fig. 7A). Interestingly, colchicine clustered with VU‐0365114, suggesting that colchicine is the most structurally similar to VU‐0365114 among these tubulin inhibitors (Fig. 7A). This supports our finding that the major tubulin‐binding site of VU‐0365114 was colchicine site (Fig. 2F,G). According to the above analysis, we hypothesized that VU‐0365114 may escape the MDR's mechanism. To demonstrate this hypothesis, the human uterine sarcoma MES‐SA and its MDR variant MES‐SA/Dx5 cell lines were used. MES‐SA/Dx5 cells were established by continuous exposure to increasing concentrations of doxorubicin. MES‐SA/Dx5 cells express high levels of multidrug‐resistance protein 1 (MDR1; also known as P‐glycoprotein/P‐gp) and are cross‐resistant to various chemotherapeutic agents [49, 50]. We also showed that MES‐SA/Dx5 cells had high expression of P‐gp (Fig. 7B) and were resistant to doxorubicin and colchicine (Fig. 7C, left panel). In contrast, similar cytotoxicity was found in MES‐SA and MES‐SA/Dx5 cells in response to VU‐0365114 (Fig. 7C, right panel), suggesting that VU‐0365114 could overcome the MDR.

To investigate whether VU‐0365114 alters MDR pump activity, we used MDR indicator, a hydrophobic fluorescent dye molecule that rapidly penetrates cell membranes and becomes trapped in cells. This dye is excluded by MDR transporters in MDR‐overexpressing cells. As expected, the intracellular fluorescence intensity was reduced in MES‐SA/Dx5 compared with MES‐SA cells. Inhibition of MDR activity by the P‐gp inhibitor, verapamil, increased the fluorescence intensity only in MES‐SA/Dx5 cells (Fig. 7D). This confirmed the MDR phenotype in MES‐SA/Dx5 cells. VU‐0365114 had no effect on the fluorescence intensities of MES‐SA and MES‐SA/Dx5 cells (Fig. 7D), indicating that VU‐0365114 did not alter the MDR pump activity. A drug can stimulate ATPase activity if it is a substrate for transport by MDR transporters [51]. To confirm the above results, drug‐stimulated P‐gp ATPase activity assay was performed. Because verapamil is a MDR substrate analogy [52], it was used as a positive control for P‐gp ATPase activity assay. Indeed, verapamil and vinblastine, but not VU‐0365114, increased P‐gp ATPase activity (Fig. 7E). Therefore, VU‐0365114 is not an MDR substrate.

### 3.7 VU‐0365114 overcomes drug resistance in colorectal cancer cells

**Fig. 8:**
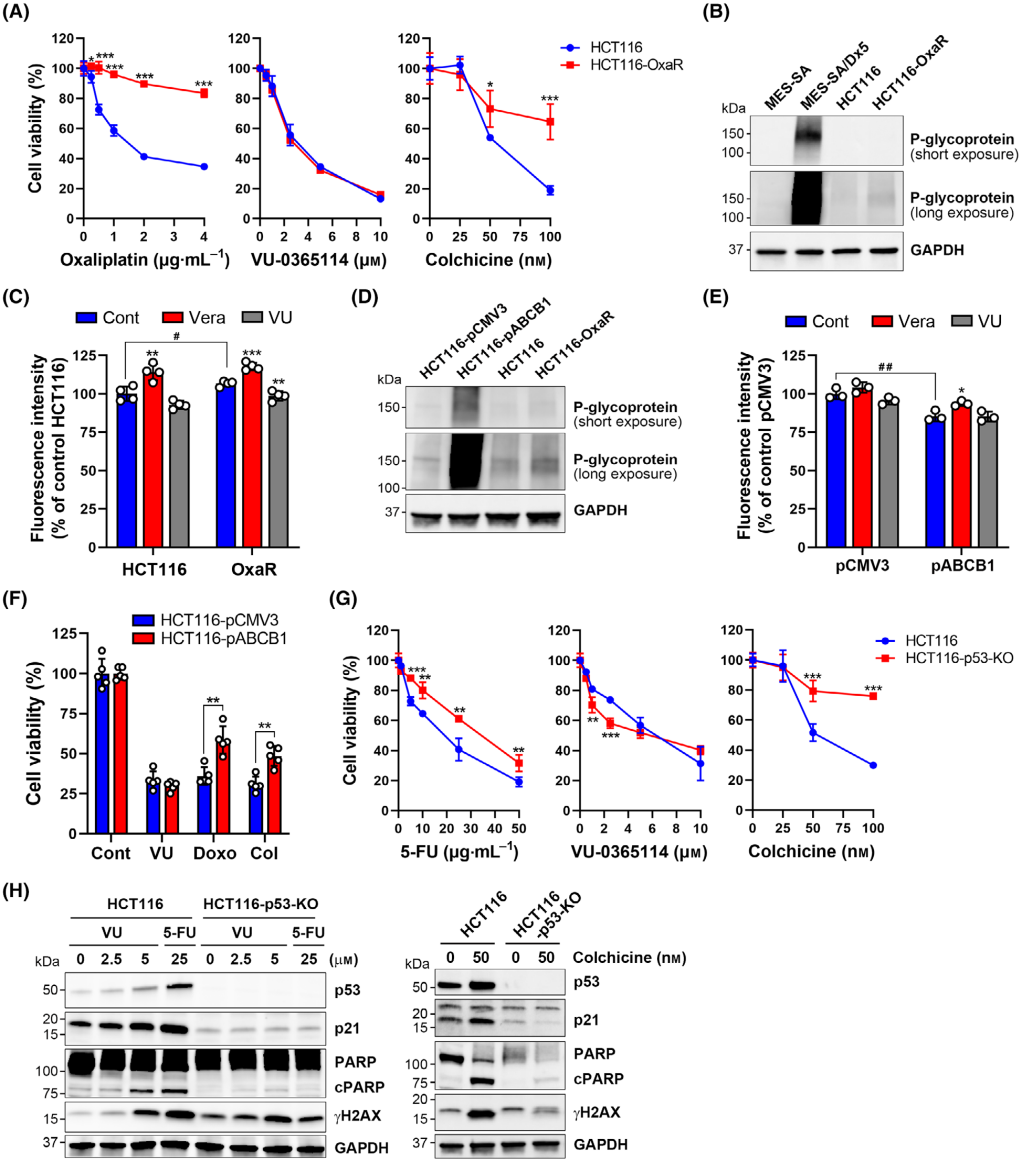
The effect of VU‐0365114 on drug resistance in colorectal cancer cells. (A) HCT116 and HCT116‐OxaR cells were treated with various concentrations of oxaliplatin, VU‐0365114, and colchicine for 72 h. Then, cell viability was examined by an MTT assay. The error bars are the mean ± SD (*n* = 4). Statistical significance, compared to parental HCT116 cells at each dose (*P < 0.05 and ***P < 0.001), was determined using a two‐tailed paired Student's *t*‐test. (B) The P‐glycoprotein (P‐gp) expression in MES‐SA, MES‐SA/Dx5, HCT116, and HCT116‐OxaR cells were examined by Western blotting. Data represent two independent experiments. (C) HCT116 and HCT116‐OxaR cells were treated with verapamil (Vera; 10 μm) or VU‐0365114 (VU; 10 μm) for 2 h. Then, an MDR assay was performed. The error bars are the mean ± SD (*n* = 4). Statistical significance, compared to untreated controls (**P < 0.01 and ***P < 0.001), was determined using a one‐way ANOVA with Tukey's *post hoc* test. Statistical significance, compared between HCT116 and HCT116‐OxaR cells (^#^ *P* < 0.05), was determined using a two‐tailed paired Student's *t*‐test. (D) The P‐gp expression in pCMV3‐ and pCMV3‐ABCB1 (pABCB1)‐transfected HCT116, as well as HCT116 and HCT116‐OxaR cells were examined by Western blotting. Data represent two independent experiments. (E) pCMV3‐ and pABCB1‐transfected HCT116 cells were treated with verapamil (10 μm) or VU‐0365114 (10 μm) for 2 h. Then, an MDR assay was performed. The error bars are the mean ± SD (*n* = 3). Statistical significance, compared to untreated controls (*P < 0.05), was determined using a one‐way ANOVA with Tukey's *post hoc* test. Statistical significance, compared between pCMV3‐ and pABCB1‐transfected HCT116 cells (^##^ *P* < 0.01), was determined using a two‐tailed paired Student's *t*‐test. (F) pCMV3‐ and pABCB1‐transfected HCT116 cells were treated with VU‐0365114 (10 μm), doxorubicin (Doxo; 0.25 μm), and colchicine (Col; 100 nm) for 72 h. Then, cell viability was examined by an MTT assay. The error bars are the mean ± SD (*n* = 5). Statistical significance, compared to pCMV3‐transfected HCT116 cells at each treatment (**P < 0.01), was determined using a two‐tailed paired Student's *t*‐test. (G) Parental and p53‐knockout HCT116 (HCT116‐p53‐KO) cells were treated with various concentrations of 5‐fluorouracil (5‐FU), VU‐0365114, and colchicine for 72 h. Then, cell viability was examined by an MTT assay. The error bars are the mean ± SD (*n* = 4). Statistical significance, compared to parental HCT116 cells at each dose (**P < 0.01 and ***P < 0.001), was determined using a two‐tailed paired Student's *t*‐test. (H) HCT116 and HCT116‐p53‐KO cells were treated with the indicated concentrations of 5‐fluorouracil (5‐FU), VU‐0365114 (VU), and colchicine for 48 h. Then, protein expressions were examined by Western blotting. Data represent two independent experiments.

To further confirm the overcoming effect of VU‐0365114 on drug resistance in colorectal cancer cells, we employed oxaliplatin‐resistant HCT116 (HCT116‐OxaR) cells and validated their phenotype using an MTT cell viability assay (Fig. 8A, left panel). We observed a similar cytotoxicity induced by VU‐0365114 in both cell lines (Fig. 8A, middle panel), while noting cross‐resistance to colchicine in HCT116‐OxaR cells (Fig. 8A, right panel). However, unlike MES‐SA/Dx5 cells (Fig. 7B,D), we observed only a slight increase in P‐gp expression in HCT116‐OxaR cells (Fig. 8B). Moreover, the fluorescence intensity of the MDR indicator did not reduce in HCT116‐OxaR cells compared to HCT116 cells (Fig. 8C). These findings suggest that MDR overexpression may not be the primary mechanism underlying the oxaliplatin‐resistant phenotype of HCT116‐OxaR cells.

To further corroborate the impact of VU‐0365114 on MDR, we transiently transfected HCT116 cells with an *ABCB1*‐encoding plasmid. We confirmed P‐gp overexpression using Western blotting (Fig. 8D) and verified the increased P‐gp pump activity by observing a reduction in the fluorescence intensity of the MDR indicator (Fig. 8E). Similar to its effects on MES‐SA and MES‐SA/Dx5 cells (Fig. 7D), VU‐0365114 did not influence MDR pump activity (Fig. 8E). Additionally, P‐gp overexpression led to HCT116 cell resistance against doxorubicin and colchicine, but not against VU‐0365114 (Fig. 8F). Thus, VU‐0365114 demonstrates potential for overcoming MDR in colorectal cancer cells.

Loss of the tumor suppressor protein p53 frequently occurs in cancers, which contributes to drug resistance [53]. p53‐knockout (p53‐KO) HCT116 cells were more resistant to 5‐FU and colchicine, but not VU‐0365114 (Fig. 8G). Chemotherapeutic agents usually induce DNA damage and activate the p53/p21 signaling pathway, leading to cell‐cycle arrest and apoptosis [54]. Indeed, 5‐FU and colchicine induced γH2AX (a DNA damage marker), p53, p21, and PARP cleavage in parental, but not in p53‐KO HCT116 cells (Fig. 8H). Interestingly, VU‐0365114 also induced p53‐dependent p21 expression and PARP cleavage. However, VU‐0365114‐induced γH2AX was not abolished in p53‐KO HCT116 cells (Fig. 8H). Therefore, VU‐0365114 may also overcome drug resistance in p53‐deficient cancer cells via activating alternative cell‐death pathways.

### 3.8 NCI‐60 COMPARE analysis revealed the potential mechanisms of action (MOAs) for VU‐0365114

**Fig. 9:**
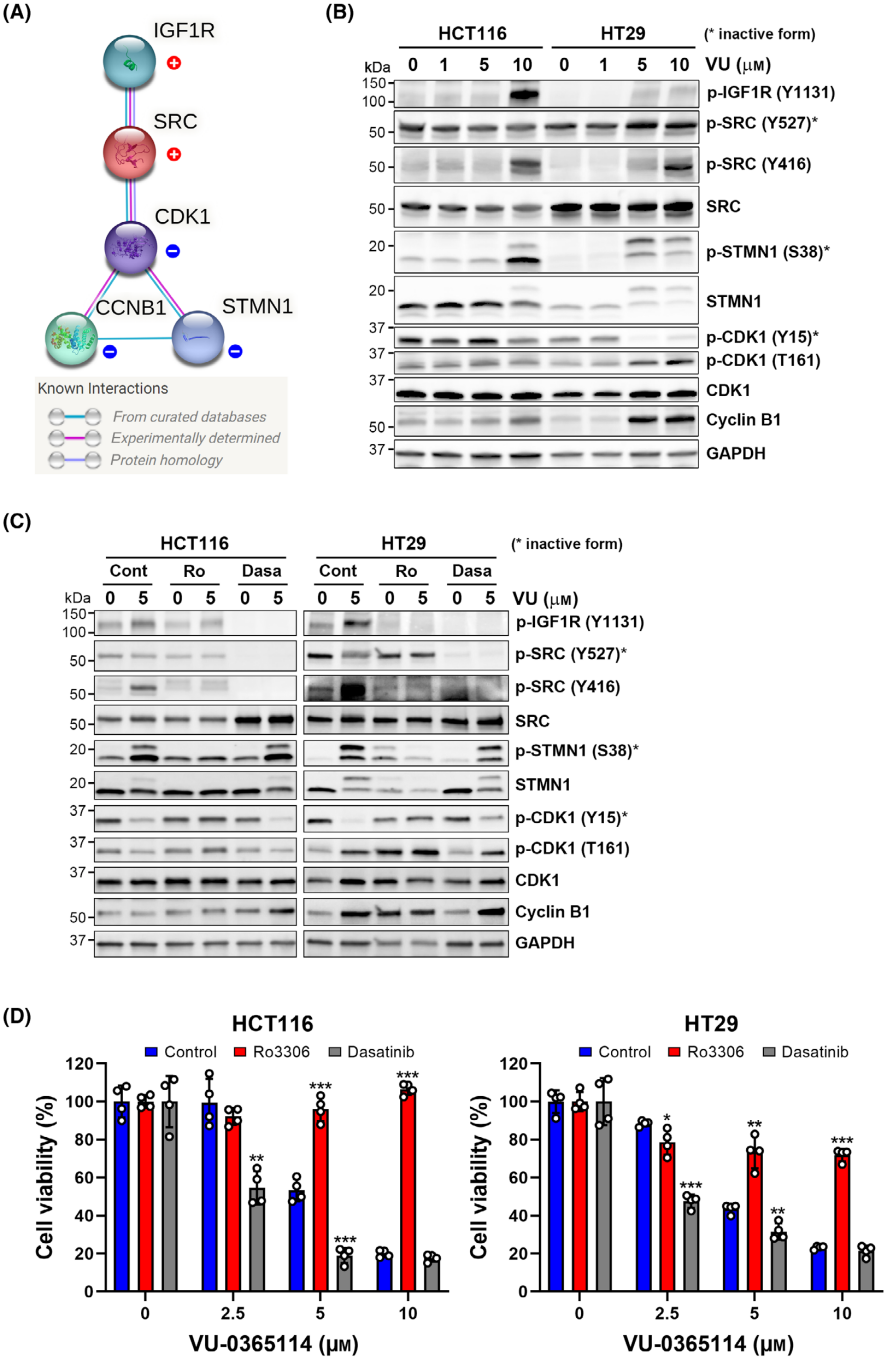
Exploration for the mechanisms of action (MOAs) of VU‐0365114. (A) The network for these molecules in Table 1 was constructed using the STRING database. (B) HCT116 and HT29 cells were treated with the indicated concentrations of VU‐0365114 (VU) for 24 h. Then, protein expressions were examined by Western blotting. Data represent two independent experiments. (C) HCT116 and HT29 cells were treated with 5 μm VU‐0365114 (VU) for 24 h in the absence or presence of Ro3306 (Ro; 5 μm) or dasatinib (Dasa; 100 nm). The protein expressions were examined by Western blotting. Data represent two independent experiments. (D) HCT116 and HT29 cells were treated with various concentrations of VU‐0365114 for 72 h in the absence or presence of Ro3306 (5 μm) or dasatinib (100 nm). The cell viability was examined by an MTT assay. The error bars are the mean ± SD (*n* = 5). The original data (Fig. S10) were normalized to untreated control, Ro3306, or dasatinib. Statistical significance, compared to VU‐0365114‐treated cells at each dose point (*P < 0.05, **P < 0.01, and ***P < 0.001), was determined using a two‐tailed paired Student's *t*‐test.

To investigate the underlying MOAs for VU‐0365114, a molecular target COMPARE [55] analysis based on the NCI‐60‐screening results (Fig. 4G) was performed. Molecules positively and negatively correlated with cell growth ability of cancer cells after VU‐0365114 treatment were shown in Table 1. The network for these molecules was constructed using the STRING database [16]. We found that insulin‐like growth factor 1 receptor (IGF1R), SRC, cyclin‐dependent kinase 1 (CDK1), cyclin B1 (CCNB1), and stathmin 1 (STMN1) formed a signaling network (Fig. 9A). Consistent with the microtubule‐targeting activity of VU‐0365114, cancer cells with higher levels of mitotic regulators (CDK1, CCNB1, and STMN1) were more sensitive to VU‐0365114 (Table 1, Fig. 9A). The CDK1‐cyclin B1 complex triggers the cell cycle into mitosis and is required for maintaining a mitotic state [41]. Stathmin 1, also known as oncoprotein 18 (Op18), exhibits microtubule‐destabilizing activity either by promoting microtubule catastrophe or by sequestering free tubulin dimers. At the onset of mitosis, stathmin 1 activity is inhibited by CDK1‐mediated phosphorylation at Ser25 and Ser38, allowing the correct formation of the mitotic spindle [56]. Mitosis‐arrested cells may either die by apoptosis during mitosis (mitotic cell death) or exit from mitosis without cytokinesis (mitotic slippage) and then escape from cell death [8]. It was reported that SRC phosphorylates CDK1 at Tyr15 and then cause mitotic slippage, leading to resistance of cancer cells to microtubule‐targeting agents [57]. Interestingly, cancer cells with higher SRC expression were more resistant to VU‐0365114 (Table 1, Fig. 9A). However, examination of the phosphorylation and expression of these molecules in various cancer cell lines did not reveal a significant correlation with the anticancer activity of VU‐0365114 (Fig. S8). It is possible that a larger set of cell lines should be used to reach a definitive conclusion, or multiple factors may collaborate to determine the sensitivities of cancer cell lines towards VU‐0365114. Additionally, it is conceivable that VU‐0365114 might influence the expression and/or activity of these molecules.

**Table 1:** COMPARE analysis of the NCI‐60 cancer cell panel revealed the mechanisms of action (MOAs) of VU‐0365114. Molecular targets that correlate with the Log (IC_50_) values of VU‐0365114 in the NCI‐60 cancer cell panel were analyzed using the COMPARE algorithm. The predicted molecular targets with absolute values of Pearson's correlation coefficients (PCCs) > 0.4 are shown.

PCC	ID	Molecular target	Full name
0.482	MT18293	IGF1R	Insulin‐like growth factor 1 receptor
0.476	MT198	ALDH3A1 (enzyme activity)	Aldehyde dehydrogenase 3 family member A1
0.462	MT18261	Phosphorylation of SRC protein at Tyr527	SRC proto‐oncogene, non‐receptor tyrosine kinase
0.445	MT18263	SRC	SRC proto‐oncogene, non‐receptor tyrosine kinase
−0.402	MT18272	CCNB1	Cyclin B1
−0.403	MT1655	CBS	Cystathionine‐beta‐synthase
−0.413	MT18314	STMN1	Stathmin 1
−0.415	MT18249	EIF4EBP1	Eukaryotic translation initiation factor 4E binding protein 1
−0.422	MT1126	CDC2 (CDK1)	Cyclin‐dependent kinase 1
−0.427	MT1692	MCM7	Minichromosome maintenance complex component 7
−0.479	MT1158	CDC2 (CDK1)	Cyclin‐dependent kinase 1

To clarify the MOAs of VU‐0365114, the effects of VU‐0365114 on the expression and activity of CDK1‐cyclin B1, stathmin 1, IGF1R, and SRC in HCT116 and HT29 cells were further examined by Western blotting. As shown in Fig. 9B, stabilization of cyclin B1 and dephosphorylation of CDK1 at Tyr15 (an inactivating site) in HCT116 and HT29 cells, as well as phosphorylation of CDK1 at Tyr161 (an activating site) in HT29 cells, indicated that CDK1 was activated by VU‐0365114. In addition, VU‐0365114 inactivated stathmin 1 as indicated by phosphorylation at Ser38. The above results indicated that VU‐0365114 induced mitotic arrest in HCT116 and HT29 cells, as supported by the increases in the mitotic index (Fig. S7). Although COMPARE analysis indicated that the inactive form of SRC (phosphorylated at Tyr527) was a resistant factor for VU‐0365114 (Table 1), we found that VU‐0365114 also activated the IGF1R/SRC signaling axis, as indicated by phosphorylation at Tyr1131 (IGF1R) and Tyr416 (SRC) (Fig. 9B). This may act as a compensatory response to VU‐0365114's antimitotic activity. However, the level of phospho‐Tyr15‐CDK1, which is the SRC phosphorylation site [57], was attenuated by VU‐0365114 (Fig. 9B). This suggests that the active SRC did not reduce CDK1 activity and cause mitotic slippage.

To further confirm the roles of CDK1‐cyclin B1/stathmin 1 and the IGF1R/SRC signaling network, inhibitors targeting CDK1 (Ro3306) and SRC (dasatinib) were employed. As illustrated in Fig. 9C, Ro3306 effectively inhibited CDK1 activity, evident from the restoration of phospho‐Tyr15 CDK1 and the reduction of phospho‐Ser38 stathmin 1. Inhibition of SRC activity by dasatinib was confirmed by the decrease in phospho‐Tyr416 SRC. Notably, dasatinib had no impact on CDK1 activity. However, similar to dasatinib, Ro3306 also suppressed the phosphorylation of both SRC and IGF1R. We proceeded to investigate the effects of Ro3306 and dasatinib on the cytotoxicity of VU‐0365114 in HCT116 and HT29 cells. Interestingly, Ro3306 exhibited an antagonistic anticancer effect when combined with VU‐0365114, while dasatinib enhanced the anticancer activity of VU‐0365114 (Fig. 9D, Fig. S10). This could potentially be attributed to the inhibition of CDK1 by Ro3306, which hampers mitotic entry, consequently preventing VU‐0365114‐induced mitotic arrest and subsequent cell death. Therefore, inhibition of SRC activity and/or activation of CDK1 could sensitize human colorectal cancer cells to VU‐0365114. Considering that HepG2/C3A displayed the highest resistance to VU‐0365114 treatment among the tested cell lines (Fig. 4A), we further investigated the combined effect of dasatinib and VU‐0365114 in HepG2/C3A cells. However, no synergistic anticancer activity was observed (Fig. S11), indicating that alternative mechanisms could be implicated in the intrinsic resistance of HepG2/C3A to VU‐0365114.

### 3.9 A kinome analysis reveals that VU‐0365114 does not exhibit other significant off‐target effects

**Fig. 10:**
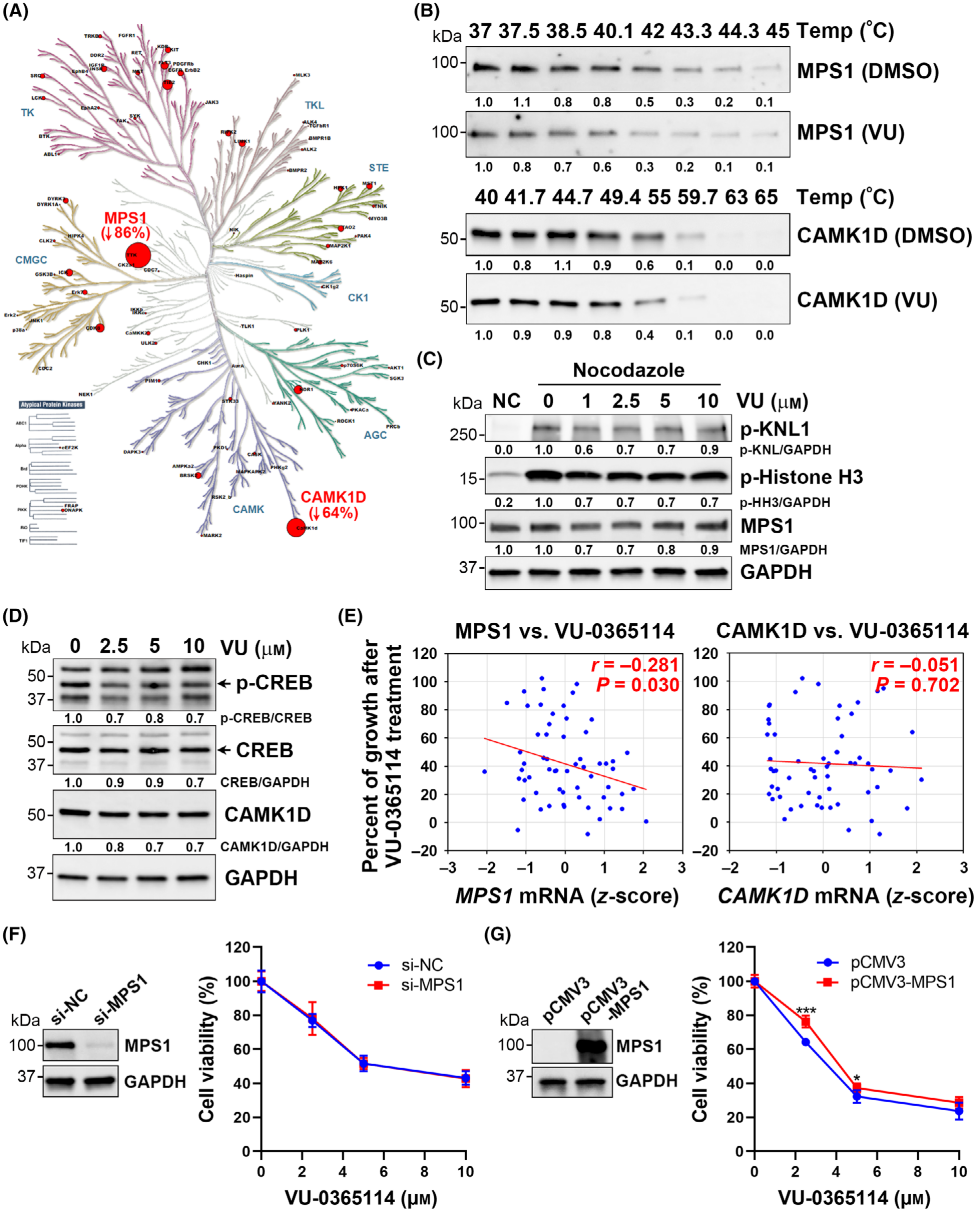
The role of monopolar spindle 1 (MPS1) in the anticancer activity of VU‐0365114. (A) The inhibitory activity of VU‐0365114 on 89 kinases (SelectScreen Kinase Profiling Services, ThermoFisher Scientific). A kinome tree was generated using KinMap (http://www.kinhub.org/kinmap/). Illustration reproduced courtesy of Cell Signaling Technology, Inc. (www.cellsignal.com). An enlarged image was provided in Fig. S12. (B) HCT116 cells were treated with 10 μm VU‐0365114 (VU) for 1 h, and then a cellular thermal shift assay (CETSA) was performed to examine the thermal stability of MPS1 and CAMK1D. Data represent three independent experiments. (C) HCT116 cells were treated with 100 ng mL^−1^ nocodazole for 18 h, and then exposed to various concentrations of VU‐0365114 (VU) for 1 h. Next, protein expressions were examined by Western blotting. (D) HCT116 cells were treated with VU‐0365114 (VU) for 24 h, and protein expressions were examined by Western blotting. Data represent three independent experiments. (E) Correlations of *MPS1* or *CAMK1D* mRNA with VU‐0365114‐induced growth inhibition in NCI‐60 cell panel. The *MPS1* and *CAMK1D* mRNA levels in NCI‐60 cancer cells were obtained from the CellMinerCDB database. Statistical significance was determined using the Pearson's correlation coefficient (*r*) and the corresponding *P* value. (F, G) HCT116 cells were transfected with *MPS1* siRNA in F or plasmid in G for 48 h, and then exposed to VU‐0365114 for 72 h. MPS1‐knockdown in F or MPS1‐overexpression in G was confirmed by Western blotting (left panel). Cell viability was examined by an MTT assay (right panel). The error bars are the mean ± SD (*n* = 5). Statistical significance, compared to the transfection control group at each dose (*P < 0.05 and ***P < 0.001), was determined using a two‐tailed paired Student's *t*‐test. si‐NC, negative control siRNA.

To further explore the pharmacological or off‐target effects of VU‐0365114, a kinome analysis was performed. Among them, monopolar spindle 1 (MPS1) and calcium/calmodulin‐dependent protein kinase ID (CAMK1D) were identified as potential targets of VU‐0365114 (Fig. 10A, Fig. S12). The physical interaction of VU‐0365114 with MPS1 or CAMK1D in cells was examined by a cellular thermal shift assay (CETSA) that compared the thermal stabilization of proteins in ligand‐free and ligand‐bound states [20]. Ligand‐bound proteins were more resistant to heating‐induced denaturation and precipitation than unbound proteins [20]. However, treatment of HCT116 cells with VU‐0365115 did not significantly alter the thermal stability of MPS1 or CAMK1D (Fig. 10B). We proceeded to evaluate the impact of VU‐0365114 on cellular MPS1 activity by examining the phosphorylation of kinetochore scaffold 1 (KNL1), an MPS1 substrate [58], as well as histone H3 [59, 60, 61]. Nevertheless, a 1‐h treatment with VU‐0365114 did not lead to changes in the phosphorylation levels of KNL1 and histone H3 (Fig. S13A), which did increase after 24 h (Fig. S13B) due to cell accumulation in the mitotic phase (Fig. S7). Given the low basal level of phospho‐KNL1, we arrested HCT116 cells in the M phase using nocodazole to enrich for active MPS1. As depicted in Fig. 10C, the activation of MPS1 induced by nocodazole was only slightly attenuated by VU‐0365114. Similarly, VU‐0365114 mildly inhibited the phosphorylation of cAMP responsive element‐binding protein (CREB) (Fig. 10D), which is a potential substrate of CAMK1D [62]. Since NCI‐60 cancer cell lines with higher *MPS1* mRNA levels, but not *CAMK1D*, showed a weak association with sensitivity to VU‐0365114 treatment (Fig. 10E), further investigation was conducted to examine the role of MPS1 in the anticancer activity of VU‐0365114. However, knockdown or overexpression of MPS1 did not significantly affect the cytotoxicity of VU‐0365114 (Fig. 10F,G). Since we observed a modest restoration of VU‐0365114‐induced cytotoxicity upon MPS1 overexpression (Fig. 10G), we utilized an MPS1‐specific inhibitor (AZ3146 [63]) to gain further insight into the role of MPS1. Co‐treatment of AZ3146 with VU‐0365114 or colchicine yielded an antagonistic cytotoxic effect (Fig. S14), implying that MPS1 likely does not play a substantial role in the anticancer activities of microtubule‐destabilizing agents. Therefore, we concluded that VU‐0365114 did not exhibit other significant off‐target effects.

## 4 Discussion

Most clinically used microtubule‐targeting agents for cancer treatment belong to taxane‐ and vinca‐site drugs. Taxane‐site drugs are nanoparticle albumin‐bound‐paclitaxel, docetaxel, and cabazitaxel. Vinca‐site drugs are eribulin, vincristine, and vinorelbine. One exception is the antibody‐drug conjugate, trastuzumab emtansine that binds to the maytansine site. Although colchicine is an approved drug for gout treatment, no colchicine‐binding site inhibitors (CBSIs) are approved for cancer therapy due to such limitations as poor drug solubility, a narrow therapeutic window, severe adverse effects, and the development of drug resistance [64]. Our study identified VU‐0365114 as a novel type of CBSI, which may bypass the limitations of classical CBSIs. However, more investigations were needed to demonstrate its safety profile, such as hematologic parameters, cytochrome P450 (CYP) inhibition, and pharmacokinetic parameters. In addition, the biological activity of VU‐0365114 is relatively poor compared with current CBSIs. For example, the dosage of VU‐0365114 to inhibit *in vitro* cell viability of cancer cells is at the micromolar range (Fig. 4A), which is much higher than that (nanomolar range) of colchicine (Fig. 8A). In addition, VU‐0365114 only slightly slowed the *in vivo* tumor growth (Fig. 5A). Therefore, modification and optimization of VU‐0365114 are required for the development of more potent CBSIs.

Despite microtubule‐targeting agents belonging to a successful class of anticancer treatments, their clinical activities in colorectal cancer have been disappointing in phase I/II trials [65, 66, 67, 68, 69, 70]. Despite no clear explanation, a high incidence of chromosomal instability in this disease, coupled with alterations in spindle checkpoint regulators, may contribute to the disappointing results associated with taxane‐based therapies for colorectal cancer [71]. Another possibility is that colorectal cancer and the colorectal mucosa generally express high levels of P‐gp, which may contribute to the unresponsiveness of colorectal cancer patients to microtubule‐targeting agents [72]. We proposed that VU‐0365114 is not a P‐gp substrate and could overcome the above concerns and provide clinical benefits for colorectal cancer patients.

Although CMap is a powerful tool for drug repurposing, there are potential pitfalls associated with this approach, including low accuracy and reproducibility [73]. Several factors may contribute to these limitations. Firstly, the gene expression profiles generated for CMap analysis involve diverse compound concentrations and cell lines. Secondly, CMap analysis is solely based on gene expression and does not consider drug effects at the protein or metabolic level. Thirdly, drugs targeting the same functional category may elicit similar transcriptomic responses. For instance, PLK inhibitors also disrupt mitosis, which led to the prediction of several PLK inhibitors (ON‐01910, HMN‐214, and GW‐843682X) as potential microtubule‐targeting agents (Fig. 1). However, while ON‐01910 has been demonstrated as a microtubule‐destabilizing agent [32], our results showed that HMN‐214 had little effect and GW‐843682X had no effect on microtubule polymerization (Fig. 2B).

The L1000FWD map comprises 3237 drugs' gene signatures covering 68 distinct cell lines, 132 dosages, and 3 time points (6, 24, and 48 h) [12]. Although drugs with similar MOAs tend to cluster together, we observed that some gene signatures of drugs, including well‐known tubulin inhibitors, spread out the cluster region (Fig. 1B–D). This phenomenon suggests that compounds only cluster within a specific region at certain concentrations, time points, or cell lines. After carefully examining the L1000FWD data for tubulin inhibitors, we found that the gene signatures of drugs treated for 6 h tended to spread out the specific cluster region. It is possible that the 6‐h treatment is insufficient to generate the gene expression profile that represents the drug's MOA.

Although targeting microtubules is a desirable approach for cancer therapy, the indiscriminate modulation of tubulin by small molecules presents a significant challenge in developing compounds aimed at treating different targets and diseases [74]. The 50% effective concentration (EC_50_) of VU‐0365114 for M5 mAChR is 2.7 μm [35], while the concentrations shown to have an effect on microtubules were 5 and 10 μm. This indicates the possibility of an off‐target effect of VU‐0365114 on microtubules when screening for drugs using higher concentrations. Knockdown and overexpression of the original target of VU‐0365114 did not significantly change VU‐0365114's cytotoxicity (Fig. 6E,F, Fig. S9), supporting the possibility of off‐target effects. Since microtubules are one of the most frequent off‐targets of small molecules, unbiased approaches to assess compound selectivity are highly demanded during drug development. For example, morphological profiling by cell painting assay enables early identification of compounds with tubulin‐binding activities [74]. Our study also supports the potential use of CMap/L1000FWD analysis for detecting compounds' off‐target effects.

mAChRs, a subfamily of G protein‐coupled receptors, regulate numerous fundamental functions of the central and peripheral nervous systems. *CHRM5*‐knockout mice display decreased prepulse inhibition (a model of psychosis) and cognitive deficits associated with CNS neuronal and cerebrovascular abnormalities [75, 76]. Additionally, the impaired cholinergic dilation of cerebral blood vessels in *CHRM5*‐knockout mice implies that M5 mAChR may play a role in the pathophysiology of Alzheimer's disease and focal cerebral ischemia [77]. These studies highlight the therapeutic potential of positive allosteric M5 modulators or agonists in the treatment of numerous CNS diseases. While VU‐0365114 was synthesized as a positive allosteric M5 modulator, its efficacy in treating these neural diseases remains unclear. Furthermore, it is uncertain whether treatment with VU‐0365114 could affect brain function. This study demonstrated, for the first time, that VU‐0365114 did not show neurological toxicity based on a behavioral test using FOB.

The role of M5 mAChR in cancer is still unclear and controversial. The major reason is its low expression. By using Chinese hamster ovary cells stably expressing *CHRM5*, stimulation with the muscarinic agonists, such as carbachol (a stable analog of acetylcholine), can reverse transformed phenotypes through the activation of receptor‐operated calcium influx [78, 79]. These results suggest the tumor‐suppressive function of M5 mAChR. Similarly, activation of endogenous M5 mAChR in A2058 melanoma cell line by the muscarinic agonists also results in calcium influx and reduces anchorage‐independent tumor cell growth [80]. In contrast, although transfection with *CHRM5* in NIH 3T3 cells does not change cell morphology, treatment with carbachol results in the formation of transforming foci, suggesting that *CHRM5* is an agonist‐dependent oncogene [81]. Further investigations are necessary to definitively establish the role of M5 mAChR in cancers.

Stathmin 1 plays a role in destabilizing microtubules and regulating the process of microtubule polymerization and depolymerization. Particularly, it promotes microtubule depolymerization during interphase and late mitosis. At the beginning of mitosis, the proper formation of the mitotic spindle is ensured by inhibiting the activity of stathmin 1 through phosphorylation at Ser25 and Ser38, which is mediated by CDK1 [56]. Our findings indicate that VU‐0365114 activated CDK1 and inactivated stathmin 1 (Fig. 9B) and increased the mitotic index (Fig. S7), suggesting that cells are undergoing mitotic arrest. However, it is counterintuitive that stathmin 1 inactivation may facilitate the formation of the mitotic spindles. Possibly, the microtubule‐destabilizing activity of VU‐0365114 depolymerizes microtubules and disrupts mitotic spindle formation.

## 5 Conclusions

This study provides an analytic strategy for drug repositioning and demonstrates its practicality. We repurposed a positive allosteric modulator of M5 mAChR, VU‐0365114, as a novel microtubule inhibitor for treating colorectal cancer and overcoming drug resistance. We think that VU‐0365114 can be a lead compound for the development of more‐effective anticancer drugs in the future.

## Conflict of interest

The authors declare no conflict of interest.

## Author contributions

Y‐YH and J‐LD performed the experiments. Y‐YH. and P‐MY analyzed the data and draft the manuscript. P‐MY designed the experiments and supervised the study. All authors reviewed and approved the final version of the paper.

### Peer review

The peer review history for this article is available at https://www.webofscience.com/api/gateway/wos/peer‐review/10.1002/1878‐0261.13536.

## Supporting information


**Table S1.** The differentially expressed genes in VU‐0365114‐treated AsPC‐1 cells.
**Table S2.** The differentially expressed genes in VU‐0365114‐treated PANC‐1 cells.
**Table S3.** Parameters for functional observational battery (FOB).
**Table S4.** Functional observational battery (FOB) test result.
**Fig. S1.** An enlarged image of Fig. 1A.
**Fig. S2.** An enlarged image of Fig. 1B.
**Fig. S3.** L1000FWD visualization of drug‐gene signatures that did not show similarity to anti‐tubulin agents.
**Fig. S4.** Effects of two positive allosteric modulators of M5 mAChRM on *in vitro* tubulin polymerization.
**Fig. S5.** Effect of drugs on vinca tubulin‐binding.
**Fig. S6.** The original image for Fig. 2H.
**Fig. S7.** Mitotic index of drug‐treated cancer cells.
**Fig. S8.** Protein expressions in various human cancer cell lines.
**Fig. S9.** Effect of CHRM5 knockdown or overexpression on the cytotoxicity of VU‐0365114 in PANC‐1 cells.
**Fig. S10.** Effect of CDK1 and SRC inhibitors on the cytotoxicity of VU‐0365114 in colorectal cancer cells.
**Fig. S11.** Effect of a SRC inhibitor on the cytotoxicity of VU‐0365114 in HepG2/C3A cells.
**Fig. S12.** An enlarged image of Fig. 10A.
**Fig. S13.** Effect of VU‐0365114 on MPS1 activity in HCT116 cells.
**Fig. S14.** Effect of a MPS1 inhibitor on the cytotoxicity of VU‐0365114 or colchicine in HCT116 cells.

## Data Availability

RNA‐Seq data (GSE168020) were deposited to NCBI GEO database. Additional data supporting this study are available on request from the corresponding author.
