# Supplementary material for: Repositioning VU‐0365114 as a novel microtubule‐destabilizing agent for treating cancer and overcoming drug resistance
Source: Mol Oncol. 2023 Oct 22;18(2):386–414. doi: 10.1002/1878-0261.13536 (PMC10850822; doi:10.1002/1878-0261.13536)
Supplement: Supplementary file 1 — Table S1. The differentially expressed genes in VU‐0365114‐treated AsPC‐1 cells. Table S2. The differentially expressed genes in VU‐0365114‐treated PANC‐1 cells. Table S3. Parameters for functional observational battery (FOB). Table S4. Functional observational battery (FOB) test result. Fig. S1. An enlarged image of Fig. 1A. Fig. S2. An enlarged image of Fig. 1B. Fig. S3. L1000FWD visualization of drug‐gene signatures that did not show similarity to anti‐tubulin agents. Fig. S4. Effects of two positive allosteric modulators of M5 mAChRM on in vitro tubulin polymerization. Fig. S5. Effect of drugs on vinca tubulin‐binding. Fig. S6. The original image for Fig. 2H. Fig. S7. Mitotic index of drug‐treated cancer cells. Fig. S8. Protein expressions in various human cancer cell lines. Fig. S9. Effect of CHRM5 knockdown or overexpression on the cytotoxicity of VU‐0365114 in PANC‐1 cells. Fig. S10. Effect of CDK1 and SRC inhibitors on the cytotoxicity of VU‐0365114 in colorectal cancer cells. Fig. S11. Effect of a SRC inhibitor on the cytotoxicity of VU‐0365114 in HepG2/C3A cells. Fig. S12. An enlarged image of Fig. 10A. Fig. S13. Effect of VU‐0365114 on MPS1 activity in HCT116 cells. Fig. S14. Effect of a MPS1 inhibitor on the cytotoxicity of VU‐0365114 or colchicine in HCT116 cells. [file MOL2-18-386-s001.pdf]

Supporting Information for:

Repositioning VU-0365114 as a novel microtubule-destabilizing agent for treating cancer and overcoming drug resistance

Yao-Yu Hsieh [1,2,3,4], Jia-Ling Du [5], and Pei-Ming Yang [3,4,5,6,7,8,*]

[1] Division of Hematology and Oncology, Taipei Medical University Shuang Ho Hospital, New Taipei City, Taiwan

[2] Division of Hematology and Oncology, Department of Internal Medicine, School of Medicine, College of Medicine, Taipei Medical University, Taipei, Taiwan

[3] Taipei Cancer Center, Taipei Medical University, Taipei, Taiwan

[4] TMU and Affiliated Hospitals Pancreatic Cancer Groups, Taipei Medical University, Taipei, Taiwan

[5] Graduate Institute of Cancer Biology and Drug Discovery, College of Medical Science and Technology, Taipei Medical University, Taipei, Taiwan

[6] Ph.D. Program for Cancer Molecular Biology and Drug Discovery, College of Medical Science and Technology, Taipei Medical University, Taipei, Taiwan

[7] TMU Research Center of Cancer Translational Medicine, Taipei, Taiwan

[8] Cancer Center, Wan Fang Hospital, Taipei Medical University, Taipei, Taiwan

***Address correspondence to**: Dr. Pei-Ming Yang, Graduate Institute of Cancer Biology and Drug Discovery, College of Medical Science and Technology, Taipei Medical University, No. 301, Yuantong Rd., Zhonghe Dist., New Taipei City 235603, Taiwan; Phone: +886-2-66202589 ext. 11107; E-mail: yangpm@tmu.edu.tw

Supporting Information includes:

1. Table S1-S4

2. Figure S1-S14

Supplementary tables:

Table S1. The differentially expressed genes in VU-0365114-treated AsPC-1 cells.

Ensembl gene ID	Fold change (Log$_2$)	*p* value	*q* value	Gene name
ENSG00000169213	5.8917	1.08E-05	0.00041794	RAB3B
ENSG00000196611	5.0103	5.61E-06	0.00022836	MMP1
ENSG00000170498	4.8994	9.79E-05	0.0029403	KISS1
ENSG00000153162	3.7498	1.53E-10	1.31E-08	BMP6
ENSG00000238266	3.5877	1.83E-11	1.71E-09	LINC00707
ENSG00000163395	3.5212	5.55E-06	0.00022658	IGFN1
ENSG00000106366	3.5025	6.95E-71	8.80E-68	SERPINE1
ENSG00000118515	3.4979	3.75E-06	0.00015848	SGK1
ENSG00000120129	3.3628	1.04E-31	3.41E-29	DUSP1
ENSG00000175040	3.3403	5.09E-05	0.0016565	CHST2
ENSG00000161638	3.3227	2.59E-17	4.24E-15	ITGA5
ENSG00000114771	3.1576	2.68E-05	0.00093047	AADAC
ENSG00000118985	3.0338	2.36E-05	0.00083006	ELL2
ENSG00000147689	2.823	1.92E-271	3.90E-267	FAM83A
ENSG00000113070	2.777	6.74E-08	3.86E-06	HBEGF
ENSG00000204949	2.6734	5.34E-10	4.19E-08	FAM83A-AS1
ENSG00000073737	2.5297	3.60E-91	5.62E-88	DHRS9
ENSG00000029153	2.514	6.07E-37	2.41E-34	ARNTL2
ENSG00000140545	2.4818	1.38E-06	6.41E-05	MFGE8
ENSG00000123358	2.4455	8.26E-05	0.0025275	NR4A1
ENSG00000125740	2.4372	2.81E-05	0.00097278	FOSB
ENSG00000124107	2.3451	1.76E-11	1.66E-09	SLPI
ENSG00000068971	2.3035	8.35E-08	4.69E-06	PPP2R5B
ENSG00000133805	2.2887	5.87E-06	0.00023708	AMPD3
ENSG00000124762	2.2848	1.85E-28	5.14E-26	CDKN1A
ENSG00000240602	2.2704	3.48E-05	0.0011812	AADACP1
ENSG00000196878	2.1792	3.92E-154	1.32E-150	LAMB3
ENSG00000157227	2.17	3.13E-33	1.08E-30	MMP14
ENSG00000102312	2.1411	1.20E-06	5.65E-05	PORCN
ENSG00000139433	2.1344	1.03E-16	1.64E-14	GLTP
ENSG00000188549	2.0864	2.12E-24	5.06E-22	C15orf52
ENSG00000177169	2.0821	2.15E-08	1.35E-06	ULK1
ENSG00000137819	2.0601	1.57E-11	1.50E-09	PAQR5

ENSG00000136002	2.0468	1.22E-05	0.00046146	ARHGEF4
ENSG00000138623	2.0167	2.18E-05	0.00077481	SEMA7A
ENSG00000058085	2.01	4.89E-214	3.30E-210	LAMC2
ENSG00000116285	1.9883	1.54E-48	9.43E-46	ERRFI1
ENSG00000196352	1.9807	2.28E-103	4.61E-100	CD55
ENSG00000147394	1.9125	1.63E-06	7.45E-05	ZNF185
ENSG00000163975	1.907	6.11E-13	6.87E-11	MELTF
ENSG00000123689	1.8919	3.65E-06	0.00015479	G0S2
ENSG00000160181	1.8912	5.81E-05	0.0018525	TFF2
ENSG00000053747	1.8766	2.15E-114	4.83E-111	LAMA3
ENSG00000122861	1.8537	2.96E-53	2.14E-50	PLAU
ENSG00000145390	1.8517	1.11E-11	1.08E-09	USP53
ENSG00000033170	1.8423	4.45E-12	4.51E-10	FUT8
ENSG00000008517	1.8354	6.47E-08	3.72E-06	IL32
ENSG00000104419	1.8333	3.18E-25	7.75E-23	NDRG1
ENSG00000166825	1.8049	1.72E-51	1.12E-48	ANPEP
ENSG00000171680	1.7938	5.01E-14	6.34E-12	PLEKHG5
ENSG00000131016	1.7689	5.24E-163	2.12E-159	AKAP12
ENSG00000065357	1.7454	9.08E-19	1.63E-16	DGKA
ENSG00000178726	1.7388	6.05E-45	3.41E-42	THBD
ENSG00000148344	1.7355	5.91E-11	5.30E-09	PTGES
ENSG00000120738	1.7278	1.16E-11	1.12E-09	EGR1
ENSG00000103044	1.7212	1.01E-16	1.63E-14	HAS3
ENSG00000187800	1.7127	6.21E-35	2.25E-32	PEAR1
ENSG00000012171	1.7119	4.60E-23	1.02E-20	SEMA3B
ENSG00000113742	1.7043	3.55E-06	0.00015188	CPEB4
ENSG00000143322	1.6952	2.03E-12	2.14E-10	ABL2
ENSG00000143797	1.6947	4.78E-41	2.31E-38	MBOAT2
ENSG00000010818	1.6784	0.00017696	0.004926	HIVEP2
ENSG00000134954	1.6772	2.55E-31	8.19E-29	ETS1
ENSG00000124466	1.6701	7.90E-06	0.00031266	LYPD3
ENSG00000158825	1.6696	1.50E-12	1.61E-10	CDA
ENSG00000214176	1.658	5.96E-07	2.92E-05	PLEKHM1P1
ENSG00000183696	1.6485	3.42E-34	1.22E-31	UPP1
ENSG00000048052	1.6429	1.95E-05	0.00070318	HDAC9
ENSG00000167508	1.6403	1.63E-05	0.00059926	MVD
ENSG00000058668	1.6378	6.73E-39	2.90E-36	ATP2B4

ENSG00000084731	1.629	3.99E-06	0.0001682	KIF3C
ENSG00000128340	1.6175	1.81E-05	0.00065783	RAC2
ENSG00000103089	1.617	2.17E-09	1.57E-07	FA2H
ENSG00000142871	1.6048	3.68E-14	4.72E-12	CYR61
ENSG00000187678	1.5713	4.27E-29	1.20E-26	SPRY4
ENSG00000119801	1.5712	0.00013612	0.0039351	YPEL5
ENSG00000164171	1.5497	1.32E-51	8.93E-49	ITGA2
ENSG00000129451	1.5468	2.98E-56	2.41E-53	KLK10
ENSG00000079337	1.5344	3.81E-09	2.64E-07	RAPGEF3
ENSG00000198959	1.5332	3.06E-178	1.55E-174	TGM2
ENSG00000155252	1.5305	8.63E-06	0.00034042	PI4K2A
ENSG00000119986	1.5241	0.0001178	0.0034647	AVPI1
ENSG00000160360	1.5187	0.000151	0.0042976	GPSM1
ENSG00000150961	1.4903	3.84E-05	0.0012787	SEC24D
ENSG00000162591	1.4876	3.10E-05	0.0010694	MEGF6
ENSG00000059728	1.4876	1.78E-08	1.12E-06	MXD1
ENSG00000133639	1.4852	2.58E-05	0.00089862	BTG1
ENSG00000225190	1.4748	4.56E-10	3.64E-08	PLEKHM1
ENSG00000117525	1.4743	3.78E-57	3.33E-54	F3
ENSG00000119953	1.4709	1.71E-10	1.46E-08	SMNDC1
ENSG00000112033	1.4594	1.16E-05	0.0004448	PPARD
ENSG00000147065	1.4574	1.18E-70	1.40E-67	MSN
ENSG00000182795	1.4524	2.75E-12	2.86E-10	C1orf116
ENSG00000052802	1.4515	1.17E-07	6.45E-06	MSMO1
ENSG00000067082	1.4363	1.01E-38	4.25E-36	KLF6
ENSG00000165915	1.4337	6.64E-05	0.002093	SLC39A13
ENSG00000138678	1.4258	7.11E-11	6.32E-09	GPAT3
ENSG00000116604	1.4257	2.40E-12	2.51E-10	MEF2D
ENSG00000087074	1.421	1.56E-08	1.00E-06	PPP1R15A
ENSG00000105520	1.412	5.42E-05	0.0017376	PLPPR2
ENSG00000137802	1.4062	3.38E-05	0.0011538	MAPKBP1
ENSG00000095383	1.3988	3.57E-06	0.00015214	TBC1D2
ENSG00000148634	1.3949	7.48E-15	1.02E-12	HERC4
ENSG00000251562	1.3936	6.68E-38	2.76E-35	MALAT1
ENSG00000109321	1.3753	2.37E-12	2.49E-10	AREG
ENSG00000185022	1.3713	1.62E-07	8.78E-06	MAFF
ENSG00000130522	1.3702	2.91E-06	0.00012618	JUND

ENSG00000141458	1.3689	1.09E-13	1.35E-11	NPC1
ENSG00000198853	1.3607	2.83E-09	2.01E-07	RUSC2
ENSG00000110888	1.3592	1.46E-21	2.96E-19	CAPRIN2
ENSG00000251322	1.3584	4.28E-07	2.14E-05	SHANK3
ENSG00000188910	1.355	4.68E-10	3.72E-08	GJB3
ENSG00000173210	1.3546	7.10E-07	3.45E-05	ABLIM3
ENSG00000164120	1.3506	4.89E-08	2.88E-06	HPGD
ENSG00000070404	1.3357	8.32E-16	1.24E-13	FSTL3
ENSG00000135926	1.333	6.87E-44	3.48E-41	TMBIM1
ENSG00000118263	1.3287	2.32E-05	0.00082017	KLF7
ENSG00000019549	1.3267	2.04E-07	1.08E-05	SNAI2
ENSG00000171223	1.3206	3.88E-08	2.32E-06	JUNB
ENSG00000090006	1.3088	1.32E-06	6.14E-05	LTBP4
ENSG00000143878	1.306	4.14E-15	5.79E-13	RHOB
ENSG00000163814	1.3007	2.79E-26	7.35E-24	CDCP1
ENSG00000143384	1.2868	3.53E-55	2.75E-52	MCL1
ENSG00000178882	1.2834	6.24E-09	4.15E-07	RFLNA
ENSG00000104549	1.2819	1.45E-08	9.36E-07	SQLE
ENSG00000100196	1.2724	0.000151	0.0042976	KDELR3
ENSG00000112972	1.2704	4.45E-07	2.22E-05	HMGCS1
ENSG00000100558	1.2585	1.39E-12	1.52E-10	PLEK2
ENSG00000002587	1.2436	2.08E-05	0.00074559	HS3ST1
ENSG00000170537	1.2405	5.00E-07	2.47E-05	TMC7
ENSG00000178038	1.2359	2.62E-07	1.36E-05	ALS2CL
ENSG00000214655	1.227	0.00012437	0.0036263	ZSWIM8
ENSG00000151651	1.2232	2.72E-08	1.67E-06	ADAM8
ENSG00000171206	1.2208	9.59E-11	8.34E-09	TRIM8
ENSG00000115295	1.2201	8.11E-05	0.0024989	CLIP4
ENSG00000175592	1.2148	8.01E-19	1.46E-16	FOSL1
ENSG00000176597	1.2134	5.22E-06	0.00021494	B3GNT5
ENSG00000147168	1.2069	5.40E-05	0.0017374	IL2RG
ENSG00000057704	1.2049	1.69E-05	0.00061936	TMCC3
ENSG00000188643	1.2039	1.63E-29	4.66E-27	S100A16
ENSG00000185262	1.1999	3.78E-05	0.0012626	UBALD2
ENSG00000071054	1.199	7.10E-52	4.96E-49	MAP4K4
ENSG00000101246	1.1987	1.98E-13	2.37E-11	ARFRP1
ENSG00000135047	1.1965	6.36E-08	3.66E-06	CTSL

ENSG00000067064	1.1956	1.93E-05	0.00069823	IDI1
ENSG00000076928	1.1866	1.30E-13	1.59E-11	ARHGEF1
ENSG00000155366	1.1848	2.45E-11	2.27E-09	RHOC
ENSG00000157827	1.1841	4.15E-13	4.75E-11	FMNL2
ENSG00000108055	1.183	1.35E-25	3.34E-23	SMC3
ENSG00000011422	1.1827	4.74E-14	6.04E-12	PLAUR
ENSG00000181191	1.182	9.71E-06	0.0003764	PJA1
ENSG00000101782	1.1734	2.15E-16	3.33E-14	RIOK3
ENSG00000198142	1.169	3.57E-06	0.0001521	SOWAHC
ENSG00000142949	1.1669	2.85E-56	2.40E-53	PTPRF
ENSG00000102265	1.1642	1.87E-12	1.99E-10	TIMP1
ENSG00000089060	1.1624	6.60E-05	0.0020851	SLC8B1
ENSG00000071859	1.1602	6.08E-08	3.52E-06	FAM50A
ENSG00000116191	1.1558	3.03E-15	4.33E-13	RALGPS2
ENSG00000089327	1.1533	4.07E-08	2.43E-06	FXYD5
ENSG00000198431	1.15	1.00E-124	2.53E-121	TXNRD1
ENSG00000115648	1.1492	2.38E-08	1.49E-06	MLPH
ENSG00000079385	1.1366	1.12E-11	1.08E-09	CEACAM1
ENSG00000114019	1.1342	3.65E-07	1.87E-05	AMOTL2
ENSG00000114480	1.1326	0.00012334	0.0036068	GBE1
ENSG00000003436	1.1265	3.43E-07	1.76E-05	TFPI
ENSG00000129657	1.1252	5.56E-07	2.73E-05	SEC14L1
ENSG00000140941	1.1225	7.94E-05	0.0024614	MAP1LC3B
ENSG00000245532	1.122	3.33E-31	1.04E-28	NEAT1
ENSG00000172216	1.1212	1.68E-08	1.07E-06	CEBPB
ENSG00000085063	1.1197	1.13E-44	6.01E-42	CD59
ENSG00000116741	1.1195	5.02E-10	3.96E-08	RGS2
ENSG00000167114	1.1166	8.58E-05	0.0026059	SLC27A4
ENSG00000116260	1.1126	1.33E-13	1.62E-11	QSOX1
ENSG00000114738	1.1016	6.25E-07	3.05E-05	MAPKAPK3
ENSG00000130827	1.1001	4.81E-07	2.40E-05	PLXNA3
ENSG00000082153	1.0989	2.36E-40	1.11E-37	BZW1
ENSG00000102316	1.0887	0.00011095	0.0032968	MAGED2
ENSG00000178104	1.0836	8.22E-05	0.002521	PDE4DIP
ENSG00000147454	1.0799	3.13E-12	3.21E-10	SLC25A37
ENSG00000148672	1.0763	4.28E-16	6.47E-14	GLUD1
ENSG00000151012	1.0742	5.32E-09	3.57E-07	SLC7A11

ENSG00000071967	1.07	3.85E-10	3.08E-08	CYBRD1
ENSG00000147010	1.0675	4.09E-09	2.81E-07	SH3KBP1
ENSG00000128595	1.0669	2.56E-27	6.93E-25	CALU
ENSG00000139289	1.0642	2.81E-31	8.91E-29	PHLDA1
ENSG00000075413	1.0638	6.48E-06	0.00025943	MARK3
ENSG00000148426	1.0632	4.86E-05	0.0015844	PROSER2
ENSG00000196428	1.0622	3.55E-05	0.0012013	TSC22D2
ENSG00000184792	1.0488	8.42E-10	6.47E-08	OSBP2
ENSG00000164951	1.0487	9.07E-07	4.31E-05	PDP1
ENSG00000135678	1.0483	5.29E-05	0.0017091	CPM
ENSG00000131069	1.0482	2.91E-06	0.00012618	ACSS2
ENSG00000173530	1.0418	7.57E-06	0.00030119	TNFRSF10D
ENSG00000159363	1.0399	8.64E-10	6.61E-08	ATP13A2
ENSG00000102359	1.0363	3.96E-05	0.0013115	SRPX2
ENSG00000065054	1.0345	9.15E-16	1.34E-13	SLC9A3R2
ENSG00000254166	1.0262	1.34E-07	7.31E-06	CASC19
ENSG00000115758	1.0197	1.06E-19	2.01E-17	ODC1
ENSG00000145623	1.0189	1.13E-08	7.40E-07	OSMR
ENSG00000167779	1.0073	3.13E-08	1.90E-06	IGFBP6
ENSG00000075391	1.0043	3.05E-10	2.47E-08	RASAL2
ENSG00000155744	1.001	2.89E-06	0.00012616	FAM126B
ENSG00000013573	-1.0014	3.08E-12	3.17E-10	DDX11
ENSG00000184992	-1.0056	6.20E-08	3.58E-06	BRI3BP
ENSG00000087586	-1.0058	1.21E-09	9.08E-08	AURKA
ENSG00000090889	-1.0069	7.23E-08	4.10E-06	KIF4A
ENSG00000149418	-1.0126	3.05E-15	4.33E-13	ST14
ENSG00000149503	-1.016	1.06E-06	5.05E-05	INCENP
ENSG00000143375	-1.0221	2.76E-05	0.0009582	CGN
ENSG00000228716	-1.0235	2.78E-10	2.27E-08	DHFR
ENSG00000143924	-1.0239	2.48E-13	2.92E-11	EML4
ENSG00000111331	-1.025	3.89E-07	1.96E-05	OAS3
ENSG00000153944	-1.0319	7.88E-06	0.00031248	MSI2
ENSG00000164611	-1.0365	1.45E-05	0.00054181	PTTG1
ENSG00000011426	-1.0389	3.78E-19	7.03E-17	ANLN
ENSG00000170312	-1.04	5.73E-11	5.16E-09	CDK1
ENSG00000169679	-1.0465	1.82E-08	1.14E-06	BUB1
ENSG00000262655	-1.0477	2.35E-16	3.61E-14	SPON1

ENSG00000137807	-1.0482	2.12E-05	0.0007593	KIF23
ENSG00000243566	-1.0508	1.72E-11	1.63E-09	UPK3B
ENSG00000135048	-1.0515	1.22E-06	5.73E-05	TMEM2
ENSG00000041982	-1.0605	2.65E-10	2.19E-08	TNC
ENSG00000148737	-1.0646	1.13E-05	0.00043539	TCF7L2
ENSG00000066279	-1.066	1.89E-09	1.38E-07	ASPM
ENSG00000170540	-1.0669	7.73E-18	1.32E-15	ARL6IP1
ENSG00000160796	-1.068	9.15E-11	7.99E-09	NBEAL2
ENSG00000135373	-1.0698	1.96E-16	3.07E-14	EHF
ENSG00000188486	-1.0711	3.16E-07	1.63E-05	H2AFX
ENSG00000156970	-1.0736	4.26E-05	0.0013979	BUB1B
ENSG00000171617	-1.0824	2.15E-16	3.33E-14	ENC1
ENSG00000168078	-1.0831	7.19E-05	0.0022417	PBK
ENSG00000049323	-1.0948	5.36E-06	0.00021977	LTBP1
ENSG00000129422	-1.0989	2.37E-17	3.91E-15	MTUS1
ENSG00000205213	-1.1042	3.26E-10	2.63E-08	LGR4
ENSG00000121152	-1.1074	5.14E-05	0.0016705	NCAPH
ENSG00000118777	-1.1084	1.39E-09	1.03E-07	ABCG2
ENSG00000182481	-1.1085	9.36E-12	9.21E-10	KPNA2
ENSG00000137942	-1.1185	3.67E-05	0.001237	FNBP1L
ENSG00000168743	-1.119	7.76E-23	1.69E-20	NPNT
ENSG00000089685	-1.1217	1.41E-06	6.51E-05	BIRC5
ENSG00000136108	-1.1468	5.79E-06	0.00023494	CKAP2
ENSG00000145287	-1.1479	2.11E-18	3.68E-16	PLAC8
ENSG00000072571	-1.1489	5.72E-09	3.83E-07	HMMR
ENSG00000148773	-1.1557	6.22E-37	2.43E-34	MKI67
ENSG00000126351	-1.1577	7.18E-05	0.0022407	THRA
ENSG00000105289	-1.1645	5.53E-05	0.0017704	TJP3
ENSG00000163694	-1.1726	2.42E-07	1.26E-05	RBM47
ENSG00000163513	-1.1857	1.23E-25	3.09E-23	TGFBR2
ENSG00000113368	-1.1953	8.99E-11	7.88E-09	LMNB1
ENSG00000163362	-1.1981	2.30E-09	1.66E-07	INAVA
ENSG00000060566	-1.2167	5.92E-12	5.97E-10	CREB3L3
ENSG00000242265	-1.2218	1.37E-05	0.00051395	PEG10
ENSG00000178401	-1.2454	0.00014588	0.0041754	DNAJC22
ENSG00000106003	-1.2543	3.18E-06	0.00013754	LFNG
ENSG00000166866	-1.2583	5.28E-06	0.0002172	MYO1A

ENSG00000127824	-1.2617	2.34E-10	1.96E-08	TUBA4A
ENSG00000124225	-1.2637	1.39E-43	6.87E-41	PMEPA1
ENSG00000137834	-1.2668	2.41E-09	1.72E-07	SMAD6
ENSG00000275395	-1.2678	5.81E-06	0.00023544	FCGBP
ENSG00000164070	-1.269	0.00016472	0.0046362	HSPA4L
ENSG00000184661	-1.2697	7.92E-06	0.00031281	CDCA2
ENSG00000186193	-1.2964	4.06E-06	0.00017063	SAPCD2
ENSG00000124429	-1.3043	3.94E-08	2.36E-06	POF1B
ENSG00000136824	-1.3057	2.98E-08	1.82E-06	SMC2
ENSG00000072210	-1.3119	3.45E-22	7.12E-20	ALDH3A2
ENSG00000090382	-1.3163	1.55E-13	1.87E-11	LYZ
ENSG00000167553	-1.3202	2.14E-22	4.47E-20	TUBA1C
ENSG00000134291	-1.3241	4.97E-06	0.00020639	TMEM106C
ENSG00000028137	-1.3263	1.30E-12	1.43E-10	TNFRSF1B
ENSG00000145386	-1.3375	3.09E-08	1.88E-06	CCNA2
ENSG00000004799	-1.3397	5.25E-05	0.0017016	PDK4
ENSG00000162063	-1.3402	1.65E-06	7.54E-05	CCNF
ENSG00000137642	-1.344	2.56E-37	1.04E-34	SORL1
ENSG00000149328	-1.3446	3.88E-07	1.96E-05	GLB1L2
ENSG00000166897	-1.3454	3.13E-13	3.64E-11	ELFN2
ENSG00000122547	-1.3586	8.54E-11	7.52E-09	EEPD1
ENSG00000137804	-1.3609	1.40E-08	9.09E-07	NUSAP1
ENSG00000024526	-1.3632	1.53E-05	0.00056586	DEPDC1
ENSG00000122952	-1.3739	2.85E-11	2.62E-09	ZWINT
ENSG00000185630	-1.383	8.04E-05	0.0024825	PBX1
ENSG00000204616	-1.3956	1.89E-05	0.00068519	TRIM31
ENSG00000122644	-1.3973	1.75E-05	0.00063919	ARL4A
ENSG00000196975	-1.4017	1.90E-58	1.75E-55	ANXA4
ENSG00000134057	-1.4047	1.52E-22	3.21E-20	CCNB1
ENSG00000138160	-1.4086	1.03E-14	1.39E-12	KIF11
ENSG00000142945	-1.4142	2.23E-06	9.96E-05	KIF2C
ENSG00000010292	-1.4174	1.20E-18	2.12E-16	NCAPD2
ENSG00000168453	-1.428	4.09E-07	2.06E-05	HR
ENSG00000176532	-1.455	0.00012009	0.0035219	PRR15
ENSG00000106066	-1.4605	3.30E-08	1.99E-06	CPVL
ENSG00000186340	-1.4643	9.82E-10	7.48E-08	THBS2
ENSG00000166851	-1.4663	1.05E-10	9.11E-09	PLK1

ENSG00000154930	-1.4717	6.94E-06	0.00027742	ACSS1
ENSG00000167767	-1.5036	1.46E-06	6.70E-05	KRT80
ENSG00000112984	-1.5039	6.76E-09	4.47E-07	KIF20A
ENSG00000065534	-1.5176	1.73E-10	1.46E-08	MYLK
ENSG00000136205	-1.5429	7.23E-21	1.45E-18	TNS3
ENSG00000083857	-1.5487	8.89E-103	1.64E-99	FAT1
ENSG00000165140	-1.5674	1.61E-07	8.72E-06	FBP1
ENSG00000133131	-1.5752	2.44E-06	0.00010824	MORC4
ENSG00000147872	-1.6068	1.40E-05	0.00052213	PLIN2
ENSG00000189060	-1.6165	2.43E-62	2.34E-59	H1F0
ENSG00000114455	-1.6246	9.26E-06	0.00036166	HHLA2
ENSG00000127324	-1.6316	1.77E-46	1.06E-43	TSPAN8
ENSG00000119922	-1.6361	0.00014542	0.0041682	IFIT2
ENSG00000117650	-1.6375	1.04E-07	5.75E-06	NEK2
ENSG00000101447	-1.6473	2.45E-11	2.27E-09	FAM83D
ENSG00000170190	-1.6566	4.64E-09	3.13E-07	SLC16A5
ENSG00000104413	-1.6573	0.00015859	0.0044822	ESRP1
ENSG00000123416	-1.6633	3.43E-26	8.92E-24	TUBA1B
ENSG00000120885	-1.6666	7.87E-13	8.76E-11	CLU
ENSG00000137135	-1.6856	3.74E-05	0.0012512	ARHGEF39
ENSG00000121691	-1.7076	8.85E-07	4.22E-05	CAT
ENSG00000166387	-1.7221	6.04E-08	3.51E-06	PPFIBP2
ENSG00000148584	-1.7257	2.28E-07	1.20E-05	A1CF
ENSG00000153292	-1.7361	7.38E-30	2.20E-27	ADGRF1
ENSG00000165215	-1.737	2.30E-06	0.00010244	CLDN3
ENSG00000102897	-1.7388	5.30E-15	7.31E-13	LYRM1
ENSG00000101144	-1.7412	1.72E-07	9.20E-06	BMP7
ENSG00000132182	-1.7499	2.23E-06	9.96E-05	NUP210
ENSG00000143013	-1.7786	1.97E-12	2.09E-10	LMO4
ENSG00000101049	-1.784	0.00011293	0.0033457	SGK2
ENSG00000117724	-1.7983	7.97E-33	2.65E-30	CENPF
ENSG00000163762	-1.8042	2.48E-09	1.76E-07	TM4SF18
ENSG00000131747	-1.8293	2.90E-54	2.18E-51	TOP2A
ENSG00000109881	-1.8404	2.66E-06	0.00011742	CCDC34
ENSG00000162878	-1.878	1.70E-20	3.34E-18	PKDCC
ENSG00000198478	-1.8799	1.51E-05	0.00055868	SH3BGRL2
ENSG00000116574	-1.9008	2.05E-13	2.43E-11	RHOU

ENSG00000188229	-1.9999	2.05E-77	2.97E-74	TUBB4B
ENSG00000165868	-2.0036	1.31E-05	0.00049433	HSPA12A
ENSG00000138449	-2.0181	4.87E-06	0.00020284	SLC40A1
ENSG00000108375	-2.0672	8.52E-05	0.0025955	RNF43
ENSG00000196230	-2.0976	2.30E-100	3.89E-97	TUBB
ENSG00000127831	-2.1503	1.20E-17	2.00E-15	VIL1
ENSG00000183018	-2.2754	4.95E-30	1.52E-27	SPNS2
ENSG00000169035	-2.3631	0.00015809	0.0044744	KLK7
ENSG00000132437	-2.3752	3.24E-14	4.21E-12	DDC
ENSG00000175311	-2.3753	1.96E-05	0.00070534	ANKS4B
ENSG00000137673	-2.444	2.15E-33	7.51E-31	MMP7
ENSG00000169903	-2.4457	5.83E-06	0.00023602	TM4SF4
ENSG00000139292	-2.4769	6.74E-11	6.02E-09	LGR5
ENSG00000106541	-2.4801	6.46E-14	8.08E-12	AGR2
ENSG00000109255	-2.4809	1.02E-07	5.66E-06	NMU
ENSG00000125378	-2.5066	1.25E-36	4.78E-34	BMP4
ENSG00000108602	-2.5338	1.27E-10	1.09E-08	ALDH3A1
ENSG00000184156	-2.5562	1.60E-19	3.00E-17	KCNQ3
ENSG00000133067	-2.5646	7.97E-30	2.34E-27	LGR6
ENSG00000176153	-2.7355	4.55E-05	0.0014897	GPX2
ENSG00000170500	-2.7699	4.20E-13	4.78E-11	LONRF2
ENSG00000131771	-3.0167	6.04E-30	1.83E-27	PPP1R1B
ENSG00000104537	-3.0244	9.68E-05	0.002915	ANXA13
ENSG00000143167	-3.1424	7.56E-07	3.65E-05	GPA33
ENSG00000023171	-3.2284	1.81E-23	4.16E-21	GRAMD1B
ENSG00000135960	-3.3943	1.04E-14	1.40E-12	EDAR
ENSG00000100079	-3.7397	1.08E-09	8.10E-08	LGALS2
ENSG00000257743	-3.87	7.32E-26	1.85E-23	MGAM2

Table S2. The differentially expressed genes in VU-0365114-treated PANC-1 cells.

Ensembl gene ID	Fold change (Log_2)	*p* value	*q* value	Gene name
ENSG00000196611	3.9896	1.93E-07	2.29E-05	MMP1
ENSG00000178726	3.6347	1.27E-17	4.36E-15	THBD
ENSG00000173110	3.3719	8.31E-06	0.00061535	HSPA6
ENSG00000113070	3.1639	1.95E-18	7.32E-16	HBEGF
ENSG00000011422	2.8315	5.21E-06	0.00042464	PLAUR
ENSG00000198959	2.8095	1.78E-77	8.70E-74	TGM2
ENSG00000159167	2.7281	3.20E-05	0.0020453	STC1
ENSG00000249992	2.6339	4.15E-13	9.91E-11	TMEM158
ENSG00000008517	2.544	4.21E-06	0.00035332	IL32
ENSG00000140379	2.5368	1.19E-05	0.00083597	BCL2A1
ENSG00000175352	2.2185	1.50E-41	2.66E-38	NRIP3
ENSG00000132561	2.1854	5.91E-05	0.0035463	MATN2
ENSG00000147394	2.1294	1.24E-18	4.86E-16	ZNF185
ENSG00000133805	2.0903	9.35E-07	9.14E-05	AMPD3
ENSG00000128567	2.0798	1.45E-32	1.78E-29	PODXL
ENSG00000171680	2.0708	1.78E-07	2.17E-05	PLEKHG5
ENSG00000104419	2.0176	6.54E-06	0.00051203	NDRG1
ENSG00000138166	1.9896	1.23E-20	6.15E-18	DUSP5
ENSG00000058085	1.9686	1.21E-35	1.57E-32	LAMC2
ENSG00000100558	1.9016	3.51E-06	0.00030511	PLEK2
ENSG00000153531	1.8909	2.41E-06	0.0002184	ADPRHL1
ENSG00000085117	1.7868	4.42E-06	0.00036974	CD82
ENSG00000177169	1.7769	1.90E-27	1.81E-24	ULK1
ENSG00000183760	1.772	3.39E-15	9.83E-13	ACP7
ENSG00000163814	1.7363	6.76E-61	2.64E-57	CDCP1
ENSG00000176170	1.7319	2.76E-09	4.39E-07	SPHK1
ENSG00000171608	1.6229	4.26E-07	4.49E-05	PIK3CD
ENSG00000225190	1.6072	6.91E-08	8.78E-06	PLEKHM1
ENSG00000128591	1.5885	1.44E-09	2.39E-07	FLNC
ENSG00000124762	1.5869	2.43E-13	5.87E-11	CDKN1A
ENSG00000128512	1.5851	1.50E-05	0.0010377	DOCK4
ENSG00000144583	1.5754	8.47E-06	0.00062018	MARCH4
ENSG00000150782	1.5695	4.58E-06	0.00038086	IL18
ENSG00000177606	1.5621	1.91E-24	1.38E-21	JUN
ENSG00000153162	1.5062	1.78E-09	2.90E-07	BMP6
ENSG00000145247	1.466	8.63E-06	0.00062983	OCIAD2
ENSG00000117143	1.4339	1.67E-23	1.12E-20	UAP1
ENSG00000100139	1.433	4.59E-07	4.75E-05	MICALL1

ENSG00000029153	1.4316	2.63E-18	9.71E-16	ARNTL2
ENSG00000172216	1.416	7.65E-07	7.68E-05	CEBPB
ENSG00000103044	1.3784	3.30E-12	7.01E-10	HAS3
ENSG00000116717	1.3735	2.07E-09	3.32E-07	GADD45A
ENSG00000106366	1.3629	8.36E-149	1.64E-144	SERPINE1
ENSG00000108691	1.3423	2.42E-07	2.77E-05	CCL2
ENSG00000128342	1.3271	8.07E-10	1.41E-07	LIF
ENSG00000176597	1.3176	3.39E-07	3.77E-05	B3GNT5
ENSG00000059758	1.2908	2.13E-06	0.00019599	CDK17
ENSG00000167772	1.2836	5.01E-09	7.72E-07	ANGPTL4
ENSG00000188643	1.282	2.25E-08	2.93E-06	S100A16
ENSG00000205336	1.2763	1.06E-05	0.00075503	ADGRG1
ENSG00000058668	1.2549	9.74E-09	1.39E-06	ATP2B4
ENSG00000164597	1.2475	3.61E-05	0.0022832	COG5
ENSG00000189410	1.2208	6.35E-10	1.13E-07	SH2D5
ENSG00000101457	1.1546	3.53E-05	0.0022429	DNTTIP1
ENSG00000100292	1.1509	2.07E-12	4.50E-10	HMOX1
ENSG00000196923	1.1409	2.13E-06	0.00019599	PDLIM7
ENSG00000156463	1.1237	4.05E-05	0.0025345	SH3RF2
ENSG00000110888	1.115	1.26E-07	1.57E-05	CAPRIN2
ENSG00000130758	1.1086	1.32E-21	7.40E-19	MAP3K10
ENSG00000166833	1.1003	1.21E-07	1.52E-05	NAV2
ENSG00000189143	1.0986	2.27E-10	4.18E-08	CLDN4
ENSG00000160213	1.0882	2.07E-12	4.50E-10	CSTB
ENSG00000147065	1.0837	1.19E-41	2.33E-38	MSN
ENSG00000134954	1.0817	8.30E-14	2.11E-11	ETS1
ENSG00000120129	1.0743	6.91E-05	0.0040329	DUSP1
ENSG00000161638	1.0726	2.49E-20	1.16E-17	ITGA5
ENSG00000107957	1.0687	5.14E-06	0.00042085	SH3PXD2A
ENSG00000181649	1.044	6.59E-11	1.29E-08	PHLDA2
ENSG00000116285	1.018	1.09E-31	1.26E-28	ERRFI1
ENSG00000166825	1.0127	1.63E-42	3.54E-39	ANPEP
ENSG00000143322	1.0125	1.98E-08	2.66E-06	ABL2
ENSG00000173559	1.009	7.31E-07	7.37E-05	NABP1
ENSG00000173801	-1.0046	6.46E-10	1.14E-07	JUP
ENSG00000134508	-1.0208	6.96E-05	0.0040509	CABLES1
ENSG00000138061	-1.023	6.91E-20	3.00E-17	CYP1B1
ENSG00000137804	-1.0367	4.18E-08	5.34E-06	NUSAP1
ENSG00000166106	-1.0646	4.76E-06	0.00039435	ADAMTS15
ENSG00000188229	-1.0673	6.01E-21	3.18E-18	TUBB4B
ENSG00000185504	-1.0833	9.04E-09	1.31E-06	FAAP100

ENSG00000183337	-1.0849	9.49E-06	0.00068231	BCOR
ENSG00000182253	-1.1023	3.03E-06	0.00026479	SYNM
ENSG00000170540	-1.1274	1.53E-26	1.30E-23	ARL6IP1
ENSG00000117650	-1.1374	8.39E-06	0.00061859	NEK2
ENSG00000171604	-1.1402	7.29E-06	0.00054662	CXXC5
ENSG00000100311	-1.1894	4.34E-05	0.0026596	PDGFB
ENSG00000143320	-1.1996	5.17E-15	1.47E-12	CRABP2
ENSG00000123080	-1.2341	2.36E-07	2.73E-05	CDKN2C
ENSG00000184371	-1.2441	2.84E-17	9.26E-15	CSF1
ENSG00000123416	-1.2535	1.39E-17	4.69E-15	TUBA1B
ENSG00000184058	-1.2986	6.29E-05	0.0037416	TBX1
ENSG00000196230	-1.32	3.21E-87	3.14E-83	TUBB
ENSG00000131747	-1.3879	5.69E-80	3.71E-76	TOP2A
ENSG00000130600	-1.4499	2.74E-07	3.09E-05	H19
ENSG00000140511	-1.4539	1.62E-09	2.67E-07	HAPLN3
ENSG00000141753	-1.4803	1.84E-21	9.97E-19	IGFBP4
ENSG00000145287	-1.4914	3.53E-06	0.00030511	PLAC8
ENSG00000185215	-1.4933	4.87E-20	2.21E-17	TNFAIP2
ENSG00000163083	-1.5787	6.50E-07	6.65E-05	INHBB
ENSG00000108821	-1.6273	1.62E-06	0.00015249	COL1A1
ENSG00000106541	-1.6336	5.14E-13	1.21E-10	AGR2
ENSG00000167767	-1.6423	8.70E-11	1.69E-08	KRT80
ENSG00000088836	-1.6772	1.14E-06	0.00011051	SLC4A11
ENSG00000118898	-1.6789	5.33E-14	1.39E-11	PPL
ENSG00000132470	-1.6871	2.49E-06	0.00022324	ITGB4
ENSG00000071575	-1.6983	3.03E-05	0.0019492	TRIB2
ENSG00000138735	-1.7386	2.01E-10	3.82E-08	PDE5A
ENSG00000185585	-1.8828	6.08E-06	0.00048136	OLFML2A
ENSG00000170412	-1.8947	2.93E-07	3.30E-05	GPRC5C
ENSG00000079257	-2.107	1.03E-13	2.54E-11	LXN
ENSG00000137834	-2.247	4.03E-07	4.31E-05	SMAD6
ENSG00000174403	-2.7635	1.63E-07	2.00E-05	C20orf166-AS1

Table S3. Parameters for functional observational battery (FOB).

Neurology clinical sign	Definition and measurement
Undisturbed cage side observation	
Body position	S: sitting/standing<br>A: alert<br>AS: asleep<br>L: lying<br>F: flattered<br>C: catalepsy
Locomotor activity	NA: no activity or movement<br>R: resting and grooming<br>E: moderate exploration<br>D: darting around cage<br>ER: extremely restless
Fur coat	1: normal<br>2: slight dirtiness<br>3: moderate dirtiness<br>4: severe dirtiness and disorder
Ptosis	Degree of palpebral closure.<br>1: normal<br>2: <25%<br>3: 25~50%<br>4: > 50%
Exophthalmos	1: no<br>2: yes
Respiration rate	Breaths per 10 sec.<br>1: normal<br>2: slow (<10) or labored (<3) breathing<br>3: breathing heavily (<5)<br>4: rapid and shallow breathing (>30)<br>5: pause of breathing (<1)
Handling	
Grip strength	Forelimb/hindlimb grip strength.<br>–1: reduced<br>1: normal<br>2: increased
Open field observation	
Gait	N: normal<br>D: drag<br>H: hindlimbs exaggerated<br>HU: hunched<br>T: tiptoes<br>A: ataxia
Arousal	1: normal (vigilant or searching movements at all times)<br>2: moderate decrease (mild paralysis, only the head can move)<br>3: significant decrease (minimal or no response to

	environmental stimuli)<br>4: moderate increase (mild excitement/nervousness, may suddenly move forward or remain still)<br>5: significant increase (in a state of high alertness, suddenly moving or running)
Convulsions	1: normal<br>2: clonic<br>3: tonic<br>4: tonic-flexion
Tremors	1: normal<br>2: mild localized body tremor<br>3: moderate tremor, affecting walking<br>4: severe tremor, unable to walk
Stereotype	Repetitive behaviors, such as head shaking, chewing, licking, etc.<br>1: no<br>2: yes
Bizarre behaviors	Abnormal behaviors, such as tilting head, spinning, raised tail, walking backward, etc.<br>1: no<br>2: yes
Vocalization	1: no<br>2: yes
Piloerection	1: normal<br>2: slight piloerection observed on the head and back<br>3: piloerection all over the body
Salivation	1: normal/no drooling<br>2: a small amount of saliva appearing around the mouth<br>3: moderate drooling (contaminated to the face or neck)<br>4: severe drooling (also observed on the abdomen)
Lacrimation	1: normal/no tears<br>2: a small amount of discharge around the eyes<br>3: a large amount of discharge flowing from the eyes
Pupillary dilation or constriction	N: normal<br>D: dilation<br>C: constriction
Urination	Numbers within 3 min
Defecation	Numbers within 3 min
Diarrhea	Watery or unformed stool.<br>1: no<br>2: yes
Tail suspension test	
Tail suspension test	Animals were suspended by the tail with their heads approximately 10 cm above the ground, recorded for 5 min. The time until the animal completely ceased struggling was measured to assess the level of antidepressant effect of the drug.

Table S4. Functional observational battery (FOB) test result.

Category / Parameter	Control		VU-0365114	
	Before	After	Before	After
Undisturbed cage side observation				
Body position	S	S	S	S
Locomotor activity	R	R	R	R
Fur coat	1	1	1	1
Ptosis	1	1	1	1
Exophthalmos	1	1	1	1
Respiration rate	1	1	1	1
Handing				
Grip strength	1	1	1	1
Open field observation				
Gait	N	N	N	N
Arousal	1	1	1	1
Convulsions	1	1	1	1
Tremors	1	1	1	1
Stereotype	1	1	1	1
Bizarre behaviors	1	1	1	1
Vocalization	1	1	1	1
Piloerection	1	1	1	1
Salivation	1	1	1	1
Lacrimation	1	1	1	1
Pupillary dilation or constriction	N	N	N	N
Urination (times in average)	0.7±0.6	0	1.0±0.0	0
Defecation (times in average)	0.3±0.6	2.3±1.5	1.3±1.5	1.7±0.6
Diarrhea	1	1	1	1
Tail suspension test				
Tail suspension test (min in average)	> 5	> 5	> 5	> 5

Supplementary figures:

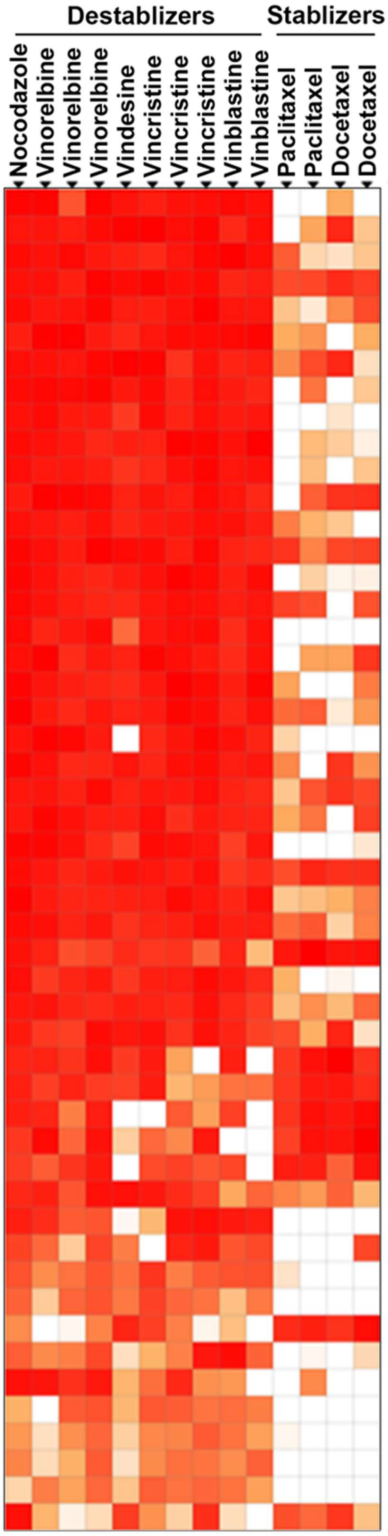

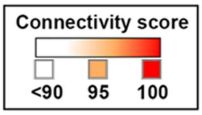

#	NAME	FUNCTION
1	Benperidol	Dopamine receptor antagonist
2	Vincristine	Microtubule destabilizer
3	Vinblastine	Microtubule destabilizer
4	Flubendazole	Microtubule destabilizer
5	Vincristine	Microtubule destabilizer
6	Vinorelbine	Microtubule destabilizer
7	Vindesine	Microtubule destabilizer
8	VU-0365114	M5 modulator
9	BRD-A16820783	Phosphodiesterase inhibitor
10	Vinblastine	Microtubule destabilizer
11	KF-38789	P-selectin inhibitor
12	Vinorelbine	Microtubule destabilizer
13	SA-63133	CK inhibitor/Microtubule destabilizer
14	Vinorelbine	Microtubule destabilizer
15	Podophyllotoxin	Microtubule destabilizer
16	Oxibendazole	Microtubule destabilizer
17	Prostratin	PKC activator
18	ON-01910	PLK inhibitor
19	Vincristine	Microtubule destabilizer
20	Nocodazole	Microtubule destabilizer
21	NPI-2358	Microtubule destabilizer
22	SA792574	Microtubule destabilizer
23	ABT-751	Microtubule destabilizer
24	HMN-214	PLK inhibitor
25	Mirin	MRE11A exonuclease inhibitor
26	Albendazole	Microtubule destabilizer
27	Mebendazole	Microtubule destabilizer
28	LY-2183240	FAAH inhibitor
29	Paclitaxel	Microtubule stabilizer
30	CMPD-1	p38 MAPK inhibitor
31	SB-225002	CC chemokine receptor antagonist
32	Fenbendazole	Microtubule destabilizer
33	Docetaxel	Microtubule stabilizer
34	Parbendazole	Microtubule destabilizer
35	Epothilone-a	Microtubule stabilizer
36	Docetaxel	Microtubule stabilizer
37	YK-4279	ETV1 inhibitor
38	Chelidonine	Microtubule destabilizer
39	Ingenol	PKC activator
40	BRD-A05680309	XIAP inhibitor
41	Helveticoside	ATPase inhibitor
42	Periplocymarin	Cardiac glycoside
43	Epothilone	Microtubule stabilizer
44	Cytochalasin-d	Actin polymerization inhibitor
45	Acitretin	Retinoid receptor agonist
46	Digitoxigenin	ATPase inhibitor
47	Digitoxin	ATPase inhibitor
48	Proscillaridin	ATPase inhibitor
49	Bufalin	ATPase inhibitor
50	GW-843682X	PLK inhibitor

Figure S1. An enlarged image for Figure 1A.

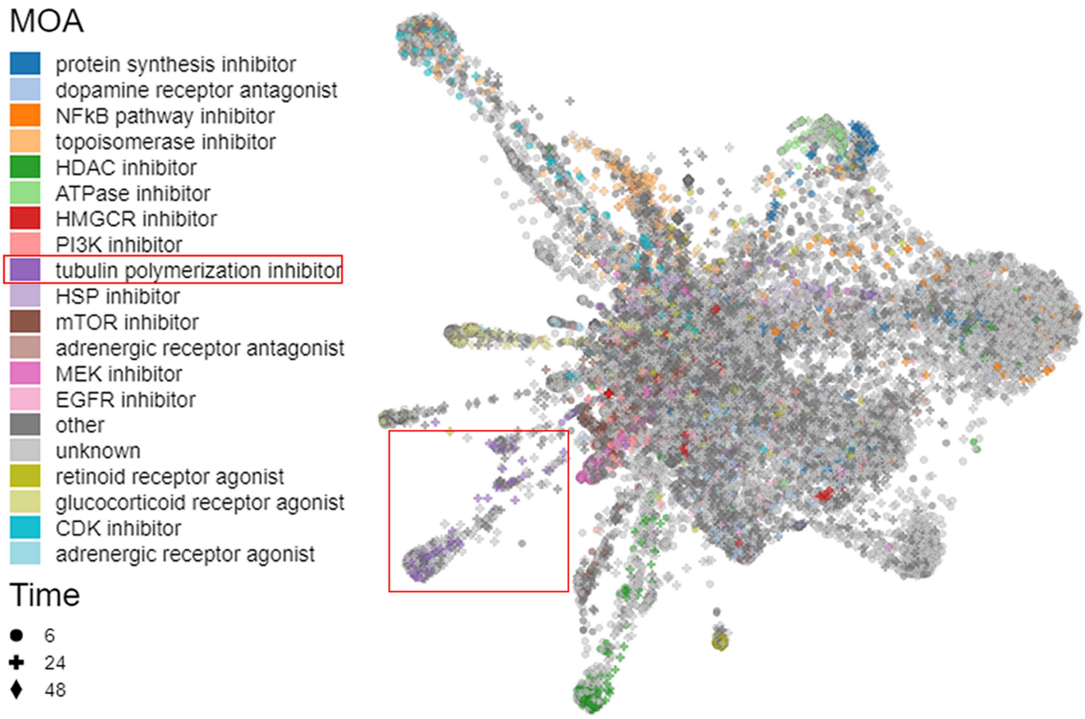

Figure S2. An enlarged image of Figure 1B.

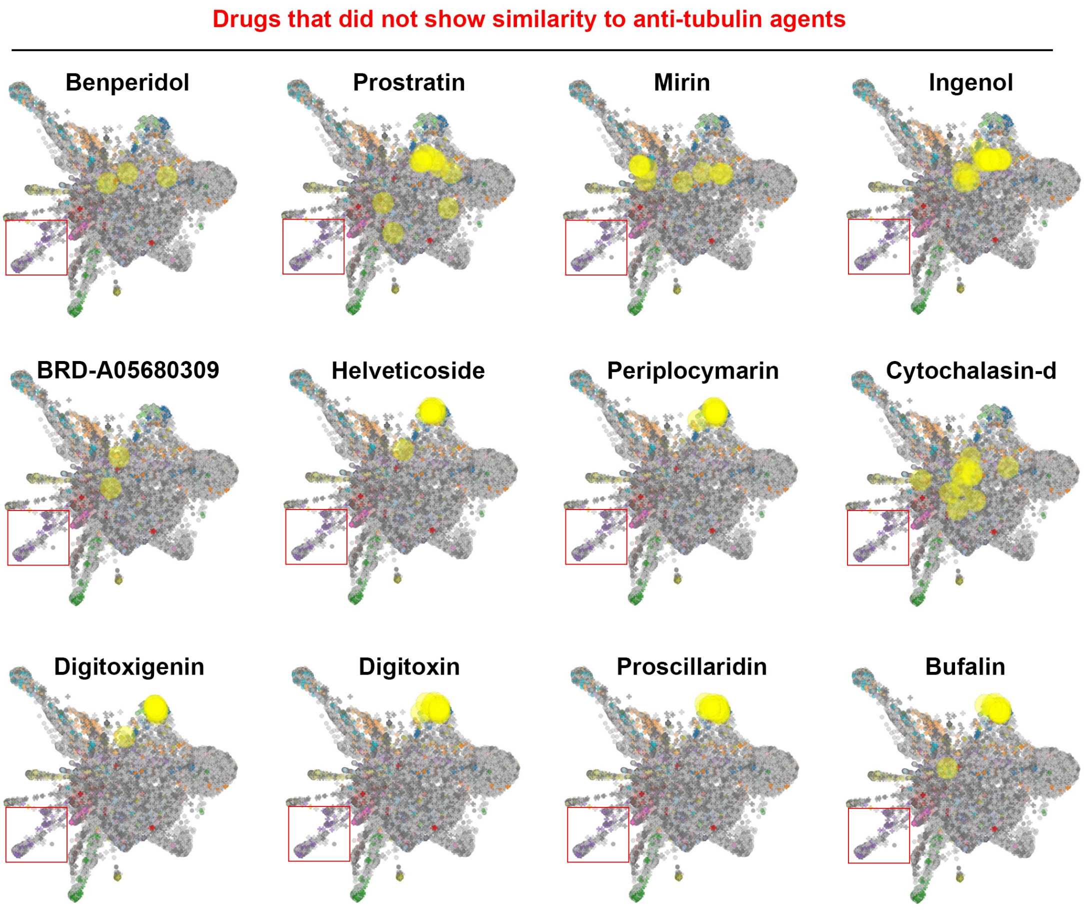

Figure S3. L1000FWD visualization of drug-gene signatures that did not show similarity to anti-tubulin agents. Each clustered point represents the gene signature of a drug in different cell lines, treated with varying doses and time intervals. The yellow circles highlight the queried drugs' gene signature.

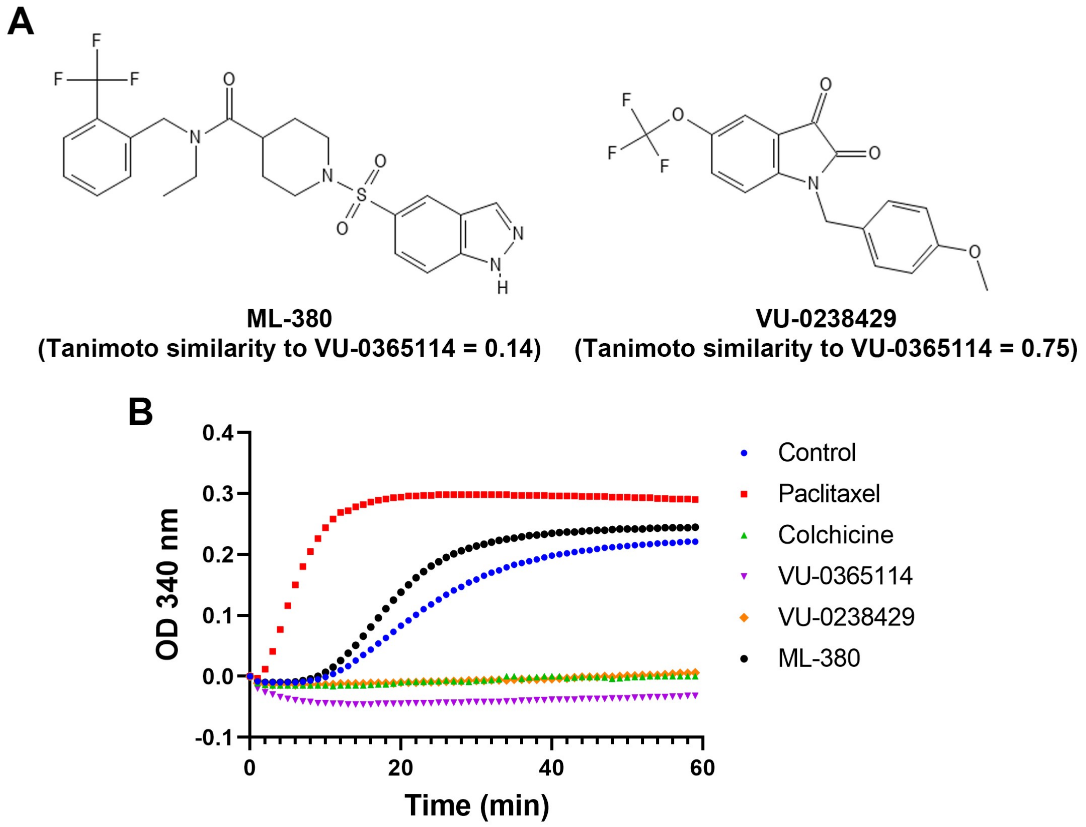

Figure S4. Effects of two positive allosteric modulators of M5 mAChRM on *in vitro* tubulin polymerization. (A) The chemical structures of ML-380 and VU-0238429. Their chemical similarities with VU-0365114 were calculated by Tanimoto coefficient. (B) Purified tubulins were incubated with 10 μM of drugs for 60 min. Tubulin polymerization was detected by measuring the absorbance at 340 nm.

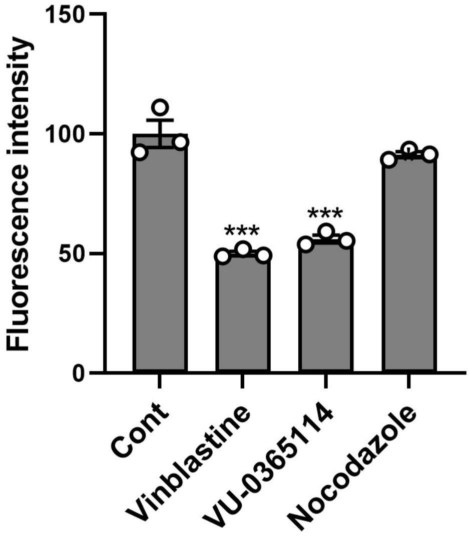

Figure S5. Effect of drugs on vinca tubulin-binding. The binding ability of vinblastine, VU-0365114, or nocodazole to vinca site was examined by the competition of tubulin-binding with BODIPY FL-vinblastine. The intrinsic fluorescence of the fluorescence of BODIPY FL-vinblastine was analyzed on a plate reader. The error bars are the mean ± SD (n = 3). Statistical significance, compared to untreated controls (***p<0.001), was determined using a one-way ANOVA with Tukey's post hoc test.

Figure S6. The original image for Figure 2H

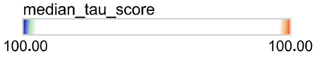

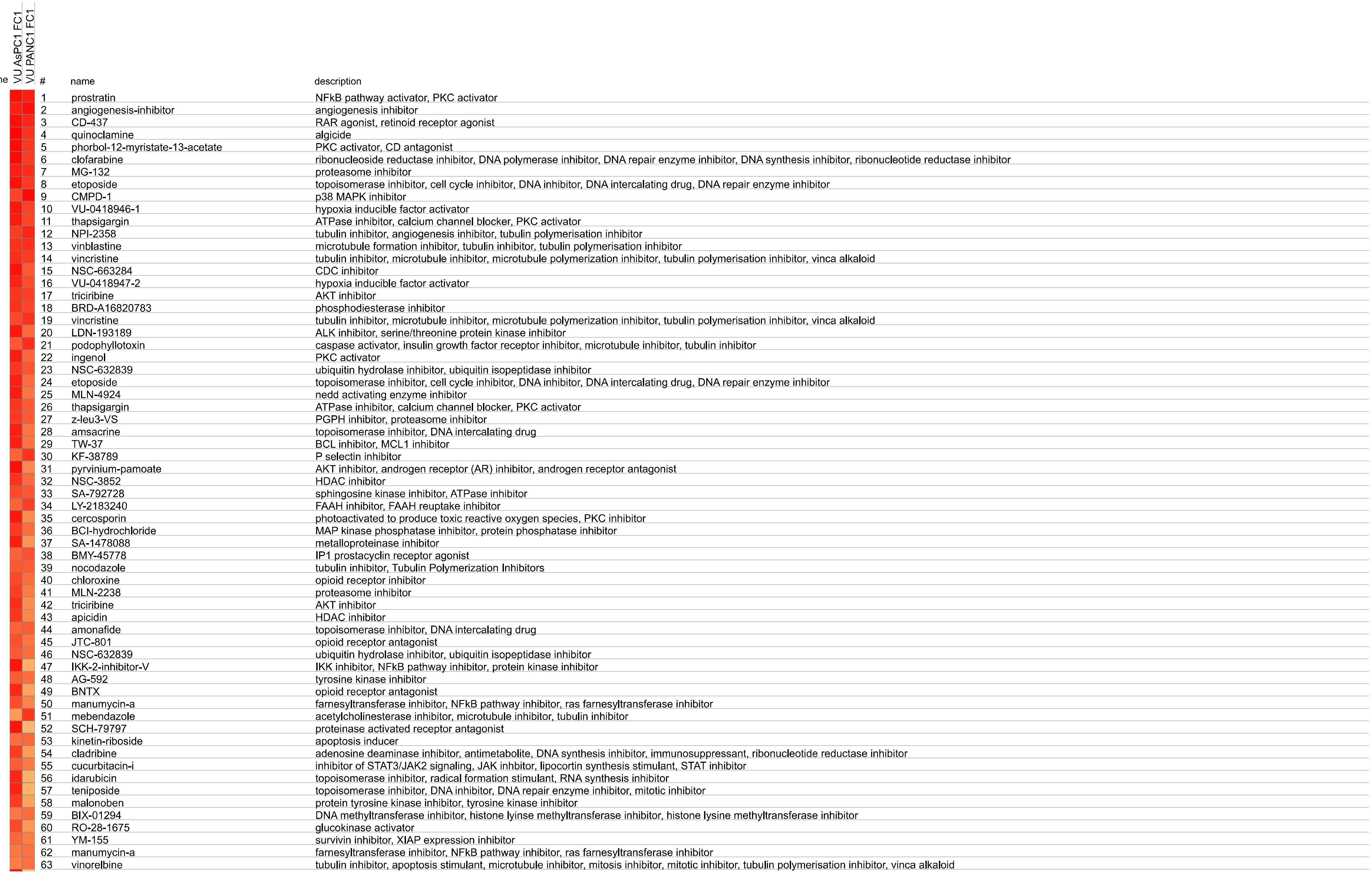

#	name	description
1	prostratin	NFkB pathway activator, PKC activator
2	angiogenesis-inhibitor	angiogenesis inhibitor
3	CD-437	RAR agonist, retinoid receptor agonist
4	quinoclamine	algicide
5	phorbol-12-myristate-13-acetate	PKC activator, CD antagonist
6	clofarabine	ribonucleoside reductase inhibitor, DNA polymerase inhibitor, DNA repair enzyme inhibitor, DNA synthesis inhibitor, ribonucleotide reductase inhibitor
7	MG-132	proteasome inhibitor
8	etoposide	topoisomerase inhibitor, cell cycle inhibitor, DNA inhibitor, DNA intercalating drug, DNA repair enzyme inhibitor
9	CMPD-1	p38 MAPK inhibitor
10	VU-0418946-1	hypoxia inducible factor activator
11	thapsigargin	ATPase inhibitor, calcium channel blocker, PKC activator
12	NPI-2358	tubulin inhibitor, angiogenesis inhibitor, tubulin polymerisation inhibitor
13	vinblastine	microtubule formation inhibitor, tubulin inhibitor, tubulin polymerisation inhibitor
14	vincristine	tubulin inhibitor, microtubule inhibitor, microtubule polymerization inhibitor, tubulin polymerisation inhibitor, vinca alkaloid
15	NSC-663284	CDC inhibitor
16	VU-0418947-2	hypoxia inducible factor activator
17	triciribine	AKT inhibitor
18	BRD-A16820783	phosphodiesterase inhibitor
19	vincristine	tubulin inhibitor, microtubule inhibitor, microtubule polymerization inhibitor, tubulin polymerisation inhibitor, vinca alkaloid
20	LDN-193189	ALK inhibitor, serine/threonine protein kinase inhibitor
21	podophyllotoxin	caspase activator, insulin growth factor receptor inhibitor, microtubule inhibitor, tubulin inhibitor
22	ingenol	PKC activator
23	NSC-632839	ubiquitin hydrolase inhibitor, ubiquitin isopeptidase inhibitor
24	etoposide	topoisomerase inhibitor, cell cycle inhibitor, DNA inhibitor, DNA intercalating drug, DNA repair enzyme inhibitor
25	MLN-4924	nedd activating enzyme inhibitor
26	thapsigargin	ATPase inhibitor, calcium channel blocker, PKC activator
27	z-leu3-VS	PGPH inhibitor, proteasome inhibitor
28	amsacrine	topoisomerase inhibitor, DNA intercalating drug
29	TW-37	BCL inhibitor, MCL1 inhibitor
30	KF-38789	P selectin inhibitor
31	pyrvinium-pamoate	AKT inhibitor, androgen receptor (AR) inhibitor, androgen receptor antagonist
32	NSC-3852	HDAC inhibitor
33	SA-792728	sphingosine kinase inhibitor, ATPase inhibitor
34	LY-2183240	FAAH inhibitor, FAAH reuptake inhibitor
35	cercosporin	photoactivated to produce toxic reactive oxygen species, PKC inhibitor
36	BCI-hydrochloride	MAP kinase phosphatase inhibitor, protein phosphatase inhibitor
37	SA-1478088	metalloproteinase inhibitor
38	BMY-45778	IP1 prostacyclin receptor agonist
39	nocodazole	tubulin inhibitor, Tubulin Polymerization Inhibitors
40	chloroxine	opioid receptor inhibitor
41	MLN-2238	proteasome inhibitor
42	triciribine	AKT inhibitor
43	apicidin	HDAC inhibitor
44	amonafide	topoisomerase inhibitor, DNA intercalating drug
45	JTC-801	opioid receptor antagonist
46	NSC-632839	ubiquitin hydrolase inhibitor, ubiquitin isopeptidase inhibitor
47	IKK-2-inhibitor-V	IKK inhibitor, NFkB pathway inhibitor, protein kinase inhibitor
48	AG-592	tyrosine kinase inhibitor
49	BNTX	opioid receptor antagonist
50	manumycin-a	farnesyltransferase inhibitor, NFkB pathway inhibitor, ras farnesyltransferase inhibitor
51	mebendazole	acetylcholinesterase inhibitor, microtubule inhibitor, tubulin inhibitor
52	SCH-79797	proteinase activated receptor antagonist
53	kinetin-riboside	apoptosis inducer
54	cladribine	adenosine deaminase inhibitor, antimetabolite, DNA synthesis inhibitor, immunosuppressant, ribonucleotide reductase inhibitor
55	cucurbitacin-i	inhibitor of STAT3/JAK2 signaling, JAK inhbitor, lipocortin synthesis stimulant, STAT inhibitor
56	idarubicin	topoisomerase inhibitor, radical formation stimulant, RNA synthesis inhibitor
57	teniposide	topoisomerase inhibitor, DNA inhibitor, DNA repair enzyme inhibitor, mitotic inhibitor
58	malonoben	protein tyrosine kinase inhibitor, tyrosine kinase inhibitor
59	BIX-01294	DNA methyltransferase inhibitor, histone lyinse methyltransferase inhibitor, histone lysine methyltransferase inhibitor
60	RO-28-1675	glucokinase activator
61	YM-155	survivin inhibitor, XIAP expression inhibitor
62	manumycin-a	farnesyltransferase inhibitor, NFkB pathway inhibitor, ras farnesyltransferase inhibitor
63	vinorelbine	tubulin inhibitor, apoptosis stimulant, microtubule inhibitor, mitosis inhibitor, mitotic inhibitor, tubulin polymerisation inhibitor, vinca alkaloid

64	CGP-71683	neuropeptide receptor antagonist
65	rhodomyrtoxin-b	cytotoxic, DNA intercalator
66	parthenolide	NFkB pathway inhibitor, adiponectin receptor agonist
67	cucurbitacin-i	inhibitor of STAT3/JAK2 signaling, JAK inhbitor, lipocortin synthesis stimulant, STAT inhibitor
68	obatoclax	BCL inhibitor, MCL1 inhibitor
69	ryuvidine	histone lyinse methyltransferase inhibitor
70	SA-63133	casein kinase inhibitor, tubulin inhibitor
71	perhexiline	carnitine palmitoyltransferase binder, carnitine palmitoyltransferase inhibitor
72	rottlerin	large conductance potassium channel activator, MAP kinase inhibitor, PKC inhibitor, protein kinase inhibitor, tissue transglutaminase inhibitor
73	calyculin	protein phosphatase inhibitor
74	SA-792574	microtubule inhibitor, tubulin inhibitor
75	puromycin	adenosine receptor agonist, protein synthesis inhibitor
76	methylene-blue	guanylate cyclase inhibitor, monoamine oxidase inhibitor, nitric oxide production inhibitor, tau aggregation inhibitor
77	radicicol	HSP inhibitor, ATP citrase lyase inhibitor, MAP kinase inhibitor, opioid receptor ligand, pyruvate dehydrogenase kinase inhibitor
78	CGK-733	ATM kinase inhibitor, ATR kinase inhibitor
79	VU-0365114-2	acetylcholine receptor allosteric modulator, M5 modulator
80	AG-879	angiogenesis inhibitor, EGFR inhibitor, tyrosine kinase inhibitor, VEGFR inhibitor
81	vinblastine	microtubule formation inhibitor, tubulin inhibitor, tubulin polymerisation inhibitor
82	BVT-948	tyrosine phosphatase inhibitor
83	azacitidine	DNA methyltransferase inhibitor, antimetabolite, DNA methylase inhibitor, DNA synthesis inhibitor, RNA synthesis inhibitor
84	puromycin	adenosine receptor agonist, protein synthesis inhibitor
85	vinorelbine	tubulin inhibitor, apoptosis stimulant, microtubule inhibitor, mitosis inhibitor, mitotic inhibitor, tubulin polymerisation inhibitor, vinca alkaloid
86	15-delta-prostaglandin-j2	PPAR receptor agonist, FXR antagonist
87	SSR-69071	leukocyte elastase inhibitor
88	BRD-K98824517	tyrosine phosphatase inhibitor
89	anisomycin	DNA synthesis inhibitor
90	penfluridol	dopamine receptor antagonist, T-type calcium channel blocker
91	JAK3-inhibitor-VI	JAK inhibitor
92	niclosamide	DNA replication inhibitor, STAT inhibitor
93	ABT-751	tubulin inhibitor, dihydropteroate synthase inhibitor, microtubule inhibitor, PABA antagonist, tubulin polymerisation inhibitor
94	BRD-K73610817	apoptosis protein inhibitor
95	auranofin	disease modifying antirheumatic drug, immunosuppressant, NFkB pathway inhibitor, prostanoid receptor inhibitor, thioredoxin reductase inhibitor
96	ZK-164015	estrogen receptor antagonist
97	cephaeline	protein synthesis inhibitor
98	7b-cis	exportin antagonist
99	alvespimycin	HSP inhibitor, HSP antagonist
100	FCCP	mitochondrial oxidative phosphorylation uncoupler
101	cyclosporin-a	calcineurin inhibitor, cyclophilin inhibitor, immunosuppressant, insulin expression inhibitor, T cell inhibitor
102	GW-405833	cannabinoid receptor agonist
103	SA-792709	retinoid receptor agonist
104	mitomycin-c	DNA alkylating drug, DNA inhibitor, DNA synthesis inhibitor
105	gossypol	BCL inhibitor, MCL1 inhibitor, 11-beta hydroxysteroid dehydrogenase inhibitor, growth factor receptor modulator, lipid peroxidase inhibitor
106	panobinostat	HDAC inhibitor, apoptosis stimulant, cell cycle inhibitor
107	diphenyleneiodonium	nitric oxide synthase inhibitor, aldehyde dehydrogenase inhibitor, cytochrome P450 inhibitor, NADPH oxidase inhibitor, xanthine oxidase inhibitor
108	emetine	protein synthesis inhibitor
109	menadione	CDC inhibitor, mitochondrial DNA polymerase inhibitor, phosphatase inhibitor, pyruvate kinase isozyme inhibitor
110	homoharringtonine	apoptosis stimulant, protein synthesis inhibitor
111	irinotecan	topoisomerase inhibitor
112	nonoxynol-9	interacts with the lipids in the membranes of the acrosome and the midpiece of the sperm
113	heliomycin	antibiotic, bacterial RNA synthesis inhibitor
114	selamectin	nematocide
115	tunicamycin	GLCNAC phosphotransferase inhibitor
116	bithionol	autotaxin inhibitor
117	AG-957	Abl kinase inhibitor, protein tyrosine kinase inhibitor
118	oxibendazole	DNA polymerase inhibitor, tubulin inhibitor
119	rottlerin	large conductance potassium channel activator, MAP kinase inhibitor, PKC inhibitor, protein kinase inhibitor, tissue transglutaminase inhibitor
120	thiostrepton	downregulates FOXM1 expression, FOXM1 expression inhibitor, protein synthesis inhibitor
121	HU-211	glutamate receptor antagonist, apoptosis stimulant, NFkB pathway inhibitor, reducing agent
122	narciclasine	coflilin signaling pathway activator, LIM kinase activator, ROCK activator
123	doxorubicin	topoisomerase inhibitor, DNA intercalating drug
124	verrucarin-a	protein synthesis inhibitor
125	lasalocid	ionophore antibiotic
126	cytochalasin-d	actin polymerization inhibitor, actin stabilizer
127	tyrphostin-A9	protein tyrosine kinase inhibitor, tyrosine kinase inhibitor
128	tegaserod	serotonin receptor partial agonist, serotonin receptor agonist
129	diphencyprone	immunostimulant
130	withaferin-a	acetylcholinesterase inhibitor, butyrylcholinesterase inhibitors, IKK inhibitor, NFkB pathway inhibitor, PKC inhibitor
131	suloctidil	adrenergic receptor antagonist, platelet aggregation inhibitor, vasodilator
132	PHA-665752	c-Met inhibitor, hepatocyte growth factor receptor inhibitor
133	alisertib	Aurora kinase inhibitor, mitotic inhibitor, protein kinase inhibitor
134	capsazepine	TRPV agonist, TRPV antagonist
135	vincristine	tubulin inhibitor, microtubule inhibitor, microtubule polymerization inhibitor, tubulin polymerisation inhibitor, vinca alkaloid
136	DL-PDMP	ceramide glucosyltransferase inhibitor, glucosyltransferase inhibitor
137	pyrrolidine-dithiocarbamate	NFkB pathway inhibitor
138	dorsomorphin	AMPK inhibitor, DNA damaging, TGF beta receptor inhibitor
139	digoxin	ATPase inhibitor, ROR antagonist
140	bufalin	ATPase inhibitor, chloride channel activator

141	purvalanol-a	CDK inhibitor, dual specificity tyrosine-(Y)-phosphorylation regulated kinase inhibitor
142	ivermectin	GABA receptor agonist, acetylcholine receptor allosteric modulator, GABA release stimulant, purinergic receptor allosteric modulator
143	cycloheximide	glycogen synthase kinase inhibitor, protein synthesis inhibitor
144	calmidazolium	calcium channel blocker, calmodulin antagonist
145	JLK-6	gamma secretase inhibitor, antiamyloidogenic agent
146	vindesine	tubulin inhibitor, microtubule inhibitor, vinca alkaloid
147	oligomycin-a	ATP synthase inhibitor, ATPase inhibitor
148	KI-8751	PDGFR alpha and c-Kit inhibitor, vascular endothelial growth factor receptor 2 (VEGFR2) inhibitor, VEGFR inhibitor
149	gemcitabine	cell cycle inhibitor, DNA repair enzyme inhibitor, DNA synthesis inhibitor, pyrimidine antagonist, ribonucleoside reductase inhibitor, ribonucleotide reductase inhibitor
150	SB-415286	glycogen synthase kinase inhibitor
151	importazole	importin-beta transport receptor inhibitor
152	NNC-55-0396	T-type calcium channel blocker
153	ouabain	ATPase inhibitor, alpha subunit binder
154	brefeldin-a	antibiotic that disrupts Golgi function, brefeldin A inhibited guanine nucleotide exchange protein inhibitors, golgi-specific brefeldin A-resistance guanine nucleotide exchange factor inhibitor, protein synthesis inhibitor
155	lylamine	cannabinoid receptor agonist
156	niguldipine	adrenergic receptor antagonist, calcium channel blocker, calcium channel antagonist, calcium channel inhibitor
157	AKT-inhibitor-IV	AKT inhibitor
158	MST-312	telomerase inhibitor
159	PAC-1	caspase activator
160	IKK-16	IKK inhibitor
161	SA-792987	PKC inhibitor
162	SN-38	topoisomerase inhibitor
163	emetine	protein synthesis inhibitor
164	homoharringtonine	apoptosis stimulant, protein synthesis inhibitor
165	disulfiram	aldehyde dehydrogenase inhibitor, DNA methyltransferase inhibitor, TRPA1 agonist
166	SB-218078	CHK inhibitor, PKC inhibitor
167	digitoxin	ATPase inhibitor
168	cycloheximide	glycogen synthase kinase inhibitor, protein synthesis inhibitor
169	WR-216174	CDK inhibitor, PFMRK inhibitor
170	CCCP	mitochondrial oxidative phosphorylation uncoupler
171	cyclosporin-a	calcineurin inhibitor, cyclophilin inhibitor, immunosuppressant, insulin expression inhibitor, T cell inhibitor
172	parthenolide	NFkB pathway inhibitor, adiponectin receptor agonist
173	sorafenib	RAF inhibitor, FLT3 inhibitor, KIT inhibitor, PDGFR tyrosine kinase receptor inhibitor, RET tyrosine kinase inhibitor, VEGFR inhibitor, angiogenesis inhibitor, VEGFR antagonist
174	cymarin	ATPase inhibitor
175	BRD-A54632525	lipoxygenase inhibitor
176	vinorelbine	tubulin inhibitor, apoptosis stimulant, microtubule inhibitor, mitosis inhibitor, mitotic inhibitor, tubulin polymerisation inhibitor, vinca alkaloid
177	butein	angiotensin converting enzyme inhibitor, epidermal growth factor receptor (EGFR) inhibitor, interleukin synthesis inhibitor, NFkB pathway inhibitor, SIRT activator, Src tyrosine kinase inhibitor, steroid 5alpha-reductase inhibitor,
178	piperlongumine	glutathione transferase inhibitor
179	avrainvillamide-analog-2	nucleophosmin inhibitor
180	brazilin	nitric oxide production inhibitor, nitric oxide synthase expression inhibitor, telomerase inhibitor
181	GR-127935	serotonin receptor antagonist
182	JW-7-24-1	LCK Inhibitor
183	MAZ-51	VEGFR inhibitor
184	aminopurvalanol-a	CDK inhibitor, tyrosine kinase inhibitor
185	digoxin	ATPase inhibitor, ROR antagonist
186	WT-171	HDAC inhibitor
187	topotecan	topoisomerase inhibitor
188	CAY-10618	NAMPT inhibitor, niacinamide phosphoribosyltransferase inhibitor
189	tyrphostin-AG-835	protein tyrosine kinase inhibitor
190	PHA-793887	CDK inhibitor
191	niguldipine	adrenergic receptor antagonist, calcium channel blocker, calcium channel antagonist, calcium channel inhibitor
192	neratinib	EGFR inhibitor, receptor tyrosine protein kinase inhibitor, tyrosine kinase inhibitor
193	phloretin	VCAM expression inhibitor, ICAM1 expression inhibitor
194	terfenadine	histamine receptor antagonist
195	bisindolylmaleimide	CDK inhibitor, PKC inhibitor, leucine rich repeat kinase inhibitor
196	tyrphostin-AG-1478	EGFR inhibitor
197	topotecan	topoisomerase inhibitor
198	flavokavain-b	carcinoma cell growth inhibitor, hypoxia inducible factor inhibitor
199	SID-26681509	cathepsin inhibitor
200	methotrexate	dihydrofolate reductase inhibitor, disease modifying antirheumatic drug, folate receptor antagonist, immunosuppressant, thymidylate synthase inhibitor
201	serdemetan	MDM inhibitor, angiogenesis stimulant, apoptosis stimulant, oncogene inhibitor
202	sappanone-a	melanogenesis inhibitor, tyrosinase inhibitor
203	arachidonyl-trifluoro-methane	cytosolic phospholipase inhibitor
204	EI-346-erlotinib-analog	epidermal growth factor receptor (EGFR) inhibitor
205	deguelin	NADH-ubiquinone oxidoreductase (Complex I) inhibitor, AKT inhibitor, cyclooxygenase inhibitor, NFkB pathway inhibitor, phorbol ester-induced ornithine decarboxylase (ODC) activity suppressor, PI3K inhibitor
206	isoliquiritigenin	guanylate cyclase activator, aldose reductase inhibitor, glutamate receptor antagonist, histamine receptor antagonist, SIRT activator
207	calmidazolium	calcium channel blocker, calmodulin antagonist
208	hinokitiol	tyrosinase inhibitor
209	BAY-11-7821	NFkB pathway inhibitor
210	RS-17053	adrenergic receptor antagonist
211	BRD-K37940862	neuropeptide receptor antagonist
212	THM-I-94	HDAC inhibitor, apoptosis stimulant, cell cycle inhibitor
213	vorinostat	HDAC inhibitor, cell cycle inhibitor
214	fenretinide	apoptosis stimulant, retinoid receptor agonist, RAR agonist
215	wortmannin	PI3K inhibitor, ATM kinase inhibitor, ATR kinase inhibitor, DNA dependent protein kinase inhibitor, mTOR inhibitor, Phosphatidylinositol 3-kinase (PI3K) inhibitor, PLK inhibitor
216	floxuridine	DNA synthesis inhibitor, MDCK inhibitor, PEPT1 inhibitor, pyrimidine antagonist
217	caffeic-acid	lipoxygenase inhibitor, HIV integrase inhibitor, NFkB pathway inhibitor, nitric oxide production inhibitor, PPAR receptor modulator, tumor necrosis factor production inhibitor

217	caffeic-acid	lipoxygenase inhibitor, HIV integrase inhibitor, NFKB pathway inhibitor, nitric oxide production inhibitor, PPAR receptor modulator, tumor necrosis factor production inhibitor
218	devazepide	CCK receptor antagonist, gastrin inhibitor
219	helveticoside	ATPase inhibitor
220	BX-912	AKT inhibitor, phosphoinositide dependent kinase inhibitor, pyruvate dehydrogenase kinase inhibitor
221	floxuridine	DNA synthesis inhibitor, MDCK inhibitor, PEPT1 inhibitor, pyrimidine antagonist
222	ouabain	ATPase inhibitor, alpha subunit binder
223	nitazoxanide	pyruvate ferredoxin oxidoreductase inhibitor, pyruvate synthase inhibitor
224	CAY-10470	NFKB pathway inhibitor
225	camptothecin	topoisomerase inhibitor, beta amyloid synthesis inhibitor, hypoxia inducible factor inhibitor
226	indirubin	CDK inhibitor, glycogen synthase kinase inhibitor, cyclin-dependent kinase inhibitor, PKC inhibitor
227	digoxin	ATPase inhibitor, ROR antagonist
228	QL-XII-47	BMX inhibitor, Bruton's tyrosine kinase (BTK) inhibitor, bruton's tyrosine kinase inhibitor, cytoplasmic tyrosine protein kinase BMX inhibitor
229	trichostatin-a	HDAC inhibitor, CDK expression enhancer, ID1 expression inhibitor
230	triclosan	DNA methyltransferase inhibitor, enoyl-(acyl-carrier protein) reductase FabI inhibitor, enoyl-[acyl-carrier-protein] reductase [NADH] inhibitor
231	ON-01910	PLK inhibitor, cell cycle inhibitor, MCL1 inhibitor, protein kinase inhibitor
232	HC-toxin	HDAC inhibitor
233	chelidonine	tubulin polymerization inhibitor
234	emetine	protein synthesis inhibitor
235	FIT	opioid receptor agonist
236	SB-225002	CC chemokine receptor antagonist, Chemokine CXCR2 (IL-8 beta Receptor) Antagonists
237	cytarabine	antimetabolite, DNA polymerase inhibitor, DNA synthesis inhibitor, ribonucleotide reductase inhibitor
238	BAPTA-AM	potassium channel blocker
239	RO-08-2750	nerve growth factor receptor ligand, NGF binding inhibitor
240	GSK-3-inhibitor-IX	glycogen synthase kinase inhibitor, lipoxygenase inhibitor, PKC inhibitor
241	SU-11652	FGFR inhibitor, PDGFR tyrosine kinase receptor inhibitor, tyrosine kinase receptor & angiogenic inhibitor, VEGFR inhibitor
242	cyclosporin-a	calcineurin inhibitor, cyclophilin inhibitor, immunosuppressant, insulin expression inhibitor, T cell inhibitor
243	tyrphostin-AG-126	ERK1 and ERK2 phosphorylation inhibitor, tyrosine kinase inhibitor
244	digitoxigenin	ATPase inhibitor
245	proscillaridin	ATPase inhibitor
246	sorafenib	RAF inhibitor, FLT3 inhibitor, KIT inhibitor, PDGFR tyrosine kinase receptor inhibitor, RET tyrosine kinase inhibitor, VEGFR inhibitor, angiogenesis inhibitor, VEGFR antagonist
247	flubendazole	acetylcholinesterase inhibitor, microtubule inhibitor, tubulin inhibitor
248	carmofur	thymidylate synthase inhibitor, pyrimidine antagonist
249	hyperforin	cyclooxygenase inhibitor, dopamine reuptake inhibitor, interleukin receptor antagonist, lipoxygenase inhibitor, serotonin uptake inhibitor
250	lasalocid	ionophore antibiotic
251	PAC-1	caspase activator
252	trichostatin-a	HDAC inhibitor, CDK expression enhancer, ID1 expression inhibitor
253	L-168049	glucagon receptor antagonist, human glucagon receptor antagonist
254	indolophenanthridine	CALY activator, dopamine receptor agonist
255	artesunate	DNA synthesis inhibitor
256	strophanthidin	ATPase inhibitor
257	piceatannol	syk inhibitor, cyclooxygenase inhibitor, nitric oxide synthase inhibitor, SIRT activator, tyrosinase inhibitor, tyrosine kinase inhibitor
258	CHEMBL-374350	NFkB pathway inhibitor
259	everolimus	mTOR inhibitor, angiogenesis inhibitor, cell cycle inhibitor, immunosuppressant, protein kinase inhibitor, rotamase inhibitor
260	ouabain	ATPase inhibitor, alpha subunit binder
261	LE-135	retinoid receptor agonist
262	roscovitine	CDK inhibitor, apoptosis stimulant
263	5-nonyloxytryptamine	serotonin receptor agonist
264	mibefradil	T-type calcium channel blocker, angiogenesis inhibitor, calcium channel blocker, calcium channel inhibitor, L-type calcium channel blocker, sodium channel blocker
265	quercetin	aldose reductase inhibitor, cytokine production inhibitor, EGFR inhibitor, glucosidase inhibitor, monoamine oxidase inhibitor, polar auxin transport inhibitor, quorum sensing signaling modulator, SIRT activator, sodium channel bl
266	T-98475	gonadotropin releasing factor hormone receptor antagonist
267	WAY-170523	metalloproteinase inhibitor
268	XL-147	Phosphatidylinositol 3-kinase (PI3K) inhibitor, PI3K inhibitor
269	belinostat	HDAC inhibitor, cell cycle inhibitor
270	tamoxifen	estrogen receptor antagonist, selective estrogen receptor modulator (SERM), estrogen receptor agonist, estrogen receptor modulator, PKC inhibitor
271	BAX-channel-blocker	cytochrome C release inhibitor
272	L-690488	nositol monophosphatase inhibitor
273	torin-2	mTOR inhibitor
274	MBCQ	phosphodiesterase inhibitor
275	penicillic-acid	bacterial quorum sensing inhibitor, caspase inhibitor
276	cyclopiazonic-acid	ATPase inhibitor
277	JNJ-7706621	CDK inhibitor, Aurora kinase inhibitor
278	BRD-K77681376	casein kinase inhibitor, FLT3 inhibitor
279	ISOX	HDAC inhibitor
280	NNC-05-2090	GABA uptake inhibitor, GAT inhibitor
281	tetrindole	monoamine oxidase inhibitor
282	nitrofural	POXB inhibitor
283	CA-074-Me	cathepsin inhibitor, antiamyloidogenic agent
284	cinobufagin	ATPase inhibitor, chloride channel activator
285	Y-134	estrogen receptor antagonist, selective estrogen receptor modulator (SERM)
286	BI-2536	PLK inhibitor, apoptosis stimulant, cell cycle inhibitor, protein kinase inhibitor
287	rhodomyrtoxin	cytotoxic, DNA intercalator
288	reserpine	vesicular monoamine transporter inhibitor
289	fenbendazole	cytochrome P450 inhibitor, tubulin inhibitor
290	KU-0060648	DNA dependent protein kinase, DNA dependent protein kinase inhibitor, PI3K inhibitor
291	BX-795	IKK inhibitor, PDK1 inhibitor, phosphoinositide dependent kinase inhibitor, serine/threonine kinase inhibitor, TBK1 inhibitor
292	KU-C103428N	CDC inhibitor, rho GTPase inhibitor
293	dacinostat	HDAC inhibitor
294	oligomycin-a	ATP synthase inhibitor, regulatory volume decrease inhibitor

294	oligomycin-c	ATP synthase inhibitor, regulatory volume decrease inhibitor
295	BIBU-1361	EGFR inhibitor
296	16,16-dimethylprostaglandin-e2	15-hydroxyprostaglandin dehydrogenase inhibitor, unidentified pharmacological activity
297	erbstatin-analog	EGFR inhibitor, tyrosine kinase inhibitor
298	kenpaullone	CDK inhibitor, glycogen synthase kinase inhibitor, src inhibitor
299	BRD-K53780220	casein kinase inhibitor, FLT3 inhibitor
300	PD-198306	MAP kinase inhibitor, MEK inhibitor
301	thiothixene	dopamine receptor, dopamine receptor antagonist
302	droxinostat	HDAC inhibitor
303	thiazolopyrimidine	CDC inhibitor
304	ispinesib	kinesin family member inhibitor, kinesin inhibitor, Kinesin-Like Spindle Protein KIF11 (KSP, Eg5) Inhibitors
305	AT-9283	Aurora kinase inhibitor, JAK inhibitor, Abl kinase inhibitor, Bcr-Abl kinase inhibitor, FLT3 inhibitor, mitotic inhibitor, protein kinase inhibitor
306	sertraline	serotonin receptor antagonist, serotonin reuptake inhibitor, serotonin uptake inhibitor
307	tyrphostin-AG-527	protein tyrosine kinase inhibitor
308	albendazole	tubulin inhibitor, acetylcholinesterase inhibitor, microtubule inhibitor
309	NCH-51	HDAC inhibitor
310	simvastatin	HMGCR inhibitor
311	oxindole-I	protein kinase inhibitor, vascular endothelial growth factor receptor (VEGFR) inhibitor
312	mammea-a	cytotoxic, antibacterial
313	lypressin	vasopressin receptor agonist
314	oxymetholone	androgen receptor agonist, synthetic hormone with anabolic and androgenic properties
315	sulconazole	sterol demethylase inhibitor, cell wall synthesis inhibitor
316	nortriptyline	PI3K inhibitor, potassium channel blocker, tricyclic antidepressant (TCA)
317	pifithrin-mu	HSP inhibitor
318	givinostat	HDAC inhibitor, interleukin receptor antagonist, interleukin synthesis inhibitor, tumor necrosis factor receptor antagonist, tumor necrosis factor release inhibitor
319	CP-55940	cannabinoid receptor agonist, CC chemokine receptor antagonist
320	pimozide	dopamine receptor antagonist, dopamine receptor, opioid receptor antagonist, serotonin receptor antagonist
321	latrunculin-b	actin destabilizer, unidentified pharmacological activity
322	masitinib	KIT inhibitor, PDGFR tyrosine kinase receptor inhibitor, src inhibitor, c-kit inhibitor, FGFR antagonist, FGFR inhibitor, tyrosine kinase inhibitor
323	SA-792541	caspase activator, CDC inhibitor
324	4-hydroxy-2-nonenal	cytotoxic lipid peroxidation product
325	periplocymarin	apoptosis inducer
326	CGP-53353	EGFR inhibitor, PKC inhibitor
327	mirin	inhibitor of MRE11A exonuclease activity
328	scriptaid	HDAC inhibitor
329	WZ-4-145	EGFR inhibitor
330	BCL2-inhibitor	BCL inhibitor
331	pyroxamide	HDAC inhibitor, cell cycle inhibitor
332	wortmannin	PI3K inhibitor, ATM kinase inhibitor, ATR kinase inhibitor, DNA dependent protein kinase inhibitor, mTOR inhibitor, Phosphatidylinositol 3-kinase (PI3K) inhibitor, PLK inhibitor
333	cerivastatin	HMGCR inhibitor
334	WYE-125132	mTOR inhibitor, PI3K inhibitor
335	15-delta-prostaglandin-j2	PPAR receptor agonist, FXR antagonist
336	wortmannin	PI3K inhibitor, ATM kinase inhibitor, ATR kinase inhibitor, DNA dependent protein kinase inhibitor, mTOR inhibitor, Phosphatidylinositol 3-kinase (PI3K) inhibitor, PLK inhibitor
337	nisoldipine	calcium channel blocker, L-type calcium channel blocker
338	guggulsterone	estrogen receptor agonist, FXR antagonist, progesterone receptor agonist, cholesterol inhibitor, IKK inhibitor, PXR agonist
339	triciribine	AKT inhibitor
340	crizotinib	ALK tyrosine kinase receptor inhibitor, c-Met inhibitor, hepatocyte growth factor receptor inhibitor, tyrosine kinase inhibitor
341	H-89	AKT inhibitor, cAMP dependent protein kinase inhibitor, PKA inhibitor, voltage-gated potassium channel blocker
342	BMS-345541	IKK inhibitor
343	Merck60	HDAC inhibitor
344	iodoacetic-acid	cysteine peptidase inhibitor
345	GW-843682X	PLK inhibitor
346	TER-14687	inhibitor of translocation of PKCq in T cells
347	phenamil	acid sensing ion channel blocker, Sodium Channel Blockers, TRPP3 channel inhibitor, TRPV modulator
348	elesclomol	apoptosis stimulant, HSP agonist, HSP inducer, oxidative stress inducer, topoisomerase inhibitor
349	KN-93	calcium/calmodulin dependent protein kinase inhibitor
350	berbamine	calmodulin antagonist
351	staurosporine	PKC inhibitor, AKT inhibitor, BMX inhibitor, CDK inhibitor, CHK inhibitor, G protein coupled receptor agonist, glycogen synthase kinase inhibitor, leucine rich repeat kinase inhibitor, ribosomal protein inhibitor, sodium/hydrogen e
352	calcipotriol	vitamin D receptor agonist
353	AR-A014418	glycogen synthase kinase inhibitor
354	cytochalasin-b	microtubule inhibitor, phagocytosis inhibitor
355	sarmentogenin	ATPase inhibitor
356	NTNCB	neuropeptide receptor antagonist
357	VU-0404997-2	glutamate receptor modulator
358	PD-102807	acetylcholine receptor antagonist
359	sirolimus	mTOR inhibitor, CCR expression inhibitor, cell cycle inhibitor, proteasome inhibitor, protein kinase inhibitor, T cell inhibitor
360	tyrphostin-AG-556	epidermal growth factor receptor (EGFR) inhibitor, tyrosine kinase inhibitor
361	methyl-2,5-dihydroxycinnamate	EGFR inhibitor, tyrosine kinase inhibitor
362	isorotenone	mitochondrial complex I inhibitor, NADH-ubiquinone oxidoreductase (Complex I) inhibitor
363	exemestane	aromatase inhibitor
364	PF-562271	focal adhesion kinase inhibitor, angiogenesis inhibitor, apoptosis stimulant
365	phenoxybenzamine	adrenergic receptor antagonist
366	JNK-9L	JNK inhibitor
367	mitoxantrone	topoisomerase inhibitor, DNA intercalating drug, HCV inhibitor, immunosuppressant, Pim kinase inhibitor
368	BRD-K61463582	aryl hydrocarbon receptor agonist
369	JNJ-26854165	HDAC inhibitor, MDM inhibitor
370	NVP-TAE684	ALK tyrosine kinase receptor inhibitor, ALK tyrosine kinase receptor mutant inhibitor, leucine rich repeat kinase inhibitor

371	benperidol	dopamine receptor antagonist
372	fludarabine	DNA synthesis inhibitor, DNA repair enzyme inhibitor, purine antagonist, ribonucleotide reductase inhibitor
373	cyproheptadine	histamine receptor antagonist, serotonin receptor antagonist
374	KU-C103443N	CDC inhibitor, rho GTPase inhibitor
375	ZG-10	JNK inhibitor
376	SU-11274	hepatocyte growth factor receptor inhibitor, tyrosine kinase inhibitor
377	westcort	corticosteroid
378	Ala-Ala-Phe-CMK	tripeptidyl peptidase inhibitor
379	afatinib	EGFR inhibitor, receptor tyrosine protein kinase inhibitor, tyrosine kinase inhibitor
380	AZD-8055	mTOR inhibitor
381	xanthohumol	aromatase inhibitor, diacylglycerol O acyltransferase inhibitor, valosin containing protein inhibitor
382	etacrynic-acid	not applicable, sodium/potassium/chloride transporter inhibitor
383	GANT-61	GLI antagonist
384	sunitinib	FLT3 inhibitor, KIT inhibitor, PDGFR tyrosine kinase receptor inhibitor, RET tyrosine kinase inhibitor, VEGFR inhibitor, angiogenesis inhibitor, colony stimulating factor receptor antagonist, colony stimulating factor receptor inhib
385	docetaxel	tubulin inhibitor, microtubule depolymerization inhibitor, microtubule stabilizing agent, microtubule stimulant, taxane
386	ZK-93423	benzodiazepine receptor agonist
387	megestrol	progesterone receptor agonist, DNA inhibitor, HegG2 inhibitor
388	SU-1498	receptor tyrosine protein kinase inhibitor, vascular endothelial growth factor receptor 2 (VEGFR2) inhibitor, VEGFR inhibitor
389	MT-21	adenine nucleotide translocase inhibitor, caspase activator, neurotrophic agent
390	securinine	GABA receptor antagonist, TP53 activator
391	indatraline	norepinephrine transporter inhibitor, dopamine transporter inhibitor, dopamine uptake inhibitor, serotonin transporter (SERT) inhibitor, serotonin uptake inhibitor
392	canertinib	EGFR inhibitor, EGFR antagonist, receptor tyrosine protein kinase inhibitor, tyrosine kinase inhibitor
393	salubrinal	eukaryotic translation initiation factor inhibitor, GlyT-1 inhibitor
394	bisindolylmaleimide-ix	PKC inhibitor, glycogen synthase kinase inhibitor, leucine rich repeat kinase inhibitor, SIRT inhibitor
395	doconexent	PPAR receptor agonist, unidentified pharmacological activity
396	amlodipine	breast cancer resistance protein inhibitor, calcium channel blocker, calcium channel inhibitor, L-type calcium channel blocker
397	exemestane	aromatase inhibitor
398	orlistat	cholesterol inhibitor, diacylglycerol lipase inhibitor, fatty acid synthase inhibitor, gastric triacylglycerol lipase inhibitor, Hypolipemic pancreatic, gastric and carboxylester lipase inhibitor, lipase inhibitor, pancreatic lipase inhibitor
399	ZM-39923	JAK inhibitor
400	mycophenolate-mofetil	dehydrogenase inhibitor, hydroxycarboxylic acid receptor agonist, immunosuppressant, IMPDH inhibitor, inosine monophosphate dehydrogenase inhibitor
401	TG-101348	JAK inhibitor, FLT3 inhibitor, RET tyrosine kinase inhibitor
402	tyrphostin-AG-555	CDK inhibitor, epidermal growth factor receptor (EGFR) inhibitor, tyrosine kinase inhibitor
403	embelin	HCV inhibitor, XIAP inhibitor
404	raltitrexed	thymidylate synthase inhibitor, pyrimidine antagonist
405	BRD-K30351863	APEX inhibitor
406	PQ-401	insulin growth factor receptor inhibitor
407	triptolide	RNA polymerase inhibitor
408	fenretinide	apoptosis stimulant, retinoid receptor agonist, RAR agonist
409	CGP-60474	CDK inhibitor, PKC inhibitor
410	celastrol	anti-inflammatory agent, antioxidant, HSP90 Inhibitor, NFKB pathway inhibitor, topoisomerase inhibitor
411	entinostat	HDAC inhibitor, cell cycle inhibitor
412	ABT-737	BCL inhibitor
413	1-phenylbiguanide	serotonin receptor agonist
414	AT-7519	CDK inhibitor, cell cycle inhibitor
415	AG-494	epidermal growth factor receptor (EGFR) inhibitor, tyrosine kinase inhibitor
416	aripiprazole	serotonin receptor agonist, serotonin receptor antagonist, dopamine receptor agonist, dopamine receptor partial agonist, serotonin receptor partial agonist
417	mefloquine	acidifying agent non gastric, adenosine receptor antagonist, calmodulin antagonist, hemoglobin antagonist, pannexin inhibitor
418	idebenone	calcium channel modulator, reducing agent
419	CGS-15943	adenosine receptor antagonist
420	RS-39604	serotonin receptor antagonist
421	XMD-1150	leucine rich repeat kinase inhibitor
422	clofazimine	GK0582 inhibitor
423	tyrphostin-AG-18	epidermal growth factor receptor (EGFR) inhibitor, tyrosine kinase inhibitor
424	mycophenolic-acid	dehydrogenase inhibitor, immunosuppressant, IMPDH inhibitor, inosine monophosphate dehydrogenase inhibitor
425	bisbenzimide	BCL inhibitor, DNA binding, topoisomerase inhibitor
426	BRD-K48974000	CDC inhibitor
427	dinoprostone	prostanoid receptor agonist
428	antimycin-a	antibiotic, electron transfer inhibitor
429	pyrimethamine	dihydrofolate reductase inhibitor, hexosaminidase stimulant, pharmacological chaperone, STAT inhibitor
430	aloisine	CDK inhibitor, CFTR channel activator, glycogen synthase kinase inhibitor, JNK inhibitor
431	rosiglitazone	PPAR receptor agonist, insulin sensitizer, thiazolidinedione
432	AM-281	cannabinoid receptor antagonist, cannabinoid receptor inhibitor
433	calcifediol	vitamin D receptor agonist
434	BRD-K06956503	glucosylceramidase inhibitor
435	GSK-1059615	PI3K inhibitor, mTOR inhibitor
436	bromocriptine	dopamine receptor agonist, dopamine receptor antagonist, prolactin secretion inhibitor
437	BRD-K91781484	nuclear factor erythroid derived, like (NRF2) activator
438	alvocidib	CDK inhibitor, apoptosis stimulant, BCL inhibitor, cell cycle inhibitor, MCL1 inhibitor, survivin inhibitor, XIAP inhibitor
439	ENMD-2076	Aurora kinase inhibitor, FLT3 inhibitor, VEGFR inhibitor, angiogenesis inhibitor, colony stimulating factor receptor antagonist, ephrin receptor inhibitor, FGFR inhibitor, KIT inhibitor, PDGFR tyrosine kinase receptor inhibitor, src i
440	terreic-acid	Bruton's tyrosine kinase (BTK) inhibitor
441	ebelactone-b	pancreatic lipase inhibitor
442	NU-7441	DNA dependent protein kinase, DNA dependent protein kinase inhibitor, P glycoprotein inhibitor
443	BRD-K88761633	glucosylceramidase inhibitor
444	QL-XI-92	DDR1 inhibitor
445	maprotiline	norepinephrine reputake inhibitor, tricyclic antidepressant (TCA)
446	GW-7647	PPAR receptor agonist
447	loratadine	histamine receptor antagonist

448	gedunin	HSP inhibitor
449	dilazep	adenosine reuptake inhibitor, calcium channel antagonist, platelet aggregation inhibitor
450	ER-27319	mediator release inhibitor, syk inhibitor
451	QS-11	ARFGAP inhibitor
452	YC-1	activator of soluble guanylyl cyclase, guanylate cyclase activator, hypoxia inducible factor inhibitor
453	temsirolimus	mTOR inhibitor, cell cycle inhibitor, immunosuppressant, protein kinase inhibitor
454	BAY-59-3074	cannabinoid receptor partial agonist
455	imatinib	PDGFR tyrosine kinase receptor inhibitor, Bcr-Abl kinase inhibitor, KIT inhibitor, Abl kinase inhibitor, apoptosis stimulant, breast cancer resistance protein inhibitor, colony stimulating factor receptor inhibitor
456	torin-1	mTOR inhibitor, PI3K inhibitor
457	paclitaxel	tubulin inhibitor, microtubule stabilizing agent, microtubule stimulant, P glycoprotein inhibitor, taxane
458	chlorprothixene	dopamine receptor, dopamine receptor antagonist
459	vanoxerine	dopamine reuptake inhibitor, dopamine transporter inhibitor, monoamine oxidase inhibitor
460	dactinomycin	DNA directed RNA polymerase inhibitor, nucleic acid syntheis inhibitor, protein synthesis inhibitor
461	olaparib	PARP inhibitor, DNA repair enzyme inhibitor
462	losartan	angiotensin receptor antagonist
463	clomifene	estrogen receptor antagonist, estrogen receptor modulator, selective estrogen receptor modulator (SERM), testosterone receptor agonist
464	isogedunin	HSP inhibitor
465	taxifolin	apolipoprotein secretion inhibitor, beta amyloid aggregation inhibitor, cholesterol biosynthesis inhibitor, HMGCR inhibitor, NFkB pathway modulator, opioid receptor antagonist, reverse transcriptase inhibitor
466	metergoline	dopamine receptor agonist, serotonin receptor antagonist, prolactin inhibitor
467	idarubicin	topoisomerase inhibitor, radical formation stimulant, RNA synthesis inhibitor
468	budesonide	glucocorticoid receptor agonist, glucocordicoid receptor antagonist, immunosuppressant
469	RO-90-7501	amyloid precursor protein inhibitor, beta amyloid aggregation inhibitor
470	BRD-K08438429	glucosylceramidase inhibitor, somatostatin receptor agonist
471	rimcazole	sigma receptor antagonist, dopamine reuptake inhibitor
472	eriodictyol	cytochrome P450 inhibitor, TRPM3 antagonist, xanthine oxidase inhibitor
473	AKT-inhibitor-1-2	AKT inhibitor, PI3K inhibitor
474	BRD-K20168484	lipoxygenase inhibitor, leukotriene synthesis inhibitor, PPAR receptor modulator
475	tyrphostin-47	EGFR inhibitor, epidermal growth factor receptor (EGFR) inhibitor
476	oleoylethanolamide	Glucose-Dependent Insulinotropic Receptor (GDIR, GPR119) Agonists, potassium channel blocker, PPAR receptor agonist
477	BRD-K00313977	APEX inhibitor
478	arctigenin	adiponectin receptor agonist, AP inhibitor, aryl hydrocarbon receptor antagonist, HIV integrase inhibitor, MEK inhibitor, NFkB pathway inhibitor, topoisomerase inhibitor
479	MRS-1220	adenosine receptor antagonist
480	WYE-354	mTOR inhibitor
481	daunorubicin	RNA synthesis inhibitor, topoisomerase inhibitor, DNA synthesis inhibitor, radical formation stimulant
482	BRD-A07614565	botulin neurotoxin inhibitor
483	fluphenazine	dopamine receptor antagonist, acetylcholine receptor ligand, dopamine receptor, histamine receptor antagonist, serotonin receptor antagonist
484	LY-303511	casein kinase inhibitor, mTOR inhibitor, PI3K inhibitor
485	SB-216641	serotonin receptor antagonist
486	isoquercetin	aldose reductase inhibitor, falcipain inhibitor
487	levonorgestrel	estrogen receptor agonist, glucocorticoid receptor antagonist, progesterone receptor agonist, progesterone receptor antagonist
488	4-hydroxyretinoic-acid	retinoic acid metabolite
489	picrotoxin	GABA receptor antagonist
490	mesalazine	cyclooxygenase inhibitor, arylamine N-acetyltransferase inhibitor, beta catenin inhibitor, lipoxygenase inhibitor, prostanoid receptor inhibitor, protein phosphatase inhibitor, thromboxane receptor antagonist, thromboxane syntha
491	HEAT	adrenergic receptor antagonist
492	nelfinavir	HIV protease inhibitor, proteasome inhibitor
493	arvanil	TRPV agonist, cannabinoid receptor agonist
494	SCH-58261	adenosine receptor antagonist
495	fipronil	GABA gated chloride channel blocker, glutamate gated chloride channel blocker
496	medroxyprogesterone	progesterone receptor agonist
497	r(-)-propylnorapomorphine	dopamine receptor agonist, antiamyloidogenic agent
498	thiothixene	dopamine receptor, dopamine receptor antagonist
499	BI-78D3	JNK inhibitor
500	KU-C103871	GSP agonist
501	A-443644	AKT inhibitor
502	KUC103904N	opioid receptor antagonist
503	AS-601245	JNK inhibitor
504	chlormadinone	progesterone receptor agonist
505	RS-504393	CC chemokine receptor antagonist
506	alprazolam	GABA benzodiazepine site receptor agonist, benzodiazepine receptor agonist
507	BRD-K84421793	dopamine receptor agonist
508	primaquine	antimalarial agent, DNA inhibitor
509	indirubin	CDK inhibitor, glycogen synthase kinase inhibitor, cyclin-dependent kinase inhibitor, PKC inhibitor
510	mifepristone	glucocorticoid receptor antagonist, progesterone receptor antagonist, androgen receptor ligand, internal ribosomal entry site inhibitor
511	quinidine	cytochrome P450 inhibitor, P glycoprotein inhibitor, sodium current blocker
512	staurosporine	PKC inhibitor, AKT inhibitor, BMX inhibitor, CDK inhibitor, CHK inhibitor, G protein coupled receptor agonist, glycogen synthase kinase inhibitor, leucine rich repeat kinase inhibitor, ribosomal protein inhibitor, sodium/hydrogen e
513	antimycin-a	antibiotic, electron transfer inhibitor
514	lacidipine	calcium channel blocker, L-type calcium channel blocker
515	BRD-K97274161	beta-catenin inhibitor, opioid receptor antagonist
516	doxorubicin	topoisomerase inhibitor, DNA intercalating drug
517	sphingosine	ceramidase inhibitor
518	ellipticine	topoisomerase inhibitor, DNA intercalating drug
519	tosyl-phenylalanyl-chloromethyl-ketone	chymotrypsin inhibitor, serine protease inhibitor
520	clocortolone	glucocorticoid receptor agonist
521	CDC	lipoxygenase inhibitor
522	tremulacin	5-lipoxygenase inhibitor
523	physostigmine	acetylcholinesterase inhibitor, cholinesterase inhibitor
524	eliprodil	glutamate receptor antagonist

524	eliprodil	glutamate receptor antagonist
525	BRD-K85853281	radical formation stimulant, RNA synthesis inhibitor, topoisomerase inhibitor
526	tyrphostin-AG-494	epidermal growth factor receptor (EGFR) inhibitor, tyrosine kinase inhibitor
527	epoxycholesterol	LXR agonist
528	APHA-compound-8	HDAC inhibitor
529	dactinomycin	DNA directed RNA polymerase inhibitor, nucleic acid syntheis inhibitor, protein synthesis inhibitor
530	AG-14361	PARP inhibitor
531	aloisine	CDK inhibitor, CFTR channel activator, glycogen synthase kinase inhibitor, JNK inhibitor
532	BIBX-1382	EGFR inhibitor, tyrosine kinase inhibitor
533	H-9	cAMP dependent protein kinase inhibitor, PKA inhibitor
534	vidarabine	adenylyl cyclase inhibitor, DNA directed DNA polymerase inhibitor, viral DNA synthesis inhibitor
535	trimidox	antimetabolite, ribonucleoside reductase inhibitor, ribonucleotide reductase inhibitor
536	chlorambucil	DNA damaging, DNA inhibitor
537	R-59022	diacylglycerol kinase inhibitor, protein kinase inhibitor
538	digoxigenin	ATPase inhibitor
539	mifepristone	glucocorticoid receptor antagonist, progesterone receptor antagonist, androgen receptor ligand, internal ribosomal entry site inhibitor
540	XMD-885	leucine rich repeat kinase inhibitor, MAP kinase inhibitor
541	ZK-93426	benzodiazepine receptor agonist, benzodiazepine receptor antagonist, GABA benzodiazepine site receptor antagonist
542	tacrolimus	calcineurin inhibitor, FK506-binding protein inhibitor, immunosuppressant, insulin expression inhibitor, interleukin receptor antagonist, macrolide calcineurin inhibitor, rotamase inhibitor, T cell inhibitor
543	alimemazine	histamine receptor ligand
544	lysylphenylalanyl-tyrosine	heparin activation inhibitor
545	pregnenolone	acetylcholine release enhancer, dopamine release enhancer, GABA receptor negative allosteric modulator, glutamate receptor modulator, progesterone receptor agonist, steroid hormone inhibitor of CYP17A1 and SULT2B1
546	zidovudine	nucleoside reverse transcriptase inhibitor, reverse transcriptase inhibitor
547	PD-160170	neuropeptide receptor antagonist
548	chromomycin-a3	DNA binding
549	mitotane	antineoplastic agent
550	latanoprost	prostanoid receptor agonist
551	capsaicin	TRPV agonist, NFkB pathway inhibitor, tumor NADH oxidase inhibitor
552	dihydrosamidin	nitric oxide production inhibitor, phospholipase inhibitor, platelet activating factor receptor antagonist
553	rifapentine	DNA directed RNA polymerase inhibitor, DNA directed DNA polymerase inhibitor
554	flavanone	11-beta hydroxysteroid dehydrogenase inhibitor
555	rhamnetin	aldose reductase inhibitor, JNK inhibitor, lipoxygenase inhibitor, nitric oxide production inhibitor, p38 MAPK inhibitor
556	daunorubicin	RNA synthesis inhibitor, topoisomerase inhibitor, DNA synthesis inhibitor, radical formation stimulant
557	cyclopamine	smoothened receptor antagonist, hedgehog pathway inhibitor
558	loteprednol	corticosteroid agonist, phospholipase inhibitor
559	linifanib	PDGFR tyrosine kinase receptor inhibitor, VEGFR inhibitor, angiogenesis inhibitor, colony stimulating factor receptor antagonist, colony stimulating factor receptor inhibitor, FLT3 inhibitor, macrophage colony stimulating factor a
560	INCA-6	calcineurin inhibitor, Inhibitor of interaction between calcineurin and its substrate nuclear factor of activated T cells (NFAT)
561	SB-408124	orexin receptor antagonist, orexin type 1 receptor antagonist
562	JWE-035	Aurora kinase inhibitor
563	clotrimazole	cell wall synthesis inhibitor, cytochrome P450 inhibitor, imidazole inhibitor, intermediate conductance potassium channel activator, intermediate conductance potassium channel blocker, lanosterol demethylase inhibitor, sterol d
564	fludarabine	DNA synthesis inhibitor, DNA repair enzyme inhibitor, purine antagonist, ribonucleotide reductase inhibitor
565	phenethyl-isothiocyanate	cancer cell growth inhibitor, unidentified pharmacological activity
566	butoconazole	cell wall synthesis inhibitor, sterol demethylase inhibitor
567	oxybutynin	acetylcholine receptor antagonist
568	NVP-BEZ235	mTOR inhibitor, PI3K inhibitor, protein kinase inhibitor
569	paroxetine	cyclophilin inhibitor, norepinephrine reputake inhibitor, selective serotonin reuptake inhibitor (SSRI), serotonin reuptake inhibitor, serotonin uptake inhibitor
570	papaverine	phosphodiesterase inhibitor
571	honokiol	AKT inhibitor, neurotrophic agent
572	aminolevulinic-acid	oxidizing agent
573	fluticasone	glucocorticoid receptor agonist
574	rimcazole	sigma receptor antagonist, dopamine reuptake inhibitor
575	arcyriaflavin-a	CDK inhibitor
576	cefdinir	cell wall synthesis inhibitor
577	tamibarotene	retinoid receptor agonist, RAR agonist
578	razoxane	topoisomerase inhibitor, chelating agent
579	ezetimibe	cholesterol absorption inhibitor, Niemann-Pick C1-like 1 protein antagonist, liver bile acid transporter inhibitor, Niemann-Pick C1-like 1 protein inhibitior
580	OMDM-2	FAAH reuptake inhibitor
581	KN-62	calcium/calmodulin dependent protein kinase inhibitor, purinergic receptor antagonist, calmodulin antagonist
582	epinephrine	carbonic anhydrase activator, hormone, neurotransmitter
583	prednicarbate	corticosteroid agonist, immunosuppressant, phospholipase activator
584	BRD-K71726959	CDK inhibitor
585	spiperone	dopamine receptor antagonist
586	danazol	estrogen receptor antagonist, luteinizing hormone releasing hormone antagonist, progesterone receptor agonist, steroid derivative with antigonadotropic and anti-estrogenic activities, tumor necrosis factor modulator
587	AG-490	epidermal growth factor receptor (EGFR) inhibitor, ErbB2 and JAK2 inhibitor, JAK inhibitor
588	formoterol	adrenergic receptor agonist
589	cefixime	cell wall synthesis inhibitor
590	BRD-A05680309	caspase activator, XIAP inhibitor
591	doxorubicin	topoisomerase inhibitor, DNA intercalating drug
592	syrosingopine	vesicular monoamine transporter inhibitor
593	BIX-01338	histone methyltransferase inhibitor
594	norgestimate	progesterone receptor agonist
595	ITE	aryl hydrocarbon receptor agonist, aryl hydrocarbon receptor ligand
596	propylpyrazole	estrogen receptor agonist
597	pterostilbene	cyclooxygenase inhibitor, FAAH inhibitor, MAP kinase inhibitor, PPAR receptor agonist
598	suloctidil	adrenergic receptor antagonist, platelet aggregation inhibitor, vasodilator
599	dihydroergocristine	adrenergic receptor antagonist, prolactin inhibitor, adrenergic receptor partial agonist, dopamine receptor agonist, dopamine receptor partial agonist, dopamine receptor partial antagonist, serotonin receptor antagonist
600	ascorbic-acid	vitamin C, antioxidant
601	NSC-119889	botulin neurotoxin inhibitor, mur synthetase inhibitor

601	NSC-119889	botulin neurotoxin inhibitor, mur synthetase inhibitor
602	clobetasol	glucocorticoid receptor agonist
603	NBI-27914	CRF receptor antagonist
604	nelfinavir	HIV protease inhibitor, proteasome inhibitor
605	5-iodotubercidin	adenosine kinase inhibitor, nucleoside transporter inhibitor
606	diphenoxylate	opoid receptor agonist
607	D-64406	PDGFR tyrosine kinase receptor inhibitor
608	ethinyl-estradiol	DNA directed DNA polymerase stimunlant, estrogen receptor agonist, estrogenic component in oral contraceptives
609	doxazosin	adrenergic receptor antagonist
610	SDZ-NKT-343	tachykinin antagonist
611	gingerol	nitric oxide synthase inhibitor
612	hexamethyleneamiloride	gonadotropin releasing factor hormone receptor antagonist, sodium/hydrogen antiport inhibitor, sodium/hydrogen exchanger inhibitor, urokinase inhibitor
613	indometacin	cyclooxygenase inhibitor
614	SNS-314	Aurora kinase inhibitor
615	VAMA-37	DNA dependent protein kinase, DNA dependent protein kinase inhibitor, PI3K inhibitor
616	metaxalone	muscle relaxant
617	methandriol	androgenic horomone
618	olmesartan	angiotensin receptor antagonist, angiotensin antagonist
619	dehydrocholic-acid	gastrointestinal agent that stimulates bile production or the flow of bile into the duodenum
620	motesanib	KIT inhibitor, PDGFR tyrosine kinase receptor inhibitor, VEGFR inhibitor, angiogenesis inhibitor, RET tyrosine kinase inhibitor, vascular endothelial growth factor receptor (VEGFR) inhibitor, VEGFR antagonist
621	KUC103898N	G protein coupled receptor
622	U-18666A	oxidosqualene cyclase inhibitor
623	CP466722	ATM kinase inhibitor
624	zaldaride	calmodulin antagonist, calmodulin inhibitor
625	topiramate	carbonic anhydrase inhibitor, glutamate receptor antagonist, kainate receptor antagonist, GABA receptor agonist, Sodium Channel Blockers, voltage-gated sodium channel blocker
626	MW-STK33-3B	large conductance potassium channel activator
627	dimercaptosuccinic-acid	chelating agent, DNA methyltransferase inhibitor
628	flumetasone	glucocorticoid receptor agonist
629	flunisolide	cytochrome P450 inhibitor, corticosteroid agonist, corticosteroid hormone receptor agonist, glucocorticoid receptor agonist, immunosuppressant
630	artesunate	DNA synthesis inhibitor
631	lobeline	acetylcholine receptor antagonist, dopamine receptor modulator, opioid receptor antagonist, vesicular monoamine transporter ligand
632	medrysone	corticosteroid hormone receptor agonist, glucocorticoid receptor agonist
633	HA-14-1	BCL inhibitor
634	GBR-13069	dopamine uptake inhibitor
635	skatole	thrombin inhibitor
636	fluvastatin	HMGCR inhibitor
637	flumetasone	glucocorticoid receptor agonist
638	XMD-892	MAP kinase inhibitor, BMK inhibitor, leucine rich repeat kinase inhibitor
639	AY-9944	hedgehog pathway inhibitor
640	H-7	PKA inhibitor
641	palbociclib	CDK inhibitor
642	zimelidine	serotonin uptake inhibitor
643	tibolone	estrogen receptor agonist, androgen receptor agonist, progesterone receptor agonist, selective estrogen receptor modulator (SERM), steryl sulfatase inhibitor
644	indole	aryl hydrocarbon receptor agonist, indoleamine 2,3-dioxygenase inhibitor
645	mibefradil	T-type calcium channel blocker, angiogenesis inhibitor, calcium channel blocker, calcium channel inhibitor, L-type calcium channel blocker, sodium channel blocker
646	wiskostatin	neural Wiskott-Aldrich syndrome protein inhibitor
647	norethindrone	ovulation inhibitor, progesterone receptor agonist
648	bifonazole	cell wall synthesis inhibitor, sterol demethylase inhibitor
649	ganciclovir	DNA directed DNA polymerase inhibitor, DNA polymerase inhibitor, DNA synthesis inhibitor
650	altrenogest	progestogen steroid hormone
651	clemastine	histamine receptor antagonist
652	amperozide	dopamine receptor antagonist, FAAH inhibitor, serotonin receptor antagonist
653	emodic-acid	laxative, free radical generator
654	pidorubicine	topoisomerase inhibitor, DNA intercalating drug
655	thiazolidinecarboxylic-acid	glutathione synthase stimulant, reducing agent
656	benactyzine	acetylcholine receptor antagonist
657	YM-90709	IL5 inhibitor, interleukin receptor antagonist
658	daunorubicin	RNA synthesis inhibitor, topoisomerase inhibitor, DNA synthesis inhibitor, radical formation stimulant
659	KIN001-220	Aurora kinase inhibitor
660	MNITMT	lymphocyte inhibitor
661	KU-C103885	cystic fibrosis transmembrane conductance regulator inhibitor
662	ezetimibe	cholesterol absorption inhibitor, Niemann-Pick C1-like 1 protein antagonist, liver bile acid transporter inhibitor, Niemann-Pick C1-like 1 protein inhibitior
663	PI-103	PI3K inhibitor, mTOR inhibitor
664	DCPIB	chloride channel blocker, gap junction modulator, glutamate transporter inhibitor, membrane permeability inhibitor
665	etomoxir	carnitine palmitoyltransferase inhibitor, carnitine O-palmitoyltransferase inhibitor, fatty acid oxidation inhibitor
666	diflorasone	corticosteroid agonist, cytochrome P450 inhibitor, glucocorticoid receptor agonist, immunosuppressant
667	BRD-K14609189	PPAR receptor partial agonist
668	epothilone-a	microtubule stabilizing agent
669	perphenazine	dopamine receptor, dopamine receptor antagonist
670	duloxetine	norepinephrine reuptake inhibitor, serotonin reuptake inhibitor, adrenergic transmitter uptake inhibitor, norepinephrine transporter inhibitor, serotonin transporter (SERT) inhibitor, serotonin uptake inhibitor
671	scopoline	acetylcholine receptor antagonist
672	BRD-K65285700	cannabinoid receptor agonist
673	NGB-2904	dopamine receptor antagonist
674	LY-225910	CCK receptor antagonist
675	SIB-1893	glutamate receptor antagonist
676	dephostatin	tyrosine phosphatase inhibitor
677	benzyl-quinazolin-4-yl-amine	epidermal growth factor receptor (EGFR) inhibitor
[illegible]	[illegible]	[illegible]

678	indirubin	CDK inhibitor, glycogen synthase kinase inhibitor, cyclin-dependent kinase inhibitor, PKC inhibitor
679	econazole	cell wall synthesis inhibitor, lanosterol demethylase inhibitor, sterol demethylase inhibitor
680	MK-2206	AKT inhibitor
681	fluspirilene	dopamine receptor, dopamine receptor antagonist
682	estradiol-cypionate	estrogen receptor agonist
683	molindone	dopamine receptor antagonist
684	cabergoline	dopamine receptor agonist, prolactin inhibitor, prolactin secretion inhibitor
685	CO-101244	ionotropic glutamate receptor antagonist
686	GW-3965	LXR agonist, ABC transporter expression enhancer
687	BW-B70C	lipoxygenase inhibitor
688	rimantadine	acetylcholine channel inhibitor, unidentified pharmacological activity
689	mocimycin	protein synthesis inhibitor
690	testosterone	androgen receptor (AR) agonist, androgen receptor agonist, testosterone receptor agonist
691	lawsone	coloring agent
692	HY-11007	Abl kinase inhibitor, Bcr-Abl kinase inhibitor
693	AMG-9810	TRPV antagonist
694	olvanil	TRPV agonist
695	nitrendipine	calcium channel blocker, L-type calcium channel blocker
696	ceforanide	cell wall synthesis inhibitor, penicillin binding protein inhibitor
697	piceatannol	syk inhibitor, cyclooxygenase inhibitor, nitric oxide synthase inhibitor, SIRT activator, tyrosinase inhibitor, tyrosine kinase inhibitor
698	tacedinaline	HDAC inhibitor, cell cycle inhibitor
699	aminoindazole	ionophore
700	sirolimus	mTOR inhibitor, CCR expression inhibitor, cell cycle inhibitor, proteasome inhibitor, protein kinase inhibitor, T cell inhibitor
701	III606050	cytochrome P450 inhibitor
702	L-655240	platelet aggregation inhibitor, prostanoid receptor antagonist, thromboxane receptor antagonist
703	tacrolimus	calcineurin inhibitor, FK506-binding protein inhibitor, immunosuppressant, insulin expression inhibitor, interleukin receptor antagonist, macrolide calcineurin inhibitor, rotamase inhibitor, T cell inhibitor
704	amiodarone	acetylcholine receptor ligand, adrenergic receptor antagonist, ATP-sensitive potassium channel inhibitor, HCN (hyperpolarization activated cyclic nucleotide gated) channel modulator, polarization inhibitor, potassium channel a
705	progesterone	progesterone receptor agonist
706	tropisetron	serotonin receptor antagonist, polarization inhibitor, sodium channel blocker
707	penitrem-a	potassium channel blocker
708	CITCO	constitutive androstane receptor (CAR) agonist
709	resveratrol	apolipoprotein expression enhancer, beta-secretase inhibitor, cyclooxygenase inhibitor, cytochrome P450 inhibitor, lipid peroxidase inhibitor, MAP kinase inhibitor, monoamine oxidase inhibitor, NFkB pathway modulator, SIRT a
710	enalapril	angiotensin converting enzyme inhibitor, angiotensin receptor blocker
711	esomeprazole	ATPase inhibitor, ABC transporter expression enhancer
712	nimodipine	calcium channel blocker, L-type calcium channel blocker
713	cilnidipine	calcium channel blocker, L-type calcium channel blocker, N-type calcium channel blocker
714	isoeugenol	nitric oxide production inhibitor
715	O-2050	cannabinoid receptor antagonist
716	lofepramine	adrenergic transmitter uptake inhibitor, norepinephrine reuptake inhibitor, serotonin reuptake inhibitor, serotonin uptake inhibitor, tricyclic antidepressant
717	nefazodone	serotonin receptor antagonist, serotonin reuptake inhibitor, adrenergic transmitter uptake inhibitor, norepinephrine reuptake inhibitor, serotonin uptake inhibitor
718	eudesmic-acid	in touchstone
719	clobetasol	glucocorticoid receptor agonist
720	arachidonamide	cannabinoid receptor agonist
721	RG-14620	EGFR inhibitor
722	parbendazole	tubulin inhibitor
723	mocetinostat	HDAC inhibitor, cell cycle inhibitor
724	GR-46611	serotonin receptor agonist
725	dexketoprofen	cyclooxygenase inhibitor
726	UCL-2077	potassium channel blocker, slow afterhyperpolarization channel blocker
727	isradipine	calcium channel blocker, L-type calcium channel blocker
728	itraconazole	cell wall synthesis inhibitor, cytochrome P450 inhibitor, sterol demethylase inhibitor, VEGFR inhibitor
729	amitriptyline	norepinephrine inhibitor, serotonin receptor inhibitor, sigma receptor inhibitor
730	methylergometrine	dopamine receptor antagonist, partial serotonin receptor agonist
731	parthenolide	NFkB pathway inhibitor, adiponectin receptor agonist
732	GW-6471	PPAR receptor antagonist
733	fludarabine	DNA synthesis inhibitor, DNA repair enzyme inhibitor, purine antagonist, ribonucleotide reductase inhibitor
734	lidoflazine	calcium channel antagonist
735	triamcinolone	antiinflammatory agent, corticosteroid agonist, corticosteroid binding globulin binder, corticosteroid hormone receptor agonist, cytochrome P450 inhibitor, glucocorticoid receptor agonist, immunosuppressant, immunosuppressi
736	alfacalcidol	hypercalcaemic agent, vitamin D receptor agonist
737	amantadine	glutamate receptor antagonist, acetylcholine channel inhibitor, dopamine receptor agonist
738	CGP-37157	L-type calcium channel blocker, mitochondrial Na+/Ca2+ exchanger antagonist, sodium/calcium exchange inhibitor
739	sertaconazole	sterol demethylase inhibitor, cell wall synthesis inhibitor
740	entecavir	DNA directed DNA polymerase inhibitor, DNA polymerase inhibitor, DNA replication inhibitor, reverse transcription inhibitor, transcription inhibitor
741	mestanolone	androgenic steroid
742	hydrocortisone	corticosteroid agonist, glucocorticoid receptor agonist, immunosuppressant, interleukin receptor antagonist
743	iloprost	platelet aggregation inhibitor, prostanoid receptor agonist, prostacyclin analog
744	AM-404	FAAH transport inhibitor, anandamide transport inhibitor, nucelar factor of activated T-cells inhibitor, TRPV agonist
745	altretamine	DNA synthesis inhibitor
746	balsalazide	cyclooxygenase inhibitor, prostanoid receptor inhibitor, thromboxane receptor antagonist
747	midazolam	benzodiazepine receptor agonist, GABA benzodiazepine site receptor agonist
748	hyperoside	glucosidase inhibitor, free radical scavenger
749	LY-278584	serotonin receptor antagonist
750	ataluren	CFTR channel agonist, coagulation stimulant, dystrophin stimulant
751	candesartan	angiotensin receptor antagonist
752	triclabendazole	acetylcholinesterase inhibitor, DNA methyltransferase inhibitor, microtubule inhibitor
753	PETCM	caspase activator
754	modafinil	adrenergic receptor agonist, dopamine receptor reuptake inhibitor

755	BRD-K55055802	membrane integrity inhibitor, membrane permeability inhibitor
756	atracurium	acetylcholine receptor antagonist
757	rifabutin	DNA directed DNA polymerase inhibitor, DNA directed RNA polymerase inhibitor, protein synthesis inhibitor
758	acitretin	retinoid receptor agonist, microbial collagenase inhibitor, protein synthesis inhibitor
759	mirtazapine	adrenergic receptor antagonist, serotonin receptor antagonist
760	dichlorobenzamil	sodium/calcium exchange inhibitor
761	reichstein	androgen receptor antagonist
762	budesonide	glucocorticoid receptor agonist, glucocordicoid receptor antagonist, immunosuppressant
763	isoreserpine	vesicular monoamine transporter inhibitor
764	PSB-36	adenosine receptor antagonist
765	fluocinolone	corticosteroid agonist, glucocorticoid receptor agonist
766	medrysone	corticosteroid hormone receptor agonist, glucocorticoid receptor agonist
767	triflupromazine	acetylcholine receptor ligand, dopamine receptor
768	nilotinib	Bcr-Abl kinase inhibitor, Abl kinase inhibitor, discoidin domain containing receptor inhibitor, KIT inhibitor, PDGFR tyrosine kinase receptor inhibitor
769	PD-123319	angiotensin antagonist
770	flutamide	androgen receptor antagonist
771	bimatoprost	prostanoid receptor agonist
772	BRD-K11757396	neuropeptide receptor ligand
773	hyoscyamine	acetylcholine receptor inhibitor
774	coumaric-acid	antioxidant
775	terbinafine	fungal squalene monooxygenase inhibitor, squalene epoxidase inhibitor, steroid sulfatase inhibitor
776	nefopam	cyclooxygenase inhibitor, prostanoid receptor inhibitor
777	chlorpromazine	dopamine receptor, dopamine receptor antagonist, Kinesin-Like Spindle Protein KIF11 (KSP, Eg5) Inhibitors
778	trimethobenzamide	histamine receptor antagonist
779	ketoconazole	14-alpha demethylase inhibitor, androgen receptor ligand, aromatase inhibitor, cell wall synthesis inhibitor, cortisol synthesis inhibitor, P glycoprotein inhibitor, sterol demethylase inhibitor
780	tretinoin	RAR agonist, RAR receptor binder, retinoid receptor agonist, ROR ligand
781	GR-159897	tachykinin antagonist
782	roxatidine	histamine receptor antagonist
783	BRD-A17664363	steroidogenic factor antagonist
784	amiodarone	acetylcholine receptor ligand, adrenergic receptor antagonist, ATP-sensitive potassium channel inhibitor, HCN (hyperpolarization activated cyclic nucleotide gated) channel modulator, polarization inhibitor, potassium channel a[illegible]
785	purvalanol-b	tyrosine kinase inhibitor
786	noretynodrel	progestogen steroid hormone
787	BRD-A28422330	CDC inhibitor
788	BRD-A12633378	voltage-gated potassium channel activator
789	CFM-1571	guanylate cyclase activator
790	fenobam	glutamate receptor antagonist
791	pizotifen	serotonin receptor antagonist
792	avrainvillamide-analog-5	nucleophosmin inhibitor
793	leu-enkephalin	opioid receptor agonist
794	cosmosiin	cytochrome P450 inhibitor
795	nicardipine	calcium channel blocker, L-type calcium channel blocker
796	BRD-A53107311	mitotic kinesin inhibitor, Tubulin Polymerization Inhibitors
797	JTE-013	lysophospholipid receptor antagonist
798	metronidazole	DNA inhibitor
799	SB-205607	delta 1 opioid receptor agonist, opioid receptor agonist
800	estradiol	estrogen receptor agonist
801	trazodone	adrenergic receptor antagonist, serotonin receptor antagonist, serotonin reuptake inhibitor, histamine receptor antagonist, serotonin uptake inhibitor
802	BAY-36-7620	glutamate receptor antagonist
803	BRD-A94297859	XIAP inhibitor
804	terconazole	cell wall synthesis inhibitor, sterol demethylase inhibitor
805	desoxycorticosterone	mineralocorticoid receptor agonist
806	desoximetasone	glucocorticoid receptor agonist
807	tosufloxacin	topoisomerase inhibitor
808	RS-102895	CC chemokine receptor antagonist, CCR antagonist
809	nicergoline	adrenergic receptor antagonist
810	gliquidone	sulfonylurea, ATP channel blocker, ATP-sensitive potassium channel antagonist, insulin secretagogue
811	mepireserpate	catecholamine depleting sympatholytic
812	nilutamide	androgen receptor antagonist, RNA directed RNA polymerase inhibitor
813	depomedrol	glucocorticoid receptor agonist
814	paroxetine	cyclophilin inhibitor, norepinephrine reputake inhibitor, selective serotonin reuptake inhibitor (SSRI), serotonin reuptake inhibitor, serotonin uptake inhibitor
815	omeprazole	ATPase inhibitor, ABC transporter expression enhancer
816	benzoxiquine	topical anti-infective
817	mepivacaine	potassium channel antagonist, voltage-gated sodium channel blocker
818	fluocinonide	corticosteroid agonist, corticosteroid hormone receptor agonist
819	PK-11195	benzodiazepine receptor antagonist, constitutive androstane receptor (CAR) antagonist, PXR agonist, translocator protein antagonist
820	avrainvillamide-analog-4	nucleophosmin inhibitor
821	gamma-linolenic-acid	cyclooxygenase inhibitor, prostanoid receptor stimulant, thromboxane synthase stimulant
822	ricinine	casein kinase inhibitor
823	dantron	stimulant laxative
824	dexbrompheniramine	histamine receptor antagonist
825	delcorine	ganglioblocking, antiarrhythmic
826	L-755507	adrenergic receptor agonist, adrenergic receptor partial agonist
827	zonisamide	GABA receptor agonist, Sodium Channel Blockers, T-type calcium channel blocker
828	triamcinolone	antiinflammatory agent, corticosteroid agonist, corticosteroid binding globulin binder, corticosteroid hormone receptor agonist, cytochrome P450 inhibitor, glucocorticoid receptor agonist, immunosuppressant, immunosuppressi[illegible]
829	fluoxetine	selective serotonin reuptake inhibitor (SSRI), serotonin transporter (SERT) inhibitor, serotonin uptake inhibitor, Sodium Channel Blockers
830	tyrphostin-AG-538	insulin growth factor receptor inhibitor, tyrosine kinase inhibitor
831	MY-5445	phosphodiesterase inhibitor, platelet aggregation inhibitor

		[illegible]
832	nateglinide	insulin secretagogue, ATP-sensitive potassium channel antagonist, meglitinide, sulphonylurea receptor activator
833	triamcinolone	antiinflammatory agent, corticosteroid agonist, corticosteroid binding globulin binder, corticosteroid hormone receptor agonist, cytochrome P450 inhibitor, glucocorticoid receptor agonist, immunosuppressant, immunosuppressi
834	meropenem	cell wall synthesis inhibitor
835	rifapentine	DNA directed RNA polymerase inhibitor, DNA directed DNA polymerase inhibitor
836	BRD-A89208012	prostanoid receptor antagonist, thromboxane receptor antagonist
837	triamcinolone	antiinflammatory agent, corticosteroid agonist, corticosteroid binding globulin binder, corticosteroid hormone receptor agonist, cytochrome P450 inhibitor, glucocorticoid receptor agonist, immunosuppressant, immunosuppressi
838	amthamine	histamine receptor agonist
839	cyclosporin-a	calcineurin inhibitor, cyclophilin inhibitor, immunosuppressant, insulin expression inhibitor, T cell inhibitor
840	amlexanox	histamine release inhibitor, mediator release inhibitor
841	calcitriol	vitamin D receptor agonist, hypercalcaemic agent
842	strychnine	acetylcholine receptor antagonist
843	OSI-027	mTOR inhibitor
844	erythrosine	food coloring agent
845	SKF-86002	p38 MAPK inhibitor
846	halometasone	cytochrome P450 inhibitor, glucocorticoid receptor agonist, immunosuppressant
847	bezafibrate	PPAR receptor agonist, 11-beta hydroxysteroid dehydrogenase expression inhibitor, 11-beta hydroxysteroid dehydrogenase inhibitor, lipase clearing factor inhibitor
848	telenzepine	acetylcholine receptor antagonist
849	ketorolac	cyclooxygenase inhibitor
850	cyclophosphamide	alkylating agrent, BCL inhibitor, galectin inhibitor
851	scandenin	plant compound with antimicrobiial activity
852	dihydroergocristine	adrenergic receptor antagonist, prolactin inhibitor, adrenergic receptor partial agonist, dopamine receptor agonist, dopamine receptor partial agonist, dopamine receptor partial antagonist, serotonin receptor antagonist
853	amlodipine	breast cancer resistance protein inhibitor, calcium channel blocker, calcium channel inhibitor, L-type calcium channel blocker
854	cyclazosin	adrenergic receptor antagonist
855	corticosterone	mineralocorticoid receptor agonist
856	GTP-14564	FLT3 inhibitor, tyrosine kinase receptor inhibitor
857	avrainvillamide-analog-3	nucleophosmin inhibitor
858	sulpiride	carbonic anhydrase inhibitor, dopamine receptor, dopamine receptor antagonist
859	danazol	estrogen receptor antagonist, luteinizing hormone releasing hormone antagonist, progesterone receptor agonist, steroid derivative with antigonadotropic and anti-estrogenic activities, tumor necrosis factor modulator
860	dexamethasone	glucocorticoid receptor agonist, corticosteroid agonist, immunosuppressant
861	MRS-1334	adenosine receptor antagonist
862	piceid	glucosidase inhibitor, ICAM1 expression inhibitor, VCAM expression inhibitor, xanthine oxidase inhibitor
863	rilmenidine	adrenergic receptor agonist, imidazoline receptor agonist
864	enzastaurin	PKC inhibitor, AKT inhibitor, angiogenesis inhibitor, apoptosis stimulant, PI3K inhibitor
865	nabumetone	cyclooxygenase inhibitor, 3alpha hydroxysteroid dehydrogenase inhibitor
866	PP-30	RAF inhibitor
867	rifampicin	DNA directed RNA polymerase inhibitor, enzyme inducer
868	canrenoic-acid	aldosterone antagonist
869	cisapride	acetylcholine receptor agonist, serotonin receptor agonist
870	GBR-12935	dopamine uptake inhibitor
871	estradiol-benzoate	contraceptive agent
872	BRD-K28452084	opioid receptor agonist
873	estropipate	estrogen receptor agonist
874	chloramphenicol	30S ribosomal protein inhibitor, 50S ribosomal subunit inhibitor, antibacterial agent
875	BRD-A93048969	acetylcholine receptor antagonist
876	secnidazole	acetylcholinesterase inhibitor, microtubule inhibitor
877	amoxicillin	cell wall synthesis inhibitor, PBPA inhibitor
878	BRD-K73709114	glucosylceramidase inhibitor
879	paxilline	ABC transporter expression enhancer, high conductance calcium activated potassium blocker, LXR agonist
880	megestrol	progesterone receptor agonist, DNA inhibitor, HegG2 inhibitor
881	vicriviroc	CC chemokine receptor antagonist, CC chemokine receptor 5 (CCR5) antagonist
882	levonorgestrel	estrogen receptor agonist, glucocorticoid receptor antagonist, progesterone receptor agonist, progesterone receptor antagonist
883	fluphenazine	dopamine receptor antagonist, acetylcholine receptor ligand, dopamine receptor, histamine receptor antagonist, serotonin receptor antagonist
884	metoclopramide	dopamine receptor antagonist, serotonin receptor antagonist, serotonin receptor agonist
885	mefenamic-acid	cyclooxygenase inhibitor
886	T-0901317	LXR agonist, ABC transporter expression enhancer, ROR inverse agonist
887	dofetilide	polarization inhibitor, potassium channel antagonist, potassium channel blocker
888	AMN-082	glutamate receptor positive allosteric modulator
889	oxotremorine	acetylcholine receptor agonist
890	CGP-20712	adrenergic receptor antagonist
891	quinidine	cytochrome P450 inhibitor, P glycoprotein inhibitor, sodium current blocker
892	L-750667	dopamine receptor antagonist
893	HDAC3-selective	HDAC inhibitor
894	celecoxib	cyclooxygenase inhibitor, caspase activator
895	sulforaphane	anticancer agent, aryl hydrocarbon receptor antagonist, nuclear factor erythroid derived, like (NRF2) activator
896	tranilast	angiogenesis inhibitor, histamine receptor antagonist, histamine release inhibitor, indoleamine 2,3-dioxygenase activator, interferon gamma synthesis inhibitor, interleukin receptor modulator, interleukin synthesis enhancer, inte
897	geldanamycin	HSP inhibitor
898	tribenoside	vasoprotective agent
899	desoximetasone	glucocorticoid receptor agonist
900	gelsemine	acetylcholine receptor antagonist, glycine receptor antagonist
901	chlorphensin	muscle relaxant
902	glimepiride	insulin secretagogue, ATP channel blocker, ATP-sensitive potassium channel antagonist, insulinotropin agonist, sulfonylurea
903	ceramide	nitric oxide production inhibitor, phosphoenolpyruvate carboxylase activator, serine/threonine protein phosphatase activator
904	diltiazem	L-type calcium channel blocker, calcium channel antagonist, calcium channel blocker
905	dihydroergotamine	serotonin receptor agonist, adrenergic receptor partial agonist, dopamine receptor agonist
906	WAY-629	serotonin receptor agonist
907	bifemelane	acetylcholine release stimulant, adrenergic transmitter uptake inhibitor, reducing agent
908	rotenonic-acid	RAR antagonist

[illegible]	[illegible]	RAR antagonist
909	ritodrine	adrenergic receptor agonist
910	riboflavin	vitamin B2, required for flavoprotein enzyme reactions
911	nTZDpa	PPAR receptor partial agonist
912	AM-251	ACAT inhibitor, cannabinoid receptor antagonist, cannabinoid receptor inverse agonist
913	mesulergine	dopamine receptor agonist
914	GW-4064	FXR agonist
915	cinanserin	serotonin receptor antagonist
916	asiatic-acid	antioxidant and antiinflammatory agent
917	prazosin	adrenergic receptor antagonist, adrenergic receptor blocker, CDK inhibitor
918	amodiaquine	histamine N-methyltransferase inhibitor
919	thioridazine	acetylcholine receptor ligand, dopamine receptor, dopamine receptor antagonist, mucosa associated lymphoid tissue lymphoma translocation protein 1 (MALT1) inhibitor
920	GR-235	FXR antagonist, cholesterol inhibitor, estrogen receptor agonist, IKK inhibitor, progesterone receptor agonist, PXR agonist
921	meprylcaine	anesthetic
922	chaetocin	histone lyinse methyltransferase inhibitor, histone lysine N methyltransferase inhibitor
923	bisacodyl	stimulant laxative
924	lomerizine	calcium channel antagonist, calcium channel blocker, P glycoprotein inhibitor
925	ditolylguanidine	sigma receptor agonist
926	hexylresorcinol	polyphenol oxidase inhibitor
927	rabeprazole	ATPase inhibitor, gastrin inhibitor
928	BRD-K35133769	lipoxygenase inhibitor
929	benzatropine	anticholinergic
930	SB-203580	p38 MAPK inhibitor, calcium channel activator, interleukin inhibitor, stress activated protein kinase inhibitor
931	dosulepin	adrenergic transmitter uptake inhibitor, norepinephrine reuptake inhibitor, serotonin reuptake inhibitor, serotonin uptake inhibitor, tricyclic antidepressant
932	SB-239063	p38 MAPK inhibitor, interleukin inhibitor, tumor necrosis factor production inhibitor
933	valdecoxib	cyclooxygenase inhibitor
934	JAK3-inhibitor-V	JAK inhibitor
935	moracizine	sodium channel blocker
936	irilin-a	isoflavone
937	acetylcholine	neurotransmitter of the peripheral and central nervous systems
938	quinethazone	diuretic
939	ceramide	nitric oxide production inhibitor, phosphoenolpyruvate carboxylase activator, serine/threonine protein phosphatase activator
940	tubastatin-a	HDAC inhibitor
941	7-nitroindazole	nitric oxide synthase inhibitor
942	lavendustin-a	EGFR inhibitor, tyrosine kinase inhibitor
943	DMP-543	acetylcholine release enhancer
944	paroxetine	cyclophilin inhibitor, norepinephrine reputake inhibitor, selective serotonin reuptake inhibitor (SSRI), serotonin reuptake inhibitor, serotonin uptake inhibitor
945	galantamine	acetylcholinesterase inhibitor, acetylcholine receptor agonist, butyrylcholinesterase inhibitors
946	mestinon	cholinesterase inhibitor
947	verteporfin	apoptosis stimulant, photosensitizing agent, radical formation stimulant
948	GSK-461364	PLK inhibitor
949	isradipine	calcium channel blocker, L-type calcium channel blocker
950	linopirdine	acetylcholine release enhancer, acetylcholine release stimulant, neurotransmitter agonist, voltage-gated potassium channel blocker
951	JAK3-Inhibitor-II	JAK inhibitor, ALK tyrosine kinase receptor inhibitor, EGFR inhibitor
952	betamethasone	corticosteroid agonist, glucocorticoid receptor agonist
953	dihydrodeoxygedunin	growth factor receptor activator
954	alitretinoin	RXR agonist, ABC transporter expression enhancer, apoptosis stimulant, RAR agonist, retinoid receptor agonist
955	farnesylthiotriazole	prenylated protein methyltransferase (PPMTase) inhibitor
956	salmeterol	adrenergic receptor agonist
957	doxepin	histamine receptor antagonist, adrenergic transmitter uptake inhibitor, histamine receptor inhibitor, norepinephrine reputake inhibitor, serotonin reuptake inhibitor, tricyclic antidepressant
958	donepezil	acetylcholinesterase inhibitor
959	triprolidine	histamine receptor antagonist
960	dicyclohexylurea	epoxide hydolase inhibitor
961	SB-222200	tachykinin antagonist
962	flavoxate	acetylcholine receptor antagonist
963	XE-991	acetylcholine release enhancer, potassium channel blocker, voltage-gated potassium channel blocker
964	GW-9508	Free Fatty Acid Receptor 1 (FFAR1; GPR40) Agonists, G protein coupled receptor agonist, G-protein coupled receptor agonist
965	busulfan	DNA damaging, DNA inhibitor
966	fluticasone	glucocorticoid receptor agonist
967	SCH-28080	ATPase inhibitor, potassium-competitive acid antagonist
968	metformin	insulin sensitizer, AMPK activator, biguanide, gluconeogenesis inhibitor
969	dexamethasone	glucocorticoid receptor agonist, corticosteroid agonist, immunosuppressant
970	homatropine	acetylcholine receptor antagonist
971	meptazinol	opioid receptor agonist
972	cefuroxime	cell wall synthesis inhibitor
973	demeclocycline	30S ribosomal subunit inhibitor
974	PCA-4248	platelet activating factor receptor antagonist
975	BRD-K60218670	lipoxygenase inhibitor
976	BRD-K20401833	ROR inverse agonist
977	targinine	nitric oxide synthase inhibitor
978	2-aminopurine	serine/threonine protein kinase inhibitor
979	pirarubicin	topoisomerase inhibitor
980	PIK-75	DNA protein kinase inhibitor, P110 inhibitor, Phosphatidylinositol 3-kinase (PI3K) inhibitor, PI3K inhibitor
981	noreleagnine	imidazoline receptor ligand, serotonin receptor ligand
982	azelastine	histamine receptor antagonist, histamine release inhibitor, immunosuppressant
983	oxyphenonium	acetylcholine receptor antagonist
984	GW-441756	growth factor receptor inhibitor, leucine rich repeat kinase inhibitor
985	parachlorophenol	disinfectant

985	parachlorophenol	disinfectant
986	methylprednisolone	glucocorticoid receptor agonist
987	BU-226	imidazoline 2 receptor ligand
988	pyrazolanthrone	JNK inhibitor, AP inhibitor, dual specificity protein kinase inhibitor, leucine rich repeat kinase inhibitor
989	anagrelide	phosphodiesterase inhibitor
990	bisphenol-a	synthetic compound with hormone-like properties
991	bicalutamide	androgen receptor antagonist
992	mebeverine	acetylcholine receptor antagonist
993	CDK1-5-inhibitor	CDK inhibitor
994	oleylethanolamide	Glucose-Dependent Insulinotropic Receptor (GDIR, GPR119) Agonists, potassium channel blocker
995	aristolochic-acid	phospholipase inhibitor
996	triprolidine	histamine receptor antagonist
997	raloxifene	estrogen receptor antagonist, selective estrogen receptor modulator (SERM), ALK expression enhancer, cholesterol inhibitor, endoglin expression enhancer, estrogen receptor agonist
998	RO-04-5595	ionotropic glutamate receptor antagonist
999	danusertib	Aurora kinase inhibitor, growth factor receptor inhibitor, Abl kinase inhibitor, Bcr-Abl kinase inhibitor, FGFR inhibitor, mitotic inhibitor, protein kinase inhibitor, RET tyrosine kinase inhibitor
1000	erythromycin	NFkB pathway inhibitor, 50S ribosomal subunit inhibitor, motilin receptor agonist, RPLV inhibitor
1001	torasemide	cytochrome P450 antagonist, diuretic, electrolyte reabsorption inhibitor, thromboxane receptor antagonist
1002	phosphodiesterase-V-inhibitor-II	phosphodiesterase inhibitor
1003	diphemanil	acetylcholine receptor antagonist
1004	nialamide	monoamine oxidase inhibitor
1005	nobiletin	MEK phosphorylation inhibitor, nitric oxide synthase expression inhibitor, P glycoprotein inhibitor, tyrosinase inhibitor
1006	moclobemide	monoamine oxidase inhibitor
1007	GBR-12783	dopamine uptake inhibitor
1008	gitoxigenin	ATPase inhibitor
1009	BRD-K71427192	XIAP inhibitor
1010	GR-103691	dopamine receptor antagonist
1011	BU-239	imidazoline receptor agonist, imidazoline receptor ligand
1012	KUC103420N	rho GTPase inhibitor, CDC inhibitor
1013	rofecoxib	cyclooxygenase inhibitor
1014	GSK-3-inhibitor-II	glycogen synthase kinase inhibitor, PKC inhibitor, superoxide dismutase expression inhibitor
1015	retrorsine	mutagenic, antimitotic, cytotoxic
1016	fluocinolone	corticosteroid agonist, glucocorticoid receptor agonist
1017	nalbuphine	opioid receptor agonist, opioid receptor antagonist
1018	betamethasone	corticosteroid agonist, glucocorticoid receptor agonist
1019	PRE-084	alpha receptor agonist, sigma receptor agonist
1020	erlotinib	EGFR inhibitor, epidermal growth factor receptor (EGFR) inhibitor, tyrosine kinase inhibitor
1021	PHCCC	glutamate receptor agonist, glutamate receptor modulator
1022	vesamicol	acetylcholine transport inhibitor
1023	linoleamide	ACAT inhibitor
1024	naltrindole	opioid receptor antagonist
1025	ATPA	glutamate receptor agonist
1026	toremifene	selective estrogen receptor modulator (SERM), estrogen receptor antagonist
1027	olopatadine	histamine receptor antagonist, histamine blocker, mediator release inhibitor
1028	protopine	acetylcholinesterase inhibitor, norepinephrine transporter inhibitor, serotonin transporter (SERT) inhibitor
1029	benzonatate	non-narcotic cough suppressant thought to act as a local anesthetic
1030	oxcarbazepine	voltage-gated sodium channel blocker, Sodium Channel Blockers
1031	estradiol	estrogen receptor agonist
1032	haloperidol	dopamine receptor antagonist
1033	cetraxate	gastrin inhibitor, mucus protecting agent
1034	chlorphenamine	histamine receptor antagonist
1035	isoflupredone	glucocorticoid receptor agonist
1036	PPT	estrogen receptor agonist
1037	midostaurin	FLT3 inhibitor, KIT inhibitor, PKC inhibitor, angiogenesis inhibitor, cell cycle inhibitor, cyclin inhibitor, histamine release inhibitor, multi targeted kinase inhibitor, PDGFR tyrosine kinase receptor inhibitor, VEGFR antagonist, VEG
1038	ipratropium	acetylcholine receptor antagonist
1039	GW-1929	PPAR receptor agonist, insulin sensitizer
1040	fulvestrant	estrogen receptor antagonist, DNA directed DNA polymerase inhibitor, soluble epoxide hydrolase inhibitor
1041	SQ-29548	thromboxane receptor antagonist
1042	lansoprazole	ATPase inhibitor
1043	clarithromycin	50S ribosomal subunit inhibitor
1044	prednisolone	glucocorticoid receptor agonist
1045	GBR-12783	dopamine uptake inhibitor
1046	loperamide	opioid receptor agonist, atrial natriuretic peptide receptor antagonist
1047	STO-609	calcium/calmodulin dependent protein kinase inhibitor, calmodulin inhibitor
1048	4,5,6,7-tetrabromobenzotriazole	casein kinase inhibitor
1049	MDL-72832	serotonin receptor agonist
1050	bisindolylmaleimide	CDK inhibitor, PKC inhibitor, leucine rich repeat kinase inhibitor
1051	oxiconazole	cell wall synthesis inhibitor, sterol demethylase inhibitor
1052	JWH-015	cannabinoid receptor agonist
1053	methoxsalen	acetylcholinesterase inhibitor, cytochrome P450 inhibitor, DNA damaging, DNA synthesis inhibitor, immunosuppressant
1054	BML-190	cannabinoid receptor inverse agonist
1055	tamoxifen	estrogen receptor antagonist, selective estrogen receptor modulator (SERM), estrogen receptor agonist, estrogen receptor modulator, PKC inhibitor
1056	imipramine	norepinephrine reputake inhibitor, serotonin reuptake inhibitor, Sodium Channel Blockers
1057	CAY-10585	hypoxia inducible factor inhibitor
1058	fluperlapine	dopamine receptor antagonist, serotonin receptor antagonist
1059	dextrorphan	glutamate receptor antagonist, adrenergic transmitter release inhibitor, opioid receptor agonist, serotonin receptor antagonist
1060	IAA-94	chloride channel blocker
1061	desloratadine	histamine receptor antagonist
[illegible]	[illegible]	[illegible]

1062	RS-79948	adrenoreceptor antagonist
1063	ZM-241385	adenosine receptor antagonist
1064	UK-356618	metalloproteinase inhibitor
1065	fulvestrant	estrogen receptor antagonist, DNA directed DNA polymerase inhibitor, soluble epoxide hydrolase inhibitor
1066	eicosatetraynoic-acid	cyclooxygenase inhibitor, lipoxygenase inhibitor
1067	olomoucine	CDK inhibitor
1068	nicotinamide	oxidoreductase stimulant, protein synthesis stimulant
1069	pentobarbital	GABA receptor modulator
1070	CGS-12066B	serotonin receptor agonist
1071	ibuprofen	cyclooxygenase inhibitor, NFkB pathway inhibitor
1072	tiagabine	GABA uptake inhibitor, GAT inhibitor
1073	medroxyprogesterone	progesterone receptor agonist
1074	desmethylclozapine	acetylcholine receptor agonist, dopamine receptor agonist, dopamine receptor antagonist, dopamine receptor partial agonist, serotonin receptor antagonist, serotonin receptor inverse agonist
1075	hydroquinine	anti-arrhythmia agent
1076	S-14506	serotonin receptor agonist
1077	cefpodoxime	cell wall synthesis inhibitor
1078	2-(biphenyl-4-ylsulfonamido)pentanedioic-acid	matrix metalloprotease inhibitor
1079	necrostatin-1	RIPK inhibitor, indoleamine 2,3-dioxygenase inhibitor
1080	isotretinoin	retinoid receptor agonist, microbial collagenase inhibitor, protein synthesis inhibitor, RAR agonist
1081	L-670596	prostanoid receptor antagonist
1082	fosinopril	angiotensin converting enzyme inhibitor, breast cancer resistance protein inhibitor
1083	scopolamine	acetylcholine receptor antagonist
1084	vardenafil	phosphodiesterase inhibitor
1085	trimebutine	opioid receptor agonist, acetylcholine receptor antagonist, Sodium Channel Blockers
1086	granisetron	serotonin receptor antagonist
1087	letrozole	aromatase inhibitor, estrogen receptor antagonist
1088	cisapride	acetylcholine receptor agonist, serotonin receptor agonist
1089	BMS-754807	insulin growth factor receptor inhibitor
1090	esculin	antioxidant, unidentified pharmacological activity
1091	pantoprazole	ATPase inhibitor
1092	TAS-301	voltage-independent calcium influx blocker
1093	BRD-K00256256	protein phosphatase inhibitor
1094	acebutolol	adrenergic receptor antagonist
1095	trequinsin	phosphodiesterase inhibitor, platelet aggregation inhibitor, potassium channel blocker
1096	DPO-1	potassium channel blocker
1097	QL-X-138	mTOR inhibitor
1098	xylazine	adrenergic receptor agonist
1099	thiocolchicoside	GABA receptor antagonist
1100	GS-39783	GABA receptor modulator, GABA receptor positive allosteric modulator
1101	JTE-907	cannabinoid receptor inverse agonist
1102	spironolactone	mineralocorticoid receptor antagonist, androgen receptor antagonist, potassium channel blocker
1103	PF-543	sphingosine kinase inhibitor
1104	zolpidem	benzodiazepine receptor agonist, GABA benzodiazepine site receptor agonist, non-benzodiazepine hypnotic
1105	corynanthine	alpha adrenergic receptor antagonist
1106	CCMQ	inhibitor of the binding of homoquinolinic acid to non-NMDA sensitive sites
1107	irbesartan	angiotensin receptor antagonist, liver bile acid transporter inhibitor
1108	W-7	calmodulin antagonist, potassium channel blocker, squalene monooxygenase channel blocker
1109	saquinavir	HIV protease inhibitor, peptidase inhibitor
1110	mafenide	carbonic anhydrase inhibitor, unidentified pharmacological activity
1111	profenamine	butyrylcholinesterase inhibitors, cholinergic receptor antagonist, glutamate receptor antagonist
1112	mofezolac	cyclooxygenase inhibitor, cytochrome P450 inhibitor, platelet aggregation inhibitor
1113	iso-olomoucine	cyclin-related kinase inhibitor
1114	noretynodrel	progestogen steroid hormone
1115	triamcinolone	antiinflammatory agent, corticosteroid agonist, corticosteroid binding globulin binder, corticosteroid hormone receptor agonist, cytochrome P450 inhibitor, glucocorticoid receptor agonist, immunosuppressant, immunosuppressi[illegible]
1116	bromfenac	cyclooxygenase inhibitor
1117	FPL-64176	calcium channel agonist, L-type calcium channel activator
1118	J-104129	acetylcholine receptor antagonist
1119	PNU-96415E	dopamine receptor antagonist
1120	clomipramine	serotonin transporter (SERT) inhibitor, dopamine transporter (DAT) inhibitor, norepinephrine transporter (NET) inhibitor
1121	rifampicin	DNA directed RNA polymerase inhibitor, enzyme inducer
1122	pancuronium	acetylcholine receptor antagonist
1123	SB-203186	serotonin receptor antagonist
1124	NSC-693868	CDK inhibitor, glycogen synthase kinase inhibitor
1125	diltiazem	L-type calcium channel blocker, calcium channel antagonist, calcium channel blocker
1126	carvedilol	adrenergic receptor antagonist, reducing agent, ryanodine receptor channel modulator
1127	cotinine	nicotine metabolite
1128	miglitol	glucosidase inhibitor, sodium/glucose cotransporter activator
1129	BRD-K12401458	lysophospholipid receptor agonist
1130	MDL-73005EF	serotonin receptor antagonist, serotonin receptor partial agonist
1131	limonin	HIV protease inhibitor
1132	mianserin	serotonin receptor antagonist, tetracyclic antidepressant
1133	lavendustin-c	epidermal growth factor receptor (EGFR) inhibitor
1134	nalbuphine	opioid receptor agonist, opioid receptor antagonist
1135	dipyridamole	phosphodiesterase inhibitor, cyclooxygenase inhibitor, platelet aggregation inhibitor
1136	DUP-697	cyclooxygenase inhibitor
1137	abiraterone	17,20 lyase inhibitor, androgen biosynthesis inhibitor, cytochrome P450 inhibitor, steroid sulfatase inhibitor
1138	CNQX	glutamate receptor antagonist, unidentified pharmacological activity

1139	tocainide	sodium channel blocker, voltage-gated sodium channel blocker
1140	budesonide	glucocorticoid receptor agonist, glucocordicoid receptor antagonist, immunosuppressant
1141	otenzepad	acetylcholine receptor antagonist
1142	thiorphan	membrane metalloendopeptidase inhibitor
1143	felodipine	calcium channel blocker, L-type calcium channel blocker
1144	testosterone	androgen receptor (AR) agonist, androgen receptor agonist, testosterone receptor agonist
1145	MDL-28170	calpain inhibitor, cathepsin inhibitor
1146	DAU-5884	acetylcholine receptor antagonist
1147	2-(4-methoxybenzylthio)-6-methylpyrimidin-4-ol	matrix metalloprotease inhibitor
1148	ritonavir	HIV protease inhibitor, cytochrome P450 inhibitor
1149	DCEBIO	intermediate conductance potassium channel activator
1150	nomegestrol	progesterone receptor agonist
1151	beclometasone	glucocorticoid receptor agonist, corticosteroid hormone receptor agonist, immunosuppressant
1152	SB-216763	glycogen synthase kinase inhibitor
1153	crotamiton	scabicidal, antipruritic
1154	brimonidine	adrenergic receptor agonist
1155	BH3I-1	BCL inhibitor
1156	amoxapine	adrenergic transmitter uptake inhibitor, dopamine receptor, norepinephrine reputake inhibitor, serotonin receptor antagonist, serotonin reuptake inhibitor, serotonin uptake inhibitor, tricyclic antidepressant
1157	BRD-K66896231	acetylcholinesterase inhibitor
1158	ginsenoside	steroidal receptor agonist
1159	fluvoxamine	selective serotonin reuptake inhibitor (SSRI), serotonin reuptake inhibitor, serotonin uptake inhibitor
1160	talipexole	adrenergic receptor agonist, dopamine receptor agonist, dopamine autoreceptor agonist, serotonin receptor antagonist
1161	amcinonide	corticosteroid agonist, cytochrome P450 inhibitor, glucocorticoid receptor agonist, immunosuppressant
1162	PSB-11	adenosine receptor antagonist
1163	SKF-81297	dopamine receptor agonist
1164	riluzole	glutamate release inhibitor, dopamine receptor agonist, glutamate receptor antagonist, potassium channel blocker, Sodium Channel Blockers, sodium channel blocker
1165	valaciclovir	DNA directed DNA polymerase inhibitor, DNA polymerase inhibitor
1166	ropivacaine	membrane integrity inhibitor, potassium channel antagonist, sodium channel blocker
1167	dicycloverine	acetylcholine receptor antagonist, anticholinergic
1168	icariin	neuromedin receptor agonist, sorbitol dehydrogenase inhibitor
1169	AG-490	epidermal growth factor receptor (EGFR) inhibitor, ErbB2 and JAK2 inhibitor, JAK inhibitor
1170	cimetidine	histamine receptor antagonist, histamine receptor inhibitor
1171	bepridil	calcium channel blocker, L-type calcium channel blocker
1172	ICI-118551	adrenergic receptor antagonist
1173	lobendazole	anthelmintic agent
1174	levocetirizine	histamine receptor antagonist, interleukin expression inhibitor, tumor necrosis factor expression inhibitor
1175	BRD-K27074404	solute carrier family member inhibitor
1176	naringenin	aromatase inhibitor, estrogen receptor agonist, quorum sensing signaling modulator, TRPM3 antagonist
1177	quinapril	angiotensin converting enzyme inhibitor
1178	zeranol	estrogen receptor agonist
1179	toltrazuril	anti-protozoal
1180	carbidopa	aromatic L-amino acid decarboxylase inhibitor, dopa decarboxylase (aromatic L-amino acid decarboxylase) inhibitor
1181	midodrine	adrenergic receptor agonist
1182	glipizide	sulfonylurea, ATP channel blocker, ATP-sensitive potassium channel antagonist, insulin secretagogue
1183	pravastatin	HMGCR inhibitor
1184	ampiroxicam	cyclooxygenase inhibitor
1185	L-165041	PPAR receptor agonist
1186	mefexamide	psychostimulant
1187	6-benzylaminopurine	purinergic receptor activator
1188	ipsapirone	serotonin receptor agonist
1189	probucol	atherogenesis inhibitor, cholesterol inhibitor, reducing agent
1190	zafirlukast	leukotriene receptor antagonist
1191	desipramine	norepinephrine transporter inhibitor, Sodium Channel Blockers, tricyclic antidepressant (TCA)
1192	warfarin	cytochrome P450 inhibitor, vitamin inhibitor, vitamin K epoxide reductase inhibitor
1193	thiethylperazine	dopamine receptor antagonist
1194	M-3M3FBS	phospholipase activator
1195	BRL-52537	opioid receptor agonist
1196	mosapride	serotonin receptor agonist, serotonin receptor antagonist
1197	dioxybenzone	topical sunscreen agent
1198	dehydrocholic-acid	gastrointestinal agent that stimulates bile production or the flow of bile into the duodenum
1199	ascorbyl-palmitate	antioxidant
1200	indapamide	carbonic anhydrase inhibitor, thiazide diuretic
1201	mepyramine	histamine receptor antagonist
1202	ritonavir	HIV protease inhibitor, cytochrome P450 inhibitor
1203	ondansetron	serotonin receptor antagonist
1204	E-4031	polarization inhibitor, potassium channel antagonist, potassium channel blocker
1205	proadifen	nitric oxide synthase inhibitor, cytochrome P450 inhibitor, potassium channel blocker
1206	tosyllysyl-chloromethyl-ketone	chymotrypsin inhibitor
1207	trimipramine	adrenergic transmitter uptake inhibitor, dopamine receptor, norepinephrine transporter inhibitor, serotonin transporter (SERT) inhibitor, serotonin uptake inhibitor, tricyclic antidepressant, tricyclic antidepressant (TCA)
1208	methysergide	serotonin receptor antagonist
1209	picrotoxin	GABA receptor antagonist
1210	piribedil	dopamine receptor agonist, adrenergic receptor antagonist
1211	RU-28318	cytochrome P450 antagonist
1212	alfaxalone	11-beta hydroxysteroid dehydrogenase inhibitor, chloride channel agonist, general pump inhibitor
1213	naproxol	antiinflammatory agent
1214	nor-binaltorphimine	opioid receptor antagonist
1215	meclozine	constitutive androstane receptor (CAR) agonist

1216	VU-0366037-2	glutamate receptor modulator
1217	diflunisal	cyclooxygenase inhibitor, prostanoid receptor inhibitor
1218	hydrocotarnine	non-narcotic opium alkaloid used in cancer pain treatment
1219	nifedipine	calcium channel blocker, L-type calcium channel blocker
1220	androsta-1,4-dien-3,17-dione	aromatase inhibitor
1221	procarbazine	DNA alkylating drug, transmethylation inhibitor
1222	racecadotril	enkephalinase inhibitor, membrane metalloendopeptidase inhibitor, neprilysin inhibitor
1223	pifithrin-alpha	TP53 inhibitor
1224	DH-97	melatonin receptor antagonist
1225	ethinylestradiol	DNA directed DNA polymerase stimunlant, estrogen receptor agonist
1226	repaglinide	insulin secretagogue, ATP-sensitive potassium channel antagonist, cytochrome P450 inhibitor, meglitinide
1227	cefixime	cell wall synthesis inhibitor
1228	siguazodan	histamine receptor antagonist, phosphodiesterase inhibitor
1229	CGP-57380	MAP kinase inhibitor, MAPK-interacting kinase inhibitor
1230	6-aminochrysene	inhibitor of transferase activity
1231	SANT-1	smoothened receptor antagonist
1232	nifurtimox	DNA inhibitor
1233	stiripentol	GABA aminotransferase inhibitor, GABA reuptake inhibitor, sodium channel blocker
1234	tolcapone	catechol O methyltransferase inhibitor, transthyretin amyloid inhibitor
1235	tangeritin	cell cycle inhibitor
1236	flunarizine	calcium channel blocker, T-type calcium channel blocker
1237	disopyramide	voltage-gated sodium channel blocker
1238	dihydro-7-desacetyldeoxygedunin	HSP90 inhibitor
1239	VU-0413807-2	calcium channel blocker
1240	dihydroxyphenylglycine	glutamate receptor agonist
1241	erismodegib	hedgehog pathway inhibitor, smoothened receptor antagonist
1242	doxapram	potassium channel blocker
1243	SB-258585	serotonin receptor antagonist
1244	xanthinol	vasodilator
1245	MK-212	serotonin receptor agonist
1246	BRD-A73929928	adenosine receptor agonist
1247	BRD-K52640952	topoisomerase inhibitor
1248	lobaric-acid	tyrosine phosphatase inhibitor
1249	norgestrel	progesterone receptor agonist
1250	SDZ-WAG-994	adenosine receptor agonist
1251	N6-cyclopentyladenosine	adenosine receptor agonist
1252	pirenperone	serotonin receptor antagonist
1253	GR-89696	opioid receptor agonist
1254	acepromazine	dopamine receptor antagonist
1255	gabexate	serine protease inhibitor, AP inhibitor, NFkB pathway inhibitor, peptidase inhibitor, protease inhibitor
1256	minaprine	serotonin uptake inhibitor
1257	D-4476	casein kinase inhibitor, TGF beta receptor inhibitor
1258	calcitriol	vitamin D receptor agonist, hypercalcaemic agent
1259	anastrozole	aromatase inhibitor
1260	levomepromazine	dopamine receptor
1261	BRD-K32656671	mannose-6-phosphate isomerate inhibitor
1262	laudanosine	central nervous system agent
1263	ketotifen	histamine receptor ligand, leukotriene antagonist, phosphodiesterase inhibitor
1264	ZK-756326	CC chemokine receptor ligand
1265	acadesine	adenosine release stimulant, AMPK activator, AMPK stimulant
1266	rolitetracycline	antibiotic
1267	BRD-K06817181	JAK inhibitor
1268	norethindrone	ovulation inhibitor, progesterone receptor agonist
1269	doxycycline	metalloproteinase inhibitor, 30S ribosomal protein inhibitor, matrix metalloprotease inhibitor, protein arginine deiminase inhibitor
1270	loxapine	dopamine receptor antagonist, serotonin receptor antagonist, dopamine receptor ligand
1271	cortisone	glucocorticoid receptor agonist
1272	nifedipine	calcium channel blocker, L-type calcium channel blocker
1273	MAPP-D-erythro	ceramidase inhibitor
1274	BRD-K21009077	dual specificity protein phosphatase inhibitors
1275	tacrine	acetylcholinesterase inhibitor, acetylcholine release stimulant, butyrylcholinesterase inhibitors, potassium channel antagonist
1276	ifenprodil	adrenergic receptor antagonist, glutamate receptor antagonist
1277	dextromethorphan	glutamate receptor antagonist, sigma receptor agonist
1278	noscapine	bradykinin receptor antagonist, tubulin polymerization inhibitor, apoptosis stimulant, microtubule inhibitor, tubulin inhibitor
1279	L-689560	glutamate receptor antagonist
1280	bendroflumethiazide	sodium/potassium/chloride transporter inhibitor, thiazide diuretic
1281	dextromethorphan	glutamate receptor antagonist, sigma receptor agonist
1282	SA-94315	caspase inhibitor
1283	SC-9	protein kinase C activator
1284	fluvoxamine	selective serotonin reuptake inhibitor (SSRI), serotonin reuptake inhibitor, serotonin uptake inhibitor
1285	pidotimod	interferon receptor agonist, interleukin receptor agonist, T cell stimulant
1286	brompheniramine	histamine receptor antagonist
1287	liothyronine	thyroid hormone function stimulant
1288	dipivefrine	adrenergic receptor agonist
1289	apigenin	casein kinase inhibitor, cell proliferation inhibitor, cytochrome P450 inhibitor, GABA receptor antagonist, glutamate receptor antagonist, monoamine oxidase inhibitor, nitric oxide production inhibitor, ornithine decarboxylase inhil
1290	benidipine	calcium channel blocker, L-type calcium channel blocker
1291	tubaic-acid	mitochondrial complex I inhibitor, NADH-ubiquinone oxidoreductase (Complex I) inhibitor
1292	bullevaconitine-a	non-opiod analgesic

1292	bulleyaconitine-a	non-opioid analgesic
1293	norethisterone	ovulation inhibitor
1294	KI-16425	lysophosphatidic acid receptor antagonist
1295	H-9	cAMP dependent protein kinase inhibitor, PKA inhibitor
1296	amylocaine	local anesthetic
1297	DR-2313	PARP inhibitor
1298	LY-288513	CCK receptor antagonist
1299	L-152804	neuropeptide receptor antagonist
1300	azathioprine	dehydrogenase inhibitor, immunosuppressant, purine antagonist
1301	doxepin	histamine receptor antagonist, adrenergic transmitter uptake inhibitor, histamine receptor inhibitor, norepinephrine reputake inhibitor, serotonin reuptake inhibitor, tricyclic antidepressant
1302	BRD-K26947839	NADPH oxidase inhibitor
1303	BW-723C86	serotonin receptor agonist
1304	KU-14R	pancreatic β-cell I3 binding site antagonist
1305	tizanidine	adrenergic receptor agonist
1306	icilin	TRPA1 agonist, TRPM8 agonist
1307	norcyclobenzaprine	adrenergic receptor ligand, serotonin receptor ligand
1308	talniflumate	cyclooxygenase inhibitor, calcium channel blocker, calcium-activated chloride channel blocker, mucin production inhibitor
1309	BRD-K54331210	alkaline phosphatase inhibitor
1310	NAN-190	serotonin receptor agonist
1311	sirolimus	mTOR inhibitor, CCR expression inhibitor, cell cycle inhibitor, proteasome inhibitor, protein kinase inhibitor, T cell inhibitor
1312	prestwick-559	dopamine receptor agonist
1313	BRD-K98948170	kelch-like ECH-associated protein ligand
1314	sumatriptan	serotonin receptor agonist
1315	mepacrine	NFkB pathway inhibitor, cytokine production inhibitor, TP53 expression enhancer, acetylcholinesterase inhibitor, AKT inhibitor, apoptosis stimulant, DNA inhibitor, mTOR inhibitor, phospholipase inhibitor, PI3K inhibitor, secretory
1316	trimetozine	compound with mild tranquilizing effects
1317	megestrol	progesterone receptor agonist, DNA inhibitor, HegG2 inhibitor
1318	DY-131	estrogen-related receptor agonist
1319	kawain	calcium channel modulator, mTOR inhibitor, Sodium Channel Blockers
1320	triptolide	RNA polymerase inhibitor
1321	fluocinonide	corticosteroid agonist, corticosteroid hormone receptor agonist
1322	hydroxycholesterol	LXR agonist, ABC transporter expression enhancer, alpha secretase activator, beta secretase inhibitor, glutamate receptor modulator
1323	tyrphostin-AG-82	EGFR inhibitor, epidermal growth factor receptor (EGFR) inhibitor, tyrosine kinase inhibitor
1324	L-368899	oxytocin receptor antagonist
1325	ML-3163	p38 MAPK inhibitor
1326	guaifenesin	expectorant
1327	methylergometrine	dopamine receptor antagonist, partial serotonin receptor agonist
1328	BRD-K77690805	pyruvate kinase isozyme activator
1329	rhapontin	apoptosis inducer
1330	etomidate	membrane integrity inhibitor, membrane permeability inhibitor
1331	chlorphenamine	histamine receptor antagonist
1332	pargyline	monoamine oxidase inhibitor
1333	bromocriptine	dopamine receptor agonist, dopamine receptor antagonist, prolactin secretion inhibitor
1334	flupentixol	dopamine receptor antagonist
1335	ARC-239	adrenergic receptor antagonist
1336	huperzine-a	acetylcholinesterase inhibitor, glutamate receptor antagonist
1337	nafadotride	dopamine receptor antagonist
1338	telmisartan	angiotensin receptor antagonist, PPAR receptor agonist, PPAR receptor modulator, PPAR receptor partial agonist
1339	BRD-K36038115	neuropeptide receptor antagonist
1340	metergoline	dopamine receptor agonist, serotonin receptor antagonist, prolactin inhibitor
1341	andarine	androgen receptor modulator
1342	4,5-dianilinophthalimide	EGFR inhibitor, epidermal growth factor receptor (EGFR) inhibitor
1343	diloxanide	unidentified pharmacological activity
1344	bumetanide	diuretic, solute carrier family member inhibitor
1345	enrofloxacin	topoisomerase inhibitor
1346	hydrocortisone	corticosteroid agonist, glucocorticoid receptor agonist, immunosuppressant, interleukin receptor antagonist
1347	U-46619	thromboxane receptor agonist
1348	atomoxetine	norepinephrine transporter inhibitor, adrenergic transmitter uptake inhibitor
1349	baccatin-III	paclitaxel precursor
1350	rifabutin	DNA directed DNA polymerase inhibitor, DNA directed RNA polymerase inhibitor, protein synthesis inhibitor
1351	oxaprozin	cyclooxygenase inhibitor
1352	progesterone	progesterone receptor agonist
1353	bupropion	dopamine reuptake inhibitor, adrenergic transmitter uptake inhibitor, dopamine transporter inhibitor, norepinephrine transporter inhibitor, norepinephrine/dopamine dual reuptake inhibitor
1354	dydrogesterone	progesterone receptor agonist
1355	biperiden	anticholinergic
1356	hexamethylenebisacetamide	differentiation inducer, NFKB inhibitor, AKT inhibitor
1357	chlortalidone	carbonic anhydrase inhibitor, diuretic, sodium ion tranport inhibitor
1358	mercaptopurine	immunosuppressant, protein synthesis inhibitor, purine antagonist
1359	cilastatin	dehydropeptidase inhibitor
1360	goserelin	gonadotropin releasing factor hormone receptor agonist, luteinizing hormone releasing hormone agonist
1361	hesperidin	free radical scavenger
1362	canrenoic-acid	aldosterone antagonist
1363	glibenclamide	sulfonylurea, ATP channel blocker, ATP-sensitive potassium channel antagonist, biguanide, gluconeogenesis inhibitor, insulin secretagogue, insulin sensitizer, TRPA1 agonist
1364	CAY-10415	insulin sensitizer
1365	docosatrienoic-acid	inhibitor of LTB4 binding to neutrophils
1366	vincamine	adrenergic receptor antagonist
1367	GW-501516	PPAR receptor agonist, insulin sensitizer
1368	gabazine	GABA receptor antagonist
1369	thiotepa	cytochrome P450 inhibitor

1369	thiotepa	cytochrome P450 inhibitor
1370	piretanide	diuretic, glucocorticoid receptor agonist
1371	CGP-7930	GABA receptor modulator, GABA receptor positive allosteric modulator
1372	mesna	sulfhydryl compound that inactivates toxic metabolites of some chemotherapeutics
1373	nateglinide	insulin secretagogue, ATP-sensitive potassium channel antagonist, meglitinide, sulphonylurea receptor activator
1374	pioglitazone	PPAR receptor agonist, insulin sensitizer, adiponectin expression enhancer, thiazolidinedione
1375	phentolamine	adrenergic receptor antagonist
1376	GR-135531	melatonin receptor agonist
1377	YS-035	calcium channel blocker
1378	butabindide	tripeptidyl peptidase inhibitor
1379	hymecromone	monoamine oxidase inhibitor
1380	moxonidine	imidazoline receptor agonist, adrenergic receptor agonist
1381	U-99194	dopamine receptor antagonist
1382	diclofenac	cyclooxygenase inhibitor
1383	acetohydroxamic-acid	inhibitor of bacterial and plant urease
1384	methapyrilene	histamine receptor antagonist
1385	betahistine	histamine receptor agonist, histamine receptor antagonist
1386	azasetron	serotonin receptor antagonist
1387	EHNA	adenosine deaminase inhibitor
1388	propafenone	antiarrhythmic medication, potassium channel blocker, voltage-gated sodium channel blocker
1389	risperidone	dopamine receptor antagonist, serotonin receptor antagonist, adrenergic receptor antagonist
1390	NAS-181	serotonin receptor antagonist
1391	quipazine	serotonin receptor agonist
1392	altanserin	serotonin receptor antagonist, collagen stimulant
1393	betulinic-acid	apoptosis stimulant, caspase activator, diacylglycerol O acyltransferase inhibitor, HIV integrase inhibitor, NFkB pathway activator, NFKB pathway inhibitor, SARS coronavirus 3C-like protease inhibitor, topoisomerase inhibitor
1394	citalopram	serotonin reuptake inhibitor, serotonin uptake inhibitor
1395	phenanthridone	PARP inhibitor
1396	reboxetine	adrenergic receptor antagonist, adrenergic transmitter uptake inhibitor, norepinephrine reputake inhibitor, norepinephrine transporter inhibitor
1397	azaperone	dopamine receptor antagonist
1398	pilocarpine	acetylcholine receptor agonist
1399	DPPE	histamine receptor antagonist
1400	fluticasone	glucocorticoid receptor agonist
1401	famotidine	histamine receptor antagonist
1402	escitalopram	selective serotonin reuptake inhibitor (SSRI), serotonin reuptake inhibitor
1403	meclofenamic-acid	cyclooxygenase inhibitor, prostanoid receptor antagonist
1404	estropipate	estrogen receptor agonist
1405	homochlorcyclizine	antihistamine
1406	ethylestrenol	anabolic steroid, has some progesterone-like activity
1407	febuxostat	xanthine oxidase inhibitor
1408	mexiletine	sodium channel blocker, potassium channel blocker
1409	naloxone	opioid receptor antagonist
1410	noscapine	bradykinin receptor antagonist, tubulin polymerization inhibitor, apoptosis stimulant, microtubule inhibitor, tubulin inhibitor
1411	PSB-1115	adenosine receptor antagonist
1412	phentermine	adrenergic transmitter uptake inhibitor, dopamine transmitter uptake inhibitor, serotonin uptake inhibitor
1413	K3644	Kinesin-Like Spindle Protein KIF11 (KSP, Eg5) Inhibitors, mitotic kinesin inhibitor
1414	PNU-282987	acetylcholine receptor antagonist
1415	alclometasone	glucocorticoid receptor agonist, immunosuppressant
1416	LY-83583	inhibitor of soluble guanylyl cyclase, leukotriene synthesis inhibitor
1417	nitrazepam	GABA benzodiazepine site receptor agonist
1418	hexylcaine	sodium channel blocker
1419	clobetasol	glucocorticoid receptor agonist
1420	isoxsuprine	adrenergic receptor agonist
1421	etodolac	cyclooxygenase inhibitor, TRPV agonist
1422	glycocholic-acid	cholesterol inhibitor
1423	piroxicam	cyclooxygenase inhibitor
1424	guggulsterone	estrogen receptor agonist, FXR antagonist, progesterone receptor agonist, cholesterol inhibitor, IKK inhibitor, PXR agonist
1425	tolmetin	cyclooxygenase inhibitor
1426	H-8	PKA inhibitor
1427	benzo(a)pyrene	pro carcinogen that is metabolized to the DNA intercalating agent benzo(a)pyrene diol epoxide
1428	prochlorperazine	dopamine receptor antagonist, dopamine receptor
1429	genipin	aglycone, nitric oxide production inhibitor
1430	z-prolyl-prolinal	prolyl endopeptidase inhibitor
1431	decitabine	DNA methyltransferase inhibitor, antimetabolite, DNA methylase inhibitor
1432	DMBI	platelet-derived growth factor receptor (PDGFR) inhibitor, tyrosine kinase inhibitor, vascular endothelial growth factor receptor (VEGFR) inhibitor
1433	bicuculline	GABA receptor antagonist
1434	domperidone	dopamine receptor antagonist, breast cancer resistance protein inhibitor
1435	ketoprofen	cyclooxygenase inhibitor
1436	dihydrexidine	dopamine receptor agonist
1437	diazepam	benzodiazepine receptor agonist, GABA benzodiazepine site receptor agonist
1438	kynuramine	aryl hydrocarbon receptor activator
1439	benzthiazide	carbonic anhydrase inhibitor
1440	olmesartan	angiotensin receptor antagonist, angiotensin antagonist
1441	aprepitant	tachykinin antagonist
1442	QX-222	sodium channel blocker
1443	EO-1428	p38 MAPK inhibitor
1444	BRD-K56658166	N-type calcium channel blocker, Sodium Channel Blockers
1445	rosmarinic-acid	antioxidant
[illegible]	[illegible]	[illegible]

1446	HSP90-inhibitor	HSP inhibitor, HSP antagonist
1447	erythromycin	NFkB pathway inhibitor, 50S ribosomal subunit inhibitor, motilin receptor agonist, RPLV inhibitor
1448	PD-173074	FGFR inhibitor, VEGFR inhibitor
1449	SCH-442416	adenosine receptor antagonist
1450	dibutyrylcyclic-gmp	cGMP analog
1451	alfuzosin	adrenergic receptor antagonist
1452	ambelline	plant alkaloid
1453	dictamnine	furoquinoline alkaloid that causes smooth muscle contraction
1454	BRD-K91691979	glucosylceramidase inhibitor
1455	ranitidine	histamine receptor antagonist
1456	TC-2559	acetylcholine receptor agonist, acetylcholine receptor ligand
1457	REV-5901	leukotriene receptor antagonist, lipoxygenase inhibitor
1458	methimazole	antithyroid Drugs
1459	PSB-06126	NTPDase inhibitor
1460	fenofibrate	PPAR receptor agonist, apolipoprotein expression enhancer, lipase clearing factor inhibitor
1461	naloxone	opioid receptor antagonist
1462	cilostazol	phosphodiesterase inhibitor, platelet aggregation inhibitor
1463	diazoxide	ATP channel activator, potassium channel activator
1464	NCS-382	GABA receptor antagonist
1465	SU-4312	platelet-derived growth factor receptor (PDGFR) inhibitor, tyrosine kinase inhibitor, vascular endothelial growth factor receptor (VEGFR) inhibitor, vascular endothelial growth factor receptor 2 (VEGFR2) inhibitor
1466	daidzein	estrogen receptor agonist
1467	benzbromarone	calcium-activated chloride channel blocker, uric acid diuretic
1468	cinanserin	serotonin receptor antagonist
1469	SYK-inhibitor	syk inhibitor
1470	GW-0742	PPAR receptor agonist, insulin sensitizer
1471	oxantel	fumarate reductase inhibitor
1472	heraclenol	furocoumarin compound with antiproliferative activity at G2/M phase
1473	indinavir	HIV protease inhibitor
1474	epitestosterone	inactive testosterone analog
1475	L-732138	tachykinin antagonist
1476	dipropyl-5ct	serotonin receptor agonist
1477	argatroban	thrombin inhibitor, peptidase inhibitor, protease inhibitor
1478	hippeastrine	plant alkaloid with cytotoxic effects on some human tumor cell lines
1479	BMY-7378	serotonin receptor antagonist, adrenergic receptor antagonist
1480	propentofylline	acetylcholinesterase inhibitor, adenosine reuptake inhibitor, adenosine uptake inhibitor, phosphodiesterase inhibitor
1481	piceid	glucosidase inhibitor, ICAM1 expression inhibitor, VCAM expression inhibitor, xanthine oxidase inhibitor
1482	tyrphostin-AG-825	receptor tyrosine protein kinase inhibitor
1483	SB-269970	serotonin receptor antagonist
1484	ethisterone	androgen receptor agonist
1485	huperzine-a	acetylcholinesterase inhibitor, glutamate receptor antagonist
1486	gavestinel	glutamate receptor antagonist, glycine receptor antagonist
1487	OBAA	phospholipase inhibitor
1488	IRL-2500	endothelin receptor antagonist
1489	rilmenidine	adrenergic receptor agonist, imidazoline receptor agonist
1490	formoterol	adrenergic receptor agonist
1491	tubocurarine	acetylcholine receptor antagonist
1492	morphothebaine	adrenergic receptor inhibitor
1493	edrophonium	acetylcholinesterase inhibitor
1494	ciprofibrate	PPAR receptor agonist, lipase clearing factor inhibitor
1495	WAY-161503	serotonin receptor agonist
1496	cefotiam	cell wall synthesis inhibitor
1497	osthol	ACAT inhibitor, GABA receptor modulator
1498	BRD-A61189834	XIAP inhibitor
1499	L-741626	dopamine receptor, dopamine receptor antagonist
1500	HG-5-113-01	protein kinase inhibitor
1501	methyllidocaine	antiarrhythmic, phosphatidyl-inositol activator
1502	ODQ	guanylyl cyclase inhibitor, inhibitor of soluble guanylyl cyclase, potassium channel blocker
1503	SB-202190	p38 MAPK inhibitor, interleukin inhibitor, stress activated protein kinase inhibitor
1504	estradiol-valerate	estrogen receptor agonist
1505	RO-15-4513	GABA benzodiazepine site receptor inverse agonist
1506	prednisone	glucocorticoid receptor agonist
1507	zoxazolamine	IKCa channel activator
1508	metoprolol	adrenergic receptor antagonist
1509	pefloxacin	topoisomerase inhibitor
1510	NNC-711	GAT inhibitor
1511	hydrocortisone	corticosteroid agonist, glucocorticoid receptor agonist, immunosuppressant, interleukin receptor antagonist
1512	DNQX	glutamate receptor antagonist
1513	mitomycin-c	DNA alkylating drug, DNA inhibitor, DNA synthesis inhibitor
1514	pinacidil	ATP channel activator, potassium channel agonist
1515	tetryzoline	adrenergic receptor agonist
1516	GR-55562	serotonin receptor antagonist
1517	ticlopidine	purinergic receptor antagonist, platelet aggregation inhibitor
1518	RO-16-6941	monoamine oxidase inhibitor
1519	ormetoprim	antibiotic
1520	lamotrigine	glutamate release inhibitor, serotonin receptor inhibitor, Sodium Channel Blockers, sodium channel blocker, voltage-gated sodium channel blocker
1521	EXO-1	ARF inhibitor
1522	fexofenadine	histamine receptor antagonist

1523	gibberellic-acid	NFkB pathway modulator
1524	dapsone	dihydrofolate reductase inhibitor, dihydropteroate synthase inhibitor, FOLP inhibitor
1525	tubaic-acid	mitochondrial complex I inhibitor, NADH-ubiquinone oxidoreductase (Complex I) inhibitor
1526	solifenacin	acetylcholine receptor antagonist
1527	deracoxib	cyclooxygenase inhibitor
1528	fananserin	dopamine receptor antagonist, serotonin receptor antagonist
1529	hydroquinidine	anti-arrhythmia agent
1530	hydrocortisone	corticosteroid agonist, glucocorticoid receptor agonist, immunosuppressant, interleukin receptor antagonist
1531	enalaprilat	angiotensin converting enzyme inhibitor
1532	bemegride	chemoreceptor agonist
1533	zamifenacin	acetylcholine receptor antagonist
1534	fenpiverinium	acetylcholine receptor antagonist
1535	proxyfan	histamine receptor modulator
1536	BRD-K01265221	BCL inhibitor
1537	NS-1619	calcium channel activator, large conductance potassium channel activator
1538	imiloxan	adrenergic receptor antagonist
1539	azauridine	antiviral agent, blocks the conversion of orotic acid into UMP
1540	nalbuphine	opioid receptor agonist, opioid receptor antagonist
1541	diclofenamide	carbonic anhydrase inhibitor
1542	thenoyltrifluoroacetone	PPAR receptor ligand
1543	secoisolariciresinol	antioxidant
1544	YK-4279	apoptosis stimulant, binding of RNA helicase A to the transcription factor EWS-FLI1 inhibitor, EWS-FLI1 inhibitor
1545	bongkrek-acid	mitochondrial ADP, ATP translocase inhibitor
1546	pinacidil	ATP channel activator, potassium channel agonist
1547	mometasone	glucocorticoid receptor agonist, immunosuppressant
1548	SIB-1757	glutamate receptor antagonist
1549	tropisetron	serotonin receptor antagonist, polarization inhibitor, sodium channel blocker
1550	pitavastatin	HMGCR inhibitor, apolipoprotein expression enhancer, osteopontin expression inhibitor
1551	lestaurtinib	FLT3 inhibitor, growth factor receptor inhibitor, JAK inhibitor, apoptosis stimulant
1552	gemfibrozil	lipase clearing factor inhibitor, lipoprotein lipase activator, PPAR receptor agonist
1553	galantamine	acetylcholinesterase inhibitor, acetylcholine receptor agonist, butyrylcholinesterase inhibitors
1554	rescinnamine	angiotensin converting enzyme inhibitor
1555	androstanol	constitutive androstane receptor (CAR) inhibitor
1556	flufenamic-acid	calcium activated chloride channel inhibitor
1557	dibenzepin	norepinephrine reuptake inhibitor
1558	JAK3-inhibitor-I	JAK inhibitor
1559	flunarizine	calcium channel blocker, T-type calcium channel blocker
1560	salbutamol	adrenergic receptor agonist
1561	W-12	calmodulin antagonist
1562	ebselen	COX inihibitor, H+/K+-ATPase inhibitor, leukotriene receptor antagonist, NADPH oxidase inhibitor, nitric oxide synthase inhibitor, PKC inhibitor, prostanoid receptor antagonist, protein kinase C inhibitor, reducing agent
1563	marmesin	precursor in psoralen
1564	CAY-10577	casein kinase inhibitor
1565	loxoprofen	cyclooxygenase inhibitor, platelet aggregation inhibitor, prostanoid receptor inhibitor
1566	LY-165163	serotonin receptor antagonist
1567	levofloxacin	topoisomerase inhibitor, DNA gyrase inhibitor
1568	W-5	calmodulin antagonist
1569	SKF-83566	dopamine receptor antagonist
1570	SU-4312	platelet-derived growth factor receptor (PDGFR) inhibitor, tyrosine kinase inhibitor, vascular endothelial growth factor receptor (VEGFR) inhibitor, vascular endothelial growth factor receptor 2 (VEGFR2) inhibitor
1571	droperidol	dopamine receptor antagonist, dopamine receptor
1572	metixene	acetylcholine receptor antagonist
1573	ipratropium	acetylcholine receptor antagonist
1574	lovastatin	HMGCR inhibitor
1575	eicosadienoic-acid	in touchstone
1576	carbenoxolone	11-beta hydroxysteroid dehydrogenase inhibitor
1577	triamterene	sodium channel blocker
1578	phenazopyridine	unidentified pharmacological activity
1579	guanabenz	adrenergic receptor agonist
1580	trap-101	nociceptin/orphanin FQ (NOP) receptor antagonist, opioid receptor antagonist
1581	FGIN-1-27	benzodiazepine receptor agonist, inositol monophosphatase inhibitor
1582	OBAA	phospholipase inhibitor
1583	pazufloxacin	topoisomerase inhibitor
1584	BRD-K41143549	glutamate receptor antagonist
1585	salicin	antiinflammatory agent
1586	BRD-K34608650	cannabinoid receptor agonist
1587	valsartan	angiotensin receptor antagonist
1588	flucytosine	pyrimidine analog, inhibits fungal DNA synthesis
1589	venlafaxine	serotonin reuptake inhibitor, adrenergic transmitter uptake inhibitor, norepinephrine reuptake inhibitor, norepinephrine reputake inhibitor, serotonin uptake inhibitor
1590	yohimbine	adrenergic receptor antagonist
1591	dantrolene	calcium channel antagonist
1592	benzamil	epithelial sodium channel blocker
1593	depudecin	HDAC inhibitor
1594	catechin	beta secretase inhibitor, fatty acid synthase inhibitor, free radical scavenger, immunostimulant, LDL antioxidants, quorum sensing signaling modulator, reducing agent, sodium channel blocker
1595	ergocryptine	dopamine agonist
1596	prostaglandin-b2	cAMP inhibitor
1597	Y-26763	potassium channel activator, potassium channel agonist
1598	zacopride	serotonin receptor antagonist, serotonin receptor agonist
1599	lisuride	dopamine receptor agonist, prolactin inhibitor, serotonin receptor antagonist, serotonin receptor ligand

[illegible]	lisuride	dopamine receptor agonist, prolactin inhibitor, serotonin receptor antagonist, serotonin receptor ligand
1600	cefazolin	cell wall synthesis inhibitor
1601	artemether	antimalarial, interacts with heme and with COX3
1602	phenformin	AMPK activator
1603	RS-45041-190	imidazoline receptor agonist
1604	nomilin	HSP inhibitor
1605	chlortetracycline	protein synthesis inhibitor
1606	buspirone	serotonin receptor agonist, serotonin receptor partial agonist
1607	rutin	aldose reductase inhibitor, antioxidant, capillary stabilizing agent, nitric oxide scavenger, prolyl 4-hydroxylase inhibitor
1608	tenidap	cyclooxygenase inhibitor, lipoxygenase inhibitor, prostanoid receptor antagonist
1609	1-benzylimidazole	thromboxane synthase inhibitor
1610	nicotine	acetylcholine receptor agonist
1611	BRD-K14048378	lysophospholipid receptor agonist
1612	SB-334867	orexin receptor antagonist
1613	tramadol	opioid receptor agonist, norepinephrine reputake inhibitor, serotonin reuptake inhibitor, adrenergic transmitter uptake inhibitor, OP3 receptor agonist, serotonin uptake inhibitor
1614	norethisterone	ovulation inhibitor
1615	zosuquidar	P glycoprotein inhibitor, P glycoprotein modulator
1616	acarbose	glucosidase inhibitor
1617	n-formylmethionylalanine	macrophage activator
1618	prothionamide	mycobacterium tuberculosis enoyl-[acyl-carrier-protein] reductase [NADH] (inhA) inhibitor
1619	fluconazole	cell wall synthesis inhibitor, sterol demethylase inhibitor
1620	medetomidine	adrenergic receptor agonist, MAP kinase inhibitor
1621	SR-57227A	serotonin receptor agonist, serotonin release enhancer
1622	pravastatin	HMGCR inhibitor
1623	TFMPP	serotonin receptor agonist, serotonin receptor antagonist
1624	spiramide	dopamine receptor antagonist, serotonin receptor antagonist
1625	hydrocortisone	corticosteroid agonist, glucocorticoid receptor agonist, immunosuppressant, interleukin receptor antagonist
1626	vinpocetine	phosphodiesterase inhibitor, sodium channel blocker, voltage-sensitive sodium channel inhibitor
1627	tinidazole	anti-protozoal
1628	tetrahydrobiopterin	nitric oxide stimulant, nitric oxide synthase stimulant, phenylalanine 4-hydroxylase stimulant, nitric oxide synthase activator
1629	resmethrin	cytochrome P450 inhibitor
1630	trapidil	calcium channel antagonist, PDGFR tyrosine kinase receptor inhibitor, platelet aggregation inhibitor, protein kinase activator
1631	SD-169	p38 MAPK inhibitor
1632	ramipril	angiotensin converting enzyme inhibitor
1633	HA-1004	calcium channel blocker
1634	ergometrine	adrenergic receptor stimulatant, serotonin receptor stimulant
1635	SR-59230A	adrenergic receptor antagonist
1636	somatostatin	somatostatin receptor agonist, growth hormone receptor antagonist, growth hormone release inhibitor, somatostatin receptor ligand
1637	lumicolchicine	colchicine isomer, non-binder of microtubules
1638	tetramisole	alkaline phosphatase inhibitor
1639	pheniramine	acetylcholine receptor antagonist
1640	NVP-AUY922	HSP inhibitor
1641	rucaparib	PARP inhibitor
1642	isocarboxazid	monoamine oxidase inhibitor
1643	articaine	local anesthetic
1644	mecillinam	cell wall synthesis inhibitor
1645	meloxicam	cyclooxygenase inhibitor, prostanoid receptor inhibitor
1646	flecainide	sodium channel blocker, sodium channel inhibitor, voltage-gated sodium channel blocker
1647	tiaprofenic-acid	cyclooxygenase inhibitor
1648	baeomycesic-acid	lipoxygenase inhibitor
1649	BRD-A69470004	XIAP inhibitor
1650	benazepril	angiotensin converting enzyme inhibitor
1651	ZM-447439	Aurora kinase inhibitor
1652	adipiodone	serum albumin binder
1653	ICI-199441	opioid receptor agonist
1654	mead-acid	KPL-1 tumor suppressor
1655	nimesulide	cyclooxygenase inhibitor, matrix metalloprotease inhibitor, prostanoid receptor inhibitor
1656	roquinimex	T cell inhibitor, T cell stimulant, tumor necrosis factor receptor antagonist
1657	SDM25N	opioid receptor antagonist
1658	halcinonide	glucocorticoid receptor agonist
1659	HNHA	HDAC inhibitor
1660	BRD-K47448802	acetylcholine receptor allosteric modulator
1661	liquiritigenin	aromatase inhibitor
1662	CGP-12177	adrenergic receptor agonist
1663	cyclopamine	smoothened receptor antagonist, hedgehog pathway inhibitor
1664	cyclopiazonic-acid	ATPase inhibitor
1665	thioperamide	histamine receptor antagonist, histamine receptor inverse agonist
1666	RK-682	heparanase inhibitor, tyrosine phosphatase inhibitor
1667	avrainvillamide-analog-6	nucleophosmin inhibitor
1668	enobosarm	androgen receptor modulator, androgen receptor agonist
1669	iocetamic-acid	molecule used as a contrast medium
1670	axitinib	VEGFR inhibitor, PDGFR tyrosine kinase receptor inhibitor, angiogenesis inhibitor, KIT inhibitor, VEGFR antagonist
1671	naproxen	cyclooxygenase inhibitor
1672	diltiazem	L-type calcium channel blocker, calcium channel antagonist, calcium channel blocker
1673	RO-25-6981	ionotropic glutamate receptor antagonist, monamine transporter modulator
1674	PSB-069	NTPDase inhibitor
1675	MRS-1754	adenosine receptor antagonist
1676	ziprasidone	dopamine receptor antagonist, serotonin receptor antagonist, norepinephrine reuptake inhibitor, serotonin receptor agonist, serotonin reuptake inhibitor

1676	ziprasidone	dopamine receptor antagonist, serotonin receptor antagonist, norepinephrine reuptake inhibitor, serotonin receptor agonist, serotonin reuptake inhibitor
1677	tranylcypromine	cytochrome P450 inhibitor, histone lysine demethylase inhibitor, monoamine oxidase inhibitor
1678	HTMT	histamine receptor agonist
1679	zolmitriptan	serotonin receptor agonist
1680	W-13	calmodulin antagonist
1681	reversine	Aurora kinase inhibitor, adenosine receptor antagonist
1682	GR-206	aryl hydrocarbon receptor ligand
1683	practolol	adrenergic receptor antagonist
1684	nafcillin	antibiotic
1685	sulfasalazine	cyclooxygenase inhibitor, disease modifying antirheumatic drug, lipoxygenase inhibitor, NFkB pathway inhibitor, prostanoid receptor inhibitor, thromboxane receptor antagonist
1686	mevastatin	HMGCR inhibitor
1687	RHO-kinase-inhibitor-II	ROCK inhibitor
1688	CAM-9-026	ADAM10 inhibitor
1689	HG-6-64-01	RAF inhibitor, Abl kinase inhibitor, ephrin receptor inhibitor, KIT inhibitor, MAP kinase inhibitor, MEK inhibitor, p38 MAPK inhibitor, src inhibitor
1690	trifluoperazine	breast cancer resistance protein inhibitor, calcium/calmodulin dependent protein kinase inhibitor, dopamine receptor, dopamine receptor antagonist, sodium channel blocker
1691	tetrabenazine	vesicular monoamine transporter inhibitor
1692	SR-142948	neurotensin receptor antagonist
1693	eticlopride	dopamine receptor antagonist
1694	saclofen	GABA receptor antagonist
1695	BRD-A89049230	thyroid-stimulating hormone receptor agonist
1696	BRD-K67258146	mannose-6-phosphate isomerate inhibitor
1697	tropanyl-3,5-dimethylbenzoate	serotonin receptor antagonist
1698	calycanthine	GABA release inhibitor
1699	monastrol	kinesin-like spindle protein inhibitor
1700	BRD-K24127443	lipoxygenase inhibitor
1701	prostaglandin	prostanoid receptor antagonist
1702	retinyl	vitamin analog
1703	fraxetin	anti-oxidative and anti-apoptotic agent
1704	naltrexone	opioid receptor antagonist, opioid receptor ligand
1705	PD-158780	EGFR inhibitor, epidermal growth factor receptor (EGFR) inhibitor
1706	cabergoline	dopamine receptor agonist, prolactin inhibitor, prolactin secretion inhibitor
1707	flumazenil	benzodiazepine receptor antagonist, GABA benzodiazepine site receptor antagonist
1708	BRD-K72817452	lipoxygenase inhibitor
1709	edaravone	free radical scavenger
1710	remacemide	glutamate receptor antagonist, glutamate receptor agonist
1711	voriconazole	cell wall synthesis inhibitor, cytochrome P450 inhibitor, lanosterol demethylase inhibitor
1712	AG-99	tyrosine kinase inhibitor
1713	n-arachidonyl-GABA	arachidonyl amino acid that inhibits pain
1714	marbofloxacin	DNA gyrase inhibitor
1715	naftidrofuryl	adrenergic receptor antagonist
1716	naltrindole	opioid receptor antagonist
1717	CL-218872	GABA receptor agonist
1718	TUL-XXI039	serine/threonine kinase inhibitor
1719	tienilic-acid	sodium/potassium/chloride transporter inhibitor, uric acid diuretic
1720	griseofulvin	microtubule inhibitor, microtubule stabilizing agent, mitosis inhibitor
1721	BRD-K68783079	CLK inhibitor, dual specificity tyrosine-(Y)-phosphorylation regulated kinase inhibitor
1722	elvitegravir	HIV integrase inhibitor, HIV inhibitor
1723	fasudil	ROCK inhibitor, calcium sensitizer, PKA inhibitor, protein kinase inhibitor, rho associated kinase inhibitor
1724	GR-32191	thromboxane receptor antagonist, prostanoid receptor antagonist
1725	itopride	dopamine receptor antagonist, acetylcholinesterase inhibitor
1726	YM-976	phosphodiesterase inhibitor, tumor necrosis factor production inhibitor
1727	cyproterone	androgen receptor antagonist, DNA directed DNA polymerase stimunlant, Estradiol 17 beta dehydrogenase stimulant, estrone sulfotransferase stimulant, progesterone receptor agonist, testosterone receptor antagonist
1728	velnacrine	calcium channel activator
1729	naloxone	opioid receptor antagonist
1730	DMAB-anabaseine	alpha receptor partial agonist
1731	cortisone	glucocorticoid receptor agonist
1732	bucladesine	adenosine receptor agonist, cAMP stimulant, vasodilator
1733	androstenedione	cytochrome P450 inhibitor
1734	lofexidine	adrenergic receptor agonist
1735	thioperamide	histamine receptor antagonist, histamine receptor inverse agonist
1736	denbufylline	phosphodiesterase inhibitor
1737	PD-169316	p38 MAPK inhibitor
1738	ketoconazole	14-alpha demethylase inhibitor, androgen receptor ligand, aromatase inhibitor, cell wall synthesis inhibitor, cortisol synthesis inhibitor, P glycoprotein inhibitor, sterol demethylase inhibitor
1739	L-BSO	γ-glutamylcysteine synthetase inhibitor
1740	tracazolate	GABA receptor agonist, GABA receptor modulator
1741	ST-91	adrenergic receptor agonist
1742	pinocembrin	androgen receptor ligand, CYP1B1 inhibitor, reducing agent, steroid 5alpha-reductase inhibitor
1743	SDZ-205-557	serotonin receptor antagonist
1744	cediranib	VEGFR inhibitor, KIT inhibitor, angiogenesis inhibitor, VEGFR antagonist
1745	spectinomycin	30S ribosomal subunit inhibitor
1746	metanephrine	epinephrine metabolite, levels are diagnostic for the neoplasm pheochromocytoma
1747	BW-B70C	lipoxygenase inhibitor
1748	L-701252	glutamate receptor antagonist
1749	iproniazid	monoamine oxidase inhibitor
1750	P-1075	ATP channel activator, potassium channel agonist
1751	bromocriptine	dopamine receptor agonist, dopamine receptor antagonist, prolactin secretion inhibitor
1752	aliskiren	renin inhibitor, peptidase inhibitor, protease inhibitor
[illegible]	[illegible]	[illegible]

1753	met-leu-phe	in touchstone
1754	ritanserin	serotonin receptor antagonist
1755	maackiain	interleukin synthesis inhibitor, sodium/glucose cotransporter inhibitor
1756	BRD-A51929314	prostanoid receptor antagonist, thromboxane receptor antagonist
1757	pirfenidone	FGFR antagonist, p38 MAPK inhibitor, TGF beta receptor antagonist, TGF beta receptor inhibitor, tumor necrosis factor production inhibitor, tumor necrosis factor receptor antagonist
1758	isoxsuprine	adrenergic receptor agonist
1759	aminoglutethimide	antiglucocorticoid, aromatase inhibitor, corticosteroid antagonist, steroid sulfatase inhibitor
1760	dihydroergotamine	serotonin receptor agonist, adrenergic receptor partial agonist, dopamine receptor agonist
1761	bucladesine	adenosine receptor agonist, cAMP stimulant, vasodilator
1762	sulfafurazole	endothelin receptor antagonist, FOLP inhibitor
1763	fenbufen	cyclooxygenase inhibitor
1764	MDM2-inhibitor	MDM inhibitor
1765	FTase-inhibitor-B581	farnesyltransferase inhibitor, protein farnesyltransferase inhibitor
1766	Y-27152	potassium channel activator, potassium channel agonist
1767	fludroxycortide	corticosteroid, glucocorticoid receptor agonist
1768	rutin	aldose reductase inhibitor, antioxidant, capillary stabilizing agent, nitric oxide scavenger, prolyl 4-hydroxylase inhibitor
1769	RWJ-21757	toll-like receptor ligand
1770	pentylenetetrazol	GABA receptor antagonist
1771	cetirizine	histamine receptor antagonist
1772	prednisolone	glucocorticoid receptor agonist
1773	diethylcarbamazine	lipoxygenase inhibitor
1774	ramipril	angiotensin converting enzyme inhibitor
1775	pseudopelletierine	stimulant reflex trigger used as an anthelmintic and anti-amoeboid
1776	JX-401	p38 MAPK inhibitor
1777	fluprostenol	prostaglandin F receptor agonist
1778	cilostamide	phosphodiesterase inhibitor
1779	splitomycin	SIRT inhibitor
1780	SR-27897	CCK receptor antagonist
1781	siguazodan	histamine receptor antagonist, phosphodiesterase inhibitor
1782	LFM-A13	Bruton's tyrosine kinase (BTK) inhibitor
1783	nadolol	adrenergic receptor antagonist
1784	genistein	tyrosine kinase inhibitor, angiogenesis inhibitor, CFTR channel activator, EGFR inhibitor, estrogen receptor agonist, histidine kinase inhibitor, immunosuppressant, isoflavone agonist, protein tyrosine kinase inhibitor, reducing a
1785	piperidolate	acetylcholine receptor antagonist
1786	CAY-10578	casein kinase inhibitor
1787	clozapine	serotonin receptor antagonist, dopamine receptor antagonist, adrenergic receptor antagonist
1788	metyrapone	cytochrome P450 inhibitor, 11-beta hydroxysteroid dehydrogenase inhibitor, mineralocorticoid receptor antagonist
1789	prometon	photosynthesis inhibitor
1790	RX-821002	adrenergic receptor antagonist
1791	BRD-K45031696	cytohesin inhibitor
1792	quipazine	serotonin receptor agonist
1793	proxymetacaine	membrane integrity inhibitor, potassium channel antagonist, sodium channel blocker
1794	diethyltoluamide	DEET, activator of fly antenna ionotropic receptor IR40a
1795	LY-2140023	glutamate receptor agonist
1796	urapidil	adrenergic receptor antagonist, serotonin receptor agonist
1797	JNJ-38877605	hepatocyte growth factor receptor inhibitor, tyrosine kinase inhibitor
1798	pramipexole	dopamine receptor agonist
1799	famciclovir	DNA polymerase inhibitor, DNA directed DNA polymerase inhibitor
1800	AM-630	cannabinoid receptor antagonist, cannabinoid receptor inverse agonist
1801	glycodeoxycholic-acid	apoptosis activator
1802	estrone	estrogen receptor agonist, estrogenic hormone
1803	phenazone	cyclooxygenase inhibitor
1804	fusaric-acid	chelating agent, dopamine beta hydroxylase inhibitor
1805	azacyclonol	ataractive drug used to diminish hallucinations
1806	CS-1657	PARP inhibitor
1807	ruxolitinib	JAK inhibitor, tyrosine kinase inhibitor
1808	hispidin	beta secretase inhibitor, cyclooxygenase inhibitor, HIV integrase inhibitor, PKC inhibitor, prolyl endopeptidase inhibitor, xanthine oxidase inhibitor
1809	3-matida	glutamate receptor antagonist
1810	BJM-CSC-19	compound cytotoxic to breast cancer stem cells
1811	tenofovir	reverse transcriptase inhibitor, nucleoside reverse transcriptase inhibitor, nucleotide reverse transcriptase inhibitor
1812	butylparaben	antimicrobial preservative
1813	losartan	angiotensin receptor antagonist
1814	TCB2	serotonin receptor agonist
1815	apafant	platelet activating factor receptor antagonist
1816	nizatidine	histamine receptor antagonist
1817	IBC-293	hydroxycarboxylic acid receptor agonist
1818	AQ-RA741	acetylcholine receptor antagonist
1819	danazol	estrogen receptor antagonist, luteinizing hormone releasing hormone antagonist, progesterone receptor agonist, steroid derivative with antigonadotropic and anti-estrogenic activities, tumor necrosis factor modulator
1820	L-694247	serotonin receptor agonist
1821	cilastatin	dehydropeptidase inhibitor
1822	etilefrine	adrenergic receptor agonist
1823	piperine	monoamine oxidase inhibitor, ACAT inhibitor
1824	fexaramine	FXR agonist
1825	daphnetin	protein kinase inhibitor
1826	carbacyclin	IP receptor activator, PPARbeta receptor activator
1827	equilin	estrogen receptor agonist
1828	LY-16350	dopamine receptor agonist, dopamine receptor antagonist
1829	pravastatin	HMGCR inhibitor

1830	evoxine	antagonist of strychnine and pentylenetrazole; enhances effects of narcotics
1831	pergolide	dopamine receptor agonist, calcium-activated potassium channel blocker, potassium channel blocker, prolactin inhibitor
1832	sulpiride	carbonic anhydrase inhibitor, dopamine receptor, dopamine receptor antagonist
1833	aminogenistein	PKC inhibitor, src inhibitor
1834	LY-344864	serotonin receptor agonist
1835	U-0126	MEK inhibitor, JAK inhibitor, MAP kinase inhibitor
1836	molsidomine	activator of soluble guanylyl cyclase, nitric oxide donor, nitric oxide stimulant
1837	SEW-2871	lysophospholipid receptor agonist
1838	zardaverine	phosphodiesterase inhibitor
1839	ranolazine	fatty acid oxidation partial inhibitor, Sodium Channel Blockers, sodium channel blocker
1840	cefatrizine	cell wall synthesis inhibitor
1841	cinalukast	leukotriene receptor antagonist
1842	orphenadrine	acetylcholine receptor antagonist
1843	tetrahydropalmatine	serotonin release inhibitor
1844	carbinoxamine	histamine receptor antagonist, L-type calcium channel blocker
1845	auraptene	lipoxygenase inhibitor, monoamine oxidase inhibitor, nitric oxide production inhibitor, nitric oxide synthase expression inhibitor
1846	BRL-54443	serotonin receptor agonist
1847	benzylpenicillin	PBP3 inhibitor
1848	GW-5074	RAF inhibitor, leucine rich repeat kinase inhibitor
1849	cromakalim	ATP channel activator, potassium channel agonist
1850	nisoxetine	dopamine transporter inhibitor, norepinephrine transporter inhibitor, serotonin transporter (SERT) inhibitor
1851	clomethiazole	GABA receptor agonist, GABA receptor antagonist, GABA receptor modulator, p38 MAPK inhibitor
1852	leucodin	melanin synthesis inhibitor
1853	dehydroisoandosterone	GABA receptor modulator
1854	tyrphostin	epidermal growth factor receptor (EGFR) inhibitor, receptor tyrosine protein kinase inhibitor
1855	ipriflavone	bone resorption inhibitor
1856	benzopurpurin-4b	binds HIV gp120 and inhibits HIV-1 entry into the neural cell line SK-N-MC
1857	benzohydroxamic-acid	metal chelator, anti bacterial
1858	GDC-0941	PI3K inhibitor
1859	prilocaine	anesthetic
1860	sildenafil	phosphodiesterase inhibitor
1861	homosalate	HSP activator
1862	bisoprolol	adrenergic receptor antagonist
1863	nevirapine	non-nucleoside reverse transcriptase inhibitor, reverse transcriptase inhibitor
1864	PRL-3-inhibitor-I	PRL phosphatase inhibitor, tyrosine phosphatase inhibitor
1865	cinnarizine	calcium ion channel antagonist, histamine receptor antagonist
1866	ponalrestat	aldose reductase inhibitor, reductase inhibitor
1867	boldine	acetylcholine receptor inhibitor, dopamine receptor antagonist
1868	PI-828	PI3K inhibitor
1869	anandamide	cannabinoid receptor agonist, potassium channel blocker, TRPV agonist
1870	biochanin-a	estrogen receptor agonist
1871	ethotoin	anticonvulsant
1872	BRD-K28680267	CCK receptor antagonist
1873	flurbiprofen	cyclooxygenase inhibitor
1874	lamivudine	nucleoside reverse transcriptase inhibitor, reverse transcriptase inhibitor
1875	tomelukast	leukotriene receptor antagonist
1876	methysergide	serotonin receptor antagonist
1877	GYKI-52466	glutamate receptor antagonist, kainate receptor antagonist
1878	genistein	tyrosine kinase inhibitor, angiogenesis inhibitor, CFTR channel activator, EGFR inhibitor, estrogen receptor agonist, histidine kinase inhibitor, immunosuppressant, isoflavone agonist, protein tyrosine kinase inhibitor, reducing a
1879	purmorphamine	smoothened receptor agonist
1880	bromhexine	mucolytic
1881	penicillin	cell wall synthesis inhibitor
1882	byssochlamic-acid	mycotoxin
1883	phenelzine	monoamine oxidase inhibitor
1884	etifenin	compound used in hepatobiliary scans of the liver
1885	captopril	angiotensin converting enzyme inhibitor
1886	veratridine	sodium channel activator
1887	etodolac	cyclooxygenase inhibitor, TRPV agonist
1888	PHTPP	estrogen receptor antagonist
1889	nornicotine	acetylcholine receptor agonist
1890	theophylline	adenosine receptor antagonist, chitinase inhibitor, GABA receptor antagonist, phosphodiesterase inhibitor
1891	rimexolone	glucocorticoid receptor agonist, immunosuppressant, mineralocorticoid receptor agonist
1892	DPN	estrogen receptor agonist
1893	tebuthiuron	photosynthesis inhibitor
1894	CO-102862	sodium channel blocker
1895	vitexin	antioxidant
1896	erythromycin	NFkB pathway inhibitor, 50S ribosomal subunit inhibitor, motilin receptor agonist, RPLV inhibitor
1897	clonidine	adrenergic receptor agonist
1898	KB-R7943	sodium/calcium exchange inhibitor
1899	BRD-K73285375	lipoxygenase inhibitor
1900	FGIN-1-43	benzodiazepine receptor ligand
1901	lithocholic-acid	FXR antagonist, MDM inhibitor, PXR ligand, tyrosine phosphatase inhibitor, vitamin D receptor ligand
1902	CP-724714	receptor tyrosine protein kinase inhibitor, EGFR inhibitor, tyrosine kinase inhibitor
1903	SB-203580	p38 MAPK inhibitor, calcium channel activator, interleukin inhibitor, stress activated protein kinase inhibitor
1904	yohimbine	adrenergic receptor antagonist
1905	ranitidine	histamine receptor antagonist
1906	pifithrin	interleukin receptor antagonist

1907	LE-300	dopamine receptor antagonist, dopamine receptor ligand, serotonin receptor ligand
1908	cyclopentolate	acetylcholine receptor antagonist
1909	ZD-7114	adrenergic receptor agonist
1910	trans-7-hydroxy-pipat	dopamine receptor ligand
1911	quetiapine	dopamine receptor antagonist, serotonin receptor antagonist, adrenergic receptor antagonist, histamine receptor antagonist
1912	zibotentan	endothelin receptor antagonist
1913	sunitinib	FLT3 inhibitor, KIT inhibitor, PDGFR tyrosine kinase receptor inhibitor, RET tyrosine kinase inhibitor, VEGFR inhibitor, angiogenesis inhibitor, colony stimulating factor receptor antagonist, colony stimulating factor receptor inhib
1914	pregnenolone	acetylcholine release enhancer, dopamine release enhancer, GABA receptor negative allosteric modulator, glutamate receptor modulator, progesterone receptor agonist, steroid hormone inhibitor of CYP17A1 and SULT2B1
1915	prednisolone	glucocorticoid receptor agonist
1916	zatebradine	HCN (hyperpolarization activated cyclic nucleotide gated) channel blocker, HCN channel antagonist, potassium channel antagonist, sodium channel blocker
1917	VX-702	p38 MAPK inhibitor
1918	scopolamine	acetylcholine receptor antagonist
1919	BRD-K06217810	CLK inhibitor, dual specificity tyrosine-(Y)-phosphorylation regulated kinase inhibitor, dual-specificity tyrosine-(Y)-phosphorylation regulated kinase 1B inhibitor
1920	W-9	calmodulin antagonist
1921	immepip	histamine receptor agonist
1922	imperatorin	acetylcholinesterase inhibitor, nitric oxide production inhibitor, Sodium Channel Blockers
1923	L-655708	GABA receptor inverse agonist
1924	fenoldopam	dopamine receptor agonist
1925	memantine	glutamate receptor antagonist, glutamate release inhibitor
1926	guanfacine	adrenergic receptor agonist, HCN (hyperpolarization activated cyclic nucleotide gated) channel blocker
1927	clioquinol	chelating agent, antiamyloidogenic agent, beta amyloid antagonist, carbonic anhydrase inhibitor
1928	yohimbine	adrenergic receptor antagonist
1929	bethanechol	acetylcholine receptor agonist
1930	diphenhydramine	histamine receptor antagonist
1931	apoptosis-activator-II	carboxylesterase inhibitor
1932	epigallocatechin	AP inhibitor, aromatase inhibitor, bacterial efflux pump inhibitor, beta amyloid aggregation inhibitor, beta amyloid protein neurotoxicity inhibitor, beta secretase inhibitor, DNA gyrase inhibitor, dual specificity tyrosine-(Y)-phospho
1933	promazine	dopamine receptor, dopamine receptor antagonist
1934	pivmecillinam	cell wall synthesis inhibitor
1935	BD-1063	sigma receptor antagonist
1936	selegiline	monoamine oxidase inhibitor, superoxide dismutase stimulant
1937	kitasamycin	protein synthesis inhibitor
1938	ivachtin	caspase inhibitor
1939	rufloxacin	topoisomerase inhibitor
1940	PG-9	presynaptic cholinergic modulator
1941	cyanopindolol	adrenergic receptor antagonist
1942	clomifene	estrogen receptor antagonist, estrogen receptor modulator, selective estrogen receptor modulator (SERM), testosterone receptor agonist
1943	racephedrine	adrenergic receptor agonist
1944	methocarbamol	muscle relaxant
1945	LM-1685	cyclooxygenase inhibitor
1946	efavirenz	HIV reverse transcriptase inhibitor, non-nucleoside reverse transcriptase inhibitor, reverse transcriptase inhibitor
1947	anandamide	cannabinoid receptor agonist, potassium channel blocker, TRPV agonist
1948	tremorine	acetylcholine receptor agonist
1949	5'-guanidinonaltrindole	opioid receptor antagonist
1950	pyrazinamide	fatty acid synthase inhibitor
1951	guanabenz	adrenergic receptor agonist
1952	nicorandil	ATP channel activator, nitric oxide donor, potassium channel agonist
1953	ML-7	myosin light chain kinase inhibitor
1954	SCH-23390	dopamine receptor antagonist
1955	sibutramine	serotonin reuptake inhibitor, adrenergic transmitter uptake inhibitor, dopamine reuptake inhibitor, norepinephrine reuptake inhibitor, norepinephrine transporter inhibitor, serotonin uptake inhibitor
1956	phenolphthalein	weak acid used as a pH indicator
1957	SB-590885	RAF inhibitor
1958	synephrine	adrenergic receptor agonist, L-type calcium channel activator
1959	scopolamine	acetylcholine receptor antagonist
1960	dexamethasone	glucocorticoid receptor agonist, corticosteroid agonist, immunosuppressant
1961	tranilast	angiogenesis inhibitor, histamine receptor antagonist, histamine release inhibitor, indoleamine 2,3-dioxygenase activator, interferon gamma synthesis inhibitor, interleukin receptor modulator, interleukin synthesis enhancer, inte
1962	buflomedil	adrenergic receptor antagonist, calcium channel antagonist
1963	dihydroergocristine	adrenergic receptor antagonist, prolactin inhibitor, adrenergic receptor partial agonist, dopamine receptor agonist, dopamine receptor partial agonist, dopamine receptor partial antagonist, serotonin receptor antagonist
1964	temefos	cholinesterase inhibitor
1965	bis-tyrphostin	EGFR inhibitor, dynamin GTPase inhibitor
1966	BRD-K88742110	HDAC inhibitor, interleukin synthesis inhibitor
1967	ozagrel	thromboxane synthase inhibitor, platelet aggregation inhibitor, selective thromboxane synthetase inhibitor
1968	FR-122047	cyclooxygenase inhibitor
1969	pirenzepine	acetylcholine receptor antagonist
1970	clobenpropit	histamine receptor antagonist, histamine receptor inverse agonist
1971	gatifloxacin	topoisomerase inhibitor, DNA gyrase inhibitor
1972	L-693403	sigma receptor antagonist, sigma receptor ligand
1973	primidone	GABA receptor antagonist, unidentified pharmacological activity
1974	17-beta-estradiol	estrogen receptor agonist
1975	relcovaptan	vasopressin receptor antagonist
1976	milrinone	phosphodiesterase inhibitor
1977	PCO-400	potassium channel activator, potassium channel agonist
1978	etazolate	phosphodiesterase inhibitor, alpha secretase activator, GABA receptor modulator
1979	VX-222	HCV inhibitor, RNA-directed RNA polymerase inhibitor
1980	FPL-55712	leukotriene receptor antagonist, leukotriene synthesis inhibitor
1981	cinchonine	antimalarial agent
1982	L-741742	dopamine receptor antagonist
1983	ubenimex	leukotriene hydrolase inhibitor, alanine aminopeptidase inhibitor, aminopeptidase B inhibitor, immunostimulant, peptidase inhibitor

[illegible]	[illegible]	[illegible]
1984	atenolol	adrenergic receptor antagonist
1985	L-692585	growth hormone releasing peptide ligand agonist, growth hormone secretagogue
1986	ICI-204448	opioid receptor agonist
1987	naproxen	cyclooxygenase inhibitor
1988	heliotrine	in touchstone
1989	brinzolamide	carbonic anhydrase inhibitor
1990	sotalol	adrenergic receptor antagonist, polarization inhibitor, potassium channel antagonist
1991	nomifensine	norepinephrine-dopamine reuptake inhibitor
1992	RHC-80267	triacylglycerol lipase inhibitor
1993	SC-68376	p38 MAPK inhibitor
1994	mestranol	estrogen receptor agonist
1995	ZD-7155	angiotensin receptor antagonist
1996	alverine	smooth muscle relaxant
1997	SR-95639A	acetylcholine receptor agonist
1998	ampicillin	cell wall synthesis inhibitor
1999	TCPOBOP	constitutive androstane receptor (CAR) agonist
2000	GBR-13069	dopamine uptake inhibitor
2001	methyl-angolensate	apoptosis inhibitor
2002	rifaximin	50S ribosomal subunit inhibitor, DNA directed DNA polymerase inhibitor, PXR agonist, RNA synthesis inhibitor
2003	SKF-77434	dopamine receptor agonist, dopamine receptor partial agonist
2004	resorcinol	phosphodiesterase inhibitor
2005	ioxaglic-acid	iodine contrast agent
2006	GR-113808	serotonin receptor antagonist
2007	valsartan	angiotensin receptor antagonist
2008	oxybenzone	lipase inhibitor
2009	FK-888	tachykinin antagonist
2010	17-hydroxyprogesterone-caproate	progesterone receptor agonist
2011	SB-205384	GABA receptor modulator
2012	MR-16728	acetylcholine release enhancer
2013	acamprosate	glutamate receptor antagonist, excitatory amino acid receptor antagonist, GABA receptor agonist, glutamate receptor modulator
2014	salsolinol	monoamine oxidase inhibitor, tyrosine hydroxylase inhibitor
2015	estriol	estrogen receptor agonist, estrogen receptor antagonist
2016	GW-9662	PPAR receptor antagonist
2017	EMD-66684	angiotensin receptor antagonist
2018	nizatidine	histamine receptor antagonist
2019	naltriben	opioid receptor antagonist
2020	herniarin	acetylcholinesterase inhibitor
2021	bergenin	anti-arthritic compound that modulates Th1/Th2 cytokine balance
2022	procainamide	sodium channel blocker, voltage-gated sodium channel blocker
2023	niflumic-acid	cyclooxgenase inhibitor
2024	vecuronium	acetylcholine receptor antagonist
2025	CS-110266	dopamine receptor agonist
2026	CGP-55845	GABA receptor antagonist
2027	labetalol	adrenergic receptor antagonist
2028	taxifolin	apolipoprotein secretion inhibitor, beta amyloid aggregation inhibitor, cholesterol biosynthesis inhibitor, HMGCR inhibitor, NFkB pathway modulator, opioid receptor antagonist, reverse transcriptase inhibitor
2029	milnacipran	serotonin reuptake inhibitor, adrenergic transmitter uptake inhibitor, norepinephrine reputake inhibitor, norepinephrine reuptake inhibitor, serotonin uptake inhibitor
2030	naringin	cytochrome P450 inhibitor
2031	moexipril	angiotensin converting enzyme inhibitor, phosphodiesterase inhibitor
2032	phenprobamate	skeletal muscle relaxant
2033	lorazepam	chloride channel agonist, GABA benzodiazepine site receptor agonist, GABA receptor agonist, potassium channel agonist
2034	quinidine	cytochrome P450 inhibitor, P glycoprotein inhibitor, sodium current blocker
2035	ethoprop	acetylcholinesterase inhibitor
2036	pentoxifylline	phosphodiesterase inhibitor, acetylcholinesterase inhibitor, chitinase inhibitor, tumor necrosis factor production inhibitor
2037	BRD-K86682249	tyrosine phosphatase inhibitor
2038	quercetagetin	Pim kinase inhibitor
2039	TCS-359	FLT3 inhibitor
2040	psoromic-acid	Rab-Prenylation Inhibitor
2041	CP-93129	serotonin receptor agonist
2042	prostaglandin-e1	prostanoid receptor agonist, prostanoid receptor antagonist
2043	dovitinib	EGFR inhibitor, FGFR inhibitor, FLT3 inhibitor, PDGFR tyrosine kinase receptor inhibitor, VEGFR inhibitor, angiogenesis inhibitor, FGFR antagonist, KIT inhibitor, VEGFR antagonist
2044	finasteride	5 alpha reductase inhibitor, androgen receptor antagonist, steroid 5alpha-reductase inhibitor
2045	IB-MECA	adenosine receptor agonist, granulocyte colony stimulating factor agonist
2046	BRD-K66782112	histamine receptor antagonist
2047	felbamate	carbonic anhydrase inhibitor, GABA receptor modulator, glutamate receptor blocker
2048	BRD-K39187410	antiamyloidogenic agent
2049	troxipide	glucosamine synthetase stimulant
2050	SB-200646	serotonin receptor antagonist
2051	CP-94253	serotonin receptor agonist
2052	alaproclate	serotonin receptor antagonist, serotonin reuptake inhibitor
2053	diprotin-a	dipeptidyl peptidase inhibitor
2054	veliparib	PARP inhibitor, DNA repair enzyme inhibitor
2055	JNJ-10191584	histamine receptor antagonist, histamine receptor silent antagonist
2056	meclocycline	tetracycline antibiotic
2057	BRD-K70751730	histone lysine demethylase inhibitor
2058	tripelennamine	histamine receptor antagonist
2059	SRC-kinase-inhibitor-I	src inhibitor
[illegible]	[illegible]	[illegible]

2060	oxprenolol	adrenergic receptor antagonist
2061	SQ-22536	adenylyl cyclase inhibitor
2062	tacrolimus	calcineurin inhibitor, FK506-binding protein inhibitor, immunosuppressant, insulin expression inhibitor, interleukin receptor antagonist, macrolide calcineurin inhibitor, rotamase inhibitor, T cell inhibitor
2063	SC-560	cyclooxygenase inhibitor
2064	L-161982	prostanoid receptor antagonist, angiotensin receptor antagonist
2065	cromoglicic-acid	immunosuppressant
2066	brucine	glycine receptor antagonist
2067	AM-92016	glutamate receptor antagonist, ionotropic glutamate receptor antagonist, time-dependent delayed rectifier potassium current blocker
2068	procaterol	adrenergic receptor agonist
2069	RU-24969	serotonin receptor agonist
2070	zuclopenthixol	dopamine receptor antagonist
2071	tolterodine	acetylcholine receptor antagonist
2072	iopanoic-acid	radiopaque medium
2073	arecaidine	acetylcholine receptor agonist
2074	benproperine	anti-tussive
2075	hypericin	PKC inhibitor, tyrosine kinase inhibitor, unidentified pharmacological activity
2076	ozagrel	thromboxane synthase inhibitor, platelet aggregation inhibitor, selective thromboxane synthetase inhibitor
2077	pirlindole	monoamine oxidase inhibitor
2078	NPC-15199	ICAM 1 antagonist, increases intracellular calcium levels
2079	niacin	vitamin agonist
2080	dicloxacillin	bacterial cell wall synthesis inhibitor
2081	tolbutamide	ATP channel blocker
2082	ibudilast	phosphodiesterase inhibitor, leukotriene receptor antagonist, toll-like receptor antagonist, macrophage migration inhibiting factor inhibitor, macrophage migration inhibiting factor modulator, mediator release inhibitor, nitric oxide
2083	VU-0400195-3	glutamate receptor modulator
2084	naftopidil	adrenergic receptor antagonist, calcium channel antagonist
2085	epirizole	cyclooxygenase inhibitor
2086	minoxidil	antialopecia agent, ATP channel activator, ATP-sensitive potassium channel agonist, KATP activator, Kir6 channel (KATP) activator, polarization activator, renin secretion stimulant
2087	aminopentamide	anticholinergic agent
2088	PP-3	EGFR kinase inhibitor
2089	thalidomide	angiogenesis inhibitor, cereblon inhibitor, tumor necrosis factor production inhibitor, tumor necrosis factor receptor antagonist, tumor necrosis factor receptor inhibitor
2090	RO-10-5824	dopamine receptor partial agonist
2091	L-745870	dopamine receptor antagonist
2092	DAPT-GSI-IX	antiamyloidogenic agent, gamma secretase inhibitor
2093	tamoxifen	estrogen receptor antagonist, selective estrogen receptor modulator (SERM), estrogen receptor agonist, estrogen receptor modulator, PKC inhibitor
2094	barasertib	Aurora kinase inhibitor, mitotic inhibitor, protein kinase inhibitor
2095	niridazole	oogenesis inhibitor, spermatogenesis inhibitor
2096	tenoxicam	cyclooxygenase inhibitor, prostanoid receptor inhibitor
2097	acarbose	glucosidase inhibitor
2098	dopamine	dopamine receptor agonist
2099	carpindolol	adrenergic receptor antagonist, serotonin receptor antagonist
2100	BMS-182874	endothelin receptor antagonist
2101	formestane	aromatase inhibitor
2102	iodophenpropit	histamine receptor antagonist, histamine receptor inhibitor, ionotropic glutamate receptor antagonist
2103	chenodeoxycholic-acid	11-beta hydroxysteroid dehydrogenase inhibitor, FXR agonist
2104	CGP-52432	GABA receptor antagonist
2105	NS-3694	glutamate receptor antagonist
2106	ciclacillin	cell wall synthesis inhibitor
2107	asiaticoside	possible antitumor agent
2108	tropicamide	acetylcholine receptor antagonist, anticholinergic
2109	simvastatin	HMGCR inhibitor
2110	GSK-0660	PPAR receptor inhibitor
2111	BRD-K98404142	glucose 6 phosphate dehydrogenase inhibitor, immunostimulant
2112	BRD-A97035593	XIAP inhibitor
2113	epothilone	inhibition of microtubulefunction, microtubule stabilizing agent, microtubule stimulant, tubulin inhibitor
2114	kavain	calcium channel modulator, mTOR inhibitor, Sodium Channel Blockers
2115	isoxicam	cyclooxygenase inhibitor
2116	timolol	adrenergic receptor antagonist
2117	didanosine	nucleoside reverse transcriptase inhibitor, reverse transcriptase inhibitor
2118	tyrphostin-46	tyrosine kinase inhibitor
2119	cycloserine	glutamate receptor agonist, glutamate receptor modulator
2120	gabazine	GABA receptor antagonist
2121	lysergol	ergoline alkaloid
2122	labetalol	adrenergic receptor antagonist
2123	eprosartan	angiotensin receptor antagonist
2124	penciclovir	DNA directed DNA polymerase inhibitor
2125	gestrinone	progesterone receptor antagonist, Estradiol 17 beta dehydrogenase stimulant, estrone sulfotransferase stimulant
2126	dihydrexidine	dopamine receptor agonist
2127	desoxycortone	mineralocorticoid receptor agonist
2128	raclopride	dopamine receptor antagonist
2129	BU-224	imidazoline receptor ligand
2130	CI-966	GABA uptake inhibitor, GAT inhibitor
2131	talampicillin	antibiotic
2132	bemesetron	serotonin receptor antagonist
2133	alpha-estradiol	estrogen receptor agonist
2134	BAS-09104376	HIV integrase inhibitor
2135	alosetron	serotonin receptor antagonist
2136	xaliproden	serotonin receptor agonist, nerve growth factor agonist, neurotrophic factor enhancer
[illegible]	[illegible]	[illegible]

2137	tolazamide	ATP channel blocker
2138	oleanolic-acid	antitumor agent
2139	Ro-04-6790	serotonin receptor antagonist, serotonin receptor inhibitor
2140	betaxolol	adrenergic receptor antagonist
2141	ALW-II-49-7	ephrin inhibitor
2142	larixinic-acid	comopund that interacts with metal centers
2143	cimetidine	histamine receptor antagonist, histamine receptor inhibitor
2144	nimetazepam	chloride channel agonist, GABA receptor agonist, potassium channel agonist
2145	nadolol	adrenergic receptor antagonist
2146	alprenolol	adrenergic receptor antagonist
2147	calpeptin	calpain inhibitor, proteasome inhibitor, tyrosine phosphatase inhibitor
2148	protein-tyrosine-phosphatase-inhibitor-IV	tyrosine phosphatase inhibitor
2149	SB-218795	tachykinin antagonist
2150	U-74389F	lipid peroxidase inhibitor
2151	BRD-K78883024	neuropeptide receptor antagonist
2152	YM-298198	glutamate receptor antagonist
2153	m-chlorophenylbiguanide	serotonin receptor agonist
2154	terguride	adrenergic receptor antagonist, dopamine autoreceptor agonist, dopamine receptor agonist, dopamine receptor partial agonist, platelet aggregation inhibitor, prolactin secretion inhibitor, serotonin receptor antagonist
2155	anabasine	acetylcholine receptor agonist
2156	piperacetazine	antipsychotic
2157	LY-294002	PI3K inhibitor, mTOR inhibitor, DNA dependent protein kinase inhibitor, phosphodiesterase inhibitor, PLK inhibitor
2158	ofloxacin	antibiotic that inhibits DNA gyrase, topoisomerase inhibitor
2159	palmitoylethanolamide	cannabinoid receptor agonist, Glucose-Dependent Insulinotropic Receptor (GDIR, GPR119) Agonists, unidentified pharmacological activity
2160	methylnorlichexanthone	Aurora kinase inhibitor, Pim kinase inhibitor, VEGFR inhibitor
2161	cephalosporanic-acid	core structure for synthesis of cephalosporin antibiotics
2162	L-701324	glutamate receptor antagonist
2163	ambroxol	glucosylceramidase inhibitor, mucolytic agent, sodium channel blocker
2164	BRD-K16604360	antiamyloidogenic agent, dopamine receptor agonist
2165	tetrahydrocannabinol-7-oic-acid	analgesic, anti-inflammatory
2166	EI-231	casein kinase inhibitor
2167	SRC-kinase-inhibitor-II	src inhibitor
2168	probenecid	MRP inhibitor, TRPV agonist, uricosuric blocker
2169	VU-0400071-3	glutamate receptor modulator
2170	GW-311616	leukocyte elastase inhibitor
2171	TPCA-1	IKK inhibitor
2172	RS-16566	(R)-zacopride ligand
2173	MMPX	phosphodiesterase inhibitor
2174	metolazone	carbonic anhydrase inhibitor, quinazoline diuretic, inhibitor of sodium chloride symporters
2175	PNU-22394	serotonin receptor agonist
2176	zileuton	leukotriene synthesis inhibitor, lipoxygenase inhibitor
2177	docetaxel	tubulin inhibitor, microtubule depolymerization inhibitor, microtubule stabilizing agent, microtubule stimulant, taxane
2178	FR-139317	endothelin receptor antagonist
2179	AVA	nucleophosmin inhibitor
2180	xanthoxyline	plant-derived antifungal
2181	carbamazepine	potassium channel modulator, Sodium Channel Blockers, sodium channel blocker, voltage-gated sodium channel blocker
2182	clopidogrel	purinergic receptor antagonist, platelet aggregation inhibitor, purinergic receptor inhibitor
2183	cycloheximide	glycogen synthase kinase inhibitor, protein synthesis inhibitor
2184	melperone	dopamine receptor antagonist, serotonin receptor antagonist
2185	deltaline	acetylcholine receptor antagonist
2186	karakoline	phytotoxin
2187	atovaquone	cytochrome b-c1 complex inhibitor, dihydroorotate dehydrogenase inhibitor, mitochondrial electron transport inhibitor
2188	CGS-21680	adenosine receptor agonist
2189	BRD-K87426499	caspase inhibitor
2190	buddleoflavonoloside	acetylcholinesterase inhibitor
2191	l-stepholidine	dopamine receptor antagonist, dopamine receptor agonist
2192	T-0156	phosphodiesterase inhibitor
2193	UBP-302	glutamate receptor antagonist
2194	CV-1808	adenosine receptor agonist
2195	KIN001-127	ITK inhibitor
2196	GR-144053	integrin antagonist
2197	4-(2-Amino-ethyl)-benzenesulfonamide	carbonic anhydrase inhibitor
2198	equilin	estrogen receptor agonist
2199	YM-298198	glutamate receptor antagonist
2200	fursultiamine	thiamine deriviative
2201	aspirin	cyclooxygenase inhibitor, nitric oxide donor, platelet aggregation inhibitor, thromboxane synthase inhibitor, TP53 expression enhancer
2202	l-erythro-MAPP	ceramidase inhibitor, negative control for D-erythro-MAPP
2203	MRS-1845	calcium channel blocker
2204	RO-3306	CDK inhibitor
2205	actarit	interleukin receptor agonist
2206	avrainvillamide-analog-1	nucleophosmin inhibitor
2207	GSK-1904529A	insulin growth factor receptor inhibitor, insulin receptor ligand
2208	ALX-5407	glycine transporter inhibitor, GlyT-1 inhibitor
2209	dolasetron	serotonin receptor antagonist
2210	tenovins	SIRT inhibitor, TP53 activator
2211	GR-79236	adenosine receptor agonist
2212	SU-6656	src inhibitor
2213	amisulpride	dopamine receptor antagonist

2214	GW-5074	RAF inhibitor, leucine rich repeat kinase inhibitor
2215	caffeine	adenosine receptor antagonist, ATM kinase inhibitor, ATR kinase inhibitor
2216	cefalexin	cell wall synthesis inhibitor
2217	treprostinil	prostacyclin analog
2218	quinpirole	dopamine receptor agonist
2219	robustic-acid	cAMP inhibitor
2220	CGP-13501	GABA receptor modulator
2221	phenylbutyrate	HDAC inhibitor, cell cycle inhibitor, pharmacological chaperone, pyruvate dehydrogenase kinase inhibitor
2222	atorvastatin	HMGCR inhibitor, dipeptidyl peptidase inhibitor, tumor necrosis factor expression inhibitor
2223	mifobate	cholesterol inhibitor, PPAR receptor antagonist, renin inhibitor
2224	PLX-4720	RAF inhibitor
2225	testosterone	androgen receptor (AR) agonist, androgen receptor agonist, testosterone receptor agonist
2226	cirazoline	alpha receptor agonist
2227	teicoplanin	bacterial cell wall synthesis inhibitor
2228	luzindole	melatonin receptor antagonist
2229	doxercalciferol	vitamin D receptor agonist
2230	BD-1008	sigma receptor antagonist
2231	fraxidin	carbonic anhydrase inhibitor
2232	BRD-K14236372	lysophospholipid receptor agonist
2233	lonidamine	glucokinase inhibitor, protein synthesis inhibitor
2234	erastin	anion channel modulator, ion channel antagonist
2235	EBPC	aldose reductase inhibitor
2236	BRD-K17693482	potassium channel activator
2237	AG-370	PDGFR tyrosine kinase receptor inhibitor, tyrosine kinase inhibitor
2238	bosentan	endothelin receptor antagonist
2239	clofibric-acid	PPAR receptor agonist
2240	n-(3-acetamidophenyl)-3-chlorobenzamide	glutamate receptor inhibitor
2241	testosterone	androgen receptor (AR) agonist, androgen receptor agonist, testosterone receptor agonist
2242	nifekalant	potassium channel antagonist, potassium channel blocker
2243	rizatriptan	serotonin receptor agonist
2244	estriol	estrogen receptor agonist, estrogen receptor antagonist
2245	ornidazole	antiprotozoal agent
2246	oxymetazoline	adrenergic receptor agonist, unidentified pharmacological activity
2247	terbutaline	adrenergic receptor agonist
2248	SC-19220	prostanoid receptor antagonist
2249	phentolamine	adrenergic receptor antagonist
2250	BRD-K48969316	caspase inhibitor
2251	vinpocetine	phosphodiesterase inhibitor, sodium channel blocker, voltage-sensitive sodium channel inhibitor
2252	montelukast	leukotriene receptor antagonist
2253	skimmianine	acetylcholinesterase inhibitor
2254	BRD-K14844937	GABA benzodiazepine site receptor inverse agonist
2255	mesoridazine	dopamine receptor antagonist
2256	streptozotocin	DNA alkylating drug
2257	gamma-homolinolenic-acid	cholesterol inhibitor, platelet aggregation inhibitor, prostaglandin G/H synthase 1, prostanoid receptor stimulant
2258	eriochrome-black-t	azo dye used in titrations to detect metal ions
2259	dipropyl-dopamine	dopamine receptor agonist
2260	HDAC1-selective	HDAC inhibitor
2261	BML-257	AKT inhibitor, HCV NS5B RdRp inhibitor
2262	IB-MECA	adenosine receptor agonist, granulocyte colony stimulating factor agonist
2263	NBQX	glutamate receptor antagonist, kainate receptor antagonist
2264	testosterone	androgen receptor (AR) agonist, androgen receptor agonist, testosterone receptor agonist
2265	NCS-382	GABA receptor antagonist
2266	chlordiazepoxide	GABA benzodiazepine site receptor agonist
2267	dexchlorpheniramine	histamine receptor antagonist
2268	brucine	glycine receptor antagonist
2269	AM-580	RAR agonist, retinoid receptor agonist
2270	ropinirole	dopamine receptor agonist, dopamine receptor modulator
2271	RS-67333	serotonin receptor partial agonist
2272	beta-CCP	indoleamine 2,3-dioxygenase inhibitor
2273	montelukast	leukotriene receptor antagonist
2274	propylthiouracil	thyroperoxidase inhibitor
2275	BRD-K13872703	chlorine/bicarbinate exchanger inhibitor, purinergic receptor antagonist
2276	coumestrol	AGE inhibitor, estrogen receptor agonist, estrogen receptor ligand
2277	lopinavir	HIV protease inhibitor
2278	cardiogenol-c	pyrimidine compound, induces embryonic stem cell ifferentiation into cardiomyocytes
2279	ciglitazone	PPAR receptor agonist, insulin sensitizer
2280	propoxycaine	anesthetic
2281	nemonapride	dopamine receptor antagonist, serotonin receptor agonist
2282	cortisone	glucocorticoid receptor agonist
2283	BRD-A83255679	estrogen receptor antagonist
2284	epicatechin	beta amyloid protein neurotoxicity inhibitor, beta secretase inhibitor, cyclooxygenase inhibitor, DNA gyrase inhibitor, DNA polymerase beta inhibitor, fatty acid synthase inhibitor, HCV inhibitor, sodium/glucose cotransporter inhib
2285	phenylbutazone	cyclooxygenase inhibitor, platelet aggregation inhibitor, prostanoid receptor inhibitor
2286	eugenol	androgen receptor (AR) inhibitor, free radical scavenger, monoamine oxidase inhibitor, quorum sensing signaling modulator
2287	tadalafil	phosphodiesterase inhibitor
2288	epitestosterone	inactive testosterone analog
2289	ryanodine	calcium channel inhibitor
2290	7,4'-dihydroxyflavone	opioid receptor antagonist

[illegible]	[illegible]	[illegible]
2291	PF-3845	FAAH inhibitor
2292	clofibrate	PPAR receptor agonist
2293	azithromycin	50S ribosomal subunit inhibitor, protein synthesis inhibitor
2294	esmolol	adrenergic receptor antagonist
2295	TAK-715	p38 MAPK inhibitor, tumor necrosis factor production inhibitor
2296	MPEP	glutamate receptor antagonist
2297	mephenytoin	cytochrome P450 substrate
2298	cefoxitin	cell wall synthesis inhibitor
2299	CDK2-5-inhibitor	inhibitor of CDK2/cyclin E and CDK5/p25
2300	AM-404	FAAH transport inhibitor, anandamide transport inhibitor, nucelar factor of activated T-cells inhibitor, TRPV agonist
2301	CL-82198	metalloproteinase inhibitor
2302	L-803087	somatostatin receptor agonist
2303	glycopyrrolate	acetylcholine receptor antagonist
2304	GW-441756	growth factor receptor inhibitor, leucine rich repeat kinase inhibitor
2305	10H-phenothiazin-10-yl)(p-tolyl)methanone	BCHE inhibitor
2306	ZD-7288	HCN (hyperpolarization activated cyclic nucleotide gated) channel blocker, HCN channel antagonist, potassium channel antagonist, sodium channel blocker
2307	SKF-89976A	GABA uptake inhibitor
2308	chloroquine	antimalarial agent
2309	pazopanib	VEGFR inhibitor, KIT inhibitor, PDGFR tyrosine kinase receptor inhibitor, angiogenesis inhibitor, RAF inhibitor, tyrosine kinase inhibitor, VEGFR antagonist
2310	pemoline	non-narcotic dopaminergic compound
2311	mephentermine	adrenergic receptor agonist
2312	lapatinib	EGFR inhibitor, EGFR antagonist, epidermal growth factor receptor (EGFR) inhibitor, receptor tyrosine protein kinase inhibitor, tyrosine kinase inhibitor
2313	biotin	vitamin cofactor for many metabolic reactions
2314	ecopipam	dopamine receptor antagonist
2315	cefaclor	cell wall synthesis inhibitor
2316	EI-273	PKC inhibitor
2317	propofol	membrane integrity inhibitor, membrane permeability inhibitor, TRPV agonist
2318	icosapent	fatty acid with anti-inflammatory, antithrombotic and immunomodulatory properties
2319	cephalotaxine	protein synthesis inhibitor
2320	naltrexone	opioid receptor antagonist, opioid receptor ligand
2321	M2-PK-activator	pyruvate kinase isozyme activator
2322	BH3I-1	BCL inhibitor
2323	leflunomide	dihydroorotate dehydrogenase inhibitor, PDGFR tyrosine kinase receptor inhibitor, disease modifying antirheumatic drug, immunosuppressant, JAK inhibitor, STAT inhibitor
2324	linezolid	50S ribosomal subunit inhibitor, protein synthesis inhibitor, monoamine oxidase inhibitor
2325	BRD-K51557114	GlyT-2 inhibitor
2326	naphazoline	adrenergic receptor agonist, peptide agonist
2327	nitrocaramiphen	acetylcholine receptor antagonist
2328	SB-205607	delta 1 opioid receptor agonist, opioid receptor agonist
2329	hydrastine	GABA receptor antagonist
2330	tandutinib	FLT3 inhibitor, KIT inhibitor, PDGFR tyrosine kinase receptor inhibitor
2331	NNC-63-0532	opioid receptor agonist
2332	BRD-A32917072	ROR antagonist
2333	equol	estrogen receptor agonist
2334	immethridine	histamine receptor agonist
2335	methazolamide	carbonic anhydrase inhibitor, insulin sensitizer
2336	desoxypeganine	acetylcholinesterase inhibitor, monoamine oxidase inhibitor
2337	BRD-K61106942	pyruvate kinase isozyme activator
2338	epibatidine	acetylcholine receptor agonist
2339	semaxanib	VEGFR inhibitor, angiogenesis inhibitor, FLT3 inhibitor, hepatocyte growth factor receptor inhibitor, vascular endothelial growth factor receptor (VEGFR) inhibitor, VEGFR antagonist
2340	alrestatin	aldose reductase inhibitor
2341	VX-745	p38 MAPK inhibitor
2342	TGX-221	PI3K inhibitor
2343	CPCCOEt	glutamate receptor antagonist
2344	phenothiazine	dopamine receptor antagonist
2345	altizide	thiazide diuretic
2346	I-OMe-AG-538	inhibitor of insulin growth factor 1 receptor protein tyrosine kinase
2347	oxybuprocaine	anesthetic
2348	BRD-K16618170	lysophospholipid receptor agonist
2349	farnesylthioacetic-acid	inhibitor of methyl esterification of farnesylated proteins
2350	hydroflumethiazide	sodium/potassium/chloride transporter inhibitor
2351	linoleic-acid	oxidative stress inducer
2352	acyclovir	DNA polymerase inhibitor, DNA directed DNA polymerase inhibitor
2353	BD-1047	alpha receptor antagonist, sigma receptor antagonist
2354	naltrexone	opioid receptor antagonist, opioid receptor ligand
2355	biperiden	anticholinergic
2356	5-methoxytryptamine	serotonin receptor agonist
2357	estradiol	estrogen receptor agonist
2358	salvinorin-a	opioid receptor agonist
2359	KU-0063794	mTOR inhibitor
2360	KIN001-055	EGFR inhibitor, JAK inhibitor, leukotriene synthesis inhibitor, mediator release inhibitor
2361	carmoxirole	dopamine receptor agonist
2362	quinine	cytochrome P450 inhibitor, hemozoin biocrystallization inhibitor, P glycoprotein inhibitor
2363	EMF-BCA1-64	caspase inhibitor
2364	thioproperazine	dopamine receptor antagonist
2365	selinidin	cumarin derivative with antiallergic properties
2366	isamoltan	adrenergic receptor antagonist
2367	RO-60-0175	serotonin receptor agonist

2367	RO-60-0175	serotonin receptor agonist
2368	methoprene-acid	RXR agonist
2369	O-1918	cannabinoid receptor antagonist
2370	procyclidine	anticholinergic
2371	deoxycholic-acid	biliverdin reductase A activator, G protein coupled receptor agonist, unidentified pharmacological activity
2372	guanabenz	adrenergic receptor agonist
2373	BML-ST330	phospholipase inhibitor, DNA inhibitor, RNA synthesis inhibitor
2374	L-2167	PPAR receptor agonist
2375	iobenguane	radiopharmaceutical and antineoplastic agent
2376	loreclezole	GABA receptor agonist
2377	noscapine	bradykinin receptor antagonist, tubulin polymerization inhibitor, apoptosis stimulant, microtubule inhibitor, tubulin inhibitor
2378	acetyl-farnesyl-cysteine	inhibitor of methylation of endogenous isoprenylated proteins
2379	beclometasone	glucocorticoid receptor agonist, corticosteroid hormone receptor agonist, immunosuppressant
2380	nitrendipine	calcium channel blocker, L-type calcium channel blocker
2381	ribavirin	antiviral guanosine ribonucleoside analog, IMPDH inhibitor, inosine monophosphate dehydrogenase inhibitor
2382	BP-554	serotonin receptor agonist
2383	varenicline	acetylcholine receptor partial agonist
2384	BRD-A18279961	pyruvate kinase isozyme activator
2385	eicosatrienoic-acid	vasodilator
2386	nicardipine	calcium channel blocker, L-type calcium channel blocker
2387	irsogladine	diuretic, phosphodiesterase inhibitor
2388	dibenzoylmethane	tumorigenesis inhibitor
2389	ranitidine	histamine receptor antagonist
2390	CGS-20625	benzodiazepine receptor agonist, GABA benzodiazepine site receptor partial agonist
2391	eugenitol	androgen receptor (AR) inhibitor, free radical scavenger, monoamine oxidase inhibitor, quorum sensing signaling modulator
2392	o-3M3FBS	phospholipase activator
2393	indoprofen	cyclooxygenase inhibitor, platelet aggregation inhibitor, prostanoid receptor inhibitor
2394	PD-168077	dopamine receptor agonist
2395	retinol	RAR receptor binder
2396	ephedrine	adrenergic receptor agonist
2397	proguanil	dihydrofolate reductase inhibitor
2398	PU-H71	HSP inhibitor
2399	carteolol	adrenergic receptor antagonist
2400	BIIB021	HSP inhibitor
2401	flucloxacillin	antibiotic of the penicillin class
2402	decafluorobutane	ultrasound contrast agent
2403	dasatinib	KIT inhibitor, src inhibitor, Bcr-Abl kinase inhibitor, ephrin receptor inhibitor, PDGFR tyrosine kinase receptor inhibitor, yes kinase inhibitor, Abl kinase inhibitor, Bruton's tyrosine kinase (BTK) inhibitor, discoidin domain containin
2404	pirinixic-acid	PPAR receptor agonist
2405	fluorometholone	corticosteroid agonist, glucocorticoid receptor agonist
2406	mead-ethanolamide	cannabinoid receptor agonist
2407	tropisetron	serotonin receptor antagonist, polarization inhibitor, sodium channel blocker
2408	fludrocortisone	glucocorticoid receptor agonist, mineralocorticoid receptor agonist
2409	LFM-A12	EGFR inhibitor, epidermal growth factor receptor (EGFR) inhibitor
2410	tetracycline	30S ribosomal protein inhibitor, 30S ribosomal subunit inhibitor, protein arginine deiminase inhibitor
2411	perospirone	dopamine receptor antagonist, serotonin receptor antagonist
2412	zolantidine	histamine receptor antagonist
2413	KIN001-242	protein kinase inhiibor
2414	acetyl-geranygeranyl-cysteine	inhibitor of methyl esterification of geranylgeranylated proteins
2415	reserpic-acid	norepinephrine transporter inhibitor
2416	entacapone	catechol O methyltransferase inhibitor
2417	tianeptine	selective serotonin reuptake enhancer (SSRE), serotonin reuptake enhancer, serotonin uptake enhancer
2418	ochratoxin-a	phenylalanyl tRNA synthetase inhibitor
2419	pinacidil	ATP channel activator, potassium channel agonist
2420	EMF-bca1-60	caspase inhibitor
2421	pipamperone	dopamine receptor, dopamine receptor antagonist, serotonin receptor antagonist
2422	dubinidine	anticonvulsant that reduces motor activity
2423	tretinoin	RAR agonist, RAR receptor binder, retinoid receptor agonist, ROR ligand
2424	GDC-0879	RAF inhibitor
2425	tozasertib	Aurora kinase inhibitor, Bcr-Abl kinase inhibitor, FLT3 inhibitor, JAK inhibitor, Abl kinase inhibitor, mitotic inhibitor
2426	caffeic-acid	lipoxygenase inhibitor, HIV integrase inhibitor, NFkB pathway inhibitor, nitric oxide production inhibitor, PPAR receptor modulator, tumor necrosis factor production inhibitor
2427	dichloroacetic-acid	pyruvate dehydrogenase kinase inhibitor, pyruvate dehydrogenase stimulant
2428	tolazoline	adrenergic receptor antagonist
2429	BMS-299897	gamma secretase inhibitor, antiamyloidogenic agent
2430	BRD-K19985802	protein phosphatase inhibitor
2431	methyllycaconitine	acetylcholine receptor antagonist
2432	FG-7142	GABA benzodiazepine site receptor inverse agonist
2433	dexamethasone	glucocorticoid receptor agonist, corticosteroid agonist, immunosuppressant
2434	stavudine	DNA directed DNA polymerase inhibitor, nucleoside reverse transcriptase inhibitor, reverse transcriptase inhibitor
2435	ZD-2079	adrenergic receptor agonist, insulin sensitizer
2436	ergocornine	dopamine receptor agonist
2437	dobutamine	adrenergic receptor agonist
2438	doxepin	histamine receptor antagonist, adrenergic transmitter uptake inhibitor, histamine receptor inhibitor, norepinephrine reputake inhibitor, serotonin reuptake inhibitor, tricyclic antidepressant
2439	RS-67506	serotonin receptor partial agonist
2440	aniracetam	glutamate receptor agonist
2441	damnacanthal	src inhibitor
2442	R-96544	serotonin receptor antagonist
2443	SB-366791	TRPV antagonist
2444	ALW-II-38-3	ephrin inhibitor

2444	ALW-II-38-3	ephrin inhibitor
2445	BRD-K49061529	GABA benzodiazepine site receptor inverse agonist
2446	RG-13022	EGFR inhibitor, PDGFR tyrosine kinase receptor inhibitor
2447	harpagoside	acetylcholinesterase inhibitor
2448	cefotaxime	cell wall synthesis inhibitor
2449	mestranol	estrogen receptor agonist
2450	LY-255283	leukotriene receptor antagonist
2451	RO-19-4605	GABA benzodiazepine site receptor inverse agonist
2452	SB-525334	TGF beta receptor inhibitor
2453	GSK-1070916	Aurora kinase inhibitor
2454	metrizamide	iodine contrast agent
2455	coumarin	vitamin antagonist
2456	tiabendazole	quinol-fumarate reductase inhibitor
2457	apigenin	casein kinase inhibitor, cell proliferation inhibitor, cytochrome P450 inhibitor, GABA receptor antagonist, glutamate receptor antagonist, monoamine oxidase inhibitor, nitric oxide production inhibitor, ornithine decarboxylase inhil
2458	homoveratrylamine	dopamine analog
2459	LY-456236	glutamate receptor antagonist
2460	rivaroxaban	coagulation factor inhibitor, coagulation inhibitor, serine protease inhibitor
2461	BRD-K55186349	apelin receptor antagonist
2462	vinburnine	adrenergic receptor antagonist
2463	HG-5-88-01	protein kinase inhibitor
2464	physostigmine	acetylcholinesterase inhibitor, cholinesterase inhibitor
2465	anpirtoline	serotonin receptor agonist, serotonin receptor antagonist
2466	austricine	hypolipidemic
2467	estriol	estrogen receptor agonist, estrogen receptor antagonist
2468	alfadolone	GABA receptor agonist
2469	SRT-1720	SIRT activator
2470	troglitazone	PPAR receptor agonist, insulin sensitizer, CCK ligand expression inhibitor, EGR1 expression enhancer, glycogen synthase kinase stimulant
2471	glycodeoxycholic-acid	apoptosis activator
2472	pseudoephedrine	histamine receptor antagonist, interleukin expression inhibitor, tumor necrosis factor expression inhibitor
2473	BMY-14802	sigma receptor antagonist, glutamate receptor antagonist, serotonin receptor agonist
2474	homatropine	acetylcholine receptor antagonist
2475	capecitabine	dihydropyrimidine dehydrogenase inhibitor, DNA synthesis inhibitor, pyrimidine antagonist, RNA synthesis inhibitor, thymidylate synthase inhibitor
2476	rosuvastatin	HMGCR inhibitor
2477	pregnenolone	acetylcholine release enhancer, dopamine release enhancer, GABA receptor negative allosteric modulator, glutamate receptor modulator, progesterone receptor agonist, steroid hormone inhibitor of CYP17A1 and SULT2B1
2478	ST-638	tyrosine kinase inhibitor
2479	protriptyline	tricyclic antidepressant (TCA)
2480	BRD-K64314806	lipoxygenase inhibitor
2481	finasteride	5 alpha reductase inhibitor, androgen receptor antagonist, steroid 5alpha-reductase inhibitor
2482	SB-239063	p38 MAPK inhibitor, interleukin inhibitor, tumor necrosis factor production inhibitor
2483	LY-320135	cannabinoid receptor antagonist
2484	OM-137	Aurora kinase inhibitor
2485	U-0126	MEK inhibitor, JAK inhibitor, MAP kinase inhibitor
2486	tyrphostin-AG-1295	FLT3 inhibitor, PDGFR tyrosine kinase receptor inhibitor
2487	fenoterol	adrenergic receptor agonist
2488	BRL-15572	serotonin receptor antagonist
2489	bucladesine	adenosine receptor agonist, cAMP stimulant, vasodilator
2490	cinacalcet	calcimimetic that activates calcium-sensing receptors
2491	nimodipine	calcium channel blocker, L-type calcium channel blocker
2492	scopolamine	acetylcholine receptor antagonist
2493	aminomethyltransferase	nitric oxide synthase inhibitor
2494	danoprevir	HCV inhibitor, serine protease inhibitor
2495	seneciphylline	cytochrome P450 inhibitor
2496	BRD-A38793261	ROR antagonist
2497	ketanserin	serotonin receptor antagonist, collagen stimulant
2498	VU-0415374-1	glutamate receptor modulator
2499	arecaidine	acetylcholine receptor agonist
2500	TTNPB	RAR agonist, retinoid receptor agonist
2501	tubocurarine	acetylcholine receptor antagonist
2502	benzanthrone	aromatic hydrocarbon derivate with fluorescent and luminescent properties
2503	BRD-K76211160	cytochrome P450 inhibitor
2504	BRD-K91485395	dual specificity protein phosphatase inhibitors
2505	maprotiline	norepinephrine reputake inhibitor, tricyclic antidepressant (TCA)
2506	pindolol	adrenergic receptor antagonist
2507	dienestrol	estrogen receptor agonist
2508	benazepril	angiotensin converting enzyme inhibitor
2509	norketamine	glutamate receptor antagonist
2510	resveratrol	apolipoprotein expression enhancer, beta-secretase inhibitor, cyclooxygenase inhibitor, cytochrome P450 inhibitor, lipid peroxidase inhibitor, MAP kinase inhibitor, monoamine oxidase inhibitor, NFkB pathway modulator, SIRT a
2511	VUF-5681	histamine receptor antagonist
2512	diphenidol	acetylcholine receptor ligand
2513	BRD-K97951054	Aurora kinase inhibitor, Pim kinase inhibitor, VEGFR inhibitor
2514	praziquantel	membrane permeability enhancer, schistosomicide
2515	levetiracetam	acetylcholine receptor agonist, N-type calcium channel blocker, synaptic vesicle glycoprotein ligand
2516	BMS-191011	large conductance calcium activated potassium channel opener, large conductance potassium channel activator
2517	hydralazine	smooth muscle relaxant
2518	MDL-29951	glutamate receptor antagonist, fructose biphosphate inhibitor, UDP/CysLT (GPR17, P2Y-like) Agonists
2519	PJ-34	PARP inhibitor
2520	WZ-4002	EGFR inhibitor

2521	betamethasone	corticosteroid agonist, glucocorticoid receptor agonist
2522	BRD-A41630653	glutamate receptor antagonist, sigma receptor agonist
2523	etamivan	respiratory stimulant
2524	NSC-23766	RAC1 GTPase inhibitor
2525	ritodrine	adrenergic receptor agonist
2526	MDL-11939	serotonin receptor antagonist
2527	propantheline	acetylcholine receptor antagonist
2528	picotamide	prostanoid receptor antagonist, thromboxane receptor antagonist, thromboxane synthase inhibitor
2529	EI-247	insulin growth factor receptor inhibitor
2530	naftifine	squalene epoxidase inhibitor, steroid sulfatase inhibitor
2531	levcromakalim	potassium channel activator, potassium channel agonist
2532	naloxone	opioid receptor antagonist
2533	MK-5108	Aurora kinase inhibitor
2534	toremifene	selective estrogen receptor modulator (SERM), estrogen receptor antagonist
2535	saracatinib	src inhibitor, Abl kinase inhibitor
2536	BRD-K34437622	thymidylate synthase inhibitor
2537	zalcitabine	nucleoside reverse transcriptase inhibitor, reverse transcriptase inhibitor
2538	XAV-939	PARP inhibitor, TNKS inhibitor
2539	ML-9	myosin light chain kinase inhibitor
2540	ibuprofen	cyclooxygenase inhibitor, NFkB pathway inhibitor
2541	PF-04217903	c-Met inhibitor, hepatocyte growth factor receptor inhibitor
2542	hydrocortisone	corticosteroid agonist, glucocorticoid receptor agonist, immunosuppressant, interleukin receptor antagonist
2543	quinpirole	dopamine receptor agonist
2544	voriconazole	cell wall synthesis inhibitor, cytochrome P450 inhibitor, lanosterol demethylase inhibitor
2545	tubacin	HDAC inhibitor
2546	PD-166793	collagenase inhibitor, metalloproteinase inhibitor, stromelysin inhibitor
2547	ZM-323881	vascular endothelial growth factor receptor 2 (VEGFR2) inhibitor, VEGFR inhibitor
2548	JNJ-16259685	glutamate receptor antagonist
2549	phenytoin	sodium channel blocker
2550	quinisocaine	anesthetic
2551	pyridine-2-aldoxime	acetylcholinesterase inhibitor
2552	buphenine	adrenergic receptor agonist
2553	levocabastine	histamine receptor antagonist
2554	doxylamine	histamine receptor antagonist
2555	sulfinpyrazone	platelet aggregation inhibitor
2556	chromanol	potassium channel blocker
2557	1-monopalmitin	P glycoprotein inhibitor
2558	propranolol	adrenergic receptor antagonist
2559	3-methyl-GABA	GABA aminotransferase activator
2560	BRD-K98297262	1,3-beta-glucan synthase inhibitor
2561	BRD-A61599461	thyroid-stimulating hormone receptor inverse agonist
2562	estrone	estrogen receptor agonist, estrogenic hormone
2563	flurofamide	bacterial urease inhibitor
2564	CGP-54626	GABA receptor antagonist
2565	lupanine	ATP channel blocker, insulin secretagogue, Sodium Channel Blockers, sodium channel blocker
2566	sirolimus	mTOR inhibitor, CCR expression inhibitor, cell cycle inhibitor, proteasome inhibitor, protein kinase inhibitor, T cell inhibitor
2567	etomoxir	carnitine palmitoyltransferase inhibitor, carnitine O-palmitoyltransferase inhibitor, fatty acid oxidation inhibitor
2568	oxfendazole	anthelmintic agent
2569	NM-PP1	calcium/calmodulin dependent protein kinase inhibitor, growth factor receptor inhibitor, MAP kinase inhibitor, mutant kinase inhibitor
2570	erythromycin	NFkB pathway inhibitor, 50S ribosomal subunit inhibitor, motilin receptor agonist, RPLV inhibitor
2571	phylloquinone	antifibrinolytic agent, gamma carboxylase enzyme
2572	paclitaxel	tubulin inhibitor, microtubule stabilizing agent, microtubule stimulant, P glycoprotein inhibitor, taxane
2573	alpha-linolenic-acid	omega-3 fatty acid
2574	LY-341495	glutamate receptor antagonist
2575	ilomastat	matrix metalloprotease inhibitor
2576	sulfacetamide	antibiotic
2577	phenacetin	cyclooxygenase inhibitor
2578	moxifloxacin	DNA gyrase inhibitor, topoisomerase inhibitor
2579	foliosidine	anticonvulsant
2580	EMD-386088	serotonin receptor agonist
2581	tofacitinib	JAK inhibitor, disease modifying antirheumatic drug, immunosuppressant, MAP kinase inhibitor, tyrosine kinase inhibitor
2582	minoxidil	antialopecia agent, ATP channel activator, ATP-sensitive potassium channel agonist, KATP activator, Kir6 channel (KATP) activator, polarization activator, renin secretion stimulant
2583	T-0070907	PPAR receptor anatagonist, PPAR receptor antagonist
2584	umbelliferone	carbonic anhydrase inhibitor, cyclooxygenase inhibitor
2585	hydrocortisone	corticosteroid agonist, glucocorticoid receptor agonist, immunosuppressant, interleukin receptor antagonist
2586	RHO-kinase-inhibitor-III[rockout]	ROCK inhibitor
2587	terazosin	adrenergic receptor antagonist
2588	mirtazapine	adrenergic receptor antagonist, serotonin receptor antagonist
2589	glimepiride	insulin secretagogue, ATP channel blocker, ATP-sensitive potassium channel antagonist, insulinotropin agonist, sulfonylurea
2590	bucladesine	adenosine receptor agonist, cAMP stimulant, vasodilator
2591	amcinonide	corticosteroid agonist, cytochrome P450 inhibitor, glucocorticoid receptor agonist, immunosuppressant
2592	diethylstilbestrol	estrogen receptor agonist, chloride channel blocker
2593	lidocaine	histamine receptor agonist, voltage-gated sodium channel modulator
2594	BRD-K19059335	runt-related transcription factor inhibitor, tyrosine aminotransferase inhibitor
2595	HO-013	PPAR receptor agonist
2596	U-54494A	kappa opioid agonist
2597	deforolimus	mTOR inhibitor, angiogenesis inhibitor, cell cycle inhibitor, immunosuppressant, protein kinase inhibitor, serine/threonine kinase inhibitor, VEGFR antagonist

2598	RS-102221	serotonin receptor antagonist
2599	forskolin	adenylyl cyclase activator, Adenylate cyclase stimulant, growth hormone receptor agonist, phosphokinase stimulant
2600	tiotidine	histamine receptor antagonist
2601	sulindac	ABC transporter expression enhancer, cyclooxygenase inhibitor, NFkB pathway inhibitor
2602	QX-314	sodium channel blocker
2603	forskolin	adenylyl cyclase activator, Adenylate cyclase stimulant, growth hormone receptor agonist, phosphokinase stimulant
2604	spiroxatrine	serotonin receptor antagonist
2605	lobelanidine	acetylcholine receptor antagonist, dopamine receptor modulator, opioid receptor antagonist, vesicular monoamine transporter ligand
2606	cytosporone-b	HMR receptor agonist
2607	BRD-K58033748	acetylcholine receptor antagonist
2608	myricetin	androgen receptor ligand, cytochrome P450 inhibitor
2609	PKCbeta-inhibitor	PKC inhibitor
2610	QW-BI-011	histone lyinse methyltransferase inhibitor
2611	benzydamine	membrane integrity inhibitor, prostanoid receptor inhibitor
2612	raltegravir	HIV integrase inhibitor
2613	tramadol	opioid receptor agonist, norepinephrine reputake inhibitor, serotonin reuptake inhibitor, adrenergic transmitter uptake inhibitor, OP3 receptor agonist, serotonin uptake inhibitor
2614	L-689560	glutamate receptor antagonist
2615	orciprenaline	adrenergic receptor agonist
2616	tyrphostin-B44	EGFR inhibitor
2617	etodolac	cyclooxygenase inhibitor, TRPV agonist
2618	IKK-2-inhibitor	IKK inhibitor, syk inhibitor
2619	darinaparsin	apoptosis stimulant, NADPH oxidase stimulant
2620	TWS-119	glycogen synthase kinase inhibitor
2621	BRD-K63784565	beta amyloid synthesis inhibitor, topoisomerase inhibitor
2622	fludroxycortide	corticosteroid, glucocorticoid receptor agonist
2623	warfarin	cytochrome P450 inhibitor, vitamin inhibitor, vitamin K epoxide reductase inhibitor
2624	sinensetin	cyclooxygenase inhibitor
2625	scoulerine	adrenergic receptor antagonist, GABA receptor antagonist, serotonin receptor antagonist
2626	AS-703026	MEK inhibitor
2627	bosutinib	src inhibitor, Abl kinase inhibitor, Bcr-Abl kinase inhibitor, apoptosis stimulant, STAT inhibitor
2628	catechin	beta secretase inhibitor, fatty acid synthase inhibitor, free radical scavenger, immunostimulant, LDL antioxidants, quorum sensing signaling modulator, reducing agent, sodium channel blocker
2629	zaprinast	phosphodiesterase inhibitor, histamine release inhibitor
2630	SB-431542	TGF beta receptor inhibitor, ALK inhibitor
2631	DMEOB	glutamate receptor modulator
2632	latrepirdine	glutamate receptor antagonist, histamine receptor antagonist, serotonin receptor antagonist
2633	KIN001-244	phosphoinositide dependent kinase inhibitor
2634	2',5'-dideoxyadenosine	adenylyl cyclase inhibitor
2635	carbofuran	cholinesterase inhibitor
2636	hyoscyamine	acetylcholine receptor inhibitor
2637	NSC-94258	antitumor agent
2638	tyrphostin-1	epidermal growth factor receptor (EGFR) inhibitor
2639	olanzapine	dopamine receptor antagonist, serotonin receptor antagonist
2640	gefitinib	EGFR inhibitor
2641	1,2-dichlorobenzene	hepatotoxicant that induces oxidative stress and inflammatory response
2642	austricine	hypolipidemic
2643	FTI-276	farnesyltransferase inhibitor
2644	temozolomide	DNA alkylating drug, DNA damage inducer, DNA inhibitor, topoisomerase inhibitor
2645	ZSTK-474	PI3K inhibitor
2646	mupirocin	isoleucyl-tRNA synthetase inhibitor
2647	BRL-50481	phosphodiesterase inhibitor
2648	AS-604850	Phosphatidylinositol 3-kinase (PI3K) inhibitor, PI3K inhibitor
2649	flunisolide	cytochrome P450 inhibitor, corticosteroid agonist, corticosteroid hormone receptor agonist, glucocorticoid receptor agonist, immunosuppressant
2650	remoxipride	dopamine receptor antagonist
2651	7,8-dihydro-L-biopterin	dihydroneopterin aldolase inhibitor
2652	RS-56812	serotonin receptor antagonist, serotonin receptor partial agonist
2653	PNU-120596	acetylcholine receptor modulator, acetylcholine receptor positive allosteric modulator
2654	oligomycin-a	ATP synthase inhibitor, ATPase inhibitor
2655	RITA	MDM inhibitor, thioredoxin reductase inhibitor
2656	cobalt(II)-chloride	HSP agonist
2657	ipratropium	acetylcholine receptor antagonist
2658	taurodeoxycholic-acid	bile acid
2659	NU-1025	PARP inhibitor, DNA dependent protein kinase inhibitor
2660	eplerenone	cytochrome P450 antagonist, mineralocorticoid receptor antagonist
2661	quizartinib	FLT3 inhibitor, colony stimulating factor receptor inhibitor, KIT inhibitor, RET tyrosine kinase inhibitor
2662	rolipram	phosphodiesterase inhibitor, interleukin receptor antagonist
2663	VU-0400193-3	glutamate receptor modulator
2664	PIT	purinergic receptor antagonist
2665	SJ-172550	MDM inhibitor
2666	perindopril	angiotensin converting enzyme inhibitor
2667	NU-7026	DNA dependent protein kinase inhibitor, mTOR inhibitor, PI3K inhibitor
2668	sildenafil	phosphodiesterase inhibitor
2669	IWR-1-ENDO	PARP inhibitor
2670	fluoropyruvate	PDH inhibitor
2671	UB-165	acetylcholine receptor agonist
2672	BRD-K52219182	phosphodiesterase inhibitor
2673	carbenoxolone	11-beta hydroxysteroid dehydrogenase inhibitor
2674	androstenol	GABA receptor modulator

2674	[illegible]	[illegible]
2675	BRD-A80383043	glutamate receptor agonist, glutamate receptor antagonist
2676	JZL-184	monoacylglucerol lipase inhibitor
2677	ethambutol	bacterial arabinosyl transferase inhibitor
2678	sulmazole	calcium sensitizer, phosphodiesterase inhibitor
2679	SB-206553	serotonin receptor antagonist, serotonin receptor partial agonist
2680	orantinib	PDGFR tyrosine kinase receptor inhibitor, FGFR inhibitor, VEGFR inhibitor, angiogenesis inhibitor, apoptosis stimulant, Aurora kinase inhibitor, FGFR antagonist, fibroblast growth factor receptor (FGFR) antagonist, fibroblast g
2681	PT-630	dipeptidyl peptidase inhibitor, fibroblast activation protein inhibitor
2682	oxalomalic-acid	isocitrate dehydrogenase inhibitor
2683	retinol	RAR receptor binder
2684	parecoxib	cyclooxygenase inhibitor
2685	pepstatin	aspartic protease inhibitor
2686	fillalbin	increases arterial blood pressure
2687	ganglioside	src activator
2688	cilomilast	phosphodiesterase inhibitor, interleukin synthesis inhibitor, tumor necrosis factor production inhibitor
2689	tegafur	DNA synthesis inhibitor, thymidylate synthase inhibitor
2690	ICI-89406	adrenergic receptor antagonist
2691	SKF-96365	calcium channel blocker, cytochrome P450 inhibitor
2692	BRD-A81377415	CLK inhibitor, dual specificity tyrosine-(Y)-phosphorylation regulated kinase inhibitor, dual-specificity tyrosine-(Y)-phosphorylation regulated kinase 1B inhibitor
2693	piperacillin	cell wall synthesis inhibitor
2694	brivanib	alcohol dehydrogenase inhibitor, FGFR inhibitor, fibroblast growth factor receptor (FGFR) inhibitor, vascular endothelial growth factor receptor (VEGFR) inhibitor, VEGFR inhibitor
2695	AICA-ribonucleotide	adenosine release stimulant, AMPK stimulant
2696	SB-221284	serotonin receptor antagonist
2697	norepinephrine	adrenergic receptor agonist, DNA inhibitor, free radical scavenger
2698	KU-55933	ATM kinase inhibitor
2699	WZ-3146	EGFR inhibitor, epidermal growth factor receptor (EGFR) inhibitor
2700	enalapril	angiotensin converting enzyme inhibitor, angiotensin receptor blocker
2701	tyrphostin-AG-1288	catechol O methyltransferase inhibitor, tumor necrosis factor inhibitor
2702	carprofen	cyclooxygenase inhibitor, prostanoid receptor inhibitor
2703	RS-100329	adrenoceptor antagonist
2704	benzamil	epithelial sodium channel blocker
2705	BRD-K75430629	PI3K inhibitor
2706	BRD-K76425766	metalloproteinase inhibitor
2707	KUC104502N	opioid receptor antagonist
2708	lenalidomide	angiogenesis inhibitor, cereblon inhibitor, tumor apoptosis inducer, tumor necrosis factor production inhibitor, tumor necrosis factor receptor antagonist
2709	tetramethylsilane	organosilicon compound used as an internal standard in NMR spectroscopy
2710	BRD-K57954781	apoptosis stimulant, DNA inhibitor
2711	tyrphostin-AG-1296	FLT3 inhibitor
2712	verapamil	calcium channel blocker, L-type calcium channel blocker, dopamine receptor antagonist
2713	BRD-K79511609	adenosine receptor agonist
2714	hemado	adenosine receptor agonist
2715	acetyl-geranyl-cysteine	Ras carboxyl methylation inhibitor
2716	amiloride	sodium channel blocker, diuretic, epithelial sodium channel blocker, sodium/hydrogen exchanger inhibitor
2717	tetradecylthioacetic-acid	lipid peroxidase inhibitor
2718	clebopride	dopamine receptor antagonist
2719	farnesol	amine oxidase B inhibitor, FXR agonist
2720	AZ-10417808	caspase inhibitor
2721	lapatinib	EGFR inhibitor, EGFR antagonist, epidermal growth factor receptor (EGFR) inhibitor, receptor tyrosine protein kinase inhibitor, tyrosine kinase inhibitor
2722	memantine	glutamate receptor antagonist, glutamate release inhibitor
2723	LY-364947	TGF beta receptor inhibitor, p38 MAPK inhibitor
2724	dexamethasone	glucocorticoid receptor agonist, corticosteroid agonist, immunosuppressant
2725	VEGF-receptor-2-kinase-inhibitor-IV	VEGFR inhibitor
2726	SAL-1	adenosine receptor antagonist
2727	BRD-K63954456	macrophage migration inhibiting factor inhibitor, nitric oxide production inhibitor, tumor necrosis factor production inhibitor
2728	tyrphostin-51	EGFR inhibitor
2729	cyclopenthiazide	diuretic, inhibitor of sodium chloride symporter
2730	VU-0420363-1	SARS coronavirus 3C-like protease inhibitor
2731	imiquimod	interferon inducer, toll-like receptor agonist, immunostimulant
2732	prunetin	breast cancer resistance protein inhibitor
2733	maraviroc	CC chemokine receptor antagonist, CC chemokine receptor 5 (CCR5) antagonist, reverse transcriptase inhibitor
2734	neurodazine	neurogenesis of non-pluripotent C2C12 myoblast inducer
2735	BMS-641988	androgen receptor antagonist
2736	hydrastinine	alkaloid with hemostatic properties
2737	noscapine	bradykinin receptor antagonist, tubulin polymerization inhibitor, apoptosis stimulant, microtubule inhibitor, tubulin inhibitor
2738	L-733060	tachykinin antagonist
2739	vecuronium	acetylcholine receptor antagonist
2740	solanine	acetylcholinesterase inhibitor
2741	evodiamine	ATPase inhibitor, TRPV agonist
2742	ZM-306416	Src and Abl inhibitor, vascular endothelial growth factor receptor 1 (VEGFR1) inhibitor
2743	flupirtine	glutamate receptor antagonist, apoptosis inhibitor, opioid receptor agonist, potassium channel agonist
2744	colforsin	adenylyl cyclase activator, Adenylate cyclase stimulant, growth hormone receptor agonist, phosphokinase stimulant
2745	BRD-K15107389	ribosomal protein inhibitor
2746	myriocin	sphingolipid biosynthesis inhibitor
2747	BRL-37344	adrenergic receptor agonist
2748	BAY-K8644	L-type calcium channel activator
2749	EMF-bca1-57	caspase inhibitor
2750	linsitinib	insulin growth factor receptor inhibitor, insulin receptor antagonist
2751	triacsin-c	adrenergic receptor antagonist

2751	[illegible]	adrenergic receptor antagonist
2752	iloperidone	dopamine receptor antagonist, serotonin receptor antagonist
2753	AC-55649	RAR agonist, retinoid receptor agonist
2754	GW-583340	EGFR inhibitor, epidermal growth factor receptor (EGFR) inhibitor, ErbB2 tyrosine kinase inhibitor, receptor tyrosine protein kinase inhibitor
2755	hydroxyfasudil	ROCK inhibitor
2756	FR-180204	MAP kinase inhibitor
2757	safinamide	dopamine reuptake inhibitor, glutamate release inhibitor, monoamine oxidase inhibitor, calcium channel antagonist, calcium channel modulator, Sodium Channel Blockers, sodium channel blocker
2758	nikkomycin	chitinase inhibitor
2759	prima-1-met	thioredoxin inhibitor, TP53 activator
2760	trioxsalen	DNA synthesis inhibitor
2761	peucedanin	apoptosis inducer, antileukemic
2762	UNC-0321	histone lyinse methyltransferase inhibitor, histone lysine methyltransferase inhibitor
2763	prostaglandin-a1	HSP inducer, NFKB pathway inhibitor
2764	BRD-K30064966	caspase activator
2765	rilmenidine	adrenergic receptor agonist, imidazoline receptor agonist
2766	WAY-213613	glutamate transporter inhibitor
2767	BRD-K70693222	CLK inhibitor, dual specificity tyrosine-(Y)-phosphorylation regulated kinase inhibitor
2768	U-0124	MEK inhibitor
2769	cimaterol	adrenergic receptor agonist
2770	Y-27632	ROCK inhibitor, calcium sensitizer, leucine rich repeat kinase inhibitor
2771	huperzine-a	acetylcholinesterase inhibitor, glutamate receptor antagonist
2772	methantheline	acetylcholine receptor antagonist
2773	3-amino-benzamide	PARP inhibitor
2774	taurocholic-acid	bile acid
2775	palonosetron	serotonin receptor antagonist
2776	cholic-acid	ferrochelatase inhibitor, unidentified pharmacological activity
2777	dexamethasone	glucocorticoid receptor agonist, corticosteroid agonist, immunosuppressant
2778	AZ-628	RAF inhibitor
2779	AS-605240	Phosphatidylinositol 3-kinase (PI3K) inhibitor, PI3K inhibitor
2780	3,3'-diindolylmethane	androgen receptor antagonist, aryl hydrocarbon receptor agonist, beta glucuronidase inhibitor, CHK inhibitor, cytochrome P450 activator, indoleamine 2,3-dioxygenase inhibitor
2781	TTNPB	RAR agonist, retinoid receptor agonist
2782	curcumin	cyclooxygenase inhibitor, AP inhibitor, CCN expression inhibitor, DNA methyltransferase inhibitor, EGFR expression inhibitor, free radical scavenger, FtsZ inhibitor, glucose 6 phosphatase inhibitor, histone N-acetyltransferase i[illegible]
2783	deferiprone	chelating agent, cytochrome P450 inhibitor, iron absorption inhibitor, reducing agent
2784	navitoclax	BCL inhibitor, apoptosis stimulant
2785	MLN-8054	Aurora kinase inhibitor, mitotic inhibitor, protein kinase inhibitor
2786	WH-4023	src inhibitor
2787	AGK-2	SIRT inhibitor, SIRT pathway
2788	forskolin	adenylyl cyclase activator, Adenylate cyclase stimulant, growth hormone receptor agonist, phosphokinase stimulant
2789	BRD-K64835161	CLK inhibitor, dual specificity tyrosine-(Y)-phosphorylation regulated kinase inhibitor, dual-specificity tyrosine-(Y)-phosphorylation regulated kinase 1B inhibitor
2790	AR-C133057XX	nitric oxide synthase inhibitor
2791	atorvastatin	HMGCR inhibitor, dipeptidyl peptidase inhibitor, tumor necrosis factor expression inhibitor
2792	NECA	adenosine receptor agonist
2793	tipifarnib-P2	farnesyltransferase inhibitor, angiogenesis inhibitor, apoptosis stimulant
2794	vemurafenib	RAF inhibitor, protein kinase inhibitor
2795	fatostatin	sterol regulatory element binding protein (SREBP) inhibitor
2796	selumetinib	MEK inhibitor, MAP kinase inhibitor
2797	sitagliptin	dipeptidyl peptidase inhibitor, HMGCR inhibitor, insulin secretagogue, tumor necrosis factor expression inhibitor
2798	BRD-K07872006	lipoxygenase inhibitor
2799	valproic-acid	HDAC inhibitor, ABAT inhibitor, GABA receptor agonist, GABAergic transmission enhancer, voltage-gated sodium channel blocker
2800	PD-184352	MEK inhibitor, MAP kinase inhibitor
2801	geranylgeraniol	farnesyltransferase inhibitor
2802	TG100-115	PI3K inhibitor
2803	AZD-6482	PI3K inhibitor
2804	9-methyl-5H-6-thia-4,5-diaza-chrysene-6,6-dioxide	NFkB pathway inhibitor
2805	BRD-K59222562	CLK inhibitor, dual specificity tyrosine-(Y)-phosphorylation regulated kinase inhibitor, dual-specificity tyrosine-(Y)-phosphorylation regulated kinase 1B inhibitor
2806	Cyclo-[Arg-Gly-Asp-D-Phe-Val]	integrin antagonist
2807	PP-1	src inhibitor, Abl kinase inhibitor
2808	tyrphostin-AG-112	protein tyrosine kinase inhibitor
2809	MK-1775	wee1 kinase inhibitor
2810	GSK-429286A	ROCK inhibitor
2811	NF-449	purinergic receptor antagonist
2812	VER-155008	HSP inhibitor
2813	phensuximide	anticonvulsant
2814	MEK1-2-inhibitor	MEK inhibitor
2815	L-690330	inositol monophophatase inhibitor
2816	PD-0325901	MEK inhibitor, MAP kinase inhibitor, protein kinase inhibitor
2817	PIK-90	PI3K inhibitor
2818	carbetocin	analogue of oxytocin
2819	tivozanib	VEGFR inhibitor, KIT inhibitor, tyrosine kinase inhibitor
2820	AZD-7762	CHK inhibitor
2821	calpeptin	calpain inhibitor, proteasome inhibitor, tyrosine phosphatase inhibitor
2822	BMS-536924	insulin growth factor receptor inhibitor, insulin receptor ligand
2823	tozasertib	Aurora kinase inhibitor, Bcr-Abl kinase inhibitor, FLT3 inhibitor, JAK inhibitor, Abl kinase inhibitor, mitotic inhibitor
2824	HLI-373	MDM inhibitor
2825	zebularine	DNA methyltransferase inhibitor, cytidine deaminase inhibitor
2826	GANT-58	GLI antagonist
2827	PP-2	src inhibitor
[illegible]	[illegible]	[illegible]

2828	sirolimus	mTOR inhibitor, CCR expression inhibitor, cell cycle inhibitor, proteasome inhibitor, protein kinase inhibitor, T cell inhibitor
2829	PTB1	AMPK activator, tyrosine phosphatase inhibitor
2830	U0126	MEK inhibitor, JAK inhibitor, MAP kinase inhibitor
2831	forskolin	adenylyl cyclase activator, Adenylate cyclase stimulant, growth hormone receptor agonist, phosphokinase stimulant
2832	Y-27632	ROCK inhibitor, calcium sensitizer, leucine rich repeat kinase inhibitor
2833	tipifarnib	farnesyltransferase inhibitor, angiogenesis inhibitor, apoptosis stimulant
2834	PD-0325901	MEK inhibitor, MAP kinase inhibitor, protein kinase inhibitor
2835	fostamatinib	syk inhibitor, FLT3 inhibitor
2836	CG-930	JNK inhibitor
2837	PD-98059	MEK inhibitor, MAP kinase inhibitor

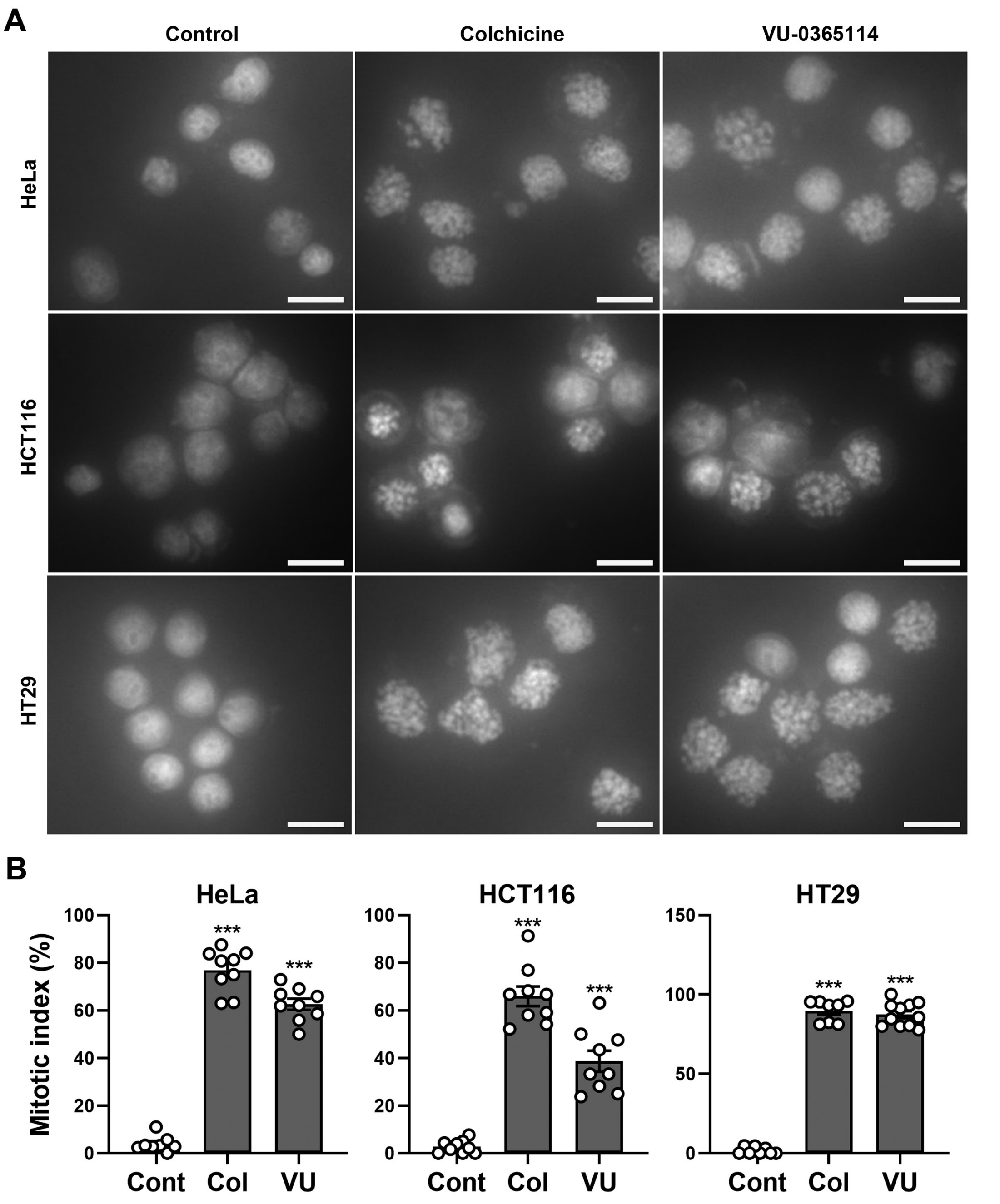

Figure S7. Mitotic index of drug-treated cancer cells. (A) HeLa, HCT116, and HT29 cells were treated with colchicine (100 nM) or VU-0365114 (10 μM) for 24 h. Both floating and adherent cells were harvested, fixed with ethanol, and then stained with Hoechst 33258. Fluorescence was observed under a fluorescence microscope (scale bar: 20 μm). (B) Cells with condensed chromosomes were counted, and the percentage of mitotic cells (mitotic index) was quantified. The error bars are the mean ± SD (*n* = 8~11). Statistical significance, compared to untreated controls (****p*<0.001), was determined using a one-way ANOVA with Tukey's post hoc test.

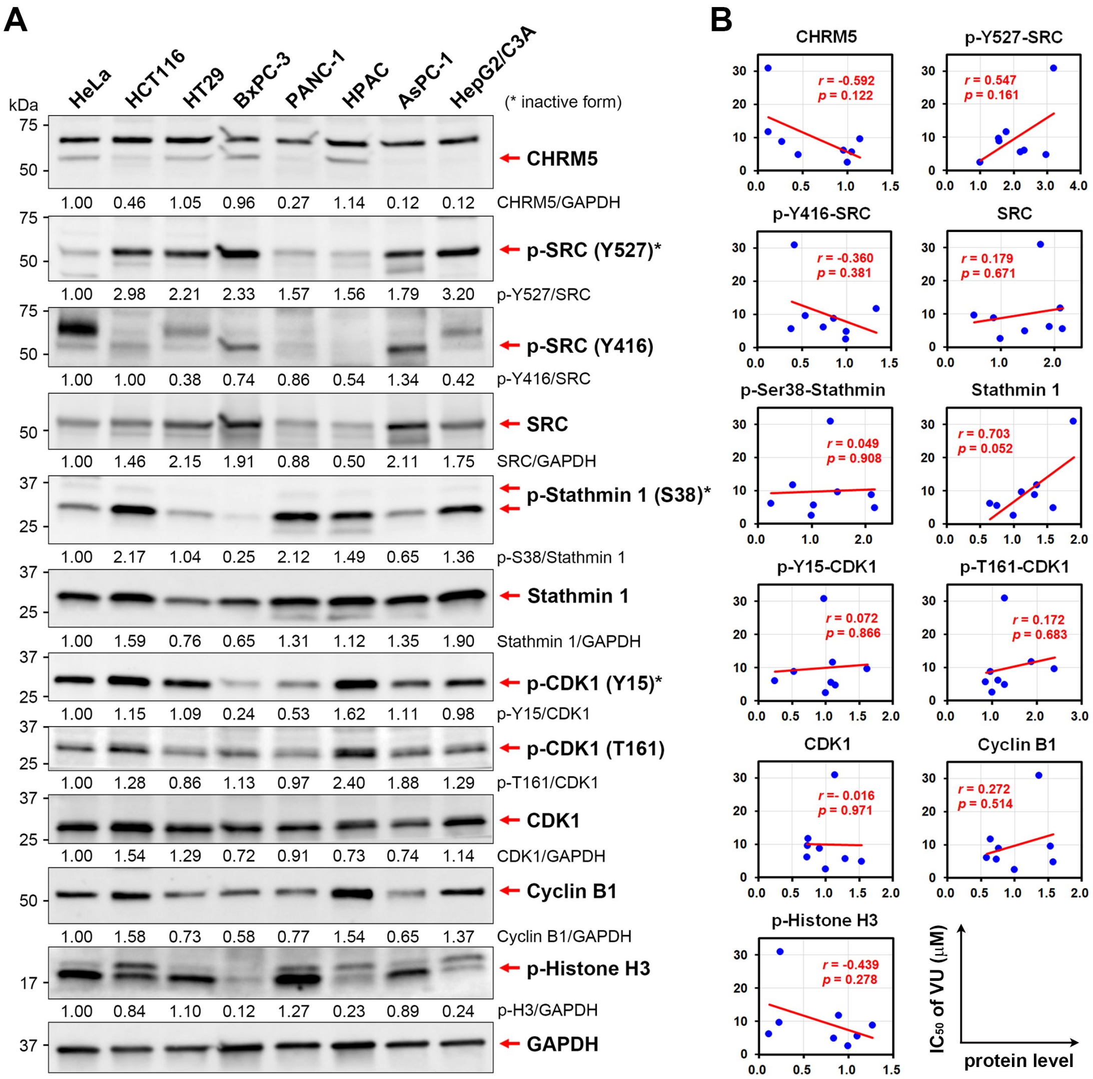

Figure S8. Protein expressions in various human cancer cell lines. (A) The protein expressions of interest in HeLa, HCT116, HT29, BxPC-3, PANC-1, HPAC, AsPC-1, and HepG2/C3A cells were examined using Western blotting. Band intensities were quantified, divided by either GAPDH or the corresponding total protein, and then normalized to the band intensity in HeLa cells. (B) The correlation between the IC_{50} values of VU-0365114 and protein expressions in these cell lines was calculated using Pearson's correlation coefficient (r).

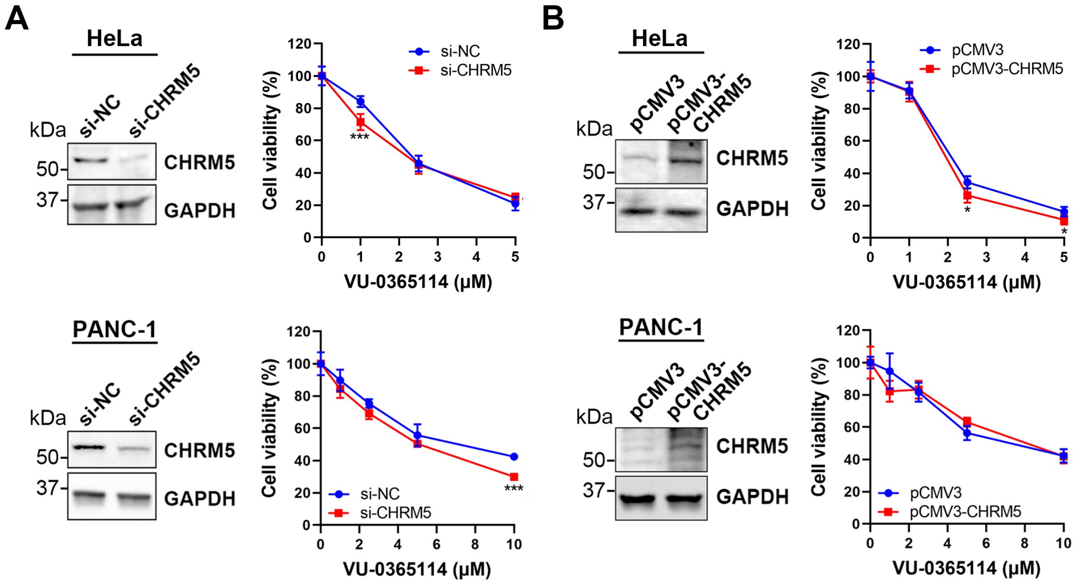

Figure S9. Effect of CHRM5 knockdown or overexpression on the cytotoxicity of VU-0365114 in PANC-1 cells. (A, B) HeLa and PANC-1 cells were transfected with *CHRM5* siRNA in A or plasmid in B for 48 h, and then exposed to VU-0365114 for 72 h. Knockdown or overexpression of M5 mAChR in A or B was confirmed by Western blotting (left panel). Cell viability was examined by an MTT assay (right panel). The error bars are the mean ± SD (n = 5). Statistical significance, compared to the transfection control group at each dose (*p<0.05 and ***p<0.001), was determined using a two-tailed paired Student's t-test.

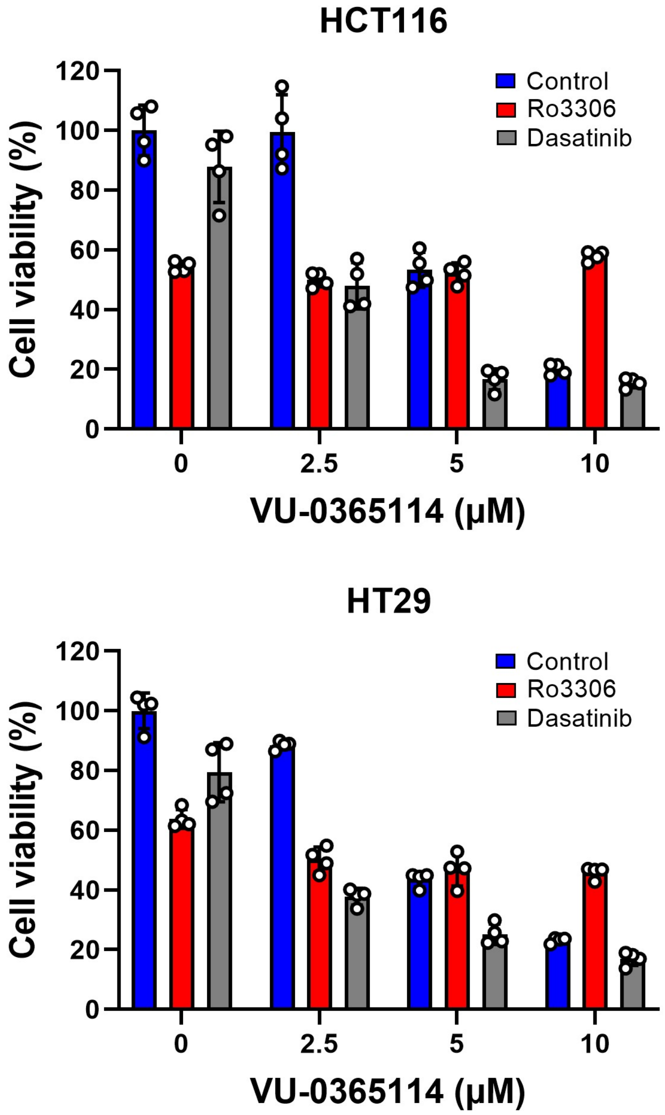

Figure S10. Effect of CDK1 and SRC inhibitors on the cytotoxicity of VU-0365114 in colorectal cancer cells. HCT116 and HT29 cells were treated with various concentrations of VU-0365114 for 72 h in the absence or presence of Ro3306 (5 μM) or dasatinib (100 nM). The cell viability was examined by an MTT assay. The error bars are the mean ± SD (*n* = 5).

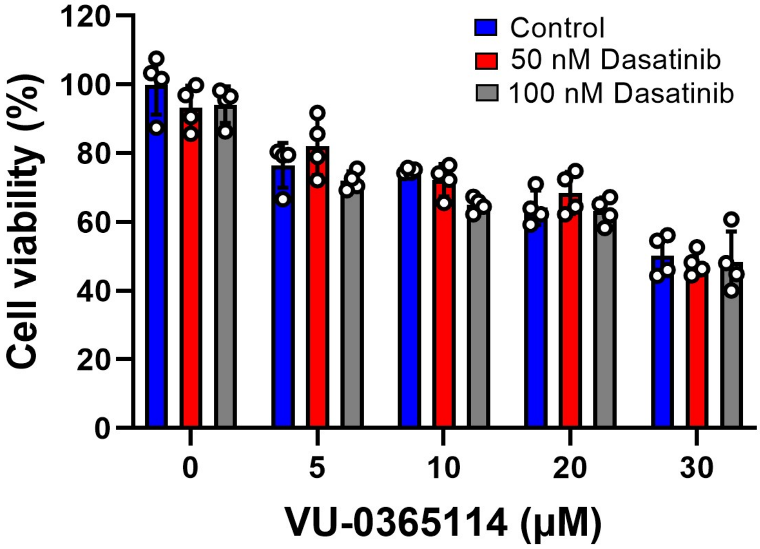

Figure S11. Effect of a SRC inhibitor on the cytotoxicity of VU-0365114 in HepG2/C3A cells. HepG2/C3A cells were treated with various concentrations of VU-0365114 for 72 h in the absence or presence of dasatinib (50 or 100 nM). The cell viability was examined by an MTT assay. The error bars are the mean ± SD (n = 4). Statistical significance, compared to VU-0365114-treated cells at each dose point, was determined using a two-tailed paired Student′s t-test.

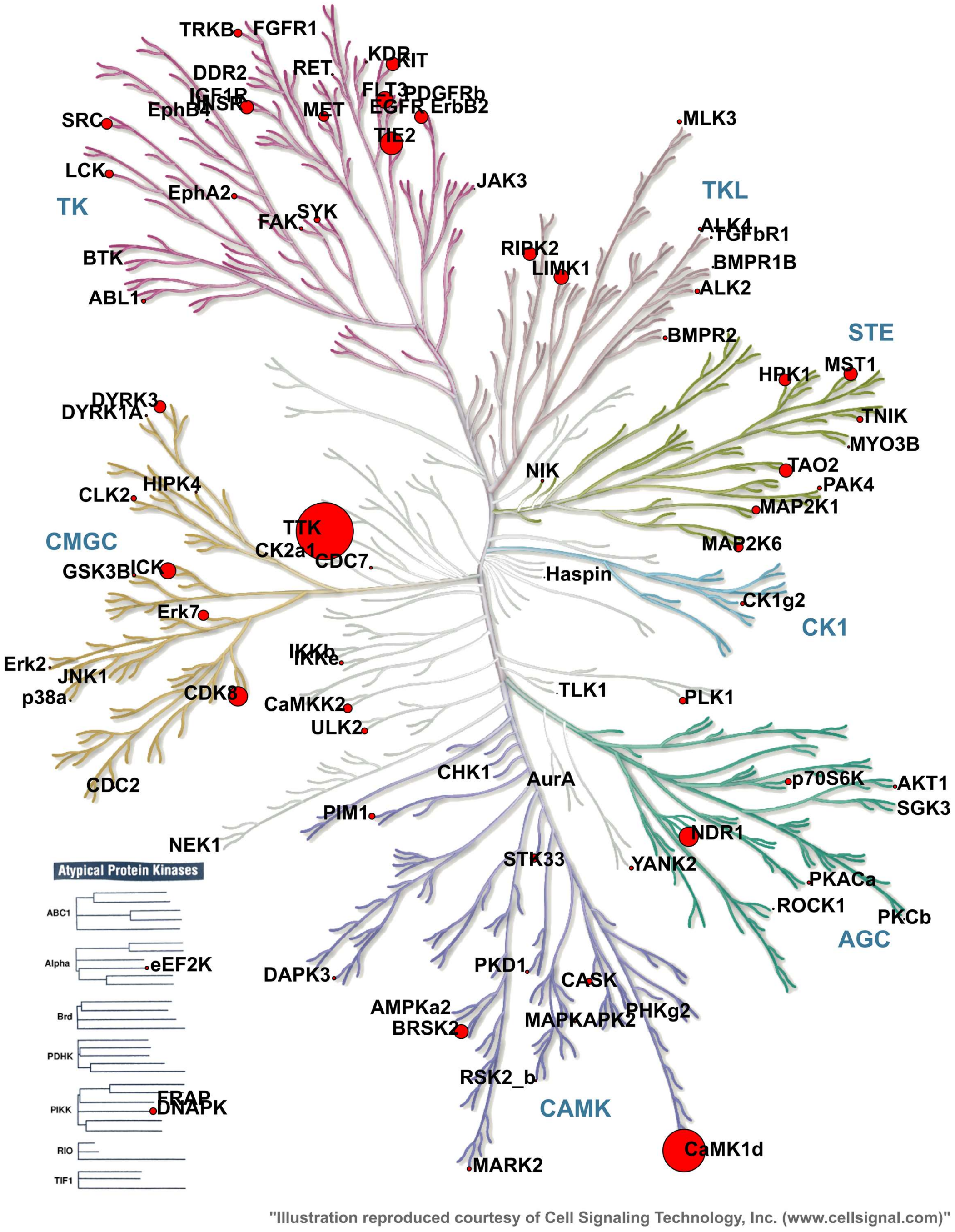

Figure S12. An enlarged image for Figure 10A.

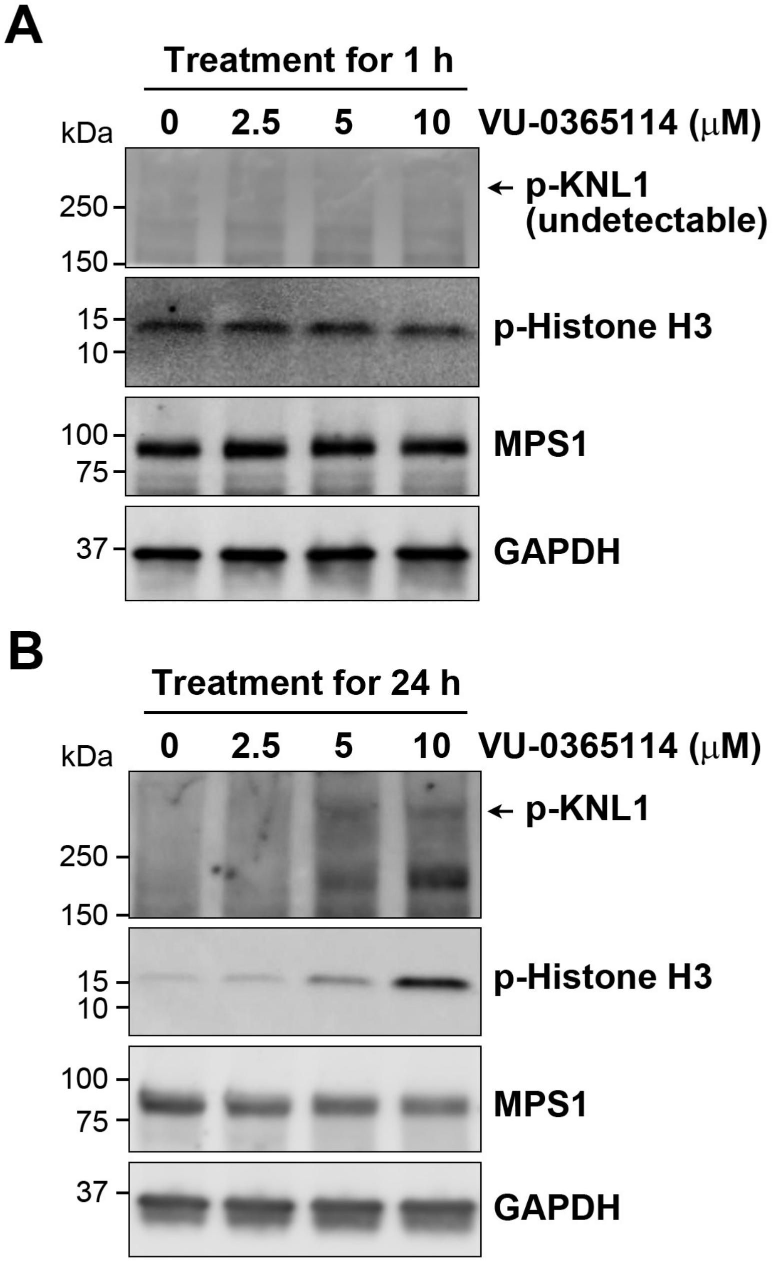

Figure S13. Effect of VU-0365114 on MPS1 activity in HCT116 cells. (A, B) HCT116 cells were treated with the indicated doses of VU-0365114 for 1 h in A and 24 h in B. Then, protein expressions were examined by Western blotting.

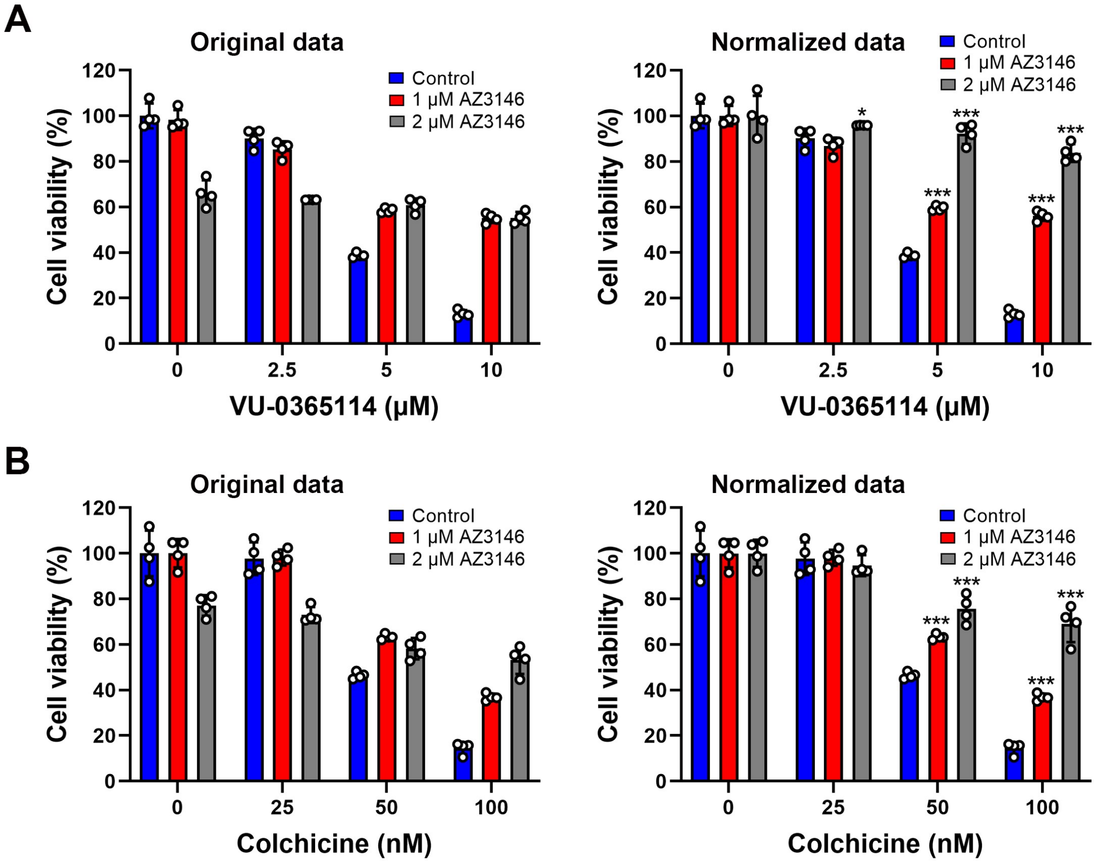

Figure S14. Effect of a MPS1 inhibitor on the cytotoxicity of VU-0365114 or colchicine in HCT116 cells. (A, B) HCT116 cells were treated with various concentrations of VU-0365114 in A or colchicine in B for 72 h in the absence or presence of AZ3146 (1 or 2 μM). The cell viability was examined by an MTT assay. The error bars are the mean ± SD (n = 4). The original data in A were normalized to untreated control or AZ3146. Statistical significance, compared to VU-0365114-treated cells at each dose point (***p<0.001), was determined using a two-tailed paired Student's t-test.
